# Transmembrane protein GRINA modulates aerobic glycolysis and promotes tumor progression in gastric cancer

**DOI:** 10.1186/s13046-018-0974-1

**Published:** 2018-12-12

**Authors:** Dan-Hua Xu, Qing Li, Hao Hu, Bo Ni, Xu Liu, Chen Huang, Zi-Zhen Zhang, Gang Zhao

**Affiliations:** 1 Department of Gastrointestinal Surgery, Ren Ji Hospital, School of Medicine, Shanghai Jiao Tong University, No. 1630, Dong Fang Road, Pu Dong New District, Shang Hai, 200127 Pu Dong, People’s Republic of China; 2 State Key Laboratory of Oncogenes and Related Genes, Shanghai Cancer Institute, Shanghai Jiao Tong University, Shanghai, 200240 People’s Republic of China

**Keywords:** Chromosome 8q24, C-Myc, Proliferation, Apoptosis, Glycolysis

## Abstract

**Background:**

Recent observations indicate a decreased cancer risk in patients with Alzheimer’s disease (AD). AD is a severe neurodegenerative disorder characterized by progressive cognitive decline. The 8q24 region has been shown to be involved in AD aetiology. We aimed to identify and explore the potential oncogenes or antioncogenes on chromosome 8q24.

**Methods:**

We compared expression of genes on Chromosome 8q24 in 32 pairs of samples from The Cancer Genome Atlas (TCGA) database. We conducted bioinformatics analysis of the commonly used gastric cancer databases and performed clinical verification of gastric cancer samples, combined with assessment of biological function both in vitro and in vivo to determine the relationship between upregulated expression of GRINA and gastric cancer progression. We also explored the molecular mechanism of GRINA upregulation and its function in gastric cancer development and progression.

**Results:**

The expression of GRINA in cancer tissues was significantly higher than that in normal tissues. GRINA indicated poor prognosis in gastric cancer. GRINA promoted the proliferation, migration and invasion capacity of gastric cancer cells. GRINA was transcriptionally mediated by c-Myc and promotes cell cycle transition. GRINA knockdown decreased PI3K/Akt/mTOR signaling and glycolytic metabolism in gastric cancer cells. The apoptosis rate was significantly increased in gastric cancer cell lines after knockdown of GRINA. The expression of pro-apoptotic protein Bax was significantly upregulated, whereas the anti-apoptotic protein Bcl-2 was significantly downregulated in GRINA silenced cells.

**Conclusions:**

Human gastric cancers have increased levels of GRINA, which promotes growth of gastric cancer and inhibits tumor cells apoptosis.

**Electronic supplementary material:**

The online version of this article (10.1186/s13046-018-0974-1) contains supplementary material, which is available to authorized users.

## Background

Gastric cancer is associated with significant morbidity and mortality, being the third leading cause of cancer-related deaths globally [1]. A significant number of patients present with advanced disease, thus leading to poor survival. The prognosis for advanced gastric cancer is poor; less than 10–15% of patients with metastases live for more than 2 years [2]. Currently, gastric cancer treatment still depends on classical surgery, chemotherapy, and radiotherapy. Systemic chemotherapy remains the primary mode of treatment for advanced disease and has been shown to improve survival when compared to supportive care; however, even with optimal chemotherapy, the median survival for fit patients treated in first line clinical trials is 9–11 months [3]. Trastuzumab and ramucirumab have resulted in modest improvements in overall survival for patients with HER2-positive gastric cancer and in the second line, respectively [4, 5]. Under the circumstances that these therapies do not make a breakthrough in gastric cancer, there is a need to identify potential novel therapeutic targets.

Recent observations indicate a decreased cancer risk in patients with Alzheimer’s disease (AD) [6]. AD is a severe neurodegenerative disorder characterized by progressive cognitive decline. The 8q24 region has been shown to be involved in AD aetiology [7]. In gastric cancer, copy number alteration (CNA) on Chromosome 8q24.3 is the main mechanism underlying tumorigenesis [8]. We aimed to identify and explore the potential oncogenes or antioncogenes on chromosome 8q24.3. Glutamate Receptor, Ionotropic, N-Methyl D-Aspartate-Associated Protein 1 (GRINA) is located at chromosome 8q24.3 and belongs to the N-Methyl D-Aspartate (NMDA) receptors which are closely associated with tumour progression according to some studies [9]. Blockade of the NMDA receptor in breast cancer or small cell lung cancer can promote apoptosis of tumour cells, and NMDA receptor is also regarded as a potential therapeutic target in ovarian cancer [10]. In primary encephaloma, NMDA2B phosphorylation can prevent the epileptic seizures caused by tumours [11]. In gastric cancer, knockdown of NR2A, which also belongs to NMDA receptor family, induced cell cycle to arrest in the G1 phase and suppressed the proliferation of MKN45 cells [12].

As a transmembrane protein, the domain of GRINA is similar to that of the other five members of the Transmembrane BAX Inhibitor Motif Containing (TMBIM) family, members of which contain the bax inhibitor domain [13]. GRINA is also known as TMBIM3. TMBIM family members inhibit apoptosis through distinct mechanisms. All TMBIM family members have inhibitory activities in different settings of apoptosis including death receptor regulation, modulation of endoplasmic reticulum (ER) calcium homeostasis, ER stress signalling, autophagy, reactive oxygen species production, and so on [14]. Studies have demonstrated that ER stress can trigger GRINA upregulation and protect against ER stress-mediated apoptosis [15]. In our previous studies, GRINA promoted cell proliferation, invasion, and migration, and inhibited cell apoptosis.

B cell lymphoma-2 (Bcl-2) is one of the most valued oncogene in studies related to apoptosis. As important apoptosis regulatory factors, Bcl-2 family members have dual effects, in that, Bcl-2 and Bcl-2 like protein 1 (Bcl-xl) inhibit apoptosis whereas Bcl-2 associated x protein (Bax) and Bcl-2 antagonist/killer 1 (Bak) promote apoptosis [16]. These proteins are generally expressed in human tumours and are closely associated with tumour cell survival and death. A few studies have reported that interactions between TMBIM family members and Bcl-2 family members have a regulatory function on cell death [14], and that TMBIM6 can directly bind Bcl-2 to affect apoptosis. Being a membrane protein structurally similar to TMBIM6, whether GRINA influences gastric cancer cell apoptosis by regulating Bcl-2 family members remains to be verified. Early research demonstrated that GRINA knockdown in AGS and BGC-823 cell lines resulted in downregulation of Bcl-2 and Bcl-xl but upregulation of Bax and Bak, which suggest that GRINA could regulate gastric cancer cell apoptosis through Bcl-2 family members.

## Materials and methods

### Patients

This retrospective analysis includes 569 patients with resected primary gastric cancer who were treated at Ren Ji Hospital, School of Medicine, Shanghai Jiao Tong University from 2005 to 2011. Tumours were assigned by tumour pathology, node, and metastasis stage as defined by the American Joint Committee on Cancer (7th edition). For each case, the diagnosis was confirmed by a review of the H&E stained slides, and a representative block from each specimen was chosen for immunohistochemical (IHC) analysis. Patient characteristics were obtained from the Ren Ji Hospital, School of Medicine, Shanghai Jiao Tong University electronic medical records. The study was approved by the Research Ethics Committee of Ren Ji Hospital, School of Medicine, Shanghai Jiao Tong University and was carried out in accordance with the ethical standards of the World Medical Association’s Declaration of Helsinki. Signed informed consent was obtained from all the patients included in this study. The inclusion criteria for our study were as follows: 1) an obvious pathologic diagnosis of gastric cancer; 2) primary gastric cancer cases without a history of other solid tumours; 3) accepted radical surgery treatment without residual tumour; 4) no exposure to chemotherapy, radiotherapy, or other anti-cancer therapies before surgery; and 5) availability of complete clinicopathologic and follow-up data.

### Immunohistochemical staining and evaluation

In total, 569 gastric cancer cases, diagnosed pathologically and treated from 2005 to 2011, were retrospectively identified from the hospitalization archives of the Department of Gastrointestinal Surgery, Ren Ji Hospital, School of Medicine, Shanghai Jiao Tong University. The paraffin-embedded tissue samples of these patients were used for tissue microarray construction and immunohistochemical staining. Briefly, after tissue sections were deparaffinised, rehydrated with graded ethanol, incubated with 0.3% hydrogen peroxide for 30 min, and blocked with 10% bovine serum albumin (BSA) (Sangon, Shanghai, China), slides were first incubated overnight with an antibody against GRINA (dilution 1:50, ABGENT, AP13558c) at 4 °C, and then labelled with an HRP-conjugated (rabbit) secondary antibody (ThermoFisher Scientific, USA) at room temperature for 1 h. Finally, positive staining was visualized using diaminobenzidine (DAB) substrate liquid (Gene Tech, Shanghai), followed by counterstaining with haematoxylin. All sections were observed and photographed with a microscope (Carl Zeiss, Germany). Scoring was performed based on the ratio of positively stained cells: 0–5% scored 0; 6–35% scored 1; 36–70% scored 2; more than 70% scored 3, and staining intensity: no staining scored 0, weakly stained scored 1, moderately stained scored 2 and strongly stained scored 3. The final score was determined using the percentage of the positive cell score × staining intensity score as follows: “–” for a score of 0–1, “+” for a score of 2–3, “+ +” for a score of 4–6, and “+ + +” for a score of > 6; low expression was defined as a total score < 4 and high expression with a total score ≥ 4. These scores were determined independently by two senior pathologists in a blinded manner.

### Cell culture and reagents

Human gastric cancer cell lines SGC-7901, MGC-803, BGC-823, MKN45, HGC27, AGS, N87, and GES-1 were all maintained at Shanghai Cancer Institute, Ren Ji Hospital, School of Medicine, Shanghai Jiao Tong University. Cells were cultured in the suggested medium according to American Type Culture Collection (ATCC, Manassas, VA) protocols, supplemented with 10% (*v*/v) foetal bovine serum (FBS) and 1% antibiotics at 37 °C in a humidified incubator under 5% CO_2_ conditions.

### Quantitative real-time PCR

Total RNA was extracted using Trizol reagent (Takara), and reverse transcribed with the PrimeScript RT-PCR kit (Takara) according to the manufacturer’s instructions. Real-time PCR analysis was performed using the SYBR Premix Ex Taq (Takara) on a 7500 Real-time PCR system (Applied Biosystems) at the recommended thermal cycling settings: one initial cycle at 95 °C for 2 min followed by 40 cycles of 10 s at 95 °C and 34 s at 60 °C. Relative mRNA expression was calculated using the 2^(-ΔCT)^ method and normalized to 18S mRNA levels. The primer sequences used in this study are listed in Additional file 1: Table S1.

### Western blotting analysis

Cell lysis was performed using a total protein extraction buffer (Beyotime, Shanghai, China) and protein concentration was measured using a BCA Protein Assay Kit (Pierce Biotechnology). Cell lysates were separated by 6–12% SDS-PAGE and transferred to a PVDF membrane. After blocking with 5% skimmed milk, the membrane was probed with one of the following primary antibodies: GRINA (1:1000, ABGENT, AP13558c), β-ACTIN (1:1000, Servicebio, GB13001–3), Bcl-2 (1:1000, Cell Signaling Technology, #4223), Bax (1:1000, Cell Signaling Technology, #5023), Cleaved-caspase3 (1:1000, Cell Signaling Technology, #9664), Cleaved-caspase7 (1:1000, Cell Signaling Technology, #8438), Cleaved-caspase9 (1:1000, Cell Signaling Technology, #9505), Cyclin D1 (1:10000, Abcam, ab134175), Cyclin E (1:1000, Abcam, ab3927), Akt (1:1000, Cell Signaling Technology, #4685), p-Akt (1:2000, Cell Signaling Technology, #4060), P70S6K (1:1000, Cell Signaling Technology, #9202), p-P70S6K (1:1000, Cell Signaling Technology, #9204), 4EBP1 (1:1000, Cell Signaling Technology, #9644), p-4EBP1 (1:1000, Cell Signaling Technology, #2855), AMPK (1:1000, Cell Signaling Technology, #2532), p-AMPK (1:1000, Cell Signaling Technology, #2535), and respective species-specific secondary antibodies (ThermoFisher Scientific). The bound secondary antibodies were detected using the Odyssey imaging system (LI-COR Biosciences, Lincoln, NE).

### Cell viability assay

Cell viability was measured using a Cell Counting Kit-8 (CCK-8, Dojindo Molecular Technologies, Japan). Briefly, cells were seeded into 96-well plates at 5000 cells per well and incubated overnight. At indicated times, 10% (*v*/v) CCK-8 was added to the culture medium and incubated for 1 h; cell viability was monitored by measuring absorbance at 450 nm using a Power Wave XS microplate reader (BIO-TEK). The experiment was performed in quintuplicate and repeated twice.

### Wound healing assay

Cells were seeded in 6-well plates in complete RPMI 1640 medium. The monolayers were scratched with a 200 μl plastic pipette tip to create a uniform wound. The monolayers were then washed with 1 × PBS and incubated in culture medium without FBS. The wound margin distances between the two edges of the migrating cell sheets were photographed after scratching using phase-contrast microscopy. All experiments were performed in triplicate.

### Transwell experiment

The invasive potential of gastric cancer cells was measured by a Transwell assay (Corning, NY, USA) according to the manufacturer’s instructions. For the migration assay, 2 × 10^4^ cells in 200 μl medium were seeded into the upper chamber of the Transwell inserts. RPMI 1640 medium containing 10% (*v*/v) FBS was added to the bottom chamber. Cells were incubated at 37 °C and allowed to migrate for 24 h. At the designated time points, the non-invading cells that remained on the upper surface were removed. The migrated cells were fixed with 4% paraformaldehyde and stained with 0.5% crystal violet. The number of cells on the lower surface was counted under a light microscope in six random fields. Each experiment was performed in triplicate and repeated twice.

### Cell apoptosis assay

For the apoptosis assay, 10 × 10^5^ cells per well were cultured under serum-deprivation conditions in 6-well plates. Adherent cells were detached with 0.25% trypsin without EDTA in 1 × PBS. Cells were harvested in complete RPMI 1640 medium and centrifuged at 1000 rpm for 5 min. Each of the cells were washed with 1 × PBS, stained with 50 μg/ml propidium iodide (PI) and Annexin V-FITC (BD Pharmingen, USA) following the manufacturer’s instructions. The percentage of Annexin V (+) and PI (−) cells was analysed by flow cytometry.

### Cell cycle assay

Cells were cultured in 6-well plates in complete RPMI 1640 medium. In a different set of experiments, cells were cultured for 48 h after transfection with the shRNA against GRINA or the negative control shRNA. Cells were then harvested by trypsin treatment and fixed with 70% ethanol at 4 °C. Fixed cells were incubated with RNase A for 1 h at 37 °C and stained with propidium iodide for 20 min on ice. The number of cells in each phase of the cell cycle was determined at an excitation of 488 nm and an emission of 585 nm using a flow cytometer (FACSCalibur; Becton Dickinson, San Jose, CA, USA) and analysed with Mod Fit LT software (Verity Software House, Topsham, USA).

### Lentivirus constructs

The sequences of the GRINA short hairpin RNA (shRNA) are shown in Additional file 2: Table S2. The shRNA-containing plasmids and a negative control plasmid were purchased from GenePharma (Shanghai, China). The plasmid containing GRINA-HA and a negative control plasmid were obtained from Asia-Vector Biotechnology (Shanghai, China). Lentivirus packaging was performed in 293 T cells with Lipofectamine 2000 (Invitrogen, Carlsbad, CA, USA) and virus titres were determined according to standard protocols. Cells were infected with 1 × 10^6^ recombinant lentivirus-transducing units in the presence of 6 mg/ml polybrene (Sigma-Aldrich, H9268). When grown at 60–70% confluence, the indicated cells were infected with the supernatant containing viral particles. Stable GRINA knockdown or overexpression cells were then cultured in the presence of 2 mg/ml puromycin (Gibco, A1113802).

### Cell transfection

Cells were plated at 60–70% confluence in 6-well plates. The sequences of the small interfering RNA (siRNA) used are shown in Additional file 3: Table S3. Scrambled siRNA targeting no known gene sequence was used as the negative control. Lipofectamine® RNAiMAX reagent (ThermoFisher Scientific, #13778030) was used to perform siRNA transfection according to the manufacturer’s protocol.

### Animal experiment

Athymic nu/nu mice aged 6 to 8 weeks were kept on a 12-h day/night cycle with free access to food and water. For subcutaneous xenografts in the genetic inhibition study, 2 × 10^6^ sh-Ctrl or sh-GRINA AGS cells in 200 ml Hanks buffered saline were injected subcutaneously in the lower back. For subcutaneous xenografts in the genetic overexpression study, 2 × 106 GRINA-Ctrl or GRINA-OE N87 cells in 200 ml Hanks buffered saline were injected subcutaneously in the lower back. After 4 weeks, the mice were sacrificed, the tumour was isolated and tumour weight was measured. Tumour volumes were calculated as volume = length × width^2^/2.

### Immunofluorescence staining

Paraffin sections (5 mm) of mice tumors were deparaffinized, rehydrated with graded ethanol, and subjected to antigen retrieval. After blocking with 10% BSA, sections were incubated with primary antibodies for 1 h followed by incubation with secondary antibodies for 30 min at room temperature. For staining of Ki67, primary and secondary antibodies were rabbit anti-Ki67 (1:200, Abcam, ab15580), donkey anti-rabbit Alexa Fluor 594 (1:400, Jackson ImmunoResearch, #711–585-152). ApopTag Red In Situ Apoptosis Detection Kit (S7165, Millipore) was used for apoptosis detection. Nuclei were counterstained with DAPI (40,6-diamidino-2-phenylindole dihydrochloride; AppliChem, A4099). Digital images were acquired with fluorescence or confocal microscopes equipped with a digital camera (Nikon).

### Chromatin immunoprecipitation

A Pierce Agarose ChIP Kit was purchased from Thermo Fisher Scientific. Cells were cross-linked with 1% formaldehyde for 10 min at room temperature and quenched by adding glycine (1.25 M). Fixed cells were harvested in SDS buffer with a protease inhibitor and then sonicated to generate DNA fragments of 200–1000 base pairs (bp) in length. The sheared chromatin-lysed extracts were incubated overnight at 4 °C with the anti-c-Myc antibody or control IgG with rotation. After immunoprecipitation (IP) of the cross-linked protein/DNA, the immunocomplexes were reversed to release the DNA. PCR was performed with the input DNA or the immunoprecipitates. The PCR products were separated by agarose gel electrophoresis. The primers used for ChIP analysis are listed in Additional file 4: Table S4.

### Measurement of ECAR and OCR

In vitro cell metabolic alternations were monitored with the Seahorse XF96 Flux Analyser (Seahorse Bioscience), according to the manufacturer’s instructions. Cells were seeded in a XF96-well plate at a density of 2 × 10^4^ per well and allowed to attach overnight, followed by serum starvation for 24 h. For assessment of the real-time glycolytic rate (ECAR), which is an indicator of net proton loss during glycolysis, cells were incubated in unbuffered medium followed by a sequential injection of 10 mM glucose, 1 mM oligomycin (Sigma-Aldrich), and 80 mM 2-deoxyglucose (2-DG, Sigma-Aldrich, D8375). The mitochondrial respiration (OCR) was assessed using a sequential injection of 1 mM oligomycin, carbonyl cyanide 4-(trifluoromethoxy) phenylhydrazone (FCCP, Sigma-Aldrich, C2920), and 2 mM antimycin A and rotenone (Sigma-Aldrich). Oligomycin A is applied to inhibit proton flow through ATP synthase and induce maximal glycolytic metabolism. FCCP, an uncoupler of electron transport, leads to collapse of membrane potential and peak oxygen consumption. To achieve maximal OCR, the concentration of FCCP used in gastric cancer cells was 500 nM. Antimycin A and rotenone were used to inhibit mitochondrial respiration by blocking complex III (Ubiquinone: Cytochrome b-c complex). Both ECAR and OCR measurements were normalized to total protein content and reported as mPH/min for ECAR and pmol/min for OCR. Each datum was determined in triplicate.

### Bioinformatics analysis

The gene expression data for stomach adenocarcinoma (STAD) were downloaded from TCGA, which is maintained by Broad Institute’s TCGA workgroup. The RNA-seq level 4 gene expression data contained log2-transformed RNA-seq by expectation maximization (RSEM) values summarized at the gene level. The data from the Gene Expression Omnibus (GEO) database had the series accession GSE13911.

### Statistical analysis

Data were presented as means ± SD. The SPSS software program (version 19.0, IBM Corporation) was used for statistical analysis. Graphical representations were prepared with GraphPad Prism 5 (San Diego, CA, USA) software. The correlation of GRINA expression with clinicopathological parameters in patients with gastric cancer was evaluated by the chi-square test or Fisher’s exact test. Survival curves were evaluated using the Kaplan-Meier method and differences between survival curves were tested by the log-rank test. All statistical tests were two-sided. The Student’s *t*-test or one-way ANOVA was used for comparison between groups. *P* < 0.05 was considered statistically significant.

## Results

### GRINA expression is elevated in gastric cancer tissues compared with that in normal tissues

**Fig. 1:**
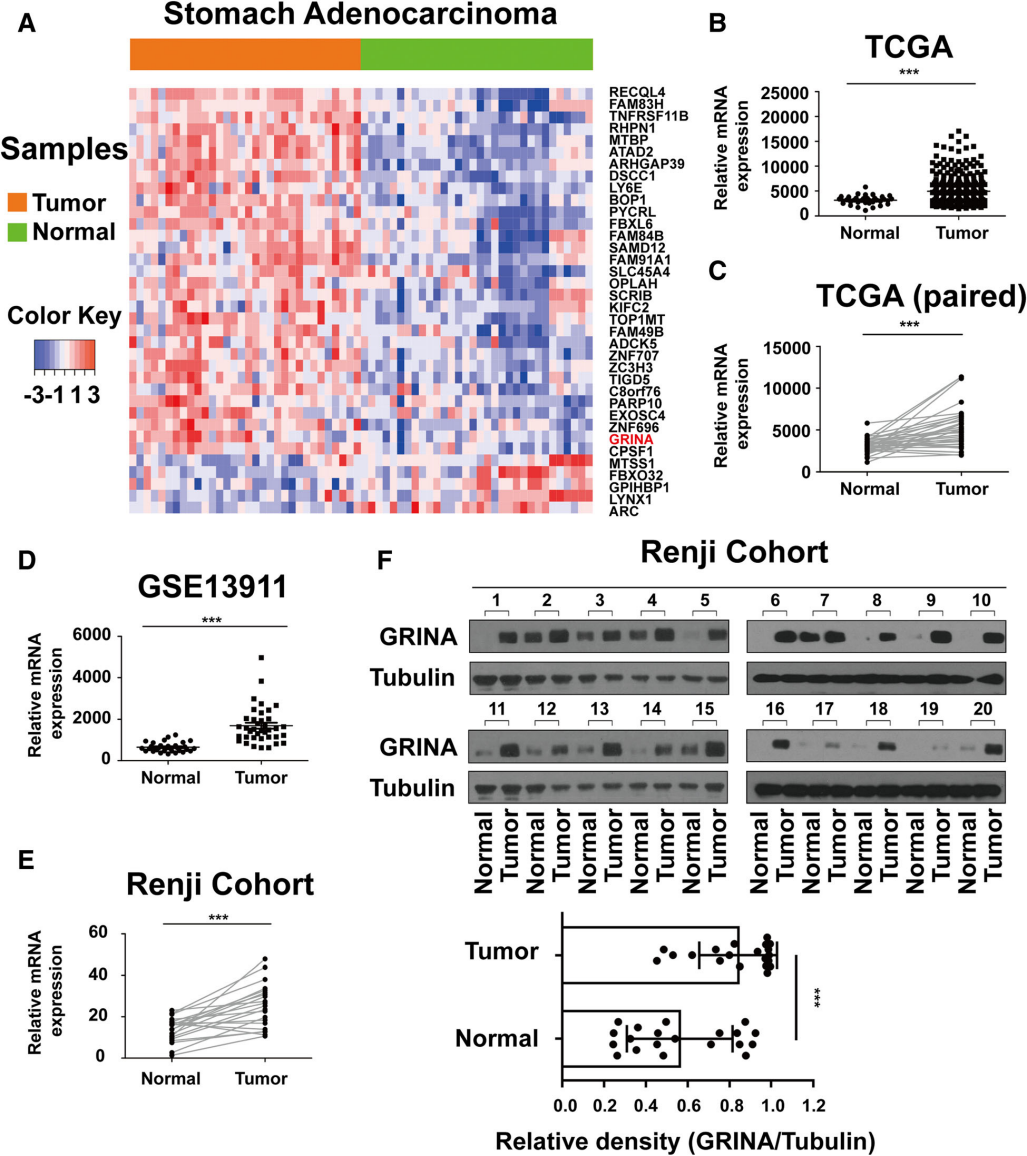
Elevated GRINA expression in gastric cancer. **a** Heatmap of 36 dysregulated genes on chromosome 8q24 (fold change > 1.5 or fold < − 1.5; *q* value < 0.001). Each column represents an individual sample and each row represents one gene. **b** Relative GRINA mRNA expression in 415 gastric cancer samples and 35 normal samples from TCGA database. **c** Relative GRINA mRNA expression in 32 gastric cancer samples and 32 matched normal samples from TCGA database. **d** Relative GRINA mRNA expression in 38 gastric cancer samples and 31 normal samples from GSE13911 database. **e**-**f** mRNA and protein levels of GRINA in 20 pairs of fresh gastric cancer samples were determined by real-time PCR and western blotting. T, tumor sample; N, normal sample. (*N* = 3). Values are mean ± s.d., ***P < 0.001 (Student’s *t*-test)

Comparing gene expression in 32 pairs of cancerous and noncancerous tissues from patients with gastric cancer in The Cancer Genome Atlas (TCGA) database revealed 36 dysregulated genes on chromosome 8q24 (fold change > 1.5 or fold change < − 1.5, q value < 0.001) (Fig. 1a, Additional file 5: Figure S1A), 5 of which were downregulated and 31 upregulated in gastric cancer tissues (Additional file 6: Table S5, Additional file 7: Table S6). GRINA was one of the most significantly upregulated genes on chromosome 8q24.3 (Additional file 8: Figure S1B).

In samples from TCGA database and Gene Expression Omnibus (GEO) database, GRINA expression was found to be significantly increased in gastric cancer samples compared with that in normal samples (matched or not matched) (Figs 1b-1d). However, no such difference was found for the other five members of the TMBIM family (Additional file 9: Figure S1C-G). These results indicated that GRINA was significantly upregulated in gastric cancer.

To validate the results of bioinformatics analysis, 20 fresh gastric cancer samples and their matched normal tissues were collected. The mRNA and protein levels of GRINA was assessed (Figs 1e-1f). The results demonstrated elevated expression of GRINA in tumour tissues consistent with the bioinformatics results.

### GRINA expression shows significant correlation with several clinicopathological parameters and indicates poor prognosis in gastric cancer

Analysis of 876 gastric cancer patients with 33 months of follow-up data in KMplotter database demonstrated that patients with high GRINA expression had worse overall survival (OS) than those with low GRINA expression (Additional file 10: Figure S2), suggesting that GRINA indicates poor prognosis in gastric cancer.

**Fig. 2:**
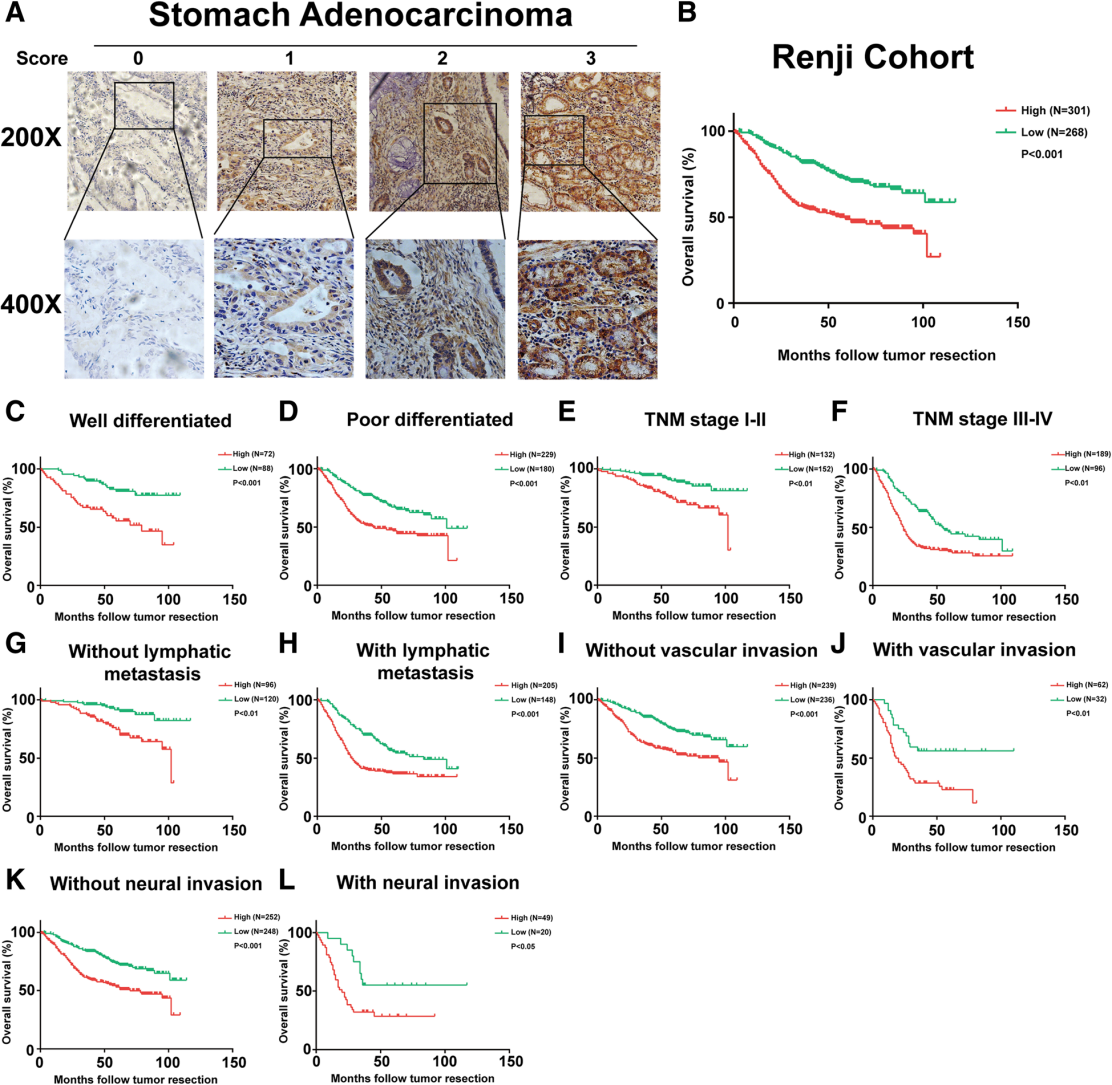
Association between GRINA expression and prognosis in gastric cancer. **a** GRINA protein expression was assessed immunohistochemically in TMAs containing 569 gastric cancer samples. GRINA was located in the plasma membrane and cytoplasm. Scoring was conducted according to the ratio of positively stained cells: 0–5% scored 0; 6–35% scored 1; 36–70% scored 2; and more than 70% scored 3, and staining intensity: no staining scored 0, weakly stained scored 1, moderately stained scored 2, and strongly stained scored 3. **b** The Kaplan-Meier analysis showed that the patients with higher GRINA expression had poorer overall survival. **c**-**l** The Kaplan-Meier curves of overall survival related to histological differentiation, TNM stage (stage I and II, stage III and IV), lymphatic metastasis, vascular invasion, and neural invasion according to the GRINA levels in 569 gastric cancer samples

To further understand GRINA expression in gastric cancer, tissue microarrays (TMA) containing 569 gastric cancer samples were constructed and subjected to IHC staining (Fig. 2a). The correlation analysis between GRINA expression and clinicopathological parameters in gastric cancer demonstrated that GRINA expression was significantly correlated with histological differentiation (*P* = 0.04), T stage (*P* = 0.027), N stage (*P* = 0.03), distant metastasis (*P* = 0.032), blood vessel invasion (P = 0.04) and perineuronal invasion (*P* = 0.005), but not with age and gender (Table 1).

**Table 1:** Correlations between GRINA expression and clinicopathological parameters in 569 gastric cancer patients

Clinicopathological variables	GRINA Expression		*P* Value
	Low(*n* = 268)	High(*n* = 301)	
Age			
< 60	123	176	
≥ 60	145	125	0.06
Sex			
Male	190	201	
Female	78	100	0.595
Histological differentiation			
Well	88	72	
Moderate/Poor	180	229	0.04*
Depth of tumor (pT)			
T1	54	32	
T2	42	38	
T3	62	89	
T4	110	152	0.027*
Nodal stage (pN)			
N0	120	96	
N1	49	56	
N2	48	66	
N3	51	83	0.03*
Distant metastasis (pM)			
M0	267	299	
M1	1	2	0.032*
Blood vessel invasion			
Negative	236	239	
Positive	32	62	0.04*
Perineuronal invasion			
Negative	248	252	
Positive	20	49	0.005**

*P < 0.05 statistically significant difference

We then analysed the correlation between GRINA expression and OS by the Kaplan-Meier method in the aforementioned 569 gastric cancer patients. The Kaplan-Meier analysis of overall survival related to histological differentiation, TNM stage (stage I and II, stage III and IV), lymphatic metastasis, vascular invasion, and neural invasion also suggested that patients with higher GRINA expression had worse prognosis than those with lower GRINA expression (*P* < 0.05) (Figs 2B-2L).

### GRINA silencing significantly inhibits gastric cancer cell growth in vitro and in vivo

**Fig. 3:**
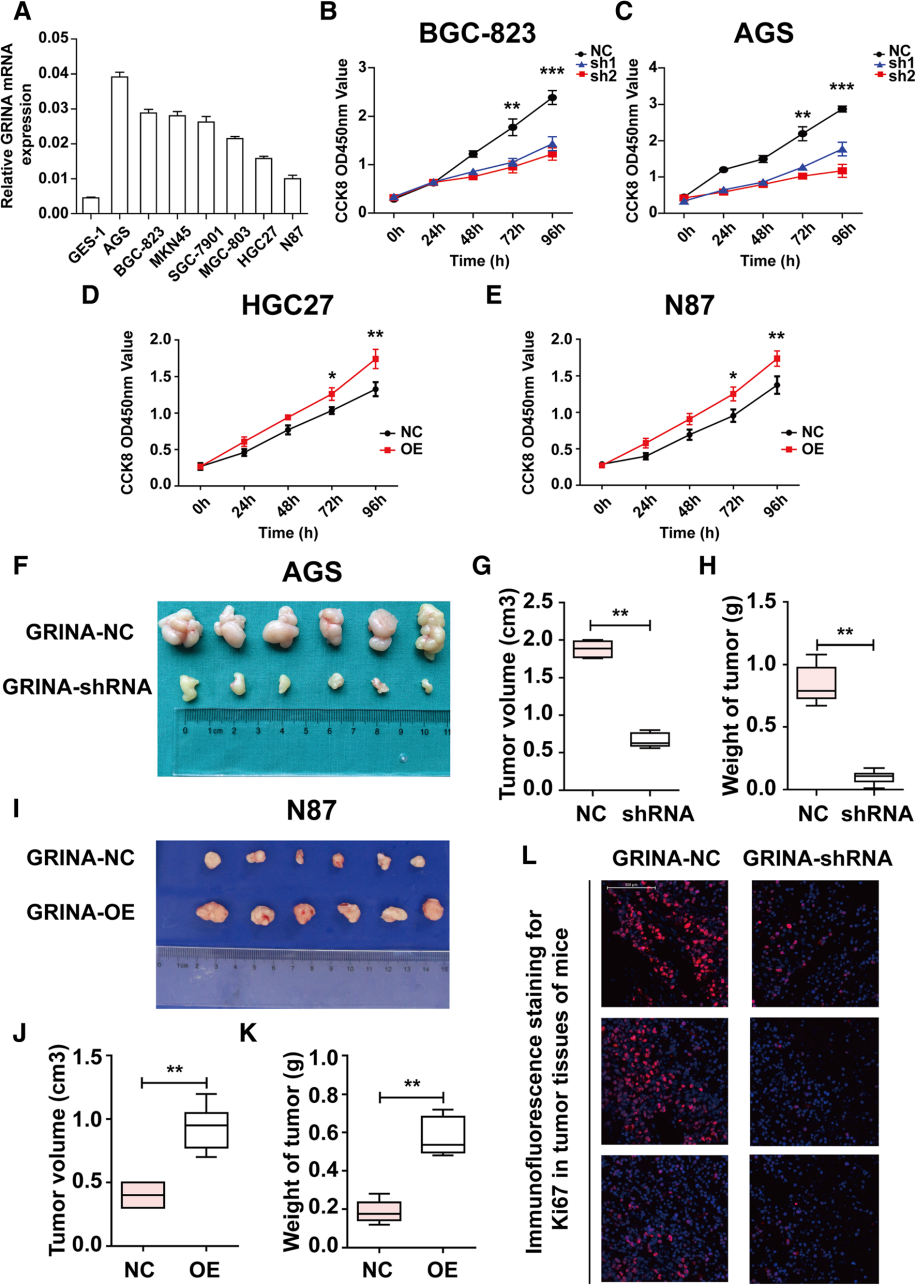
GRINA promoted gastric cancer proliferation in vitro and in vivo. **a** Relative mRNA levels of GRINA in a panel of gastric cell lines (SGC-7901, MGC-803, BGC-823, MKN45, HGC27, AGS, N87, GES-1). GRINA expression was higher in the 7 gastric cancer cell lines than in the normal immortalized gastric mucosal epithelial cell line GES-1 (*N* = 3). **b**-**c** Cell proliferation rates were measured using CCK8 assay in BGC-823 and AGS cells following GRINA silencing and the results showed that GRINA knockdown significantly inhibited the growth of gastric cancer cells (N = 3). **d**-**e** Cell proliferation rates were measured using CCK8 assay in HGC27 and N87 cells following GRINA overexpression and the results showed that GRINA overexpression significantly promoted the growth of gastric cancer cells (N = 3). (**f**-**h**) AGS cells stably transfected with GRINA-shRNA or vector-control were respectively injected into nude mice. Tumour weights and sizes were significantly smaller in the shRNA group than in the control group. **i**-**k** N87 cells stably transfected with GRINA-OE or vector-control were respectively injected into nude mice. Tumour weights and sizes were significantly larger in the GRINA-OE group than in the control group. **l** Immunofluorescence staining for Ki67 reaveled that tumor tissues in the control group showed higher positivity rate than those in the shRNA group (*N* = 3). Values are mean ± s.d., *P < 0.05, **P < 0.01, ***P < 0.001 (Student’s *t*-test)

The mRNA levels of GRINA in 7 gastric cancer cell lines (SGC-7901, MGC-803, BGC-823, MKN45, HGC27, AGS, N87) and in a normal immortalized gastric mucosal epithelial cell line (GES-1) were examined by Real-time PCR. We found higher expression of GRINA in the 7 gastric cancer cell lines than in GES-1 (Fig. 3a). The results demonstrated that GRINA expression was higher in AGS and BGC-823 cell lines while lower in HGC27 and N87 cell lines. Therefore, AGS, BGC-823, HGC27 and N87 cell lines were selected for further experiments in vitro.

Lentiviral transfection of two short hairpin RNAs (shRNA) in AGS and BGC-823 cells significantly downregulated the GRINA mRNA levels by over 85% as well as the protein levels (Additional file 11: Figure S3A-D). In addition, cell proliferation was decreased in the shRNA-1 and shRNA-2 groups compared with that in the control groups (Figs. 3b-3c). Then we overexpressed GRINA in HGC27 and N87 cell lines (Additional file 3: Fig. S3E-H). Cell proliferation was increased in the GRINA-OE group compared with that in the control group (Figs. 3d-3e). The results indicated that GRINA remarkably promoted gastric cancer cell proliferation.

Next, we aimed to determine the role of GRINA in tumour growth in vivo. AGS cells stably transfected with GRINA-shRNA or vector-control were respectively injected subcutaneouslly into nude mice. After 4 weeks, we found that tumour weights and sizes were significantly smaller in the shRNA group than in the control group (*P* < 0.01) (Figs. 3F-3H). Then N87 cells stably transfected with GRINA-OE or vector-control were also injected subcutaneouslly into nude mice. Instead, tumour weights and sizes were markedly larger in the GRINA-OE group than in the control group 4 weeks later (P < 0.01) (Figs. 3I-3K) Immunofluorescence staining for Ki67 reaveled that tumor tissues in the control group showed higher positivity rate than those in the shRNA group (Fig. 3L).

### GRINA knockdown inhibits cell migratory and invasive capacity

**Fig. 4:**
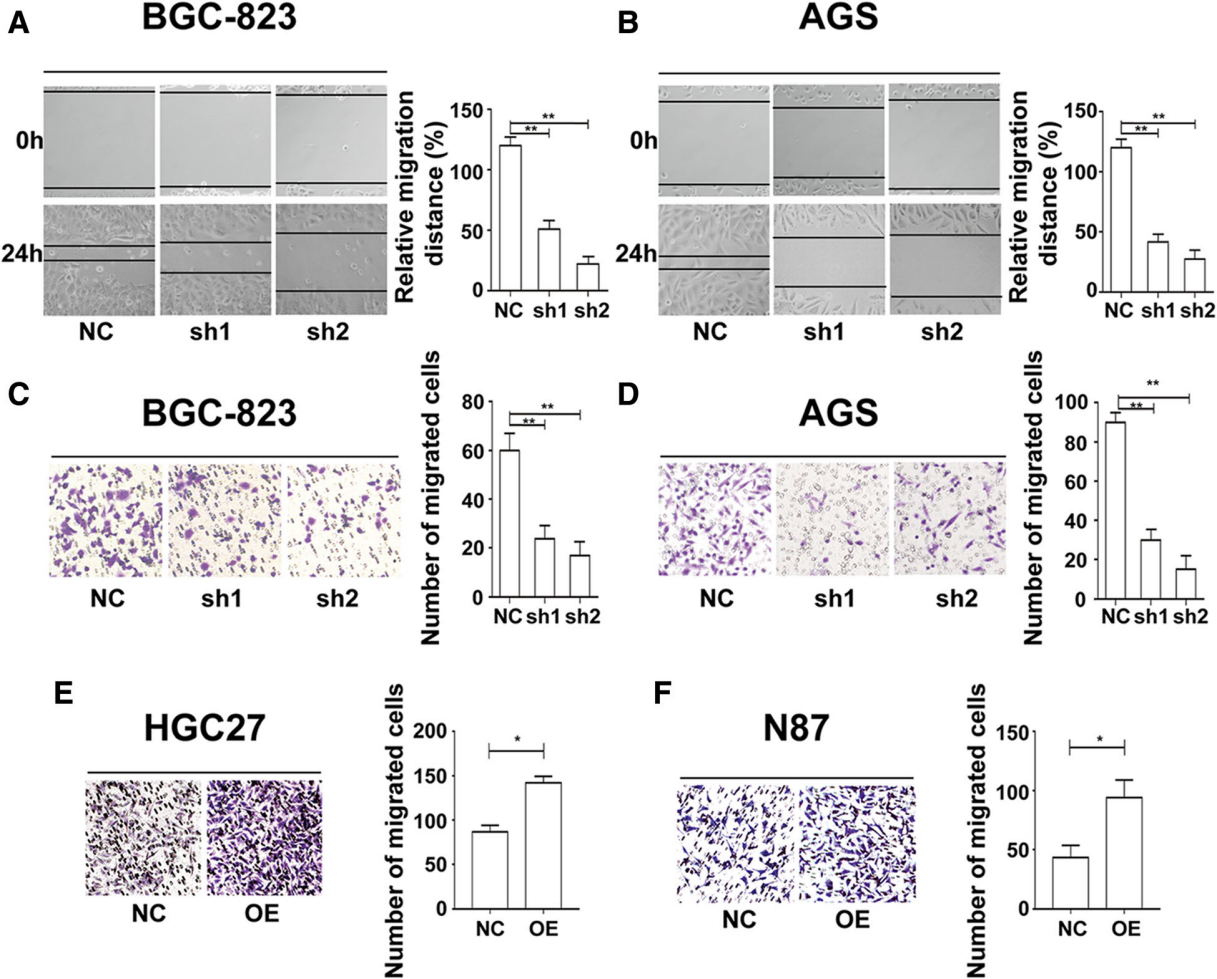
GRINA enhanced the migration and invasion ability of gastric cancer cells. **a**-**b** Scratch wound assays revealed that the migration distance was decreased in GRINA siRNA group compared with that in control group after cells were cultured in medium without FBS for 24 h (N = 3). **c**-**d** Transwell assay showed that GRINA-depleted cells had less migratory and invasive capacity in BGC-823 and AGS cells (*N* = 3). **e**-**f** Transwell assay showed that GRINA-overexpressed cells had enhanced migratory and invasive capacity in HGC27 and N87 cells (*N* = 3). Values are mean ± s.d., *P < 0.05, **P < 0.01 (Student’s *t*-test)

To determine the influence of GRINA on cell migratory and invasive capacity, we performed scratch wound assays. In both AGS and BGC-823 cells, cell migration distances were shorter in the shRNA groups than that in the control groups. The results demonstrated that cell migration and invasion were restrained upon GRINA knockdown (Figs. 4A-4B).

Next, the results of transwell experiments showed that the number of migrated cells was significantly reduced after GRINA was knocked down in AGS and BGC-823 cell lines (Figs. 4C-4D). On the contrary, the number of migrated cells was remarkably increased after GRINA was overexpressed in HGC27 and N87 cells (Figs 4E-4F). These data suggested that GRINA could promote cell migratory and invasive capacity.

### GRINA is transcriptionally mediated by c-Myc and promotes cell cycle transition

**Fig. 5:**
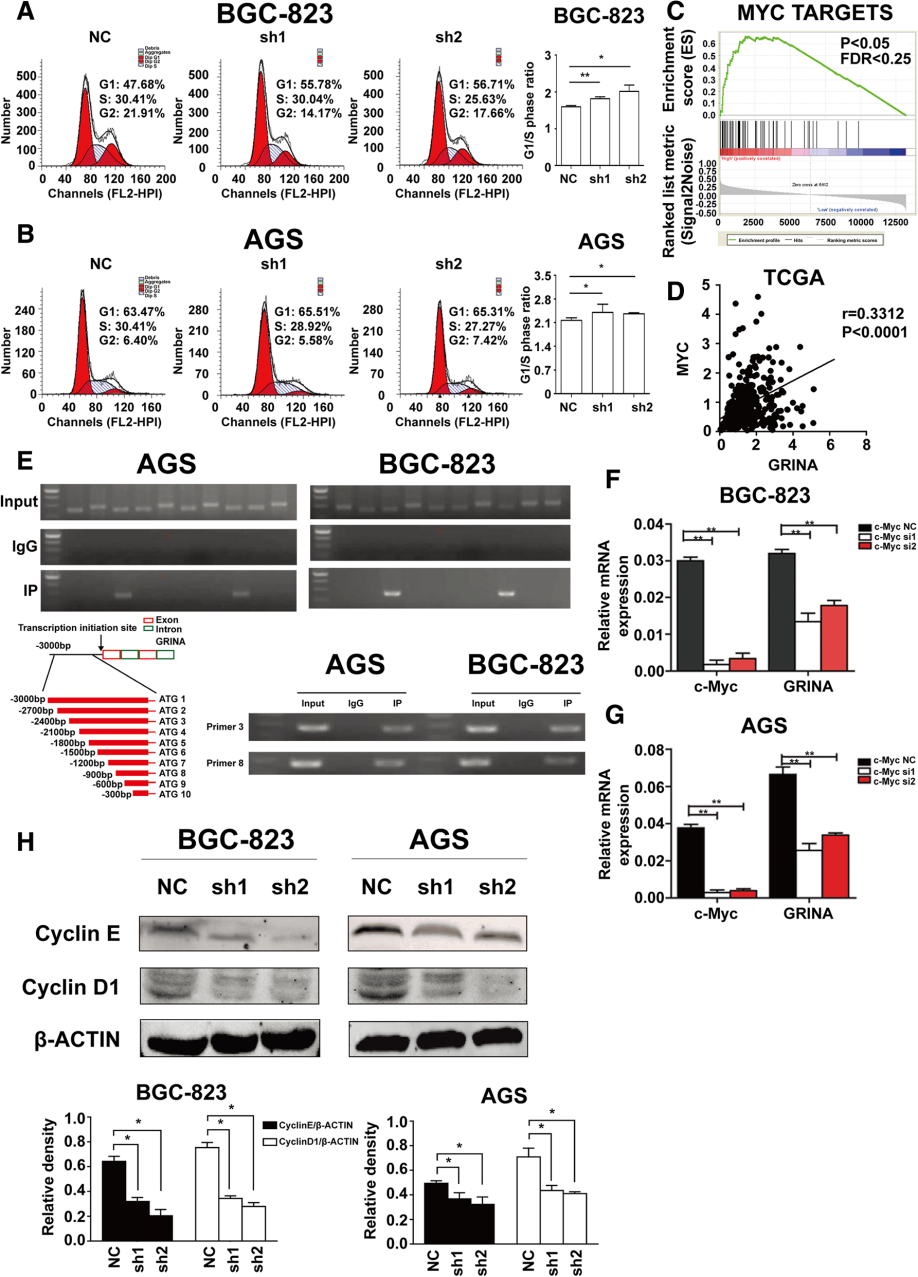
GRINA was transcriptionally mediated by c-Myc and promoted cell cycle transition. **a**-**b** Cell cycle assay indicated that the proportion of cells in G1/S phase was much larger in the shRNA groups than in the control groups (*N* = 3). **c** GSEA pathway analysis using TCGA database showed that GRINA expression was related to Myc targets alteration. **d** Pearson’s correlation coefficient revealed a positive correlation between GRINA and Myc expression in TCGA database. **e** A ChIP assay was performed to confirm the potential c-Myc binding site in the GRINA promoter region. In total, 10 pairs of primers were constructed according to the promoter of GRINA. **f**-**g** Knockdown of c-Myc in BGC-823 and AGS cells decreased GRINA expression significantly (*N* = 3). (H) CyclinD1 and CyclinE were measured by western blotting analysis after GRINA was silenced in BGC-823 and AGS cell lines (*N* = 3). Values are mean ± s.d., *P < 0.05, **P < 0.01 (Student’s *t*-test)

To further understand the mechanism by which GRINA promoted gastric cancer cell proliferation, we added 1 mM thymidine to each cell group for 24 h and cultured the cells in RPMI 1640 medium without FBS for another 24 h, and then counted the proportion of cells in each cell cycle phase. The results demonstrated that the proportion of cells in G1/S phase was larger in the shRNA groups than in the control groups (Figs. 5A-5B), indicating that GRINA knockdown induced cell cycle arrest in the G1 phase and suppressed cell proliferation.

Oncogenes can be activated by factors such as transcription factor regulation, chromosome translocation, genome copy number amplification, and point mutation [17]. We inferred that GRINA upregulation in gastric cancer was mainly induced by transcription factors. Thus, we searched for putative transcription factors that possibly bound the promoter of GRINA in the JASPAR (http://jaspar.genereg.net/) database and found a few transcription factors that probably regulate GRINA expression (Additional file 11: Table S7). The oncogene c-Myc aroused our interest [18]. Gene set enrichment analysis (GSEA) of TCGA data using the Hallmarks gene sets also showed that GRINA expression was related to Myc targets alteration (Fig. 5C). Additional analysis revealed a positive correlation between GRINA and Myc expression in TCGA database (Fig. 5D). We then designed 10 pairs of primers based on the 0–3000 bp nucleotide sequence in the GRINA promoter. The results of ChIP analysis indicated that c-Myc can bind the GRINA promoter both at the − 2400 bp to − 2100 bp and − 900 bp to − 600 bp stretches (Fig. 5E). To further confirm the ChIP results, we knocked down c-Myc in AGS and BGC-823 cells. Strikingly, GRINA expression was also decreased with decreased c-Myc (Figs. 5F-5G). These results suggested that c-Myc could transcriptionally regulate GRINA expression.

Next, we assessed CyclinD1 and CyclinE, two key members of cell cycle, and found that their expression were decreased when GRINA was knocked down (Fig. 5H). These data suggested that GRINA was transcriptionally mediated by c-Myc and promoted cell cycle transition.

### GRINA facilitates gastric cancer cell survival by modulating aerobic glycolysis

**Fig. 6:**
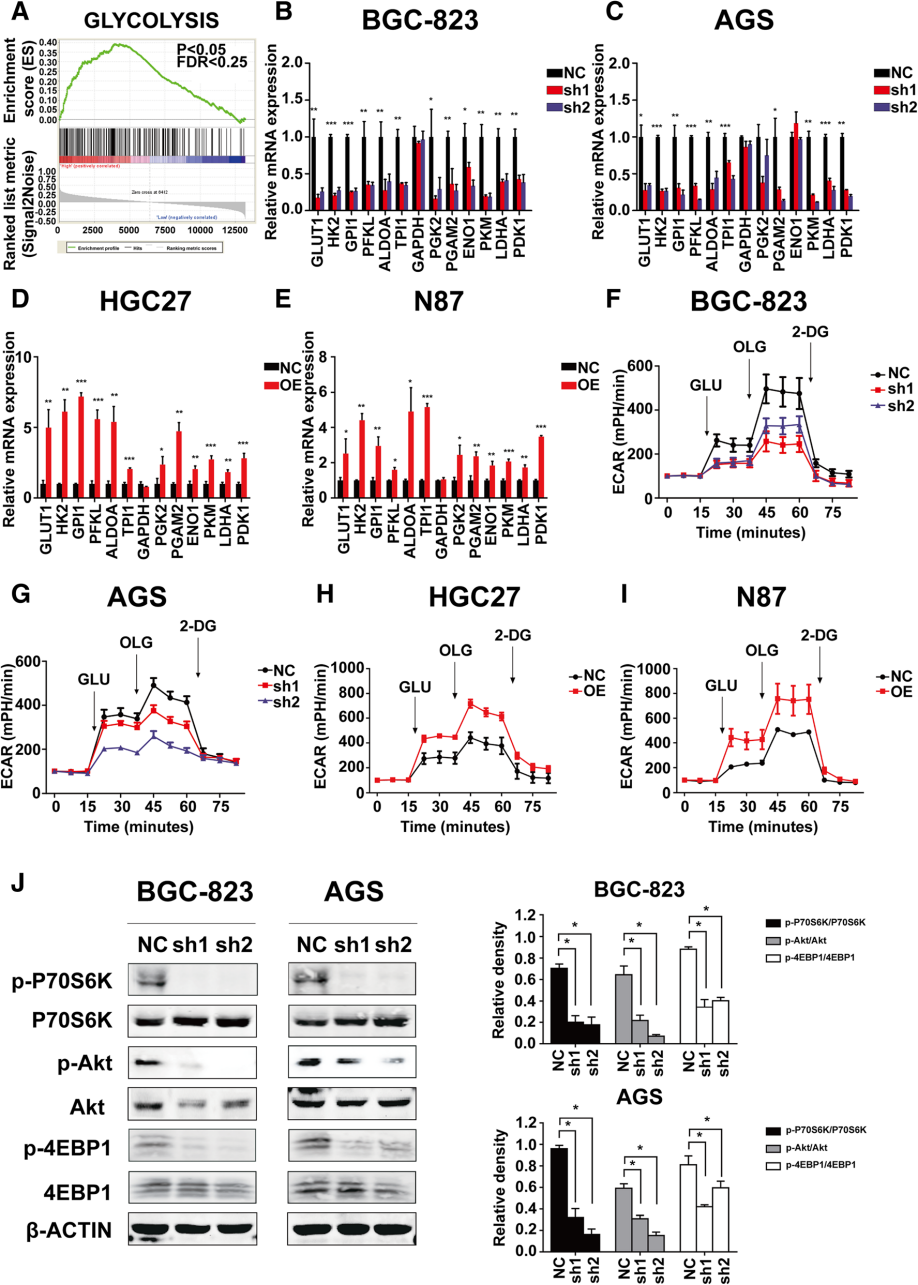
GRINA facilitated gastric cancer cell survival by modulating aerobic glycolysis. **a** GSEA plot based on the gene expression profiles of gastric cancer samples from TCGA database. **b**-**c** Relative mRNA levels of glycolysis-related genes in the control groups and the shRNA groups (*N* = 3). **d**-**e** Relative mRNA levels of glycolysis-related genes in the control groups and the GRINA-OE groups (*N* = 3). **f**-**g** Extracellular acid ratio (ECAR) upon knockdown of GRINA or not in BGC-823 and AGS cells. Glc, glucose; O, oligomycin; 2-DG, 2-deoxyglucose (*N* = 3). (H-I) ECAR upon overexpression of GRINA or not in HGC27 and N87 cells. Glc, glucose; O, oligomycin; 2-DG, 2-deoxyglucose (*N* = 3). **j** After GRINA expression was downregulated, total and phosphorylated Akt, P70S6K, 4EBP1 were measured by western blotting analysis (*N* = 3). Values are mean ± s.d., *P < 0.05, **P < 0.01, ***P < 0.001 (Student’s *t*-test)

The results of GSEA using TCGA database also showed striking alterations in metabolic processes including upregulation of glycolysis and reactive oxygen species pathways (Fig. 6A, Additional file 4: Fig. S4A), which indicated a correlation between GRINA expression and enhanced glycolytic metabolism in gastric cancer. Consistently, real-time qualitative PCR confirmed that several metabolic enzymes involved in glycolysis (Figs. 6B-6E), phosphate pentose pathway (PPP) (Additional file 4: Fig. S4B-C, G-H), hexosamine biosynthesis pathway (HBP) (Additional file 4: Fig. S4D, I), and glutamine (Gln) metabolism (Additional file 4: Fig. S4E-F, J-K) were significantly downregulated by GRINA knockdown and upregulated by GRINA overexpression. To further study the glucose metabolism modulated by GRINA, we measured the extracellular acid ratio (ECAR) and oxygen consumption ratio (OCR) upon GRINA silencing. The results showed that silencing of GRINA attenuated whereas overexpression of GRINA enhanced the glycolytic capability of gastric cancer cells but not oxidative phosphorylation, indicating that GRINA principally affects the glycolytic component of glucose metabolism (Figs 6F-6I, Additional file 4: Fig. S4L-O). To determine the signalling pathway that allows GRINA to affect glycolysis, we determined the activity of AMP-activated protein kinase (AMPK) and mammalian target of rapamycin (mTOR) signalling, which are known to be influenced by the intracellular energy status [19, 20]. GRINA knockdown did not influence phosphorylation of AMPK (Additional file 4: Fig. S4P); on the contrary, GRINA knockdown inhibited the phosphorylation of Akt and the downstream effectors of mTOR, P70S6K and 4EBP1, indicating that the mTOR pathway could be activated by GRINA (Fig. 6J). These findings indicated that GRINA facilitates gastric cancer cell survival by modulating aerobic glycolysis.

### GRINA regulates apoptosis via Bcl-2 family members

**Fig. 7:**
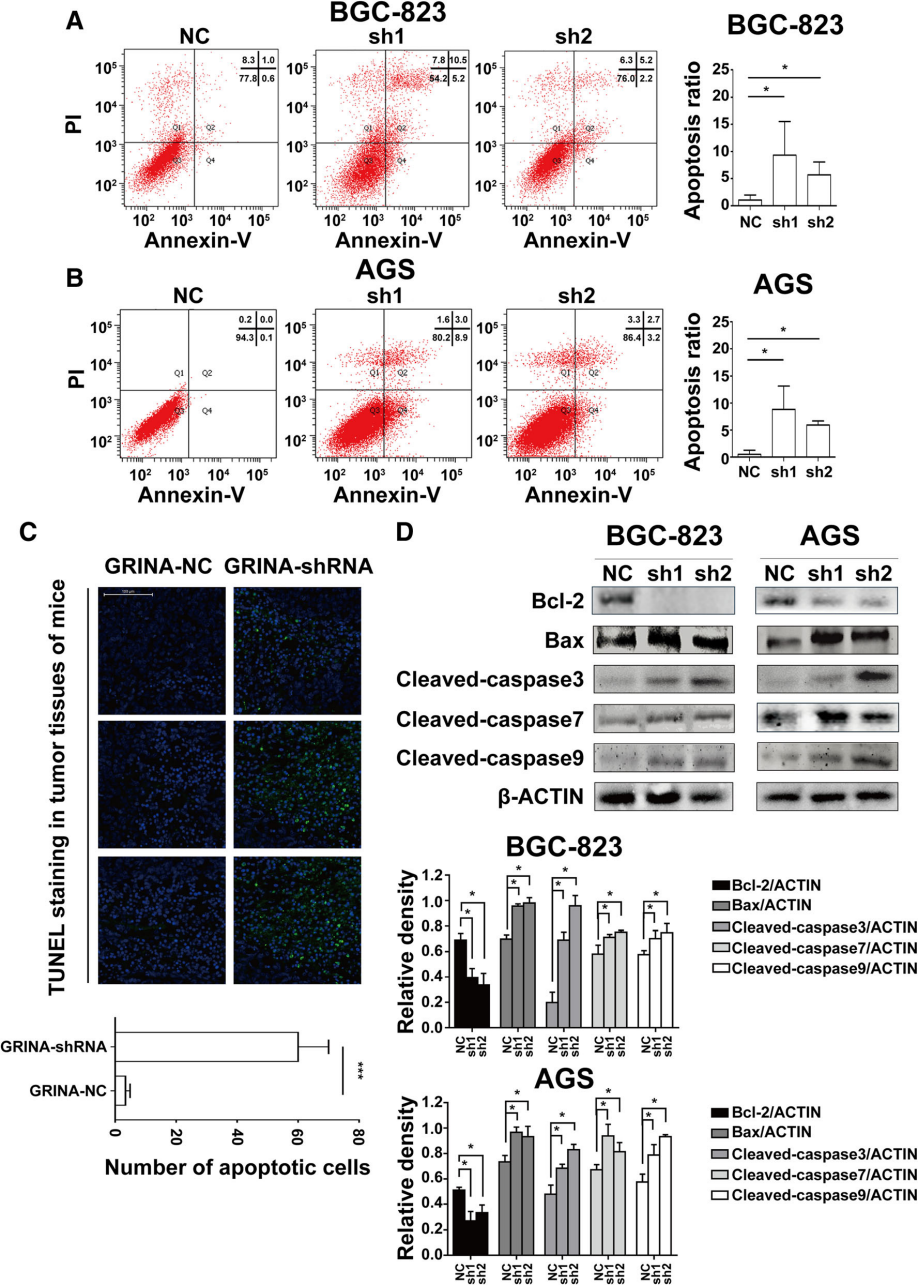
GRINA knockdown promoted cell apoptosis via Bcl-2 family members. **a**-**b** Cell apoptosis assay revealed that apoptosis rates of BGC-823 and AGS cells increased when GRINA was silenced (*N* = 3). **c** TUNEL staining reaveled that mice tumor tissues in the GRINA-shRNA group showed higher apoptosis rate than those in the control group (*N* = 3). **d** Bcl-2, Bax, Cleaved-caspase3, Cleaved-caspase7, Cleaved-caspase9 were measured by western blotting analysis after GRINA was silenced in BGC-823 and AGS cell lines (*N* = 3). Values are mean ± s.d., *P < 0.05, ***P < 0.001 (Student’s *t*-test)

Studies have shown that GRINA could participate in biological processes such as apoptosis and had a close relationship with Bcl-2 family members [16]. Therefore, we examined apoptosis in the GRINA-shRNA and control groups by the Annexin V/PI double labelling method. The percentages of apoptotic cells were obviously augmented in shRNA groups compared with those in control groups after cells were cultured in medium without foetal bovine serum (FBS) for 48 h (Figs 7A-7B). TUNEL staining also reaveled that mice tumor tissues in the shRNA group showed higher apoptosis rate than those in the control group (Fig. 7C). Then we assessed Bcl-2 and Bax expression after GIRNA knockdown in AGS and BGC-823 cells. We found that Bcl-2 expression was significantly decreased whereas Bax expression was obviously increased in both cell lines (Fig. 7D). The levels of Cleaved-caspase3, Cleaved-caspase7, and Cleaved-caspase9 were significantly increased (Fig. 7D). These results suggested GRINA could regulate apoptosis via Bcl-2 family members.

## Discussion

AD is a severe neurodegenerative disorder characterized by progressive cognitive decline during mid to late adult life [21]. Its main neuropathological signs are extracellular depositions of amyloid-β peptide produced by sequential protease cleavage of the amyloid precursor protein, and intracellular neurofibrillary tangles composed of hyper-phosphorylated tau protein [22]. In the field of neurodegeneration, recent observations indicating a decreased cancer risk in patients with AD are particularly intriguing [6]. The 8q24 region has been shown to be involved in AD aetiology [7]. In gastric cancer, CNA on chromosome 8q24.3 is the main mechanism underlying tumourigenesis [8]. We therefore aimed to identify and explore the potential oncogenes or antioncogenes on chromosome 8q24.3.

Many advances in cancer diagnosis and treatment have been made in the past decades, but cancer is still the leading cause of mortality globally [23]. Thus, it is very important to explore the mechanisms underlying tumorigenesis and cancer progression. The occurrence and development of cancer are accompanied by cytogenetic, epigenetic, and tumour microenvironmental (TME) changes, which lead to dysregulation of cell growth and apoptosis, ultimately resulting in tumour formation [24]. Currently, it is widely recognized that activation of oncogenes and subsequent dysregulation of the related signalling pathways are the main causes of tumorigenesis [25].

Gastric cancer is one of the most common cancers in the world. Despite the recent rapid development in medical technology, gastric cancer is still highly lethal especially in developing countries, making it a major public health problem. Gastric cancer is common in Asia, and east and south-central Europe [26, 27]. It is a serious threat to human life and a huge drain on social resources. Gastric cancer diagnosed in the early stage can achieve complete remission through surgeries or endoscopic resections [28]. Nevertheless, most patients feel the symptoms when disease enters progressive stages, leading to difficulties in treatment. The classical therapeutic methods include surgery, radiotherapy, and chemotherapy [29]. Studies have reported that patients with gastric cancer in the late stages had poor prognosis with the five-year survival rate being less than 30% [30, 31]. Recently, therapies targeting human epidermal growth factor receptor-2 (HER-2), epidermal growth factor receptor (EGFR), vascular endothelial growth factor (VEGF) and so on, have provided gastric cancer patients with better survival rates [32, 33]. Trastuzumab and ramucirumab have produced in modest improvements in OS for patients with HER-2 positive gastric cancer and in the second-line setting, respectively [34]. Even so, studies on more effective and selective targeted drugs need to be undertaken. There is a pressing need to explore novel and effectual molecular targets in order to ameliorate the poor prognosis of gastric cancer.

In this study, we analysed GRINA expression in several databases and found that it was significantly elevated in gastric cancer tissues. In the KM-plotter database we found that high GRINA expression indicated a poor prognosis in gastric cancer. IHC staining on TMAs containing 569 gastric cancer samples was performed and the results showed that GRINA expression was significantly correlated with clinicopathological parameters such as classification, TNM stages, lymphatic metastasis, and so on. Survival analysis indicated poor prognosis for patients with high GRINA expression.

We then assessed GRINA expression in gastric cancer cell lines and tissues. GRINA expression was higher in tumour cells than in immortalized gastric mucosal epithelial cells, and was also higher in tumour tissues compared to matched normal tissues. These results were consistent with those of previous studies [35].

We next studied the biological function of GRINA in gastric cancer progression. GRINA promoted gastric cancer cell proliferation, migration, and invasion. Meanwhile, GRINA accelerated tumour growth in nude mice. These results suggest that GRINA is a vital oncogene for gastric cancer development.

Increasing studies indicate that tumorigenesis is closely related to cell cycle regulation [36]. Alteration of the cell cycle is a feature of cancer cells. G1/S phase disruption was found to impact cell proliferation and resulted in carcinogenesis [37]. We found that CyclinD1 and CyclinE, which induce G1 phase transition and promote cell cycle progression, were downregulated upon GRINA knockdown.

In the following work, we probed the molecular mechanisms underlying GRINA mediated gastric cancer tumorigenesis. First, we explored the cause of elevated GRINA expression in gastric cancer. Oncogenes can be activated by transcription factor regulation, chromosome translocation, genome copy number amplification, point mutation and so on [17]. We searched for putative transcription factors that regulate GRINA expression using the JASPAR databases. We found out that c-Myc was the most probable transcription factor for GRINA activation.

C-Myc, located at 8q24.21, is one of the most important genes in cancer research [38, 39]. The protein encoded by c-Myc is multifunctional, phosphorylated in the nucleus, and plays a vital role in cell cycle, apoptosis, and cell transformation [40]. Most importantly, it is a transcription factor that regulates specific genes [41]. Mutation, overexpression, rearrangement, or translocation of c-Myc are closely related to hematopoietic tumours, including leukaemia, lymphoma, Burkitt’s lymphoma, etc. [42, 43]. Many studies have reported that c-Myc promotes the development of gastric cancer. MicroRNA-561 can downregulate c-Myc expression to inhibit tumour cell proliferation and migration [44]. Amplification and expression of c-Myc is significantly correlated with clinicopathological parameters in gastric cancer [45]. In this study, we inferred that c-Myc could activate GRINA expression. A correlation analysis based on 420 gastric cancer samples in TCGA database showed that c-Myc was highly correlated with GRINA expression. However, whether c-Myc could regulate GRINA expression remained to be confirmed. The results of ChIP analysis indicated that c-Myc could directly bind GRINA in its promoter region and regulate GRINA expression at the transcriptional level.

Our study also demonstrates the oncogenic activities of GRINA in gastric cancer cells under metabolic stress. Consistently, GRINA plays a pro-survival and anti-apoptotic role under metabolic stress. Furthermore, the protective roles of GRINA are dependent on glycolysis, as revealed by transcriptomic and metabolomic analyses. The specific transcriptional alteration of glycolytic enzymes shows a high concordance with the actual changes in metabolism.

As mentioned above, the protein domain of GRINA is similar to that of five other members of the TMBIM family. All TMBIM family members have inhibitory activities in different settings of apoptosis, including death receptor regulation, modulation of ER calcium homeostasis, ER stress signalling, autophagy, reactive oxygen species production, etc. Studies have demonstrated that ER stress triggers GRINA upregulation that protects against ER stress-mediated apoptosis. GRINA is probably relevant to Bcl-2 family members [14]. The imbalance between apoptosis and cell proliferation is the key event in tumorigenesis. We found that GRINA knockdown promoted apoptosis in BGC-823 and AGS cells, which suggested that GRINA could inhibit apoptosis.

Recent studies have revealed the mechanisms underlying the functions of the Bcl-2 family. Currently, Bcl-2 family members can be divided into two types. One type inhibits apoptosis and includes Bcl-XL, Bcl-W, Mcl-1, and A1 in mammals, Ced-9 in nematodes, E1B119kD in vaccinia virus, etc.; the other type promotes apoptosis and includes Bax, Bcl-Xs, Bak, Bik/Nbk, Bid, and Harakiri [46]. Inhibition of apoptosis by Bcl-2 was first observed in blood lymphocytes, and later in some other cells. Bax, which belongs to the Bcl-2 family, is the main apoptotic gene in *Homo sapiens*. Bax can form heterodimers with Bcl-2 and inhibit Bcl-2 function. Studies have found that the ratio of Bax and Bcl-2 is the critical factor to control apoptosis [47, 48]. Therefore, Bax is thought to be one of the most important apoptotic genes. In our work, GRINA knockdown in gastric cancer cells promoted cell apoptosis, and induced downregulation of Bcl-2 and upregulation of Bax, which indicates that GRINA inhibits apoptosis through Bcl-2 family members.

## Conclusions

GRINA is widely expressed in several cancers. Human gastric cancers have increased levels of GRINA, which promotes growth of gastric cancer and inhibits tumor cells apoptosis. This is the first study about GRINA in gastric cancer. At present gastric cancer is still lacking for efficient targeted therapies [33]. Although radical surgeries for gastric cancer in the early stages can cure a proportion of patients, those with advanced gastric cancer are still facing great challenges [49]. Effective biological target therapies are considered to improve survival time and prolong recurrence. Further studies should be conducted based on our research in order to obtain new therapeutic targets against gastric cancer.

## Additional files

Additional file 1: **Table S1.** Primers used in this study. (DOCX 358 kb)

Additional file 2: **Table S2.** shRNA sequence used in this study. (DOCX 208 kb)

Additional file 3: **Table S3.** siRNA sequence used in this study. (DOCX 443 kb)

Additional file 4: **Table S4.** ChIP primers used in this study. (DOCX 509 kb)

Additional file 5: **Figure S1.** Expression of GRINA rather than the other five members of the TMBIM family was increased in gastric cancer. (DOCX 19 kb)

Additional file 6: **Table S5.** Genes on chromosome 8. (DOCX 15 kb)

Additional file 7 **Table S6** Genes differentially expressed on chromosome 8q24. (DOCX 15 kb)

Additional file 8: **Figure S2.** KMplotter database demonstrated that patients with high GRINA expression had worse overall survival (OS) than those with low GRINA expression. (DOCX 19 kb)

Additional file 9: **Figure S3.** Knockdown of GRINA in BGC-823 and AGS cell lines by short hairpin RNA (shRNNA). (XLS 293 kb)

Additional file 10: **Table S7.** Transcription factors predicted from JASPAR database that probably regulate GRINA expression. (XLS 169 kb)

Additional file 11: **Figure S4.** Effects of GRINA knockdown on phosphate pentose pathway, hexosamine biosynthesis pathway and glutamine metabolism. (XLS 448 kb)

## Abbreviations

AD: Alzheimer’s disease
AMPK: AMP-activated protein kinase
Bak: Bcl-2 antagonist/killer 1
Bax: Bcl-2 associated x protein
Bcl-2: B cell lymphoma-2
Bcl-xl: Bcl-2 like protein 1
ChIP: Chromatin immunoprecipitation
CNA: Copy number alteration
DAB: Diaminobenzidine
ECAR: The real-time glycolytic rate
EGFR: Epidermal growth factor receptor
ER: Endoplasmic reticulum
FBS: Foetal bovine serum
GEO: The Gene Expression Omnibus
Gln: Glutamine
GRINA: Glutamate Receptor, Ionotropic, N-Methyl D-Aspartate-Associated Protein 1
GSEA: Gene set enrichment analysis
HBP: Hexosamine biosynthesis pathway
HER-2: Human epidermal growth factor receptor-2
IHC: Immunohistochemical analysis
NMDA: N-Methyl D-Aspartate receptors
OCR: The mitochondrial respiration
OS: Overall survival
PPP: Phosphate pentose pathway
shRNA: Short hairpin RNA
siRNA: Small interfering RNA
TCGA: The Cancer Genome Atlas
TMA: Tissue microarrays
TMBIM: Transmembrane BAX Inhibitor Motif Containing family
TME: Tumour microenvironmental
VEGF: Vascular endothelial growth factor
