# Supplementary material for: Transmembrane protein GRINA modulates aerobic glycolysis and promotes tumor progression in gastric cancer
Source: J Exp Clin Cancer Res. 2018 Dec 12;37:308. doi: 10.1186/s13046-018-0974-1 (PMC6292005; doi:10.1186/s13046-018-0974-1)
Supplement: Supplementary file 1 — Table S1. Primers used in this study. (DOCX 358 kb) [file 13046_2018_974_MOESM1_ESM.docx]

**Table S1. Primers used in this study**

| Gene | Forward sequence (5’-3’) | Reverse sequence (5’-3’) |
| --- | --- | --- |
| GRINA | GGATGATCGCCAGCTTCTAC | GCGAAGATGAAGAGCACCAC |
| c-Myc | GTCAAGAGGCGAACACACAAC | TTGGACGGACAGGATGTATGC |
| 18S | CGCCGCTAGAGGTGAAATTC | TTGGCAAATGCTTTCGCTC |
| GLUT1 | ATTGGCTCCGGTATCGTCAAC | GCTCAGATAGGACATCCAGGGTA |
| HK2 | GTGCCCGCCAGAAGACAT | AGGTCCAAGGCCAAGAAGTC |
| GPI1 | CAAGGACCGCTTCAACCACTT | CCAGGATGGGTGTGTTTGACC |
| PFKL | GGTGCCAAAGTCTTCCTCAT | GATGATGTTGGAGACGCTCA |
| ALDOA | AACTTTCCTCTGCCTAGCCC | GTACAGGCACAGTCGCAGAG |
| TPI1 | AGCTCATCGGCACTCTGAAC | CCACAGCAATCTTGGGATCT |
| GAPDH | CTGGGCTACACTGAGCACC | AAGTGGTCGTTGAGGGCAATG |
| PGK2 | AAACTGGATGTTAGAGGGAAGCG | GGCCGACCTAGATGACTCATAAG |
| PGAM2 | AGAAGCACCCCTACTACAACTC | TCTGGGGAACAATCTCCTCGT |
| ENO1 | GCCGTGAACGAGAAGTCCTG | ACGCCTGAAGAGACTCGGT |
| PKM | ATGTCGAAGCCCCATAGTGAA | TGGGTGGTGAATCAATGTCCA |
| LDHA | ATGGCAACTCTAAAGGATCAGC | CCAACCCCAACAACTGTAATCT |
| PDK1 | CTGTGATACGGATCAGAAACCG | TCCACCAAACAATAAAGAGTGCT |
| G6PD | AACATCGCCTGCGTTATCCTC | ACGTCCCGGATGATCCCAA |
| PGD | GTCAGTGGTGGAGAGGAAGG | CACAGCAGGGTTCTCCAGTT |
| PGLS | CTTCGATCACGCCGAGAG | AGCTCGGGGTTAATGGTGAT |
| TALDO1 | GTGAAGCGTCAGAGGATGGA | GTAGCATCCTGGGGCTTGTA |
| TKT | AACCGCCTACGTATCAGCTC | GACTTGTAGCGCATGGTGTG |
| RPE | CAGGAGCCAATCAGTACACCT | CAAGGCCAACCTGCAATGG |
| RPIA | TCGACACCCAGAGATCGACC | TCCGCCACCCTTGATGAGA |
| GFPT1 | ACAATCGGGAAAGTCAAGATACC | CACCAATCAACAGAGGGCTAC |
| SLC1A5 | TCATGTGGTACGCCCCTGT | GCGGGCAAAGAGTAAACCCA |
| GLUD1 | CTGCAACCATGTGCTGAGTC | TCACATCAGTGCTGTAACGGA |
| GLS | TCTACAGGATTGCGAACGTCT | CTTTGTCTAGCATGACACCATCT |
| GOT1 | ATGGCACCTCCGTCAGTCT | AGTCATCCGTGCGATATGCTC |
| GOT2 | AGCCTTACGTTCTGCCTAGC | AAACCGGCCACTCTTCAAGAC |
| GPT2 | GTGATGGCACTATGCACCTAC | TTCACGGATGCAGTTGACACC |
| PSAT1 | TGCCGCACTCAGTGTTGTTAG | GCAATTCCCGCACAAGATTCT |
