# Supplementary material for: Transmembrane protein GRINA modulates aerobic glycolysis and promotes tumor progression in gastric cancer
Source: J Exp Clin Cancer Res. 2018 Dec 12;37:308. doi: 10.1186/s13046-018-0974-1 (PMC6292005; doi:10.1186/s13046-018-0974-1)
Supplement: Supplementary file 2 — Table S2. shRNA sequence used in this study. (DOCX 208 kb) [file 13046_2018_974_MOESM2_ESM.docx]

**Table S2. shRNA sequence used in this study**

| GRINA shRNA-1 | Sence | CCGGACAGAGCCCAUUACATT |
| --- | --- | --- |
|  | Antisence | UGUAAUGGGCUCUGUCCGGTT |
| GRINA shRNA-2 | Sence | GGUUGUGCGUACAAAGCACTT |
|  | Antisence | GUGCUUUGUACGCACAACCTT |
| GRINA shRNA-3 | Sence | UUAAAUGACAGCUCAGAGGTT |
|  | Antisence | CCUCUGAGCUGUCAUUUAATT |
| Negative-Control | Sence | UUCUCCGAACGUGUCACGUTT |
|  | Antisence | ACGUGACACGUUCGGAGAATT |
