# Supplementary material for: Transmembrane protein GRINA modulates aerobic glycolysis and promotes tumor progression in gastric cancer
Source: J Exp Clin Cancer Res. 2018 Dec 12;37:308. doi: 10.1186/s13046-018-0974-1 (PMC6292005; doi:10.1186/s13046-018-0974-1)
Supplement: Supplementary file 3 — Table S3. siRNA sequence used in this study. (DOCX 443 kb) [file 13046_2018_974_MOESM3_ESM.docx]

**Table S3. siRNA sequence used in this study**

| C-myc siRNA-1 | Sence | AACGAUGAAAUAGAUGGAGTT |
| --- | --- | --- |
|  | Antisence | CUCCAUCUAUUUCAUCGUUTT |
| C-myc siRNA-2 | Sence | CAUUUACCAGCCCGACGAGTT |
|  | Antisence | CUCGUCGGGCUGGUAAAUGTT |
| C-myc siRNA-3 | Sence | CCACAACGUCACACAAUGUTT |
|  | Antisence | ACAUUGUGUGACGUUGUGGTT |
| Negative-Control | Sence | UUCUCCGAACGUGUCACGUTT |
|  | Antisence | ACGUGACACGUUCGGAGAATT |
