# Supplementary material for: Transmembrane protein GRINA modulates aerobic glycolysis and promotes tumor progression in gastric cancer
Source: J Exp Clin Cancer Res. 2018 Dec 12;37:308. doi: 10.1186/s13046-018-0974-1 (PMC6292005; doi:10.1186/s13046-018-0974-1)
Supplement: Supplementary file 4 — Table S4. ChIP primers used in this study. (DOCX 509 kb) [file 13046_2018_974_MOESM4_ESM.docx]

**Table S4. ChIP primers used in this study**

| GRINA primers |  | Sequence | Start and end site |
| --- | --- | --- | --- |
| 1 | F | attggcatcgtcacaagtaac | 1-21 |
|  | R | gggctgggttttggtgggctg | 267-287 |
| 2 | F | cccctgctgttcctccctcc | 290-309 |
|  | R | atgagccactgcgcccggcc | 554-573 |
| 3 | F | aatcccagcactttgggagac | 580-600 |
|  | R | ttttttttagacagagtctca | 820-840 |
| 4 | F | aacaaaaacccaaagtcctt | 841-860 |
|  | R | aaaaaaaaaggcagagtaagg | 1081-1101 |
| 5 | F | tgagacggagtttcgccctt | 1110-1129 |
|  | R | tgggtatggtggctggcacc | 1370-1389 |
| 6 | F | ttttttgagacagagtcaca | 1421-1440 |
|  | R | ccagcactttgggaggccga | 1661-1680 |
| 7 | F | gattacaggcgtaagccaccg | 1681-1701 |
|  | R | tttcaagttacccccagctgg | 1943-1963 |
| 8 | F | accataggtgcaggactcaga | 2060-2080 |
|  | R | ccacagtcaccctttgtac | 2342-2360 |
| 9 | F | ataattgtacattttaaaaca | 2367-2387 |
|  | R | caagaagcctaatcctttag | 2643-2662 |
| 10 | F | acggtcagtcccgggcagctt | 2720-2740 |
|  | R | gggcgagcttcggggtgggag | 2952-2972 |
