# Supplementary material for: Transmembrane protein GRINA modulates aerobic glycolysis and promotes tumor progression in gastric cancer
Source: J Exp Clin Cancer Res. 2018 Dec 12;37:308. doi: 10.1186/s13046-018-0974-1 (PMC6292005; doi:10.1186/s13046-018-0974-1)

**Figure S1.** Expression of GRINA rather than the other five members of the TMBIM family was increased in gastric cancer. (A) Volcano plot of 36 dysregulated genes on chromosome 8q24 (fold change > 1.5 or fold < -1.5; q value < 0.001). (B) GRINA is one of the most significantly upregulated gene on chromosome 8q24.3. (C-G) mRNA expression of TIBIM1, TIBIM2, TIBIM4, TIBIM5, TIBIM6 in 32 gastric cancer samples and 32 matched normal samples from TCGA database. *P < 0.05, **P < 0.01, ***P < 0.001, NS indicates no significance. (Student’s *t*-test).
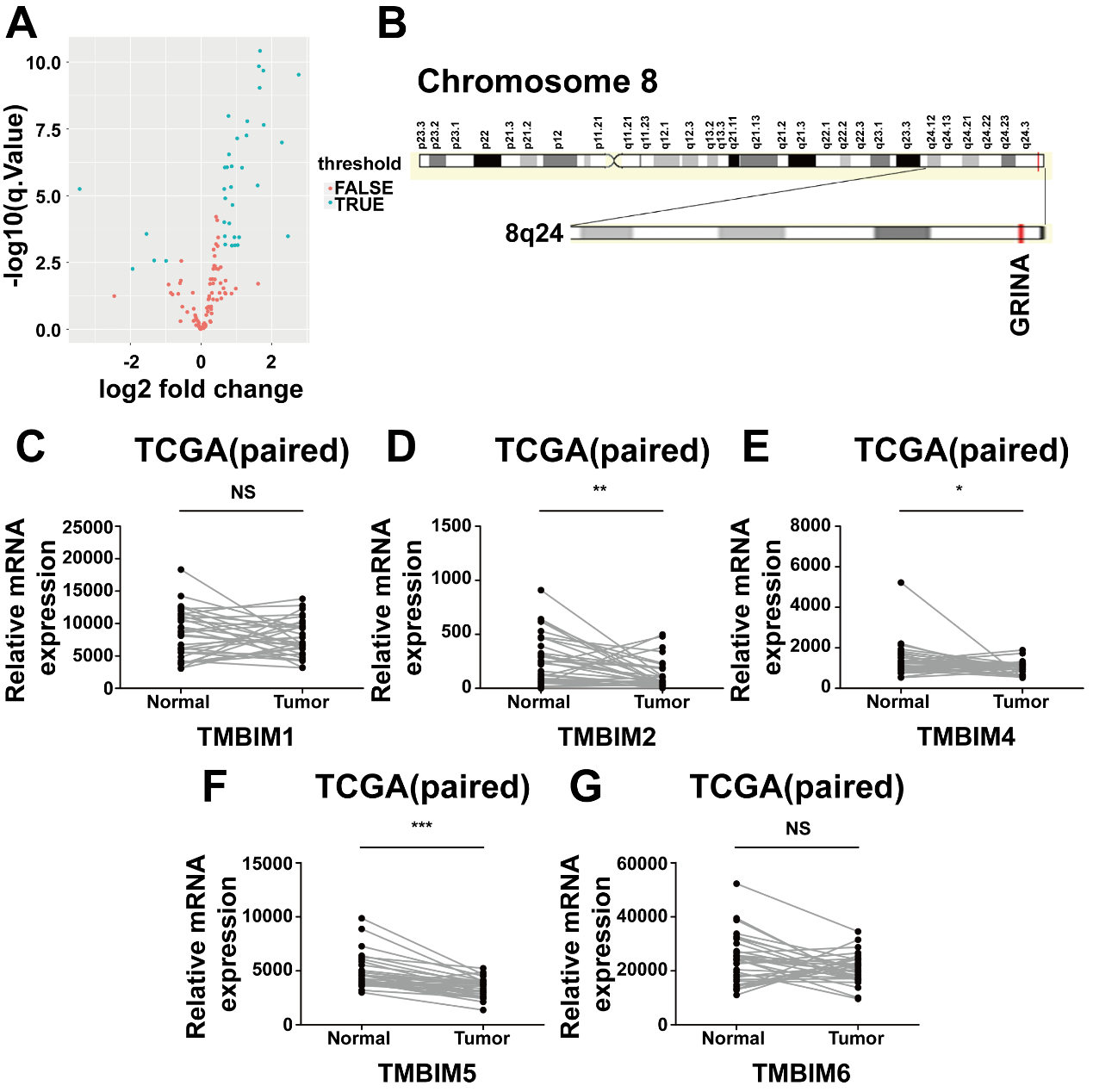

Supplement: Supplementary file 5 — Figure S1. Expression of GRINA rather than the other five members of the TMBIM family was increased in gastric cancer. (DOCX 19 kb) [file 13046_2018_974_MOESM5_ESM.docx]
