# Supplementary material for: Transmembrane protein GRINA modulates aerobic glycolysis and promotes tumor progression in gastric cancer
Source: J Exp Clin Cancer Res. 2018 Dec 12;37:308. doi: 10.1186/s13046-018-0974-1 (PMC6292005; doi:10.1186/s13046-018-0974-1)

**Figure S2.** KMplotter database demonstrated that patients with high GRINA expression had worse overall survival (OS) than those with low GRINA expression.


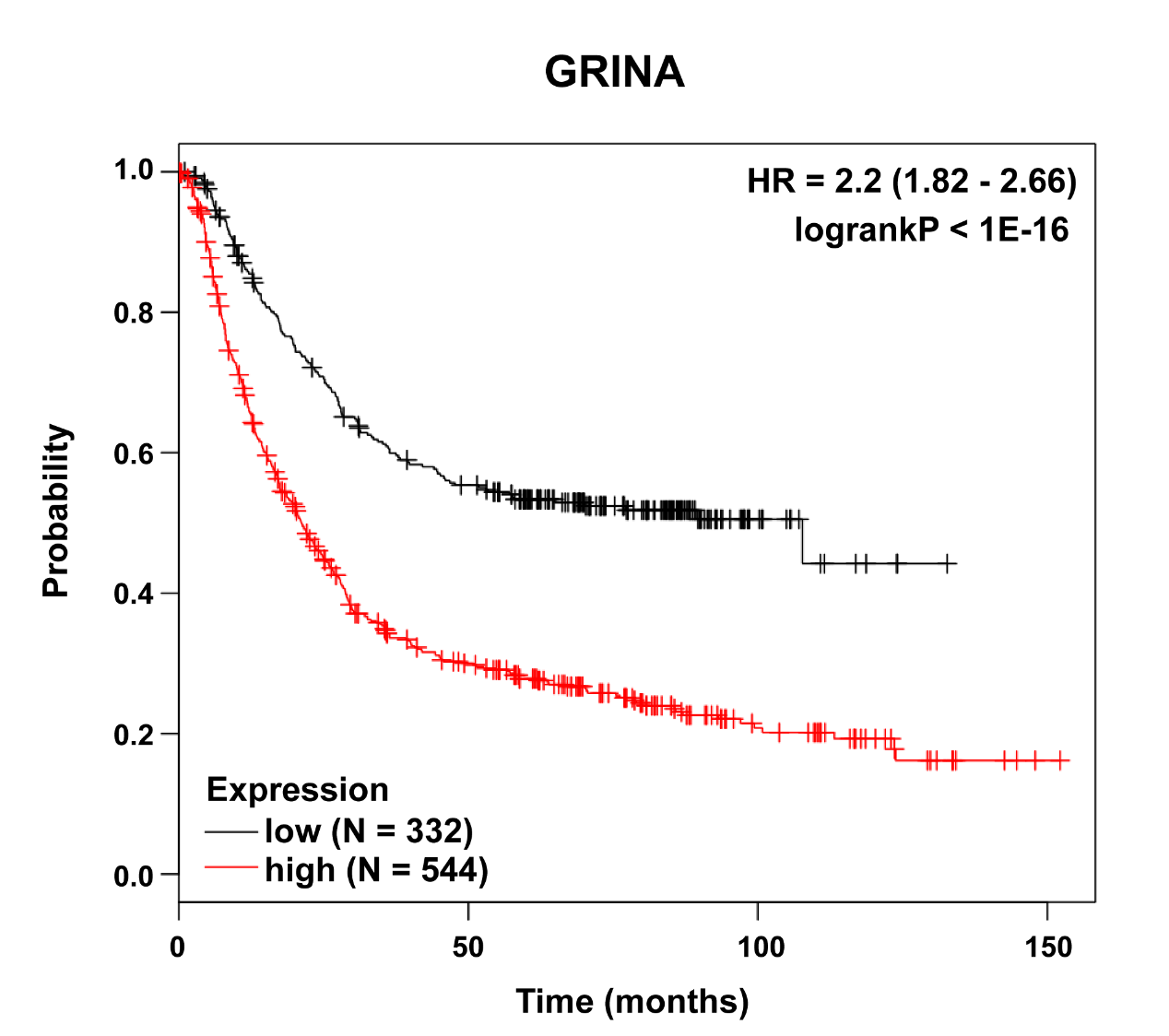

Supplement: Supplementary file 8 — Figure S2. KMplotter database demonstrated that patients with high GRINA expression had worse overall survival (OS) than those with low GRINA expression. (DOCX 19 kb) [file 13046_2018_974_MOESM8_ESM.docx]
