# Supplementary material for: Transmembrane protein GRINA modulates aerobic glycolysis and promotes tumor progression in gastric cancer
Source: J Exp Clin Cancer Res. 2018 Dec 12;37:308. doi: 10.1186/s13046-018-0974-1 (PMC6292005; doi:10.1186/s13046-018-0974-1)

**Figure S3.** Knockdown or overexpression of GRINA in gastric cancer cell lines. (A-D) Real-time PCR and western blot analysis showed shRNA targeting GRINA reduced the GRINA expression level in BGC-823 and AGS cell lines. (N=3). (E-H) Real-time PCR and western blot analysis showed plasmid containing GRINA-HA increased the GRINA expression level in HGC27 and N87 cell lines. (N=3).


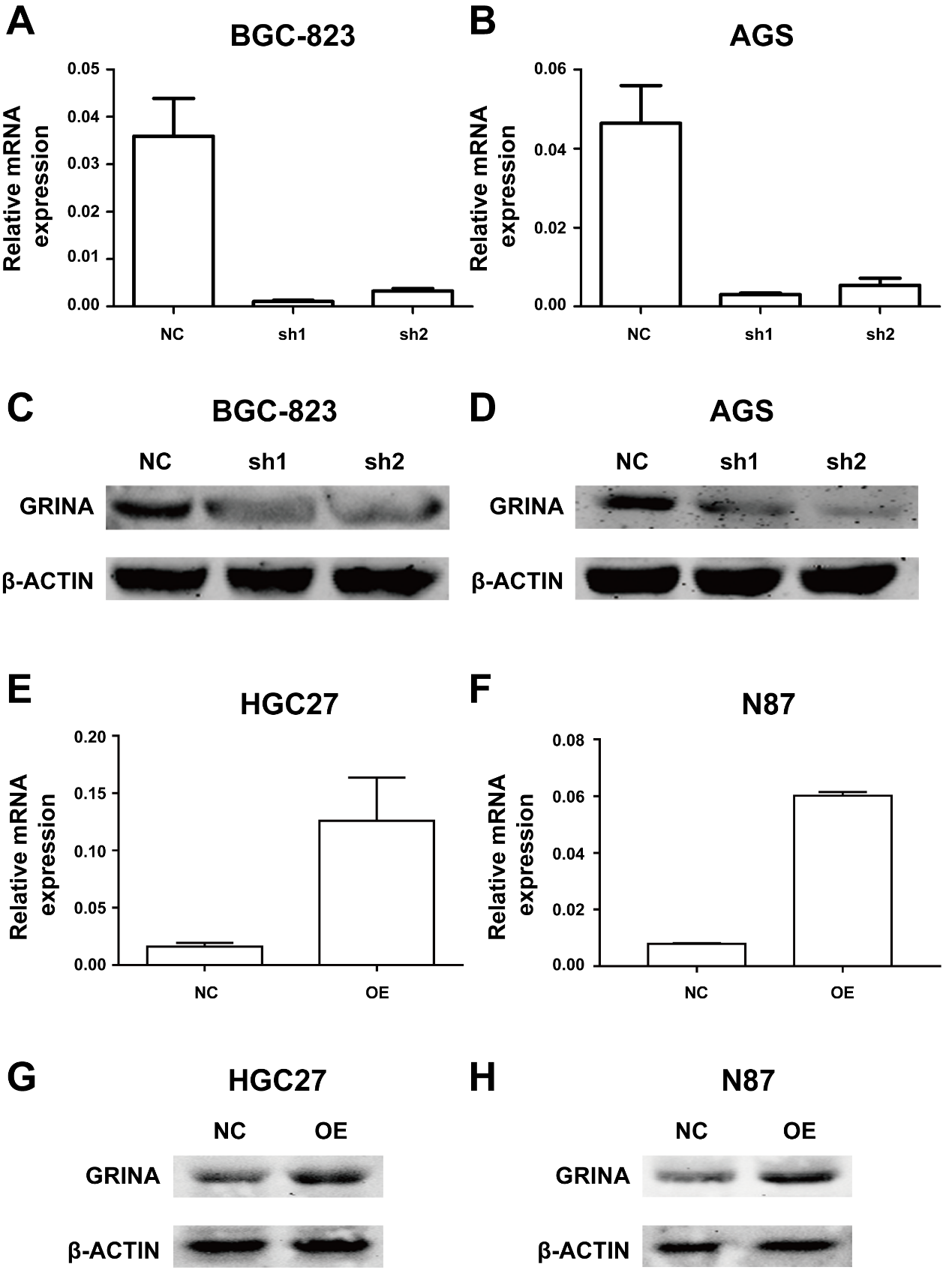

Supplement: Supplementary file 9 — Figure S3. Knockdown of GRINA in BGC-823 and AGS cell lines by short hairpin RNA (shRNNA). (XLS 293 kb) [file 13046_2018_974_MOESM9_ESM.xls]
