# Supplementary material for: Transmembrane protein GRINA modulates aerobic glycolysis and promotes tumor progression in gastric cancer
Source: J Exp Clin Cancer Res. 2018 Dec 12;37:308. doi: 10.1186/s13046-018-0974-1 (PMC6292005; doi:10.1186/s13046-018-0974-1)

**Figure S4.** Effects of GRINA knockdown or overexpression on phosphate pentose pathway, hexosamine biosynthesis pathway and glutamine metabolism. (A) GSEA plot based on the gene expression profiles of gastric cancer samples from TCGA database. (B-C) Effects of GRINA knockdown on phosphate pentose pathway in BGC-823 and AGS cells (N=3). (D) Effects of GRINA knockdown on hexosamine biosynthesis pathway in BGC-823 and AGS cells. (E-F) Effects of GRINA knockdown on glutamine metabolism in BGC-823 and AGS cells (N=3). (G-H) Effects of GRINA overexpression on phosphate pentose pathway in HGC27 and N87 cells (N=3). (I) Effects of GRINA overexpression on hexosamine biosynthesis pathway in HGC27 and N87 cells. (J-K) Effects of GRINA overexpression on glutamine metabolism in HGC27 and N87 cells (N=3). (L-M) Oxygen consumption ratio (OCR) upon knockdown of GRINA or not. O, oligomycin; F, FCCP [carbonyl cyanide 4-(trifluoromethoxy) phenylhydrazone]; A & R, antimycin A and rotenone (N=3). (N-O) OCR upon overexpression of GRINA or not. O, oligomycin; F, FCCP [carbonyl cyanide 4-(trifluoromethoxy) phenylhydrazone]; A & R, antimycin A and rotenone (N=3). (P) Total and phosphorylated AMPK were measured by western blotting analysis after GRINA was silenced (N=3). Values are mean ± s.d., *P < 0.05, **P < 0.01, ***P < 0.001, NS indicates no significance. (Student’s *t*-test).


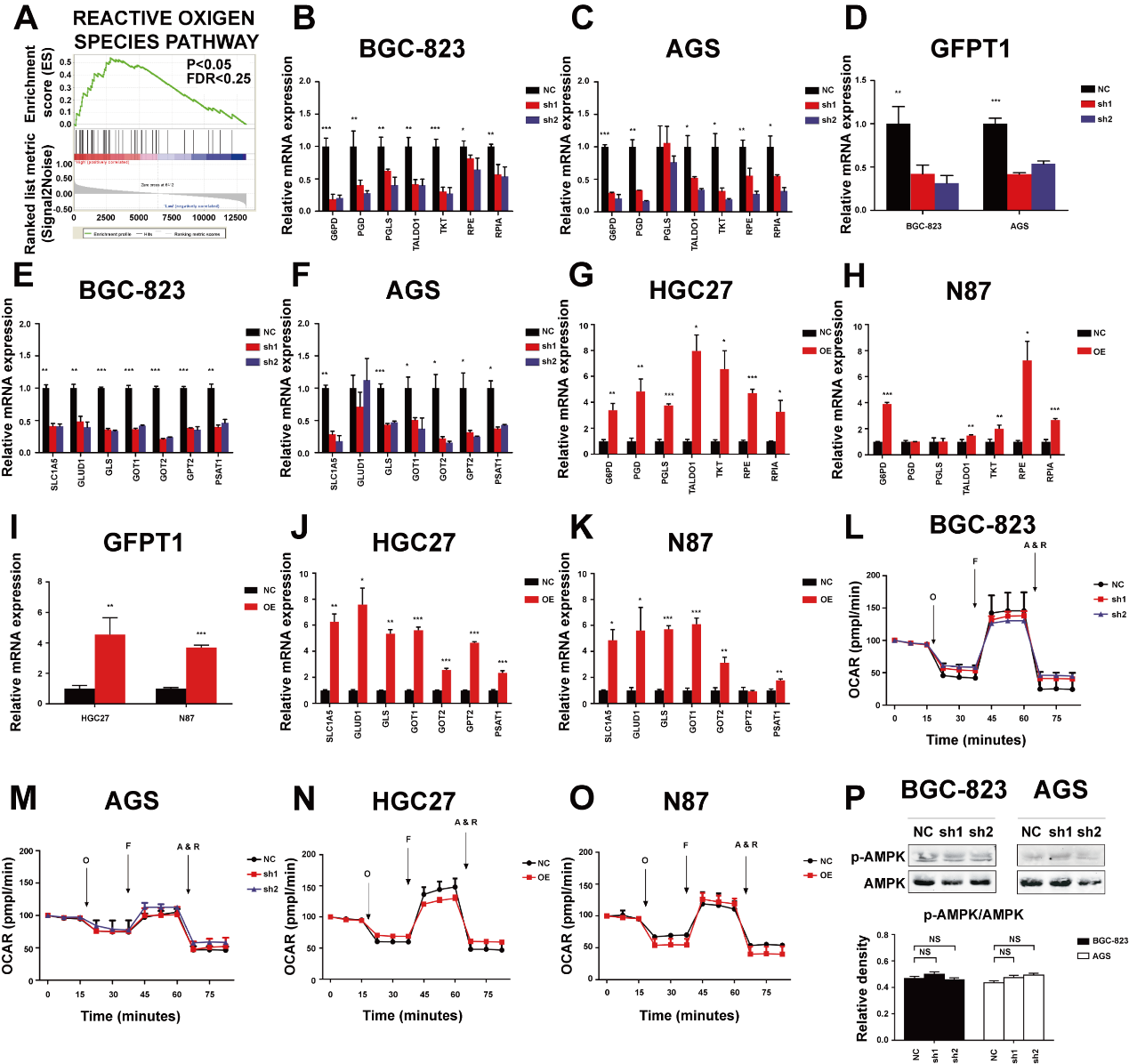

Supplement: Supplementary file 11 — Figure S4. Effects of GRINA knockdown on phosphate pentose pathway, hexosamine biosynthesis pathway and glutamine metabolism. (XLS 448 kb) [file 13046_2018_974_MOESM11_ESM.xls]
