# Supplementary material for: Transmembrane protein GRINA modulates aerobic glycolysis and promotes tumor progression in gastric cancer
Source: J Exp Clin Cancer Res. 2018 Dec 12;37:308. doi: 10.1186/s13046-018-0974-1 (PMC6292005; doi:10.1186/s13046-018-0974-1)
Supplement: Supplementary file 10 [file 13046_2018_974_MOESM6_ESM.docx]

## Sheet: 基因组所有基因

Table S5. Genes on chromosome 8
hg19_Chromosome	hg19_Start	hg19_Stop	TrainingDataset	HumanGeneID	MouseOrthologGeneID	Symbol	Synonyms	Description	EnsemblGeneID	ProteinLength	TargetP_Score	MitoDomain_Score	CoexpressionGnfN50_Score	PGC_Induction_Score	YeastMitoHomolog_Score	RickettsiaHomolog_Score	MSMS_Score	MCARTA2.0_score	MCARTA2_FDR	MCARTA2_LIST	MCARTA2_EVIDENCE	hg19_Chromosome	hg19_Start	hg19_Stop	MSMS_NUM_TISSUES	MSMS_NUM_PEPTIDES_UNIQUE	MSMS_NUM_SPECTRA	MSMS_TOTAL_INTENSITY	MSMS_PERCENT_COVERAGE	Tissues	cerebrum_total_peak_intensity_log10	cerebellum_total_peak_intensity_log10	brainstem_total_peak_intensity_log10	spinalcord_total_peak_intensity_log10	kidney_total_peak_intensity_log10	liver_total_peak_intensity_log10	heart_total_peak_intensity_log10	skeletalmuscle_total_peak_intensity_log10	adipose_total_peak_intensity_log10	smallintestine_total_peak_intensity_log10	largeintestine_total_peak_intensity_log10	stomach_total_peak_intensity_log10	placenta_total_peak_intensity_log10	testis_total_peak_intensity_log10	HPA_PrimarySubcellularLocalization_2015
chr8	116085	117024	Tnon_mito	441308		OR4F21	OR4F21P|O95013	olfactory receptor, family 4, subfamily F, member 21	ENSG00000176269	312		NonMitoDomain			NoMitoHomolog	NoHomolog		-13.4554	0.924	0		chr8	116085	117024
chr8	182136	197340	Tnon_mito	169270		ZNF596	Q8TC21	zinc finger protein 596	ENSG00000172748	504		NonMitoDomain			NoMitoHomolog	NoHomolog		-13.4554	0.924	0		chr8	182136	197340
chr8	356807	419875	Tnon_mito	26260	66822	FBXO25	FBX25|Q8TCJ0	F-box protein 25	ENSG00000147364	367		NA	2		NoMitoHomolog	NoHomolog		-8.0704	0.8449999999999999	0		chr8	356807	419875
chr8	439789	495781	Tnon_mito	157695	72148	TDRP	C8orf42|Inm01|TDRP1|TDRP2|Q86YL5	testis development related protein	ENSG00000180190	198		NA	0		NoMitoHomolog	NoHomolog	0-25ambig	-5.6553	0.751	0		chr8	439789	495781	1	2	3	1030000	13	testis														6
chr8	614199	681226	Tnon_mito	157697	234086	ERICH1	Q86X53	glutamate-rich 1	ENSG00000104714	443	4	NA	NA		NoMitoHomolog	NoHomolog		-5.6359	0.748	0		chr8	614199	681226																					Nucleoli (APE, Uncertain)
chr8	1449531	1656642	Tnon_mito	9228	244310	DLGAP2	DAP2|SAPAP2|Q9P1A6	discs, large (Drosophila) homolog-associated protein 2	ENSG00000198010	975		NonMitoDomain	0		NoMitoHomolog	NoHomolog		-10.6961	0.887	0		chr8	1449531	1656642
chr8	1711869	1734736	Tpossible_mito	2055	26889	CLN8	C8orf61|EPMR|Q9UBY8	ceroid-lipofuscinosis, neuronal 8 (epilepsy, progressive with mental retardation)	ENSG00000182372	286		NonMitoDomain	1		NoMitoHomolog	NoHomolog		-10.6961	0.887	0		chr8	1711869	1734736
chr8	1772148	1906807	Tnon_mito	9639	234094	ARHGEF10	GEF10|O15013	Rho guanine nucleotide exchange factor (GEF) 10	ENSG00000104728	1344		NonMitoDomain	0		NoMitoHomolog	NoHomolog		-10.6961	0.887	0		chr8	1772148	1906807																					Nucleus but not nucleoli (APE, Uncertain)
chr8	1922043	1955109	Tnon_mito	9920	74901	KBTBD11	KLHDC7C|O94819	kelch repeat and BTB (POZ) domain containing 11	ENSG00000176595	623		NonMitoDomain	1		NoMitoHomolog	NoHomolog		-10.6961	0.887	0		chr8	1922043	1955109
chr8	1993157	2093380	Tnon_mito	9172	17930	MYOM2	TTNAP|P54296	myomesin 2	ENSG00000036448	1465	4	NonMitoDomain	1		NoMitoHomolog	NoHomolog	25-50crude	-5.4956	0.7390000000000001	0		chr8	1993157	2093380	1	36	48	310000000	28	skeletalmuscle								8.5							Mitochondria (APE, Supportive)
chr8	2792874	4852328	Tnon_mito	64478	94109	CSMD1	PPP1R24|Q96PZ7	CUB and Sushi multiple domains 1	ENSG00000183117	3564		NonMitoDomain	0		NoMitoHomolog	NoHomolog		-10.6961	0.887	0		chr8	2792874	4852328
chr8	6264112	6501140	Tnon_mito	79648	244329	MCPH1	BRIT1|MCT|Q8NEM0	microcephalin 1	ENSG00000147316	835		SharedDomain	3		NoMitoHomolog	NoHomolog		-6.2297	0.767	0		chr8	6264112	6501140																					Nucleus but not nucleoli (APE, Uncertain)
chr8	6357174	6420784	Tnon_mito	285	11601	ANGPT2	AGPT2|ANG2|O15123	angiopoietin 2	ENSG00000091879	496		NonMitoDomain	1		NoMitoHomolog	NoHomolog		-10.6961	0.887	0		chr8	6357174	6420784
chr8	6565877	6619021	Tmito	55326	52123	AGPAT5	1AGPAT5|LPAATE|Q9NUQ2	1-acylglycerol-3-phosphate O-acyltransferase 5	ENSG00000155189	364		SharedDomain	3	0.73	NoMitoHomolog	Homolog	25-50ambig	5.9986	0.04	1	literature, Rickettsial homolog, mito protein domain, induction, MS/MS	chr8	6565877	6619021	8	16	65	562000000	47	cerebellum, brainstem, spinalcord, adipose, smallintestine, largeintestine, placenta, testis		6.6	8.3	7.7					6.7	7.6	8		8.2	7.2	Nucleus but not nucleoli (APE, Uncertain)
chr8	6666040	6693166	Tnon_mito	389610	319581	XKR5	HARL2754|UNQ2754|XRG5A|XRG5BM|Q6UX68	XK, Kell blood group complex subunit-related family, member 5		686		NonMitoDomain	0		NoMitoHomolog	NoHomolog		-10.6961	0.887	0		chr8	6666040	6693166
chr8	6728096	6735529	Tnon_mito	1672	13214	DEFB1	BD1|DEFB-1|DEFB101|HBD1|P60022	defensin, beta 1	ENSG00000164825	68		NonMitoDomain	2		NoMitoHomolog	NoHomolog		-10.6961	0.887	0		chr8	6728096	6735529
chr8	6782215	6783598	Tnon_mito	1671		DEFA6	DEF6|HD-6|Q01524	defensin, alpha 6, Paneth cell-specific	ENSG00000164822	100		NonMitoDomain			NoMitoHomolog	NoHomolog		-13.4554	0.924	0		chr8	6782215	6783598
chr8	6793344	6795786	Tnon_mito	1669		DEFA4	DEF4|HNP-4|HP-4|HP4|P12838	defensin, alpha 4, corticostatin	ENSG00000164821	97		NonMitoDomain			NoMitoHomolog	NoHomolog		-13.4554	0.924	0		chr8	6793344	6795786
chr8	6835170	6837614	Tnon_mito	1667		DEFA1	DEF1|DEFA2|HNP-1|HP-1|HP1|MRS	defensin, alpha 1	ENSG00000206047	94		NonMitoDomain			NoMitoHomolog	NoHomolog		-13.4554	0.924	0		chr8	6835170	6837614
chr8	6835170	6837614	Tnon_mito	728358		DEFA1B	HNP-1|HP-1|HP1	defensin, alpha 1B	ENSG00000206047	94		NonMitoDomain			NoMitoHomolog	NoHomolog		-13.4554	0.924	0		chr8	6835170	6837614
chr8	6873390	6875816	Tnon_mito	1668		DEFA3	DEF3|HNP-3|HNP3|HP-3|HP3|P59666	defensin, alpha 3, neutrophil-specific	ENSG00000239839	94		NonMitoDomain			NoMitoHomolog	NoHomolog		-13.4554	0.924	0		chr8	6873390	6875816
chr8	6912828	6914259	Tnon_mito	1670	23855	DEFA5	DEF5|HD-5|Q01523	defensin, alpha 5, Paneth cell-specific	ENSG00000164816	94		NonMitoDomain	NA		NoMitoHomolog	NoHomolog		-10.6961	0.887	0		chr8	6912828	6914259
chr8	7189908	7191501	Tnon_mito	401447		USP17L1P	USP17L1|Q7RTZ2	ubiquitin specific peptidase 17-like family member 1, pseudogene	ENSG00000223443	530		SharedDomain			NoMitoHomolog	NoHomolog		-8.989	0.879	0		chr8	7189908	7191501
chr8	7194636	7196229	Tnon_mito	645402		USP17L4	A6NCW7	ubiquitin specific peptidase 17-like family member 4	ENSG00000223443	530		SharedDomain			NoMitoHomolog	NoHomolog		-8.989	0.879	0		chr8	7194636	7196229
chr8	7215497	7220490	Tnon_mito	100131980		ZNF705G	A8MUZ8	zinc finger protein 705G	ENSG00000215372	300		NonMitoDomain			NoMitoHomolog	NoHomolog		-13.4554	0.924	0		chr8	7215497	7220490
chr8	7272384	7274354	Tnon_mito	100289462		DEFB4B	DEFB4P	defensin, beta 4B	ENSG00000171711	64		NonMitoDomain			NoMitoHomolog	NoHomolog		-13.4554	0.924	0		chr8	7272384	7274354
chr8	7286415	7287682	Tnon_mito	55894		DEFB103B	BD-3|DEFB103|DEFB3|HBD3|HBP3	defensin, beta 103B	ENSG00000176797	67		NonMitoDomain			NoMitoHomolog	NoHomolog		-13.4554	0.924	0		chr8	7286415	7287682
chr8	7286490	7287870	Tnon_mito	414325	244332	DEFB103A	BD-3|DEFB-3|DEFB103|DEFB3|HBD3|HBP-3|HBP3|hBD-3	defensin, beta 103A	ENSG00000176797	67		NonMitoDomain	NA		NoMitoHomolog	NoHomolog		-10.6961	0.887	0		chr8	7286490	7287870
chr8	7305275	7321192	Tnon_mito	10407	546038	SPAG11B	EDDM2B|EP2|EP2C|EP2D|HE2|HE2C|SPAG11|Q08648	sperm associated antigen 11B	ENSG00000164871	133		NonMitoDomain	NA		NoMitoHomolog	NoHomolog		-10.6961	0.887	0		chr8	7305275	7321192
chr8	7327829	7332604	Tnon_mito	140596		DEFB104A	BD-4|DEFB-4|DEFB104|DEFB4|hBD-4	defensin, beta 104A	ENSG00000176782	72		NonMitoDomain			NoMitoHomolog	NoHomolog		-13.4554	0.924	0		chr8	7327829	7332604
chr8	7327829	7332604	Tnon_mito	503618		DEFB104B	BD-4|DEFB-4|hBD-4	defensin, beta 104B	ENSG00000176782	72		NonMitoDomain			NoMitoHomolog	NoHomolog		-13.4554	0.924	0		chr8	7327829	7332604
chr8	7340025	7343909	Tnon_mito	503841	246082	DEFB106B	BD-6|DEFB-6	defensin, beta 106B	ENSG00000186579	65		NonMitoDomain	0		NoMitoHomolog	NoHomolog		-10.6961	0.887	0		chr8	7340025	7343909
chr8	7340025	7343909	Tnon_mito	245909		DEFB106A	BD-6|DEFB-6|DEFB106	defensin, beta 106A	ENSG00000186579	65		NonMitoDomain			NoMitoHomolog	NoHomolog		-13.4554	0.924	0		chr8	7340025	7343909
chr8	7345242	7347073	Tnon_mito	504180	77674	DEFB105B	BD-5|DEFB-5|B2RU30	defensin, beta 105B	ENSG00000186562	78		NonMitoDomain	NA		NoMitoHomolog	NoHomolog		-10.6961	0.887	0		chr8	7345242	7347073
chr8	7345242	7347073	Tnon_mito	245908		DEFB105A	BD-5|DEFB-5|DEFB105	defensin, beta 105A	ENSG00000186562	78		NonMitoDomain			NoMitoHomolog	NoHomolog		-13.4554	0.924	0		chr8	7345242	7347073
chr8	7353367	7366833	Tnon_mito	245910	246083	DEFB107A	BD-7|DEFB-7|DEFB107	defensin, beta 107A	ENSG00000186572	70		NonMitoDomain	0		NoMitoHomolog	NoHomolog		-10.6961	0.887	0		chr8	7353367	7366833
chr8	7353367	7366833	Tnon_mito	503614		DEFB107B	HsT21816	defensin, beta 107B	ENSG00000186572	70		NonMitoDomain			NoMitoHomolog	NoHomolog		-13.4554	0.924	0		chr8	7353367	7366833
chr8	7397148	7399972	Tnon_mito	100131608		PRR23D1	-	proline rich 23 domain containing 1	ENSG00000255251	279		NA			NoMitoHomolog	NoHomolog		-10.8297	0.921	0		chr8	7397148	7399972
chr8	7397149	7399972	Tnon_mito	100133251		PRR23D2	-	proline rich 23 domain containing 2	ENSG00000255251	279		NA			NoMitoHomolog	NoHomolog		-10.8297	0.921	0		chr8	7397149	7399972
chr8	7705401	7721319	Tnon_mito	653423		SPAG11A	EDDM2A|HE2|A0A0A0MR37	sperm associated antigen 11A	ENSG00000164871	108		NonMitoDomain			NoMitoHomolog	NoHomolog		-13.4554	0.924	0		chr8	7705401	7721319
chr8	7752198	7754237	Tnon_mito	1673		DEFB4A	BD-2|DEFB-2|DEFB102|DEFB2|DEFB4|HBD-2|SAP1	defensin, beta 4A	ENSG00000171711	64		NonMitoDomain			NoMitoHomolog	NoHomolog		-13.4554	0.924	0		chr8	7752198	7754237
chr8	7783858	7809935	Tnon_mito	100132396		ZNF705B	P0CI00	zinc finger protein 705B	ENSG00000215356	300		NonMitoDomain			NoMitoHomolog	NoHomolog		-13.4554	0.924	0		chr8	7783858	7809935
chr8	7829182	7830775	Tnon_mito	392188		USP17L8	USP17L10|P0C7I0	ubiquitin specific peptidase 17-like family member 8	ENSG00000223443	530		SharedDomain			NoMitoHomolog	NoHomolog		-8.989	0.879	0		chr8	7829182	7830775
chr8	7833914	7835507	Tnon_mito	645836		USP17L3	USP17B|USP17F|A6NCW0	ubiquitin specific peptidase 17-like family member 3	ENSG00000223443	530		SharedDomain			NoMitoHomolog	NoHomolog		-8.989	0.879	0		chr8	7833914	7835507
chr8	8175257	8239257	Tnon_mito	157285	244418	SGK223	PRAGMIN|Q86YV5	homolog of rat pragma of Rnd2	ENSG00000182319	1402		SharedDomain	0		NoMitoHomolog	NoHomolog		-6.2297	0.767	0		chr8	8175257	8239257																					Nucleus;Centrosome (APE, Uncertain)
chr8	8559665	8561617	Tnon_mito	137075	71908	CLDN23	CLDNL|hCG1646163|Q96B33	claudin 23	ENSG00000253958	292		NonMitoDomain	2		NoMitoHomolog	NoHomolog		-10.6961	0.887	0		chr8	8559665	8561617
chr8	8641998	8751131	Tnon_mito	9258	52065	MFHAS1	LRRC65|MASL1|Q9Y4C4	malignant fibrous histiocytoma amplified sequence 1	ENSG00000147324	1052	3	SharedDomain	0		HomologMitoLowConf	NoHomolog	0-25ambig	1.4271	0.224	0		chr8	8641998	8751131	1	2	2	51000000	3	testis														7.7	Nucleus but not nucleoli (APE, Supportive)
chr8	8860313	8890849	Tnon_mito	90459	67276	ERI1	3'HEXO|HEXO|THEX1|Q8IV48	exoribonuclease 1	ENSG00000104626	349		SharedDomain	2		NoMitoHomolog	NoHomolog		-6.2297	0.767	0		chr8	8860313	8890849
chr8	8993763	9009152	Tnon_mito	79660	244416	PPP1R3B	GL|PPP1R4|PTG|Q86XI6	protein phosphatase 1, regulatory subunit 3B	ENSG00000173281	285		NA	2		NoMitoHomolog	NoHomolog		-8.0704	0.8449999999999999	0		chr8	8993763	9009152																					Cytoplasm (APE, Uncertain)
chr8	9413444	9639856	Tnon_mito	8658	21951	TNKS	ARTD5|PARP-5a|PARP5A|PARPL|TIN1|TINF1|TNKS1|pART5|O95271	tankyrase, TRF1-interacting ankyrin-related ADP-ribose polymerase	ENSG00000173273	1327	2	SharedDomain	2		NoMitoHomolog	Homolog		2.0214	0.182	0		chr8	9413444	9639856																					Nucleus but not nucleoli (APE, Supportive)
chr8	9911829	10286401	Tpossible_mito	4482	110265	MSRA	PMSR|Q9UJ68	methionine sulfoxide reductase A	ENSG00000175806	235	5	SharedDomain	11		NoMitoHomolog	NoHomolog	50-75pure	10.9177	0.009000000000000001	1	mito protein domain, coexpression, MS/MS++	chr8	9911829	10286401	13	15	166	8650000000	66	cerebrum, cerebellum, brainstem, spinalcord, kidney, liver, heart, skeletalmuscle, adipose, smallintestine, largeintestine, stomach, placenta	8.2	8.1	8.6	8	9.6	9.4	8.2	6.7	8.4	8.8	8.3	8.7	8.2		Nucleus but not nucleoli (APE, Supportive)
chr8	10383055	10411676	Tnon_mito	203074	71037	PRSS55	CT153|T-SP1|TSP1|UNQ9391|Q6UWB4	protease, serine, 55	ENSG00000184647	352		SharedDomain	0		NoMitoHomolog	NoHomolog		-6.2297	0.767	0		chr8	10383055	10411676
chr8	10463859	10512617	Tnon_mito	94137	271209	RP1L1	DCDC4B|A6NKC6	retinitis pigmentosa 1-like 1	ENSG00000183638	2400	3	NonMitoDomain	NA		NoMitoHomolog	NoHomolog		-7.3264	0.8309999999999998	0		chr8	10463859	10512617
chr8	10530146	10558103	Tnon_mito	203076	67750	C8orf74	Q6P047	chromosome 8 open reading frame 74	ENSG00000171060	294	5	NA	2		NoMitoHomolog	NoHomolog		-6.0415	0.762	0		chr8	10530146	10558103																					Nucleus but not nucleoli (APE, Uncertain)
chr8	10581277	10588084	Tnon_mito	83595	20680	SOX7	Q9BT81	SRY (sex determining region Y)-box 7	ENSG00000171056	388		SharedDomain	0		NoMitoHomolog	NoHomolog		-6.2297	0.767	0		chr8	10581277	10588084
chr8	10622470	10697409	Tpossible_mito	54984	72400	PINX1	LPTL|LPTS|Q96BK5	PIN2/TERF1 interacting, telomerase inhibitor 1	ENSG00000254093	328		NonMitoDomain	4		NoMitoHomolog	NoHomolog		-10.6961	0.887	0		chr8	10622470	10697409																					Nucleus;Nucleoli (APE, Supportive)
chr8	10753656	11058875	Tnon_mito	286046	219149	XKR6	C8orf21|C8orf5|C8orf7|XRG6|Q5GH73	XK, Kell blood group complex subunit-related family, member 6	ENSG00000171044	641		NonMitoDomain	0		NoMitoHomolog	NoHomolog	0-25ambig	-8.2811	0.8690000000000001	0		chr8	10753656	11058875	1	5	5	55900000	8	brainstem			7.7
chr8	11141999	11185654	Tnon_mito	66036	210376	MTMR9	C8orf9|LIP-STYX|MTMR8|Q96QG7	myotubularin related protein 9	ENSG00000104643	549		NonMitoDomain	0		NoMitoHomolog	NoHomolog		-10.6961	0.887	0		chr8	11141999	11185654
chr8	11188494	11189695	Tnon_mito	83650		SLC35G5	AMAC|AMAC1L2|Q96KT7	solute carrier family 35, member G5	ENSG00000177710	338		SharedDomain			NoMitoHomolog	NoHomolog		-8.989	0.879	0		chr8	11188494	11189695
chr8	11278972	11324276	Tnon_mito	83648	219148	FAM167A	C8orf13|D8S265|Q96KS9	family with sequence similarity 167, member A	ENSG00000154319	214		NA	0		NoMitoHomolog	NoHomolog		-8.0704	0.8449999999999999	0		chr8	11278972	11324276
chr8	11351520	11422108	Tnon_mito	640	12143	BLK	MODY11|P51451	B lymphoid tyrosine kinase	ENSG00000136573	505		SharedDomain	1		HomologMitoLowConf	NoHomolog		-4.3576	0.6930000000000001	0		chr8	11351520	11422108
chr8	11561716	11617509	Tnon_mito	2626	14463	GATA4	ASD2|TACHD|VSD1|P43694	GATA binding protein 4	ENSG00000136574	442		NonMitoDomain	0		NoMitoHomolog	NoHomolog		-10.6961	0.887	0		chr8	11561716	11617509
chr8	11627171	11644854	Tnon_mito	252969	382913	NEIL2	NEH2|NEI2|Q969S2	nei endonuclease VIII-like 2 (E. coli)	ENSG00000154328	332	5	NonMitoDomain	NA		NoMitoHomolog	NoHomolog		-8.6672	0.875	0		chr8	11627171	11644854
chr8	11653081	11696818	Tnon_mito	2222	14137	FDFT1	DGPT|ERG9|SQS|SS|P37268	farnesyl-diphosphate farnesyltransferase 1	ENSG00000079459	476	3	SharedDomain	2		HomologMitoHighConf	NoHomolog		1.2281	0.232	0		chr8	11653081	11696818																					Endoplasmic reticulum (APE, Supportive)
chr8	11700033	11725646	Tpossible_mito	1508	13030	CTSB	APPS|CPSB|P07858	cathepsin B	ENSG00000164733	339		NonMitoDomain	1		NoMitoHomolog	NoHomolog	50-75pure	0.0417	0.323	0		chr8	11700033	11725646	13	21	973	73200000000	70	cerebrum, cerebellum, brainstem, spinalcord, kidney, liver, skeletalmuscle, adipose, smallintestine, largeintestine, stomach, placenta, testis	9.6	9.2	9	8.5	8.5	9.4		7.7	9	7.8	8.5	8.6	10.8	8.8	Vesicles (APE, Supportive)
chr8	11831445	11832108	Tnon_mito	613210	619548	DEFB136	DEFB137|Q30KP8	defensin, beta 136	ENSG00000205884	78		NA	0		NoMitoHomolog	NoHomolog		-8.0704	0.8449999999999999	0		chr8	11831445	11832108
chr8	11839829	11842099	Tnon_mito	613209	73670	DEFB135	DEFB136|Q30KP9	defensin, beta 135	ENSG00000205883	77		NonMitoDomain	NA		NoMitoHomolog	NoHomolog		-10.6961	0.887	0		chr8	11839829	11842099
chr8	11851488	11853760	Tnon_mito	613211		DEFB134	Q4QY38	defensin, beta 134	ENSG00000205882	66		NonMitoDomain			NoMitoHomolog	NoHomolog		-13.4554	0.924	0		chr8	11851488	11853760
chr8	11921897	11929256	Tnon_mito	245940	654465	DEFB130	DEFB-30|DEFB130L	defensin, beta 130	ENSG00000232948	79		NonMitoDomain	NA		NoMitoHomolog	NoHomolog		-10.6961	0.887	0		chr8	11921897	11929256
chr8	11921897	11929256	Tnon_mito	100133267		LOC100133267	-	defensin, beta 130-like	ENSG00000232948	79		NonMitoDomain			NoMitoHomolog	NoHomolog		-13.4554	0.924	0		chr8	11921897	11929256
chr8	11946846	11973025	Tnon_mito	728957		ZNF705D	ZNF705C|P0CH99	zinc finger protein 705D	ENSG00000215343	300		NonMitoDomain			NoMitoHomolog	NoHomolog		-13.4554	0.924	0		chr8	11946846	11973025
chr8	11989925	11991518	Tnon_mito	392197		USP17L7	P0C7H9	ubiquitin specific peptidase 17-like family member 7	ENSG00000223443	530		SharedDomain			NoMitoHomolog	NoHomolog		-8.989	0.879	0		chr8	11989925	11991518
chr8	11994676	11996269	Tnon_mito	377630	13532	USP17L2	DUB-3|DUB3|USP17|Q6R6M4	ubiquitin specific peptidase 17-like family member 2	ENSG00000223443	530		SharedDomain	NA		NoMitoHomolog	NoHomolog		-6.2297	0.767	0		chr8	11994676	11996269
chr8	12039612	12051624	Tnon_mito	85002		FAM86B1	Q8N7N1	family with sequence similarity 86, member B1	ENSG00000186523	296		NonMitoDomain			NoMitoHomolog	NoHomolog		-13.4554	0.924	0		chr8	12039612	12051624
chr8	12283123	12293852	Tnon_mito	653333		FAM86B2	P0C5J1	family with sequence similarity 86, member B2	ENSG00000118894	330		NonMitoDomain			NoMitoHomolog	NoHomolog		-13.4554	0.924	0		chr8	12283123	12293852
chr8	12579405	12612992	Tnon_mito	91694	244421	LONRF1	RNF191|Q17RB8	LON peptidase N-terminal domain and ring finger 1	ENSG00000154359	773	4	SharedDomain	0		NoMitoHomolog	NoHomolog		-3.7952	0.639	0		chr8	12579405	12612992																					Plasma membrane;Cytoplasm (APE, Uncertain)
chr8	12803182	12887284	Tnon_mito	57604	319582	KIAA1456	C8orf79|TRM9L|Q9P272	KIAA1456	ENSG00000250305	454		MitoDomain	0		NoMitoHomolog	NoHomolog		-1.0247	0.433	0		chr8	12803182	12887284
chr8	12940871	13372429	Tnon_mito	10395	50768	DLC1	ARHGAP7|HP|STARD12|p122-RhoGAP|Q96QB1	deleted in liver cancer 1	ENSG00000164741	1528		SharedDomain	0		NoMitoHomolog	NoHomolog		-6.2297	0.767	0		chr8	12940871	13372429																					Nucleus but not nucleoli;Golgi apparatus;Vesicles (APE, Uncertain)
chr8	13424351	13425797	Tnon_mito	157773	621080	C8orf48	Q96LL4	chromosome 8 open reading frame 48	ENSG00000164743	319		NA	0		NoMitoHomolog	NoHomolog		-8.0704	0.8449999999999999	0		chr8	13424351	13425797																					Cytoplasm (APE, Uncertain)
chr8	13947372	15095792	Tnon_mito	137868	244431	SGCZ	ZSG1|Q96LD1	sarcoglycan, zeta	ENSG00000185053	312		NonMitoDomain	NA		NoMitoHomolog	NoHomolog		-10.6961	0.887	0		chr8	13947372	15095792
chr8	15397595	15624158	Tpossible_mito	7991	80286	TUSC3	D8S1992|M33|MRT22|MRT7|N33|OST3A|Q13454	tumor suppressor candidate 3	ENSG00000104723	348		SharedDomain	2		NoMitoHomolog	NoHomolog		-6.2297	0.767	0		chr8	15397595	15624158
chr8	15965386	16050300	Tnon_mito	4481	20288	MSR1	CD204|SCARA1|SR-A|SRA|phSR1|phSR2|P21757	macrophage scavenger receptor 1	ENSG00000038945	451		NonMitoDomain	1		NoMitoHomolog	NoHomolog		-10.6961	0.887	0		chr8	15965386	16050300
chr8	16850333	16859674	Tnon_mito	26281	80857	FGF20	FGF-20|Q9NP95	fibroblast growth factor 20	ENSG00000078579	211		NonMitoDomain	NA		NoMitoHomolog	NoHomolog		-10.6961	0.887	0		chr8	16850333	16859674
chr8	16884746	16980148	Tpossible_mito	286097	78506	MICU3	EFHA2|Q86XE3	mitochondrial calcium uptake family, member 3	ENSG00000155970	530	2	SharedDomain	0		NoMitoHomolog	NoHomolog	25-50ambig	4.4332	0.077	0		chr8	16884746	16980148	6	24	157	2380000000	48	cerebrum, cerebellum, brainstem, spinalcord, skeletalmuscle, stomach	8.8	9	8.7	7.7				8.2				6.6
chr8	17013835	17080241	Tnon_mito	51201	70546	ZDHHC2	DHHC2|ZNF372|Q9UIJ5	zinc finger, DHHC-type containing 2	ENSG00000104219	367		NonMitoDomain	0		NoMitoHomolog	NoHomolog		-10.6961	0.887	0		chr8	17013835	17080241
chr8	17086739	17104387	Tnon_mito	29883	18983	CNOT7	CAF1|Caf1a|hCAF-1|Q9UIV1	CCR4-NOT transcription complex, subunit 7	ENSG00000198791	285		NonMitoDomain	4		NoMitoHomolog	NoHomolog		-10.6961	0.887	0		chr8	17086739	17104387																					Cytoplasm (APE, Uncertain)
chr8	17104400	17155533	Tnon_mito	137492	52348	VPS37A	HCRP1|PQBP2|SPG53|Q8NEZ2	vacuolar protein sorting 37 homolog A (S. cerevisiae)	ENSG00000155975	397	3	NonMitoDomain	2		NoMitoHomolog	NoHomolog		-7.3264	0.8309999999999998	0		chr8	17104400	17155533																					Vesicles (APE, Uncertain)
chr8	17154305	17271040	Tnon_mito	9108	54384	MTMR7	Q9Y216	myotubularin related protein 7	ENSG00000003987	660		NonMitoDomain	1		NoMitoHomolog	NoHomolog		-10.6961	0.887	0		chr8	17154305	17271040
chr8	17354596	17428077	Tnon_mito	6542	11988	SLC7A2	ATRC2|CAT2|HCAT2|P52569	solute carrier family 7 (cationic amino acid transporter, y+ system), member 2	ENSG00000003989	698	3	SharedDomain	2		NoMitoHomolog	Homolog		0.1441	0.309	0		chr8	17354596	17428077
chr8	17433941	17500642	Tnon_mito	5157	68797	PDGFRL	PDGRL|PRLTS|Q15198	platelet-derived growth factor receptor-like	ENSG00000104213	375		NonMitoDomain	0		NoMitoHomolog	NoHomolog		-10.6961	0.887	0		chr8	17433941	17500642																					Nucleoli;Golgi apparatus (APE, Uncertain)
chr8	17501302	17658426	Tpossible_mito	57509	102103	MTUS1	ATBP|ATIP|ICIS|MP44|MTSG1|Q9ULD2	microtubule associated tumor suppressor 1	ENSG00000129422	1270	2	NA	25		NoMitoHomolog	NoHomolog		2.8083	0.14500000000000002	0		chr8	17501302	17658426
chr8	17721899	17753047	Tnon_mito	2267	234199	FGL1	HFREP1|HP-041|LFIRE-1|LFIRE1|Q08830	fibrinogen-like 1	ENSG00000104760	312		NonMitoDomain	2		NoMitoHomolog	NoHomolog		-10.6961	0.887	0		chr8	17721899	17753047																					Mitochondria (APE, Supportive)
chr8	17780365	17887457	Tnon_mito	5108	18536	PCM1	PTC4|Q15154	pericentriolar material 1	ENSG00000078674	2024		NA	2		NoMitoHomolog	NoHomolog	0-25ambig	-5.6553	0.751	0		chr8	17780365	17887457	4	8	11	107000000	5	brainstem, spinalcord, placenta, testis			7.7	7.2									7.5	6.7	Centrosome (APE, Supportive)
chr8	17913924	17942507	Tnon_mito	427	11886	ASAH1	AC|ACDase|ASAH|PHP|PHP32|SMAPME|Q13510	N-acylsphingosine amidohydrolase (acid ceramidase) 1	ENSG00000104763	411		NA	0		NoMitoHomolog	NoHomolog	50-75pure	2.6674	0.15	0		chr8	17913924	17942507	12	30	515	25000000000	65	cerebrum, cerebellum, brainstem, spinalcord, kidney, liver, adipose, smallintestine, largeintestine, stomach, placenta, testis	9.6	9.4	9.5	9	8.6	8.8			8.8	9	8.8	9.1	9.8	9.5
chr8	18027970	18081198	Tnon_mito	9	17961	NAT1	AAC1|MNAT|NAT-1|NATI|P18440	N-acetyltransferase 1 (arylamine N-acetyltransferase)	ENSG00000171428	352		NonMitoDomain	4	-0.2800000000000001	NoMitoHomolog	NoHomolog		-10.0112	0.8830000000000001	0		chr8	18027970	18081198
chr8	18248754	18258723	Tnon_mito	10		NAT2	AAC2|NAT-2|PNAT|P11245	N-acetyltransferase 2 (arylamine N-acetyltransferase)	ENSG00000156006	290		NonMitoDomain			NoMitoHomolog	NoHomolog		-13.4554	0.924	0		chr8	18248754	18258723
chr8	18384812	18871196	Tnon_mito	23362	234353	PSD3	EFA6R|HCA67|Q9NYI0	pleckstrin and Sec7 domain containing 3	ENSG00000156011	1047		SharedDomain	0		NoMitoHomolog	Homolog		-3.2256	0.5780000000000001	0		chr8	18384812	18871196
chr8	19171080	19253729	Tnon_mito	63898	72281	SH2D4A	PPP1R38|SH2A|Q9H788	SH2 domain containing 4A	ENSG00000104611	454		SharedDomain	0		NoMitoHomolog	NoHomolog		-6.2297	0.767	0		chr8	19171080	19253729																					Cytoplasm (APE, Supportive)
chr8	19261671	19540094	Tnon_mito	55790	234356	CSGALNACT1	CSGalNAcT-1|ChGn|beta4GalNAcT|Q8TDX6	chondroitin sulfate N-acetylgalactosaminyltransferase 1	ENSG00000147408	532		NonMitoDomain	1		NoMitoHomolog	NoHomolog		-10.6961	0.887	0		chr8	19261671	19540094																					Cytoskeleton (Actin filaments) (APE, Supportive)
chr8	19674917	19709586	Tnon_mito	55174	70885	INTS10	C8orf35|INT10|Q9NVR2	integrator complex subunit 10	ENSG00000104613	710		NA	1		NoMitoHomolog	NoHomolog		-8.0704	0.8449999999999999	0		chr8	19674917	19709586																					Cytoplasm (APE, Uncertain)
chr8	19796581	19824770	Tnon_mito	4023	16956	LPL	HDLCQ11|LIPD|P06858	lipoprotein lipase	ENSG00000175445	475		NonMitoDomain	4		NoMitoHomolog	NoHomolog		-10.6961	0.887	0		chr8	19796581	19824770																					Vesicles (APE, Uncertain)
chr8	20002365	20040717	Tnon_mito	6570	110877	SLC18A1	CGAT|VAT1|VMAT1|P54219	solute carrier family 18 (vesicular monoamine transporter), member 1	ENSG00000036565	525		SharedDomain	2		NoMitoHomolog	Homolog		-3.2256	0.5780000000000001	0		chr8	20002365	20040717
chr8	20054703	20079207	Tnon_mito	526	11966	ATP6V1B2	ATP6B1B2|ATP6B2|HO57|VATB|VPP3|Vma2|P21281	ATPase, H+ transporting, lysosomal 56/58kDa, V1 subunit B2	ENSG00000147416	511		SharedDomain	0	-0.22	NoMitoHomolog	Homolog	50-75crude	0.7793000000000001	0.263	0		chr8	20054703	20079207	7	24	132	3130000000	57	cerebrum, cerebellum, brainstem, spinalcord, adipose, stomach, placenta	7.9	7.6	9.3	8.6					7.3			7	8.9		Vesicles (APE, Supportive)
chr8	20103675	20112803	Tnon_mito	11178	211134	LZTS1	F37|FEZ1|Q9Y250	leucine zipper, putative tumor suppressor 1	ENSG00000061337	596	4	NA	NA		NoMitoHomolog	NoHomolog		-5.6359	0.748	0		chr8	20103675	20112803																					Plasma membrane (APE, Uncertain)
chr8	21549529	21646346	Tnon_mito	2675	14586	GFRA2	GDNFRB|NRTNR-ALPHA|NTNRA|RETL2|TRNR2|O00451	GDNF family receptor alpha 2	ENSG00000168546	464		NonMitoDomain	0		NoMitoHomolog	NoHomolog		-10.6961	0.887	0		chr8	21549529	21646346																					Vesicles (APE, Uncertain)
chr8	21766383	21771205	Tnon_mito	9046	13449	DOK2	p56DOK|p56dok-2|O60496	docking protein 2, 56kDa	ENSG00000147443	412		NonMitoDomain	2		NoMitoHomolog	NoHomolog		-10.6961	0.887	0		chr8	21766383	21771205
chr8	21777179	21864096	Tnon_mito	23039	65246	XPO7	EXP7|RANBP16|Q9UIA9	exportin 7	ENSG00000130227	1087		NonMitoDomain	4	-0.26	NoMitoHomolog	NoHomolog	0-25ambig	-7.5961	0.8369999999999999	0		chr8	21777179	21864096	3	4	8	25400000	5	cerebrum, cerebellum, brainstem	6.8	7.1	6.8												Nucleus (APE, Uncertain)
chr8	21881620	21894408	Tnon_mito	10361	328440	NPM2	Q86SE8	nucleophosmin/nucleoplasmin 2	ENSG00000158806	214		NA	NA		NoMitoHomolog	NoHomolog		-8.0704	0.8449999999999999	0		chr8	21881620	21894408																					Nucleus;Nucleoli (APE, Uncertain)
chr8	21900427	21906319	Tnon_mito	8822	14171	FGF17	FGF-13|HH20|O60258	fibroblast growth factor 17	ENSG00000158815	216		NonMitoDomain	0		NoMitoHomolog	NoHomolog		-10.6961	0.887	0		chr8	21900427	21906319
chr8	21911080	21940036	Tnon_mito	2039	13829	DMTN	DMT|EPB49|Q08495	dematin actin binding protein	ENSG00000158856	405		NonMitoDomain	0		NoMitoHomolog	NoHomolog		-10.6961	0.887	0		chr8	21911080	21940036
chr8	21946713	21961891	Tnon_mito	64760	239170	FAM160B2	RAI16|Q86V87	family with sequence similarity 160, member B2	ENSG00000158863	743		NA	3	0.71	NoMitoHomolog	NoHomolog		-4.2621	0.69	0		chr8	21946713	21961891																					Nucleus but not nucleoli (APE, Uncertain)
chr8	21964382	21966932	Tnon_mito	79873	213484	NUDT18	MTH3|Q6ZVK8	nudix (nucleoside diphosphate linked moiety X)-type motif 18	ENSG00000173566	323		SharedDomain	1		NoMitoHomolog	NoHomolog		-6.2297	0.767	0		chr8	21964382	21966932																					Nucleus but not nucleoli (APE, Uncertain)
chr8	21971931	21988565	Tnon_mito	55806	15460	HR	ALUNC|AU|HSA277165|MUHH|MUHH1|O43593	hair growth associated	ENSG00000168453	1189		NonMitoDomain	0		NoMitoHomolog	NoHomolog	0-25ambig	-8.2811	0.8690000000000001	0		chr8	21971931	21988565	1	3	4	8190000	2	skeletalmuscle								6.9
chr8	21995532	21999448	Tnon_mito	80346	72549	REEP4	C8orf20|Q9H6H4	receptor accessory protein 4	ENSG00000168476	257		SharedDomain	2		NoMitoHomolog	NoHomolog		-6.2297	0.767	0		chr8	21995532	21999448																					Cytoplasm (APE, Uncertain)
chr8	22004342	22014344	Tnon_mito	203190	213469	LGI3	LGIL4|Q8N145	leucine-rich repeat LGI family, member 3	ENSG00000168481	548		SharedDomain	1		NoMitoHomolog	NoHomolog	0-25crude	-4.7411	0.7120000000000001	0		chr8	22004342	22014344	1	1	1	707000	3	spinalcord				5.8											Cytoplasm (APE, Uncertain)
chr8	22019183	22021991	Tnon_mito	6440	20389	SFTPC	BRICD6|PSP-C|SFTP2|SMDP2|SP-C|P11686	surfactant protein C	ENSG00000168484	197		NonMitoDomain	0		NoMitoHomolog	NoHomolog		-10.6961	0.887	0		chr8	22019183	22021991
chr8	22022652	22069840	Tnon_mito	649	12153	BMP1	OI13|PCOLC|PCP|PCP2|TLD|P13497	bone morphogenetic protein 1	ENSG00000168487	986		NonMitoDomain	2		NoMitoHomolog	NoHomolog		-10.6961	0.887	0		chr8	22022652	22069840
chr8	22077215	22089851	Tnon_mito	9796	105653	PHYHIP	DYRK1AP3|PAHX-AP|PAHXAP1|Q92561	phytanoyl-CoA 2-hydroxylase interacting protein	ENSG00000168490	330		NA	0		NoMitoHomolog	NoHomolog		-8.0704	0.8449999999999999	0		chr8	22077215	22089851
chr8	22102618	22108680	Tnon_mito	661	67065	POLR3D	BN51T|RPC4|RPC53|TSBN51|P05423	polymerase (RNA) III (DNA directed) polypeptide D, 44kDa	ENSG00000168495	398		NonMitoDomain	3		NoMitoHomolog	NoHomolog	0-25ambig	-8.2811	0.8690000000000001	0		chr8	22102618	22108680	3	3	6	152000000	9	liver, largeintestine, stomach						7.9					7.8	6.9			Nucleus but not nucleoli (APE, Supportive)
chr8	22132809	22213584	Tnon_mito	55124	57746	PIWIL2	CT80|HILI|PIWIL1L|mili|Q8TC59	piwi-like RNA-mediated gene silencing 2	ENSG00000197181	973	4	NonMitoDomain	1		NoMitoHomolog	NoHomolog	25-50crude	-5.4956	0.7390000000000001	0		chr8	22132809	22213584	2	22	29	513000000	30	stomach, testis												6.9		8.7
chr8	22224761	22291640	Tnon_mito	23516	213053	SLC39A14	LZT-Hs4|NET34|ZIP14|cig19|Q15043	solute carrier family 39 (zinc transporter), member 14	ENSG00000104635	492		NonMitoDomain	3		NoMitoHomolog	NoHomolog		-10.6961	0.887	0		chr8	22224761	22291640																					Plasma membrane;Endoplasmic reticulum;Golgi apparatus (APE, Supportive)
chr8	22298482	22398657	Tnon_mito	5533	19057	PPP3CC	CALNA3|CNA3|PP2Bgamma|P48454	protein phosphatase 3, catalytic subunit, gamma isozyme	ENSG00000120910	521	5	NonMitoDomain	0	1.38	NoMitoHomolog	NoHomolog	0-25crude	-3.3703	0.626	0		chr8	22298482	22398657	4	6	10	122000000	15	cerebrum, cerebellum, brainstem, spinalcord	7	7.8	7.6	6.4											Vesicles (APE, Uncertain)
chr8	22409250	22433008	Tnon_mito	10174	20410	SORBS3	SCAM-1|SCAM1|SH3D4|O60504	sorbin and SH3 domain containing 3	ENSG00000120896	671	5	SharedDomain	0		HomologMitoLowConf	NoHomolog		-2.3287	0.511	0		chr8	22409250	22433008																					Nucleus but not nucleoli;Focal Adhesions (APE, Uncertain)
chr8	22436253	22455538	Tnon_mito	64236	213019	PDLIM2	MYSTIQUE|SLIM|Q96JY6	PDZ and LIM domain 2 (mystique)	ENSG00000120913	602	4	SharedDomain	1	-0.25	NoMitoHomolog	NoHomolog		-3.1103	0.5700000000000001	0		chr8	22436253	22455538																					Cytoskeleton (Actin filaments);Focal Adhesions (APE, Supportive)
chr8	22457113	22461662	Tnon_mito	541565	268759	C8orf58	Q8NAV2	chromosome 8 open reading frame 58	ENSG00000241852	365		NA	0		NoMitoHomolog	NoHomolog		-8.0704	0.8449999999999999	0		chr8	22457113	22461662																					Nucleus (APE, Supportive)
chr8	22462538	22477983	Tpossible_mito	57805	219158	CCAR2	DBC-1|DBC1|KIAA1967|NET35|p30 DBC|p30DBC|Q8N163	cell cycle and apoptosis regulator 2	ENSG00000158941	923	3	NonMitoDomain	0		NoMitoHomolog	NoHomolog		-7.3264	0.8309999999999998	0		chr8	22462538	22477983																					Nucleus but not nucleoli (APE, Supportive)
chr8	22477946	22526661	Tnon_mito	55909	57784	BIN3	Q9NQY0	bridging integrator 3	ENSG00000147439	253		NonMitoDomain	0		NoMitoHomolog	NoHomolog		-10.6961	0.887	0		chr8	22477946	22526661
chr8	22545173	22550815	Tnon_mito	1960	13655	EGR3	EGR-3|PILOT|Q06889	early growth response 3	ENSG00000179388	387		NonMitoDomain	NA		NoMitoHomolog	NoHomolog		-10.6961	0.887	0		chr8	22545173	22550815
chr8	22570764	22785421	Tnon_mito	157310	73523	PEBP4	CORK-1|CORK1|GWTM1933|HEL-S-300|PEBP-4|PRO4408|hPEBP4|Q96S96	phosphatidylethanolamine-binding protein 4	ENSG00000134020	227		SharedDomain	2		NoMitoHomolog	NoHomolog		-6.2297	0.767	0		chr8	22570764	22785421
chr8	22844929	22877710	Tnon_mito	23221	246710	RHOBTB2	DBC2|Q9BYZ6	Rho-related BTB domain containing 2	ENSG00000008853	749		SharedDomain	1		NoMitoHomolog	NoHomolog		-6.2297	0.767	0		chr8	22844929	22877710
chr8	22877647	22926700	Tnon_mito	8795		TNFRSF10B	CD262|DR5|KILLER|KILLER/DR5|TRAIL-R2|TRAILR2|TRICK2|TRICK2A|TRICK2B|TRICKB|ZTNFR9|O14763	tumor necrosis factor receptor superfamily, member 10b	ENSG00000120889	440		NonMitoDomain			NoMitoHomolog	NoHomolog		-13.4554	0.924	0		chr8	22877647	22926700
chr8	22960326	22974950	Tnon_mito	8794		TNFRSF10C	CD263|DCR1|DCR1-TNFR|LIT|TRAIL-R3|TRAILR3|TRID|O14798	tumor necrosis factor receptor superfamily, member 10c, decoy without an intracellular domain	ENSG00000173535	259		NonMitoDomain			NoMitoHomolog	NoHomolog		-13.4554	0.924	0		chr8	22960326	22974950
chr8	22993100	23021543	Tnon_mito	8793		TNFRSF10D	CD264|DCR2|TRAIL-R4|TRAILR4|TRUNDD|Q9UBN6	tumor necrosis factor receptor superfamily, member 10d, decoy with truncated death domain	ENSG00000173530	386	2	NonMitoDomain			NoMitoHomolog	NoHomolog		-8.2084	0.8660000000000001	0		chr8	22993100	23021543
chr8	23048969	23082680	Tnon_mito	8797	21933	TNFRSF10A	APO2|CD261|DR4|TRAILR-1|TRAILR1|O00220	tumor necrosis factor receptor superfamily, member 10a	ENSG00000104689	468		NonMitoDomain	0		NoMitoHomolog	NoHomolog	0-25ambig	-8.2811	0.8690000000000001	0		chr8	23048969	23082680	1	2	5	161000000	8	smallintestine										8.2					Nucleus (APE, Uncertain)
chr8	23101149	23119512	Tnon_mito	91782	105513	CHMP7	Q8WUX9	charged multivesicular body protein 7	ENSG00000147457	453		NonMitoDomain	6	0.61	NoMitoHomolog	NoHomolog		-4.0022	0.6760000000000002	0		chr8	23101149	23119512																					Nucleus (APE, Uncertain)
chr8	23145611	23153792	Tnon_mito	203069	71843	R3HCC1	Q9Y3T6	R3H domain and coiled-coil containing 1	ENSG00000104679	253		NA	1		NoMitoHomolog	NoHomolog		-8.0704	0.8449999999999999	0		chr8	23145611	23153792																					Cytoplasm (APE, Uncertain)
chr8	23154409	23261722	Tnon_mito	4017	94352	LOXL2	LOR2|WS9-14|Q9Y4K0	lysyl oxidase-like 2	ENSG00000134013	774		NonMitoDomain	0		NoMitoHomolog	NoHomolog		-10.6961	0.887	0		chr8	23154409	23261722																					Nucleus but not nucleoli (APE, Supportive)
chr8	23286664	23315244	Tnon_mito	9583	67464	ENTPD4	LALP70|LAP70|LYSAL1|NTPDase-4|UDPase|Q9Y227	ectonucleoside triphosphate diphosphohydrolase 4	ENSG00000197217	616		NonMitoDomain	31		NoMitoHomolog	NoHomolog		-3.78	0.638	0		chr8	23286664	23315244																					Vesicles (APE, Supportive)
chr8	23386362	23430063	Tmito	51312	67712	SLC25A37	MFRN|MFRN1|MSC|MSCP|PRO1278|PRO1584|PRO2217|Q9NYZ2	solute carrier family 25 (mitochondrial iron transporter), member 37	ENSG00000147454	338		MitoDomain	4		OrthologMitoLowConf	NoHomolog		5.9313	0.04	1	literature, yeast mito homolog+, mito protein domain+	chr8	23386362	23430063
chr8	23536205	23540450	Tnon_mito	4824	18095	NKX3-1	BAPX2|NKX3|NKX3.1|NKX3A|Q99801	NK3 homeobox 1	ENSG00000167034	234		NonMitoDomain	2		NoMitoHomolog	NoHomolog		-10.6961	0.887	0		chr8	23536205	23540450
chr8	23559963	23564111	Tnon_mito	137814	18092	NKX2-6	CSX2|NKX2F|NKX4-2|A6NCS4	NK2 homeobox 6	ENSG00000180053	301		NonMitoDomain	1		NoMitoHomolog	NoHomolog		-10.6961	0.887	0		chr8	23559963	23564111
chr8	23699433	23712320	Tnon_mito	6781	20855	STC1	STC|P52823	stanniocalcin 1	ENSG00000159167	247		NonMitoDomain	1		NoMitoHomolog	NoHomolog		-10.6961	0.887	0		chr8	23699433	23712320
chr8	24151579	24212726	Tnon_mito	10863	13522	ADAM28	ADAM 28|ADAM23|MDC-L|MDC-Lm|MDC-Ls|MDCL|eMDC II|eMDCII|Q9UKQ2	ADAM metallopeptidase domain 28	ENSG00000042980	775		NonMitoDomain	0		NoMitoHomolog	NoHomolog		-10.6961	0.887	0		chr8	24151579	24212726
chr8	24241797	24263526	Tnon_mito	27299	58860	ADAMDEC1	M12.219|O15204	ADAM-like, decysin 1	ENSG00000134028	470		NonMitoDomain	1		NoMitoHomolog	NoHomolog		-10.6961	0.887	0		chr8	24241797	24263526
chr8	24298508	24367077	Tnon_mito	8756	11500	ADAM7	ADAM 7|ADAM-7|EAPI|GP-83|GP83|Q9H2U9	ADAM metallopeptidase domain 7	ENSG00000069206	754		NonMitoDomain	0		NoMitoHomolog	NoHomolog		-10.6961	0.887	0		chr8	24298508	24367077
chr8	24771273	24776606	Tnon_mito	4741	18040	NEFM	NEF3|NF-M|NFM|P07197	neurofilament, medium polypeptide	ENSG00000104722	916	5	NonMitoDomain	0		NoMitoHomolog	NoHomolog	0-25crude	-7.1786	0.83	0		chr8	24771273	24776606	3	19	29	222000000	25	brainstem, spinalcord, placenta			8.3	6.7									7.4		Cytoplasm (APE, Uncertain)
chr8	24808468	24814383	Tnon_mito	4747	18039	NEFL	CMT1F|CMT2E|NF-L|NF68|NFL|P07196	neurofilament, light polypeptide		543		NonMitoDomain	0		NoMitoHomolog	NoHomolog	0-25crude	-9.2075	0.8820000000000001	0		chr8	24808468	24814383	3	9	15	64400000	13	brainstem, spinalcord, placenta			7.3	7.3									7.4
chr8	25042286	25270619	Tnon_mito	80005	68813	DOCK5	Q9H7D0	dedicator of cytokinesis 5	ENSG00000147459	1870		SharedDomain	0		HomologMitoLowConf	NoHomolog	0-25ambig	-1.9426	0.49	0		chr8	25042286	25270619	1	3	3	97600000	2	largeintestine											8				Cytoplasm (APE, Supportive)
chr8	25276773	25282556	Tnon_mito	2796	14714	GNRH1	GNRH|GRH|HH12|LHRH|LNRH|P01148	gonadotropin-releasing hormone 1 (luteinizing-releasing hormone)	ENSG00000147437	96		NonMitoDomain	NA		NoMitoHomolog	NoHomolog		-10.6961	0.887	0		chr8	25276773	25282556
chr8	25285363	25315984	Tnon_mito	54793	105440	KCTD9	BTBD27|Q7L273	potassium channel tetramerization domain containing 9	ENSG00000104756	389	4	NonMitoDomain	4		NoMitoHomolog	Ortholog		-2.9969	0.5650000000000001	0		chr8	25285363	25315984																					Nucleus but not nucleoli (APE, Supportive)
chr8	25316512	25365425	Tnon_mito	157313	108912	CDCA2	Repo-Man|Q69YH5	cell division cycle associated 2	ENSG00000184661	1023		NonMitoDomain	2		NoMitoHomolog	NoHomolog		-10.6961	0.887	0		chr8	25316512	25365425																					Nucleus but not nucleoli (APE, Supportive)
chr8	25699245	25902640	Tnon_mito	64641	13592	EBF2	COE2|EBF-2|O/E-3|OE-3|Q9HAK2	early B-cell factor 2	ENSG00000221818	575		NonMitoDomain	15		NoMitoHomolog	NoHomolog		-6.3154	0.823	0		chr8	25699245	25902640
chr8	26149006	26230195	Tnon_mito	5520	71978	PPP2R2A	B55A|B55ALPHA|PR52A|PR55A|P63151	protein phosphatase 2, regulatory subunit B, alpha	ENSG00000221914	457		NA	0		NoMitoHomolog	NoHomolog		-8.0704	0.8449999999999999	0		chr8	26149006	26230195
chr8	26240522	26270644	Tmito	665	12177	BNIP3L	BNIP3a|NIX|O60238	BCL2/adenovirus E1B 19kDa interacting protein 3-like	ENSG00000104765	219		MitoDomain	5		NoMitoHomolog	NoHomolog		1.861	0.199	1	literature, mito protein domain+, coexpression	chr8	26240522	26270644																					Mitochondria (APE, Supportive)
chr8	26362195	26371483	Tnon_mito	10687	239157	PNMA2	MA2|MM2|RGAG2|Q9UL42	paraneoplastic Ma antigen 2	ENSG00000240694	364		NonMitoDomain	0		NoMitoHomolog	NoHomolog		-10.6961	0.887	0		chr8	26362195	26371483
chr8	26371708	26515693	Tpossible_mito	1808	12934	DPYSL2	CRMP-2|CRMP2|DHPRP2|DRP-2|DRP2|N2A3|ULIP-2|ULIP2|Q16555	dihydropyrimidinase-like 2	ENSG00000092964	677		NonMitoDomain	0		NoMitoHomolog	NoHomolog	50-75crude	-7.3762	0.8339999999999999	0		chr8	26371708	26515693	8	33	391	21300000000	73	cerebrum, cerebellum, brainstem, spinalcord, adipose, smallintestine, stomach, placenta	9.4	9.2	10.1	9.7					8	8.1		7.2	7		Cytoplasm (APE, Supportive)
chr8	26605666	26722922	Tnon_mito	148	11549	ADRA1A	ADRA1C|ADRA1L1|ALPHA1AAR|P35348	adrenoceptor alpha 1A	ENSG00000120907	475		NonMitoDomain	3		NoMitoHomolog	NoHomolog		-10.6961	0.887	0		chr8	26605666	26722922
chr8	27092839	27115956	Tnon_mito	81551	56471	STMN4	RB3|Q9H169	stathmin-like 4	ENSG00000015592	216		NonMitoDomain	0		NoMitoHomolog	NoHomolog		-10.6961	0.887	0		chr8	27092839	27115956
chr8	27142403	27168834	Tnon_mito	23087	66854	TRIM35	HLS5|MAIR|Q9UPQ4	tripartite motif containing 35	ENSG00000104228	493		SharedDomain	0		HomologMitoLowConf	NoHomolog		-4.3576	0.6930000000000001	0		chr8	27142403	27168834																					Nucleus but not nucleoli (APE, Supportive)
chr8	27168998	27316908	Tnon_mito	2185	19229	PTK2B	CADTK|CAKB|FADK2|FAK2|PKB|PTK|PYK2|RAFTK|Q14289	protein tyrosine kinase 2 beta	ENSG00000120899	1009		SharedDomain	0		NoMitoHomolog	NoHomolog	0-25ambig	-3.8147	0.648	0		chr8	27168998	27316908	1	3	4	15000000	4	cerebellum		7.2													Nucleus but not nucleoli;Cytoplasm (APE, Supportive)
chr8	27317277	27336813	Tnon_mito	1135	110902	CHRNA2	Q15822	cholinergic receptor, nicotinic, alpha 2 (neuronal)	ENSG00000120903	529		NonMitoDomain	NA		NoMitoHomolog	NoHomolog		-10.6961	0.887	0		chr8	27317277	27336813
chr8	27348518	27402439	Tnon_mito	2053	13850	EPHX2	CEH|SEH|P34913	epoxide hydrolase 2, cytoplasmic	ENSG00000120915	555		SharedDomain	21		NoMitoHomolog	Homolog	50-75crude	5.726	0.042	1	Rickettsial homolog, mito protein domain, coexpression	chr8	27348518	27402439	7	36	325	17500000000	74	kidney, liver, heart, skeletalmuscle, adipose, smallintestine, stomach					9.7	9.9	8.4	7.1	9.5	8.7		8.1			Cytoplasm (APE, Supportive)
chr8	27454433	27472328	Tpossible_mito	1191	12759	CLU	APO-J|APOJ|CLI|CLU1|CLU2|KUB1|NA1/NA2|SGP-2|SGP2|SP-40|TRPM-2|TRPM2|P10909	clusterin	ENSG00000120885	449		NA	0		NoMitoHomolog	NoHomolog	0-25ambig	-5.6553	0.751	0		chr8	27454433	27472328	3	5	19	140000000	11	stomach, placenta, testis												7.4	7.6	7.9
chr8	27491576	27534286	Tnon_mito	51435	219151	SCARA3	APC7|CSR|CSR1|MSLR1|MSRL1|Q6AZY7	scavenger receptor class A, member 3	ENSG00000168077	606		NonMitoDomain	0		NoMitoHomolog	NoHomolog		-10.6961	0.887	0		chr8	27491576	27534286
chr8	27590832	27630170	Tnon_mito	55246	67179	CCDC25	Q86WR0	coiled-coil domain containing 25	ENSG00000147419	208		NonMitoDomain	NA		NoMitoHomolog	NoHomolog		-10.6961	0.887	0		chr8	27590832	27630170																					Nucleus but not nucleoli (APE, Supportive)
chr8	27632057	27662424	Tnon_mito	157570	71988	ESCO2	2410004I17Rik|EFO2|RBS|Q56NI9	establishment of sister chromatid cohesion N-acetyltransferase 2	ENSG00000171320	601		NonMitoDomain	1		NoMitoHomolog	NoHomolog		-10.6961	0.887	0		chr8	27632057	27662424																					Nucleus (APE, Supportive)
chr8	27667137	27695612	Tnon_mito	55872	52033	PBK	CT84|HEL164|Nori-3|SPK|TOPK|Q96KB5	PDZ binding kinase	ENSG00000168078	333		SharedDomain	1		NoMitoHomolog	NoHomolog		-6.2297	0.767	0		chr8	27667137	27695612																					Cytoplasm (APE, Uncertain)
chr8	27727398	27850369	Tnon_mito	286133	71145	SCARA5	NET33|Tesr|Q6ZMJ2	scavenger receptor class A, member 5 (putative)	ENSG00000168079	495		NonMitoDomain	1		NoMitoHomolog	NoHomolog		-10.6961	0.887	0		chr8	27727398	27850369
chr8	27879480	27941388	Tnon_mito	389643	100503545	NUGGC	C8orf80|SLIP-GC|Q68CJ6	nuclear GTPase, germinal center associated	ENSG00000189233	796		SharedDomain	NA		NoMitoHomolog	NoHomolog		-6.2297	0.767	0		chr8	27879480	27941388
chr8	27947747	28048669	Tnon_mito	55140	74195	ELP3	KAT9|Q9H9T3	elongator acetyltransferase complex subunit 3	ENSG00000134014	547	2	SharedDomain	3		NoMitoHomolog	Homolog		2.0214	0.182	0		chr8	27947747	28048669
chr8	28174648	28200871	Tnon_mito	5368	18155	PNOC	PPNOC|Q13519	prepronociceptin	ENSG00000168081	176	4	NonMitoDomain	1		NoMitoHomolog	NoHomolog		-8.2616	0.8660000000000001	0		chr8	28174648	28200871
chr8	28203101	28243977	Tnon_mito	55893	380912	ZNF395	HDBP-2|HDBP2|HDRF-2|PBF|PRF-1|PRF1|Si-1-8-14|Q9H8N7	zinc finger protein 395	ENSG00000186918	513	2	NA	1		NoMitoHomolog	NoHomolog		-2.8234	0.539	0		chr8	28203101	28243977																					Nucleus (APE, Uncertain)
chr8	28285925	28347835	Tnon_mito	157574	50759	FBXO16	FBX16|Q8IX29	F-box protein 16	ENSG00000214050	292		SharedDomain	0		NoMitoHomolog	NoHomolog		-6.2297	0.767	0		chr8	28285925	28347835
chr8	28351721	28431785	Tnon_mito	7976	14365	FZD3	Fz-3|Q9NPG1	frizzled class receptor 3	ENSG00000104290	666		NonMitoDomain	0		NoMitoHomolog	NoHomolog		-10.6961	0.887	0		chr8	28351721	28431785																					Vesicles (APE, Uncertain)
chr8	28558989	28611207	Tnon_mito	2137	54616	EXTL3	BOTV|EXTL1L|EXTR1|REGR|RPR|O43909	exostosin-like glycosyltransferase 3	ENSG00000012232	919	5	NonMitoDomain	1	-0.25	NoMitoHomolog	NoHomolog		-7.9822	0.8439999999999999	0		chr8	28558989	28611207
chr8	28625174	28747698	Tnon_mito	55756	210925	INTS9	CPSF2L|INT9|RC74|Q9NV88	integrator complex subunit 9	ENSG00000104299	658		NonMitoDomain	0		NoMitoHomolog	NoHomolog		-10.6961	0.887	0		chr8	28625174	28747698																					Nucleus but not nucleoli (APE, Supportive)
chr8	28747910	28910242	Tnon_mito	79618	219150	HMBOX1	HNF1LA|HOT1|PBHNF|Q6NT76	homeobox containing 1	ENSG00000147421	420		NonMitoDomain	NA		NoMitoHomolog	NoHomolog		-10.6961	0.887	0		chr8	28747910	28910242																					Nucleus (APE, Supportive)
chr8	28924794	29120610	Tnon_mito	23303	16554	KIF13B	GAKIN|Q9NQT8	kinesin family member 13B	ENSG00000197892	1826		SharedDomain	0		NoMitoHomolog	NoHomolog	0-25ambig	-3.8147	0.648	0		chr8	28924794	29120610	7	10	16	420000000	7	brainstem, spinalcord, liver, smallintestine, largeintestine, stomach, placenta			8.1	5		7.3				7.9	8.3	5.3	6.6		Plasma membrane (APE, Uncertain)
chr8	29190578	29208267	Tnon_mito	1846	319520	DUSP4	HVH2|MKP-2|MKP2|TYP|Q13115	dual specificity phosphatase 4	ENSG00000120875	394	4	SharedDomain	NA	1.07	NoMitoHomolog	NoHomolog		0.0131	0.33	0		chr8	29190578	29208267																					Nucleus but not nucleoli (APE, Supportive)
chr8	29920527	29940724	Tnon_mito	51669	67887	TMEM66	FOAP-7|SARAF|XTP3|Q96BY9	transmembrane protein 66	ENSG00000133872	339		NonMitoDomain	0		NoMitoHomolog	NoHomolog		-10.6961	0.887	0		chr8	29920527	29940724
chr8	29952921	29995222	Tnon_mito	23484	68192	LEPROTL1	HSPC112|Vps55|my047|O95214	leptin receptor overlapping transcript-like 1	ENSG00000104660	169		NonMitoDomain	1		NoMitoHomolog	NoHomolog		-10.6961	0.887	0		chr8	29952921	29995222
chr8	29989186	30002200	Tnon_mito	619373	234155	MBOAT4	GOAT|OACT4|Q96T53	membrane bound O-acyltransferase domain containing 4	ENSG00000177669	435		NonMitoDomain	NA		NoMitoHomolog	NoHomolog		-10.6961	0.887	0		chr8	29989186	30002200
chr8	30013812	30041155	Tpossible_mito	10671	22428	DCTN6	WS-3|WS3|p27|O00399	dynactin 6	ENSG00000104671	190		SharedDomain	0		NoMitoHomolog	NoHomolog		-6.2297	0.767	0		chr8	30013812	30041155																					Nucleoli;Cytoplasm (APE, Supportive)
chr8	30242016	30429778	Tnon_mito	11030	19663	RBPMS	HERMES|Q93062	RNA binding protein with multiple splicing	ENSG00000157110	219		SharedDomain	0		NoMitoHomolog	NoHomolog		-6.2297	0.767	0		chr8	30242016	30429778																					Nucleus but not nucleoli (APE, Supportive)
chr8	30436030	30515738	Tnon_mito	2961	68153	GTF2E2	FE|TF2E2|TFIIE-B|P29084	general transcription factor IIE, polypeptide 2, beta 34kDa	ENSG00000197265	291		NonMitoDomain	1		NoMitoHomolog	NoHomolog		-10.6961	0.887	0		chr8	30436030	30515738																					Nucleus (APE, Supportive)
chr8	30496116	30503469	Tnon_mito	100507341	72632	SMIM18	P0DKX4	small integral membrane protein 18	ENSG00000253457	95		NA	NA		NoMitoHomolog	NoHomolog		-8.0704	0.8449999999999999	0		chr8	30496116	30503469
chr8	30535579	30585486	Tmito	2936	14782	GSR	HEL-75|P00390	glutathione reductase	ENSG00000104687	522	2	SharedDomain	4	-0.34	HomologMitoLowConf	Homolog	50-75pure	15.3163	0.003	1	literature, APEX_matrix, targetP signal, Rickettsial homolog, mito protein domain, MS/MS++	chr8	30535579	30585486	10	22	105	2400000000	55	spinalcord, kidney, liver, skeletalmuscle, adipose, smallintestine, largeintestine, stomach, placenta, testis				7.2	8.8	7.8		8.4	7.9	8.8	7.5	7.7	8.7	8.1	Cytoplasm (APE, Supportive)
chr8	30601681	30624520	Tnon_mito	7993	108159	UBXN8	D8S2298E|REP8|UBXD6|O00124	UBX domain protein 8		270		NonMitoDomain	2		NoMitoHomolog	NoHomolog		-10.6961	0.887	0		chr8	30601681	30624520
chr8	30643125	30670352	Tnon_mito	5516	19053	PPP2CB	PP2Abeta|PP2CB|P62714	protein phosphatase 2, catalytic subunit, beta isozyme	ENSG00000104695	309		NonMitoDomain	3	-0.38	NoMitoHomolog	NoHomolog		-10.0112	0.8830000000000001	0		chr8	30643125	30670352
chr8	30689059	30706533	Tnon_mito	56154	104271	TEX15	CT42|Q9BXT5	testis expressed 15	ENSG00000133863	2789		NA	1		NoMitoHomolog	NoHomolog		-8.0704	0.8449999999999999	0		chr8	30689059	30706533																					Nucleus but not nucleoli (APE, Uncertain)
chr8	30853320	30891231	Tnon_mito	29942	75029	PURG	PURG-A|PURG-B|Q9UJV8	purine-rich element binding protein G	ENSG00000172733	347		NonMitoDomain	3		NoMitoHomolog	NoHomolog		-10.6961	0.887	0		chr8	30853320	30891231
chr8	30890777	31031277	Tnon_mito	7486	22427	WRN	RECQ3|RECQL2|RECQL3|Q14191	Werner syndrome, RecQ helicase-like	ENSG00000165392	1432		SharedDomain	5		NoMitoHomolog	Homolog	0-25ambig	2.0751	0.17300000000000001	0		chr8	30890777	31031277	1	4	4	50600000	3	cerebellum		7.7													Nucleus but not nucleoli (APE, Supportive)
chr8	31497267	32622558	Tnon_mito	3084	211323	NRG1	ARIA|GGF|GGF2|HGL|HRG|HRG1|HRGA|MST131|NDF|SMDF|Q02297	neuregulin 1	ENSG00000157168	645	4	NonMitoDomain	NA		NoMitoHomolog	NoHomolog		-8.2616	0.8660000000000001	0		chr8	31497267	32622558
chr8	33228343	33330664	Tnon_mito	84750	171167	FUT10	Q6P4F1	fucosyltransferase 10 (alpha (1,3) fucosyltransferase)	ENSG00000172728	479		NonMitoDomain	0		NoMitoHomolog	NoHomolog		-10.6961	0.887	0		chr8	33228343	33330664																					Nucleus but not nucleoli;Endoplasmic reticulum;Golgi apparatus (APE, Uncertain)
chr8	33342684	33358778	Tnon_mito	84549	67920	MAK16	MAK16L|RBM13|Q9BXY0	MAK16 homolog (S. cerevisiae)	ENSG00000198042	300		NonMitoDomain	0		NoMitoHomolog	NoHomolog		-10.6961	0.887	0		chr8	33342684	33358778																					Nucleoli;Vesicles (APE, Supportive)
chr8	33356026	33370703	Tnon_mito	80185	234138	TTI2	C8orf41|MRT39|Q6NXR4	TELO2 interacting protein 2	ENSG00000129696	508		NA	NA		NoMitoHomolog	NoHomolog		-8.0704	0.8449999999999999	0		chr8	33356026	33370703																					Centrosome (APE, Supportive)
chr8	33405271	33424646	Tnon_mito	79845	68867	RNF122	Q9H9V4	ring finger protein 122	ENSG00000133874	155		SharedDomain	0	-0.34	NoMitoHomolog	NoHomolog		-5.5448	0.7390000000000001	0		chr8	33405271	33424646
chr8	33448853	33457439	Tmito	78986	66959	DUSP26	DUSP24|LDP-4|MKP8|NATA1|SKRP3|Q9BV47	dual specificity phosphatase 26 (putative)	ENSG00000133878	211	2	SharedDomain	2		NoMitoHomolog	NoHomolog		-0.9827	0.42	1	GFP, targetP signal, mito protein domain	chr8	33448853	33457439
chr8	35092974	35652181	Tnon_mito	137970	210801	UNC5D	PRO34692|Unc5h4|Q6UXZ4	unc-5 homolog D (C. elegans)	ENSG00000156687	953		NonMitoDomain	1		NoMitoHomolog	NoHomolog		-10.6961	0.887	0		chr8	35092974	35652181
chr8	36641841	36793643	Tnon_mito	157855	16532	KCNU1	KCNMC1|KCa5|KCa5.1|Kcnma3|Slo3|A8MYU2	potassium channel, subfamily U, member 1	ENSG00000215262	1149		NonMitoDomain	1		NoMitoHomolog	NoHomolog		-10.6961	0.887	0		chr8	36641841	36793643
chr8	37553300	37556396	Tnon_mito	80139	353310	ZNF703	ZEPPO1|ZNF503L|ZPO1|Q9H7S9	zinc finger protein 703	ENSG00000183779	590		NonMitoDomain	2		NoMitoHomolog	NoHomolog		-10.6961	0.887	0		chr8	37553300	37556396
chr8	37594096	37615319	Tnon_mito	11160	244373	ERLIN2	C8orf2|Erlin-2|NET32|SPFH2|SPG18|O94905	ER lipid raft associated 2	ENSG00000147475	339		SharedDomain	0	0.2	NoMitoHomolog	NoHomolog	0-25crude	-4.0561	0.6780000000000002	0		chr8	37594096	37615319	2	6	8	86500000	20	placenta, testis													6.9	7.9	Endoplasmic reticulum (APE, Supportive)
chr8	37620100	37637286	Tmito	11212	114863	PROSC	O94903	proline synthetase co-transcribed homolog (bacterial)	ENSG00000147471	275	4	NonMitoDomain	9		NoMitoHomolog	NoHomolog	50-75pure	5.3618	0.049	1	APEX_matrix, coexpression, MS/MS++	chr8	37620100	37637286	14	15	127	2760000000	65	all 14	8.4	7.8	8.2	8.4	8.4	7.9	8	7	8.3	8.2	8.1	7.8	9	7.8	Cytoplasm (APE, Uncertain)
chr8	37654400	37701504	Tnon_mito	25960	78560	GPR124	TEM5|Q96PE1	G protein-coupled receptor 124	ENSG00000020181	1338		SharedDomain	0		NoMitoHomolog	NoHomolog		-6.2297	0.767	0		chr8	37654400	37701504
chr8	37701397	37707431	Tnon_mito	55290	66653	BRF2	BRFU|TFIIIB50|Q9HAW0	BRF2, RNA polymerase III transcription initiation factor 50 kDa subunit	ENSG00000104221	419		NonMitoDomain	3		NoMitoHomolog	NoHomolog		-10.6961	0.887	0		chr8	37701397	37707431																					Nucleoli (APE, Uncertain)
chr8	37716464	37757015	Tnon_mito	80223	75767	RAB11FIP1	NOEL1A|RCP|rab11-FIP1|Q6WKZ4	RAB11 family interacting protein 1 (class I)	ENSG00000156675	1283	2	NonMitoDomain	2		NoMitoHomolog	NoHomolog		-5.4491	0.7330000000000001	0		chr8	37716464	37757015																					Vesicles (APE, Supportive)
chr8	37791799	37797647	Tnon_mito	137362	76615	GOT1L1	Q8NHS2	glutamic-oxaloacetic transaminase 1-like 1	ENSG00000169154	421		SharedDomain	1		NoMitoHomolog	NoHomolog		-6.2297	0.767	0		chr8	37791799	37797647
chr8	37820513	37824184	Tnon_mito	155	11556	ADRB3	BETA3AR|P13945	adrenoceptor beta 3	ENSG00000188778	408		NonMitoDomain	6		NoMitoHomolog	NoHomolog		-7.8105	0.8389999999999999	0		chr8	37820513	37824184
chr8	37888019	37917883	Tnon_mito	1978	13685	EIF4EBP1	4E-BP1|4EBP1|BP-1|PHAS-I|Q13541	eukaryotic translation initiation factor 4E binding protein 1	ENSG00000187840	118	4	NA	8	0.78	NoMitoHomolog	NoHomolog		1.058	0.249	0		chr8	37888019	37917883																					Nucleus but not nucleoli (APE, Supportive)
chr8	37963010	37997598	Tnon_mito	9070	23808	ASH2L	ASH2|ASH2L1|ASH2L2|Bre2|Q9UBL3	ash2 (absent, small, or homeotic)-like (Drosophila)	ENSG00000129691	628		SharedDomain	0		NoMitoHomolog	NoHomolog		-6.2297	0.767	0		chr8	37963010	37997598																					Nucleus but not nucleoli (Staining, Uncertain)
chr8	38000217	38008600	Tmito	6770	20845	STAR	STARD1|P49675	steroidogenic acute regulatory protein	ENSG00000147465	285	2	SharedDomain	8		NoMitoHomolog	NoHomolog	25-50ambig	7.3188	0.027000000000000003	1	literature, targetP signal, mito protein domain, coexpression, MS/MS	chr8	38000217	38008600	1	7	8	326000000	33	testis														8.5
chr8	38020838	38034248	Tnon_mito	27257	67207	LSM1	CASM|YJL124C|O15116	LSM1, U6 small nuclear RNA associated	ENSG00000175324	133		NonMitoDomain	8	0.89	NoMitoHomolog	NoHomolog		-4.0022	0.6760000000000002	0		chr8	38020838	38034248																					Cytoplasm;Vesicles (APE, Supportive)
chr8	38034105	38070819	Tnon_mito	9530	67384	BAG4	BAG-4|SODD|O95429	BCL2-associated athanogene 4	ENSG00000156735	457		NonMitoDomain	1		NoMitoHomolog	NoHomolog		-10.6961	0.887	0		chr8	38034105	38070819																					Nucleus but not nucleoli (APE, Uncertain)
chr8	38089008	38120287	Tnon_mito	23259	72108	DDHD2	SAMWD1|SPG54|iPLA(1)gamma|O94830	DDHD domain containing 2	ENSG00000085788	711		SharedDomain	14		HomologMitoLowConf	NoHomolog		0.023100000000000002	0.327	0		chr8	38089008	38120287																					Cytoplasm (APE, Supportive)
chr8	38120649	38126738	Tnon_mito	84513	71910	PPAPDC1B	DPPL1|HTPAP|Q8NEB5	phosphatidic acid phosphatase type 2 domain containing 1B	ENSG00000147535	264		NonMitoDomain	4		NoMitoHomolog	NoHomolog		-10.6961	0.887	0		chr8	38120649	38126738																					Vesicles (APE, Uncertain)
chr8	38132560	38239790	Tnon_mito	54904	234135	WHSC1L1	NSD3|pp14328|Q9BZ95	Wolf-Hirschhorn syndrome candidate 1-like 1	ENSG00000147548	1437		NonMitoDomain	0		NoMitoHomolog	NoHomolog	0-25ambig	-8.2811	0.8690000000000001	0		chr8	38132560	38239790	1	2	3	28600000	2	kidney					7.5
chr8	38243958	38267042	Tpossible_mito	137994	270035	LETM2	Q2VYF4	leucine zipper-EF-hand containing transmembrane protein 2	ENSG00000165046	491	2	MitoDomain	0		OrthologMitoLowConf	NoHomolog		11.1783	0.007	1	targetP signal, yeast mito homolog+, mito protein domain+	chr8	38243958	38267042																					Nucleus;Cytoplasm (APE, Uncertain)
chr8	38268655	38326352	Tnon_mito	2260	14182	FGFR1	BFGFR|CD331|CEK|FGFBR|FGFR-1|FLG|FLT-2|FLT2|HBGFR|HH2|HRTFDS|KAL2|N-SAM|OGD|bFGF-R-1|P11362	fibroblast growth factor receptor 1	ENSG00000077782	853		SharedDomain	1		NoMitoHomolog	NoHomolog		-6.2297	0.767	0		chr8	38268655	38326352
chr8	38368351	38386180	Tnon_mito	389649		C8orf86	Q6ZUL3	chromosome 8 open reading frame 86	ENSG00000196166	223		NA			NoMitoHomolog	NoHomolog		-10.8297	0.921	0		chr8	38368351	38386180
chr8	38585703	38710546	Tnon_mito	6867	320165	TACC1	Ga55|O75410	transforming, acidic coiled-coil containing protein 1	ENSG00000147526	805		NonMitoDomain	0		NoMitoHomolog	NoHomolog		-10.6961	0.887	0		chr8	38585703	38710546																					Cytoskeleton (Intermediate filaments);Cytoskeleton (Microtubules) (APE, Uncertain)
chr8	38758752	38831430	Tnon_mito	59339	83436	PLEKHA2	TAPP2|Q9HB19	pleckstrin homology domain containing, family A (phosphoinositide binding specific) member 2	ENSG00000169499	425		SharedDomain	0		NoMitoHomolog	NoHomolog		-6.2297	0.767	0		chr8	38758752	38831430
chr8	38831667	38846181	Tnon_mito	203100	330723	HTRA4	P83105	HtrA serine peptidase 4	ENSG00000169495	476		SharedDomain	0		NoMitoHomolog	Homolog	0-25ambig	-0.8105	0.385	0		chr8	38831667	38846181	3	3	7	146000000	8	liver, adipose, placenta						7.1			7.9				7.8
chr8	38846326	38854041	Tnon_mito	83877	69742	TM2D2	BLP1|Q9BX73	TM2 domain containing 2	ENSG00000169490	214		NonMitoDomain	10		NoMitoHomolog	NoHomolog		-6.3154	0.823	0		chr8	38846326	38854041																					Nucleus but not nucleoli;Plasma membrane;Vesicles (APE, Uncertain)
chr8	38854504	38962779	Tnon_mito	8754	11502	ADAM9	CORD9|MCMP|MDC9|Mltng|Q13443	ADAM metallopeptidase domain 9	ENSG00000168615	819		NonMitoDomain	2		NoMitoHomolog	NoHomolog		-10.6961	0.887	0		chr8	38854504	38962779
chr8	38965049	39142436	Tnon_mito	203102	353188	ADAM32	Q8TC27	ADAM metallopeptidase domain 32	ENSG00000197140	787		NonMitoDomain	2		NoMitoHomolog	NoHomolog		-10.6961	0.887	0		chr8	38965049	39142436
chr8	39442086	39587583	Tnon_mito	8749	13524	ADAM18	ADAM27|tMDCIII|Q9Y3Q7	ADAM metallopeptidase domain 18	ENSG00000168619	739		NonMitoDomain	2		NoMitoHomolog	NoHomolog	0-25ambig	-8.2811	0.8690000000000001	0		chr8	39442086	39587583	1	5	5	31100000	9	testis														7.5
chr8	39601254	39695808	Tnon_mito	2515	11495	ADAM2	CRYN1|CRYN2|CT15|FTNB|PH-30b|PH30|PH30-beta|Q99965	ADAM metallopeptidase domain 2	ENSG00000104755	735		NonMitoDomain	2		NoMitoHomolog	NoHomolog	0-25crude	-9.2075	0.8820000000000001	0		chr8	39601254	39695808	1	3	3	24200000	5	testis														7.4	Plasma membrane (APE, Uncertain)
chr8	39771327	39786309	Tnon_mito	3620	15930	IDO1	IDO|IDO-1|INDO|P14902	indoleamine 2,3-dioxygenase 1	ENSG00000131203	403		NA	0		NoMitoHomolog	NoHomolog		-8.0704	0.8449999999999999	0		chr8	39771327	39786309																					Nucleus but not nucleoli;Mitochondria;Vesicles;Cytoskeleton (Microtubules) (APE, Uncertain)
chr8	39792473	39873910	Tnon_mito	169355	209176	IDO2	INDOL1|Q6ZQW0	indoleamine 2,3-dioxygenase 2	ENSG00000188676	420		NA	2		NoMitoHomolog	NoHomolog		-8.0704	0.8449999999999999	0		chr8	39792473	39873910
chr8	40010986	40012827	Tnon_mito	56892	69068	C8orf4	TC-1|TC1|Q9NR00	chromosome 8 open reading frame 4	ENSG00000176907	106	3	NA	1		NoMitoHomolog	NoHomolog		-4.7007	0.7090000000000001	0		chr8	40010986	40012827
chr8	40388110	40755343	Tnon_mito	79698	320158	ZMAT4	Q9H898	zinc finger, matrin-type 4	ENSG00000165061	229		NonMitoDomain	0		NoMitoHomolog	NoHomolog		-10.6961	0.887	0		chr8	40388110	40755343
chr8	41119475	41166990	Tnon_mito	6422	20377	SFRP1	FRP|FRP-1|FRP1|FrzA|SARP2|Q8N474	secreted frizzled-related protein 1	ENSG00000104332	314		NonMitoDomain	0		NoMitoHomolog	NoHomolog		-10.6961	0.887	0		chr8	41119475	41166990
chr8	41348080	41368499	Tnon_mito	51125	57437	GOLGA7	GCP16|GOLGA3AP1|GOLGA7A|HSPC041|Q7Z5G4	golgin A7	ENSG00000147533	137	5	NonMitoDomain	NA		NoMitoHomolog	NoHomolog		-8.6672	0.875	0		chr8	41348080	41368499
chr8	41386724	41402565	Tnon_mito	84296	109145	GINS4	SLD5|Q9BRT9	GINS complex subunit 4 (Sld5 homolog)	ENSG00000147536	223		NonMitoDomain	0		NoMitoHomolog	NoHomolog		-10.6961	0.887	0		chr8	41386724	41402565																					Nucleus but not nucleoli (Staining, Uncertain)
chr8	41435706	41482520	Tnon_mito	137964	102247	AGPAT6	1-AGPAT 6|LPAAT-zeta|LPAATZ|TSARG7|Q86UL3	1-acylglycerol-3-phosphate O-acyltransferase 6	ENSG00000158669	456		SharedDomain	1		NoMitoHomolog	NoHomolog		-6.2297	0.767	0		chr8	41435706	41482520
chr8	41503828	41504878	Tnon_mito	157848		NKX6-3	NKX6.3|A6NJ46	NK6 homeobox 3	ENSG00000165066	135		NA			NoMitoHomolog	NoHomolog		-10.8297	0.921	0		chr8	41503828	41504878
chr8	41510743	41754280	Tnon_mito	286	11733	ANK1	ANK|SPH1|SPH2|P16157	ankyrin 1, erythrocytic	ENSG00000029534	1897		SharedDomain	0		NoMitoHomolog	Homolog	0-25crude	-1.7369	0.47	0		chr8	41510743	41754280	2	19	26	197000000	14	brainstem, placenta			8.1										7.8		Nucleus but not nucleoli;Cytoplasm;Cell Junctions (APE, Uncertain)
chr8	41786996	41909505	Tnon_mito	7994	244349	KAT6A	MOZ|MYST3|RUNXBP2|ZC2HC6A|ZNF220|Q92794	K(lysine) acetyltransferase 6A	ENSG00000083168	2004		NonMitoDomain	0		NoMitoHomolog	NoHomolog		-10.6961	0.887	0		chr8	41786996	41909505																					Nucleus but not nucleoli (APE, Supportive)
chr8	42010463	42028701	Tnon_mito	10947	64933	AP3M2	AP47B|CLA20|P47B|P53677	adaptor-related protein complex 3, mu 2 subunit	ENSG00000070718	418		NonMitoDomain	0	-0.15	NoMitoHomolog	NoHomolog	0-25ambig	-7.5961	0.8369999999999999	0		chr8	42010463	42028701	1	4	4	61200000	16	placenta													7.8
chr8	42032235	42065194	Tnon_mito	5327	18791	PLAT	T-PA|TPA|P00750	plasminogen activator, tissue	ENSG00000104368	562		SharedDomain	0	-0.5800000000000001	NoMitoHomolog	NoHomolog		-5.5448	0.7390000000000001	0		chr8	42032235	42065194																					Cytoskeleton (Actin filaments) (APE, Uncertain)
chr8	42128819	42190171	Tnon_mito	3551	16150	IKBKB	IKK-beta|IKK2|IKKB|IMD15|NFKBIKB|O14920	inhibitor of kappa light polypeptide gene enhancer in B-cells, kinase beta	ENSG00000104365	756	4	SharedDomain	0	-0.30000000000000004	NoMitoHomolog	NoHomolog		-3.1103	0.5700000000000001	0		chr8	42128819	42190171																					Cytoplasm (APE, Supportive)
chr8	42195972	42229331	Tnon_mito	5423	18970	POLB	P06746	polymerase (DNA directed), beta	ENSG00000070501	335		NonMitoDomain	3		NoMitoHomolog	NoHomolog		-10.6961	0.887	0		chr8	42195972	42229331																					Vesicles (APE, Uncertain)
chr8	42231585	42234674	Tnon_mito	27121	234130	DKK4	DKK-4|Q9UBT3	dickkopf WNT signaling pathway inhibitor 4	ENSG00000104371	224		NonMitoDomain	NA		NoMitoHomolog	NoHomolog		-10.6961	0.887	0		chr8	42231585	42234674
chr8	42249278	42263455	Tmito	7419	22335	VDAC3	HD-VDAC3|VDAC-3|Q9Y277	voltage-dependent anion channel 3	ENSG00000078668	284		MitoDomain	NA		OrthologMitoLowConf	NoHomolog	75-100pure	17.5999	0	1	literature, yeast mito homolog+, mito protein domain+, MS/MS++	chr8	42249278	42263455	14	25	2048	167000000000	80	all 14	10.1	9.7	10.1	10.3	9.8	9.5	10.1	9.6	9.9	10.1	10.2	9.6	10.2	10.6	Mitochondria (APE, Supportive)
chr8	42273979	42397356	Tnon_mito	6575	20516	SLC20A2	GLVR-2|GLVR2|IBGC1|IBGC3|MLVAR|PIT-2|PIT2|RAM1|Q08357	solute carrier family 20 (phosphate transporter), member 2	ENSG00000168575	652		NonMitoDomain	1	-0.83	NoMitoHomolog	NoHomolog		-10.0112	0.8830000000000001	0		chr8	42273979	42397356
chr8	42396297	42408140	Tnon_mito	114926	102032	SMIM19	C8orf40|Q96E16	small integral membrane protein 19	ENSG00000176209	107		NA	35	0.62	NoMitoHomolog	NoHomolog		2.654	0.152	0		chr8	42396297	42408140
chr8	42552561	42592209	Tnon_mito	1142	108043	CHRNB3	Q05901	cholinergic receptor, nicotinic, beta 3 (neuronal)	ENSG00000147432	458		NonMitoDomain	0		NoMitoHomolog	NoHomolog		-10.6961	0.887	0		chr8	42552561	42592209
chr8	42607779	42623929	Tnon_mito	8973	11440	CHRNA6	CHNRA6|Q15825	cholinergic receptor, nicotinic, alpha 6 (neuronal)	ENSG00000147434	494		NonMitoDomain	0		NoMitoHomolog	NoHomolog		-10.6961	0.887	0		chr8	42607779	42623929
chr8	42691816	42698474	Tnon_mito	55145	73754	THAP1	DYT6|Q9NVV9	THAP domain containing, apoptosis associated protein 1	ENSG00000131931	213		NonMitoDomain	1		NoMitoHomolog	NoHomolog		-10.6961	0.887	0		chr8	42691816	42698474
chr8	42704779	42751866	Tnon_mito	81790	77733	RNF170	ADSA|SNAX1|Q96K19	ring finger protein 170	ENSG00000120925	258		SharedDomain	3		HomologMitoLowConf	NoHomolog		-4.3576	0.6930000000000001	0		chr8	42704779	42751866
chr8	42752032	42885682	Tnon_mito	84376	320191	HOOK3	HK3|Q86VS8	hook microtubule-tethering protein 3	ENSG00000168172	718		NonMitoDomain	2		NoMitoHomolog	NoHomolog		-10.6961	0.887	0		chr8	42752032	42885682																					Cytoplasm;Microtubule organizing center (APE, Supportive)
chr8	42911441	42940932	Tnon_mito	2339	14272	FNTA	FPTA|PGGT1A|PTAR2|P49354	farnesyltransferase, CAAX box, alpha	ENSG00000168522	379		NA	1		NoMitoHomolog	NoHomolog		-8.0704	0.8449999999999999	0		chr8	42911441	42940932
chr8	42948648	42978323	Tnon_mito	84197	74653	POMK	MDDGA12|SGK196|Q9H5K3	protein-O-mannose kinase	ENSG00000185900	350		SharedDomain	1		NoMitoHomolog	NoHomolog		-6.2297	0.767	0		chr8	42948648	42978323																					Nucleus (APE, Uncertain)
chr8	42995591	43057970	Tnon_mito	138050	52120	HGSNAT	HGNAT|MPS3C|TMEM76|Q68CP4	heparan-alpha-glucosaminide N-acetyltransferase	ENSG00000165102	635		NA	0		NoMitoHomolog	NoHomolog	0-25ambig	-5.6553	0.751	0		chr8	42995591	43057970	2	5	18	80200000	12	placenta, testis													7.9	6.8
chr8	43147584	43218328	Tnon_mito	340441		POTEA	A26A1|CT104.3|POTE-8|POTE8|Q6S8J7	POTE ankyrin domain family, member A		498		SharedDomain			NoMitoHomolog	Homolog		-5.9849	0.76	0		chr8	43147584	43218328
chr8	48173469	48648563	Tnon_mito	23514	224008	SPIDR	KIAA0146|Q14159	scaffolding protein involved in DNA repair	ENSG00000164808	915	5	NonMitoDomain	2		NoMitoHomolog	NoHomolog		-8.6672	0.875	0		chr8	48173469	48648563																					Nucleus but not nucleoli (APE, Supportive)
chr8	48649475	48650726	Tnon_mito	1052	12609	CEBPD	C/EBP-delta|CELF|CRP3|NF-IL6-beta|P49716	CCAAT/enhancer binding protein (C/EBP), delta	ENSG00000221869	269		SharedDomain	1		NoMitoHomolog	NoHomolog		-6.2297	0.767	0		chr8	48649475	48650726
chr8	48685668	48872743	Tnon_mito	5591	19090	PRKDC	DNA-PKcs|DNAPK|DNPK1|HYRC|HYRC1|XRCC7|p350|P78527	protein kinase, DNA-activated, catalytic polypeptide	ENSG00000253729	4128	3	SharedDomain	3		NoMitoHomolog	NoHomolog	0-25ambig	-0.445	0.36	0		chr8	48685668	48872743	7	20	30	422000000	6	cerebrum, brainstem, spinalcord, skeletalmuscle, smallintestine, stomach, placenta	7.1		8	7.2				7.9		7.9		7.8	7.8		Nucleus but not nucleoli (APE, Supportive)
chr8	48872762	48890719	Tnon_mito	4173	17217	MCM4	CDC21|CDC54|NKCD|NKGCD|P1-CDC21|hCdc21|P33991	minichromosome maintenance complex component 4	ENSG00000104738	863	5	SharedDomain	1		NoMitoHomolog	NoHomolog		-4.2008	0.6820000000000002	0		chr8	48872762	48890719																					Nucleus but not nucleoli (APE, Supportive)
chr8	48920994	48974454	Tnon_mito	7336	70620	UBE2V2	DDVIT1|DDVit-1|EDAF-1|EDPF-1|EDPF1|MMS2|UEV-2|UEV2|Q15819	ubiquitin-conjugating enzyme E2 variant 2	ENSG00000169139	145		NonMitoDomain	1		NoMitoHomolog	NoHomolog	25-50ambig	-5.2802	0.7250000000000001	0		chr8	48920994	48974454	7	7	46	744000000	37	cerebrum, cerebellum, brainstem, spinalcord, adipose, placenta, testis	7.4	7.7	8.7	8					6.9				7.6	7.8
chr8	49627473	49647870	Tnon_mito	79645	66793	EFCAB1	Q9HAE3	EF-hand calcium binding domain 1	ENSG00000034239	211		SharedDomain	0		NoMitoHomolog	NoHomolog		-6.2297	0.767	0		chr8	49627473	49647870
chr8	49830238	49833999	Tnon_mito	6591	20583	SNAI2	SLUG|SLUGH1|SNAIL2|WS2D|O43623	snail family zinc finger 2	ENSG00000019549	268		NonMitoDomain	1	-0.66	NoMitoHomolog	NoHomolog		-10.0112	0.8830000000000001	0		chr8	49830238	49833999																					Nucleus (APE, Supportive)
chr8	49966894	49988642	Tnon_mito	492307		C8orf22	Q8WWR9	chromosome 8 open reading frame 22	ENSG00000168333	84	3	NA			NoMitoHomolog	NoHomolog		-7.46	0.8369999999999999	0		chr8	49966894	49988642
chr8	50822348	51706678	Tnon_mito	54212	71096	SNTG1	G1SYN|SYN4|Q9NSN8	syntrophin, gamma 1	ENSG00000147481	517		SharedDomain	NA		NoMitoHomolog	NoHomolog		-6.2297	0.767	0		chr8	50822348	51706678
chr8	52232136	52722005	Tnon_mito	137902		PXDNL	PMR1|PRM1|VPO2|A1KZ92	peroxidasin homolog (Drosophila)-like	ENSG00000147485	1463		SharedDomain			NoMitoHomolog	NoHomolog		-8.989	0.879	0		chr8	52232136	52722005																					Nucleus but not nucleoli;Cytoplasm (APE, Uncertain)
chr8	52730134	52811746	Tnon_mito	115294	319263	PCMTD1	Q96MG8	protein-L-isoaspartate (D-aspartate) O-methyltransferase domain containing 1	ENSG00000168300	357		NonMitoDomain	12		NoMitoHomolog	NoHomolog		-6.3154	0.823	0		chr8	52730134	52811746																					Golgi apparatus (APE, Uncertain)
chr8	53023391	53322439	Tnon_mito	9705	240690	ST18	ZC2HC10|ZNF387|O60284	suppression of tumorigenicity 18 (breast carcinoma) (zinc finger protein)	ENSG00000147488	1047		NonMitoDomain	0		NoMitoHomolog	NoHomolog	0-25ambig	-8.2811	0.8690000000000001	0		chr8	53023391	53322439	2	7	9	195000000	7	spinalcord, largeintestine				8.1							7.9
chr8	53446596	53478021	Tnon_mito	389658	620393	FAM150A	UNQ9433|Q6UXT8	family with sequence similarity 150, member A	ENSG00000196711	129		NA	NA		NoMitoHomolog	NoHomolog		-8.0704	0.8449999999999999	0		chr8	53446596	53478021
chr8	53535017	53627026	Tnon_mito	9821	12421	RB1CC1	ATG17|CC1|FIP200|Q8TDY2	RB1-inducible coiled-coil 1	ENSG00000023287	1594		NonMitoDomain	2	0.51	NoMitoHomolog	NoHomolog	0-25ambig	-4.4728	0.7070000000000001	0		chr8	53535017	53627026	3	6	7	97900000	4	brainstem, kidney, testis			7.6		6.7									7.8	Cytoplasm (APE, Supportive)
chr8	53852467	53853454	Tnon_mito	2831	226304	NPBWR1	GPR7|P48145	neuropeptides B/W receptor 1	ENSG00000183729	328		NonMitoDomain	NA		NoMitoHomolog	NoHomolog		-10.6961	0.887	0		chr8	53852467	53853454
chr8	54138275	54164257	Tnon_mito	4986	18387	OPRK1	K-OR-1|KOR|KOR-1|OPRK|P41145	opioid receptor, kappa 1	ENSG00000082556	380		NonMitoDomain	0		NoMitoHomolog	NoHomolog		-10.6961	0.887	0		chr8	54138275	54164257
chr8	54628102	54755871	Tnon_mito	51606	108664	ATP6V1H	MSTP042|NBP1|SFD|SFDalpha|SFDbeta|VMA13|Q9UI12	ATPase, H+ transporting, lysosomal 50/57kDa, V1 subunit H	ENSG00000047249	483		NonMitoDomain	1		NoMitoHomolog	NoHomolog	25-50crude	-7.9301	0.8429999999999999	0		chr8	54628102	54755871	5	9	27	452000000	28	cerebrum, cerebellum, brainstem, spinalcord, placenta	7.6	7.4	8	8.1									8.2		Cytoskeleton (Actin filaments) (Staining, Uncertain)
chr8	54764367	54871864	Tnon_mito	8601	58175	RGS20	RGSZ1|ZGAP1|g(z)GAP|gz-GAP|O76081	regulator of G-protein signaling 20	ENSG00000147509	388		SharedDomain	1		NoMitoHomolog	NoHomolog		-6.2297	0.767	0		chr8	54764367	54871864
chr8	54879113	54935016	Tnon_mito	6917	21399	TCEA1	GTF2S|SII|TCEA|TF2S|TFIIS|P23193	transcription elongation factor A (SII), 1	ENSG00000187735	301		NonMitoDomain	NA		NoMitoHomolog	NoHomolog		-10.6961	0.887	0		chr8	54879113	54935016																					Nucleus (APE, Supportive)
chr8	54958926	55014577	Tpossible_mito	10434	18777	LYPLA1	APT-1|APT1|LPL-I|LPL1|hAPT1|O75608	lysophospholipase I	ENSG00000120992	230	4	SharedDomain	9		NoMitoHomolog	Homolog	75-100pure	13.7631	0.004	1	Rickettsial homolog, mito protein domain, coexpression, MS/MS++	chr8	54958926	55014577	14	14	204	9900000000	77	all 14	8.6	8.4	8.9	8.8	9.6	9.1	8.5	7.7	8.5	8.7	8.8	8.6	8.6	8.2
chr8	55047780	55061074	Tmito	29088	27395	MRPL15	L15mt|MRP-L15|MRP-L7|RPML7|Q9P015	mitochondrial ribosomal protein L15	ENSG00000137547	296	4	SharedDomain	9	2.17	OrthologMitoHighConf	Ortholog	50-75ambig	25.0806	0	1	literature, APEX_matrix, yeast mito homolog++, Rickettsial homolog, mito protein domain, induction, coexpression, MS/MS	chr8	55047780	55061074	12	22	118	3160000000	68	cerebrum, cerebellum, brainstem, spinalcord, kidney, liver, adipose, smallintestine, largeintestine, stomach, placenta, testis	8	7.6	7.3	8	8.3	7.5			8.4	8.4	9.1	7.4	8.9	8.4
chr8	55370494	55373456	Tnon_mito	64321	20671	SOX17	VUR3|Q9H6I2	SRY (sex determining region Y)-box 17	ENSG00000164736	414		SharedDomain	0		NoMitoHomolog	NoHomolog		-6.2297	0.767	0		chr8	55370494	55373456																					Nucleus but not nucleoli (APE, Uncertain)
chr8	55528626	55543394	Tnon_mito	6101	19888	RP1	DCDC4A|ORP1|P56715	retinitis pigmentosa 1 (autosomal dominant)	ENSG00000104237	2156		NonMitoDomain	1		NoMitoHomolog	NoHomolog		-10.6961	0.887	0		chr8	55528626	55543394
chr8	56015016	56438710	Tnon_mito	114786	497097	XKR4	XRG4|Q5GH76	XK, Kell blood group complex subunit-related family, member 4	ENSG00000206579	650		NonMitoDomain	NA		NoMitoHomolog	NoHomolog		-10.6961	0.887	0		chr8	56015016	56438710																					Vesicles (APE, Uncertain)
chr8	56651302	56685966	Tnon_mito	137695	72098	TMEM68	Q96MH6	transmembrane protein 68	ENSG00000167904	324		SharedDomain	0		NoMitoHomolog	NoHomolog		-6.2297	0.767	0		chr8	56651302	56685966																					Nucleus but not nucleoli (APE, Uncertain)
chr8	56685790	56738005	Tnon_mito	96764	116940	TGS1	NCOA6IP|PIMT|PIPMT|Q96RS0	trimethylguanosine synthase 1	ENSG00000137574	853		SharedDomain	3		NoMitoHomolog	NoHomolog		-6.2297	0.767	0		chr8	56685790	56738005																					Nucleus;Cytoplasm (APE, Supportive)
chr8	56792385	56925006	Tpossible_mito	4067	17096	LYN	JTK8|p53Lyn|p56Lyn|P07948	v-yes-1 Yamaguchi sarcoma viral related oncogene homolog	ENSG00000254087	512		SharedDomain	2		NoMitoHomolog	NoHomolog		-6.2297	0.767	0		chr8	56792385	56925006
chr8	56980738	56987140	Tnon_mito	6224	67427	RPS20	S20|P60866	ribosomal protein S20	ENSG00000008988	142		SharedDomain	NA		NoMitoHomolog	Homolog	25-50crude	-0.4595	0.36200000000000004	0		chr8	56980738	56987140	9	8	64	4000000000	50	cerebellum, kidney, liver, adipose, smallintestine, largeintestine, stomach, placenta, testis		7.1			8.2	9			7	9.3	7.7	8	8.7	8.4	Cytoplasm (APE, Supportive)
chr8	57025500	57026541	Tnon_mito	4342	17451	MOS	MSV|P00540	v-mos Moloney murine sarcoma viral oncogene homolog	ENSG00000172680	346		SharedDomain	NA		NoMitoHomolog	NoHomolog		-6.2297	0.767	0		chr8	57025500	57026541
chr8	57073467	57123859	Tnon_mito	5324	56711	PLAG1	PSA|SGPA|ZNF912|Q6DJT9	pleiomorphic adenoma gene 1	ENSG00000181690	500		NonMitoDomain	NA		NoMitoHomolog	NoHomolog		-10.6961	0.887	0		chr8	57073467	57123859
chr8	57124314	57131176	Tpossible_mito	79145	66433	CHCHD7	COX23|Q9BUK0	coiled-coil-helix-coiled-coil-helix domain containing 7	ENSG00000170791	110	5	NA	15	2.91	HomologMitoHighConf	NoHomolog	75-100ambig	15.6465	0.003	1	yeast mito homolog, induction, coexpression, MS/MS+	chr8	57124314	57131176	4	7	14	242000000	79	cerebrum, smallintestine, placenta, testis	6.8									8.3			7.4	7.2	Cell Junctions (APE, Uncertain)
chr8	57212569	57233241	Tnon_mito	195814	242285	SDR16C5	RDH#2|RDH-E2|RDHE2|Q8N3Y7	short chain dehydrogenase/reductase family 16C, member 5	ENSG00000170786	309		SharedDomain	0		NoMitoHomolog	Homolog		-3.2256	0.5780000000000001	0		chr8	57212569	57233241
chr8	57353512	57359282	Tnon_mito	5179	18619	PENK	P01210	proenkephalin	ENSG00000181195	267		NonMitoDomain	0		NoMitoHomolog	NoHomolog	0-25crude	-9.2075	0.8820000000000001	0		chr8	57353512	57359282	4	4	6	218000000	15	brainstem, kidney, adipose, testis			8.3		6.4				6.3					6.4
chr8	57870487	57906430	Tnon_mito	54928	242291	IMPAD1	GPAPP|IMP 3|IMP-3|IMPA3|Q9NX62	inositol monophosphatase domain containing 1	ENSG00000104331	359		NonMitoDomain	1		NoMitoHomolog	Homolog		-7.692	0.8389999999999999	0		chr8	57870487	57906430																					Nucleus;Golgi apparatus (APE, Supportive)
chr8	58907112	59062277	Tpossible_mito	90362	242297	FAM110B	C8orf72|Q8TC76	family with sequence similarity 110, member B	ENSG00000169122	370		NonMitoDomain	0		NoMitoHomolog	NoHomolog		-10.6961	0.887	0		chr8	58907112	59062277																					Cytoplasm;Mitochondria (APE, Supportive)
chr8	59323822	59364060	Tnon_mito	137886	68053	UBXN2B	p37|Q14CS0	UBX domain protein 2B	ENSG00000215114	331		NonMitoDomain	3		NoMitoHomolog	NoHomolog		-10.6961	0.887	0		chr8	59323822	59364060																					Nucleus but not nucleoli (APE, Uncertain)
chr8	59402736	59412720	Tnon_mito	1581	13122	CYP7A1	CP7A|CYP7|CYPVII|P22680	cytochrome P450, family 7, subfamily A, polypeptide 1	ENSG00000167910	504		SharedDomain	2		NoMitoHomolog	NoHomolog		-6.2297	0.767	0		chr8	59402736	59412720
chr8	59465727	59495419	Tnon_mito	6386	53378	SDCBP	MDA-9|ST1|SYCL|TACIP18|O00560	syndecan binding protein (syntenin)	ENSG00000137575	298		SharedDomain	1		NoMitoHomolog	NoHomolog	0-25ambig	-3.8147	0.648	0		chr8	59465727	59495419	1	3	7	17000000	12	placenta													7.2		Nucleus but not nucleoli;Cytoplasm (APE, Uncertain)
chr8	59496063	59572404	Tnon_mito	8439	18201	NSMAF	FAN|Q92636	neutral sphingomyelinase (N-SMase) activation associated factor	ENSG00000035681	948	4	SharedDomain	0		HomologMitoLowConf	NoHomolog		-1.9231	0.48600000000000004	0		chr8	59496063	59572404																					Nucleoli (APE, Uncertain)
chr8	59717976	60031767	Tnon_mito	9760	252838	TOX	TOX1|O94900	thymocyte selection-associated high mobility group box	ENSG00000198846	526		SharedDomain	1		NoMitoHomolog	NoHomolog		-6.2297	0.767	0		chr8	59717976	60031767
chr8	61101422	61193954	Tnon_mito	767	12319	CA8	CA-VIII|CALS|CAMRQ3|CARP|P35219	carbonic anhydrase VIII	ENSG00000178538	290		SharedDomain	0		NoMitoHomolog	NoHomolog		-6.2297	0.767	0		chr8	61101422	61193954
chr8	61429468	61536203	Tnon_mito	5862	59021	RAB2A	LHX|RAB2|P61019	RAB2A, member RAS oncogene family	ENSG00000104388	212		SharedDomain	2		HomologMitoLowConf	NoHomolog	75-100crude	-0.9701000000000001	0.41800000000000004	0		chr8	61429468	61536203	13	21	256	6910000000	80	cerebrum, cerebellum, brainstem, spinalcord, kidney, liver, heart, skeletalmuscle, adipose, largeintestine, stomach, placenta, testis	9	9	9.3	8.9	8.3	7.8	7.8	7.7	8.2		8.7	7.7	8.7	8.8
chr8	61591323	61780586	Tnon_mito	55636	320790	CHD7	CRG|HH5|IS3|KAL5|Q9P2D1	chromodomain helicase DNA binding protein 7	ENSG00000171316	2997		SharedDomain	0		NoMitoHomolog	NoHomolog	0-25ambig	-3.8147	0.648	0		chr8	61591323	61780586	2	5	5	38600000	2	cerebellum, adipose		7.3							7.3						Nucleus but not nucleoli (APE, Supportive)
chr8	62200524	62414204	Tnon_mito	157807	74438	CLVS1	CRALBPL|RLBP1L1|Q8IUQ0	clavesin 1	ENSG00000177182	354		SharedDomain	0	-0.29000000000000004	NoMitoHomolog	NoHomolog		-5.5448	0.7390000000000001	0		chr8	62200524	62414204
chr8	62413114	62627199	Tnon_mito	444	65973	ASPH	AAH|BAH|CASQ2BP1|HAAH|JCTN|junctin|Q12797	aspartate beta-hydroxylase	ENSG00000198363	758	5	SharedDomain	0		NoMitoHomolog	NoHomolog	0-25crude	-2.7122	0.535	0		chr8	62413114	62627199	3	14	23	96200000	20	liver, skeletalmuscle, adipose						7.3		7.3	7.7						Endoplasmic reticulum (APE, Supportive)
chr8	63161500	63903628	Tnon_mito	286183	269513	NKAIN3	FAM77D	Na+/K+ transporting ATPase interacting 3	ENSG00000185942	197		NA	NA		NoMitoHomolog	NoHomolog		-8.0704	0.8449999999999999	0		chr8	63161500	63903628
chr8	63927638	63951610	Tnon_mito	8836	14590	GGH	GH|Q92820	gamma-glutamyl hydrolase (conjugase, folylpolygammaglutamyl hydrolase)	ENSG00000137563	318		SharedDomain	2		NoMitoHomolog	Ortholog	25-50ambig	4.4509	0.075	0		chr8	63927638	63951610	3	7	25	301000000	28	largeintestine, stomach, testis											8.3	7.4		7.9	Cytoplasm (APE, Uncertain)
chr8	63972047	63998612	Tnon_mito	7274	50500	TTPA	ATTP|AVED|TTP1|alphaTTP|P49638	tocopherol (alpha) transfer protein	ENSG00000137561	278		SharedDomain	1		NoMitoHomolog	NoHomolog		-6.2297	0.767	0		chr8	63972047	63998612
chr8	64081111	64125346	Tnon_mito	253943	229096	YTHDF3	Q7Z739	YTH domain family, member 3	ENSG00000185728	588		NonMitoDomain	1		NoMitoHomolog	NoHomolog		-10.6961	0.887	0		chr8	64081111	64125346																					Nucleus (APE, Uncertain)
chr8	65492794	65496191	Tnon_mito	27319	59058	BHLHE22	BHLHB5|Beta3|CAGL85|TNRC20|Q8NFJ8	basic helix-loop-helix family, member e22	ENSG00000180828	381		SharedDomain	0		NoMitoHomolog	NoHomolog		-6.2297	0.767	0		chr8	65492794	65496191
chr8	65508528	65711348	Tnon_mito	9420	13123	CYP7B1	CBAS3|CP7B|SPG5A|O75881	cytochrome P450, family 7, subfamily B, polypeptide 1	ENSG00000172817	506		SharedDomain	8		NoMitoHomolog	NoHomolog	0-25ambig	-0.9291	0.41100000000000003	0		chr8	65508528	65711348	1	3	5	15400000	8	liver						7.2
chr8	66514690	66546452	Tpossible_mito	55156	74252	ARMC1	Arcp|Q9NVT9	armadillo repeat containing 1	ENSG00000104442	282	5	NonMitoDomain	1		NoMitoHomolog	NoHomolog	25-50pure	-0.8212	0.405	0		chr8	66514690	66546452	6	7	48	798000000	43	cerebrum, cerebellum, brainstem, spinalcord, placenta, testis	8.2	8.1	8.1	7.9									8	8.4	Mitochondria (APE, Supportive)
chr8	66556887	66622798	Tmito	9650	67472	MTFR1	CHPPR|FAM54A2|Q15390	mitochondrial fission regulator 1	ENSG00000066855	348	3	MitoDomain	11		NoMitoHomolog	NoHomolog		6.7258	0.031000000000000003	1	literature, targetP signal, mito protein domain+, coexpression	chr8	66556887	66622798																					Mitochondria (APE, Supportive)
chr8	66626568	66753969	Tnon_mito	5150	18583	PDE7A	HCP1|PDE7|RP11-707M3.3|Q13946	phosphodiesterase 7A	ENSG00000205268	482		NonMitoDomain	0		NoMitoHomolog	NoHomolog		-10.6961	0.887	0		chr8	66626568	66753969
chr8	66933790	67012755	Tnon_mito	85479	66326	DNAJC5B	CSP-beta|Q9UF47	DnaJ (Hsp40) homolog, subfamily C, member 5 beta	ENSG00000147570	199		SharedDomain	2		NoMitoHomolog	Homolog		-3.2256	0.5780000000000001	0		chr8	66933790	67012755
chr8	67039277	67087718	Tnon_mito	84675	381485	TRIM55	MURF-2|RNF29|muRF2|Q9BYV6	tripartite motif containing 55	ENSG00000147573	548		SharedDomain	NA		NoMitoHomolog	NoHomolog		-6.2297	0.767	0		chr8	67039277	67087718
chr8	67088611	67090846	Tnon_mito	1392	12918	CRH	CRF|P06850	corticotropin releasing hormone	ENSG00000147571	196		NonMitoDomain	0		NoMitoHomolog	NoHomolog		-10.6961	0.887	0		chr8	67088611	67090846
chr8	67341262	67342968	Tnon_mito	23212	59014	RRS1	Q15050	RRS1 ribosome biogenesis regulator homolog (S. cerevisiae)	ENSG00000179041	365		NonMitoDomain	5		NoMitoHomolog	NoHomolog		-7.8105	0.8389999999999999	0		chr8	67341262	67342968																					Nucleoli (Staining, Supportive)
chr8	67344717	67381044	Tpossible_mito	137872	76187	ADHFE1	ADH8|HOT|Q8IWW8	alcohol dehydrogenase, iron containing, 1	ENSG00000147576	467	1	SharedDomain	29		OrthologMitoHighConf	NoHomolog	50-75pure	26.7588	0	1	targetP signal+, yeast mito homolog++, mito protein domain, coexpression, MS/MS++	chr8	67344717	67381044	6	19	110	6090000000	61	kidney, liver, heart, adipose, stomach, placenta					9.3	8.6	7.8		9.5			8.6	7.3		Vesicles (APE, Uncertain)
chr8	67405490	67430759	Tnon_mito	254778	76982	C8orf46	Q8TAG6	chromosome 8 open reading frame 46	ENSG00000169085	207		NA	0		NoMitoHomolog	NoHomolog		-8.0704	0.8449999999999999	0		chr8	67405490	67430759
chr8	67474409	67525480	Tnon_mito	4603	17864	MYBL1	A-MYB|AMYB|P10243	v-myb avian myeloblastosis viral oncogene homolog-like 1	ENSG00000185697	752		NonMitoDomain	0		NoMitoHomolog	NoHomolog		-10.6961	0.887	0		chr8	67474409	67525480																					Nucleus;Nucleoli;Cytoplasm (APE, Uncertain)
chr8	67542487	67579452	Tnon_mito	80124	70675	VCPIP1	DUBA3|VCIP135|Q96JH7	valosin containing protein (p97)/p47 complex interacting protein 1	ENSG00000175073	1222		NonMitoDomain	1		NoMitoHomolog	NoHomolog		-10.6961	0.887	0		chr8	67542487	67579452																					Plasma membrane (APE, Uncertain)
chr8	67579786	67774257	Tnon_mito	100533105		C8orf44-SGK3	-	C8orf44-SGK3 readthrough	ENSG00000104205	496		SharedDomain			NoMitoHomolog	NoHomolog		-8.989	0.879	0		chr8	67579786	67774257
chr8	67579786	67593377	Tnon_mito	56260		C8orf44	Q96CB5	chromosome 8 open reading frame 44	ENSG00000213865	159		NA			NoMitoHomolog	NoHomolog		-10.8297	0.921	0		chr8	67579786	67593377																					Nucleus but not nucleoli (APE, Supportive)
chr8	67624652	67774257	Tnon_mito	23678	170755	SGK3	CISK|SGK2|SGKL|Q53EW6	serum/glucocorticoid regulated kinase family, member 3	ENSG00000104205	496		SharedDomain	0		NoMitoHomolog	NoHomolog		-6.2297	0.767	0		chr8	67624652	67774257
chr8	67782983	67834283	Tnon_mito	157777	240697	MCMDC2	C8orf45|Q4G0Z9	minichromosome maintenance domain containing 2	ENSG00000178460	681		NonMitoDomain	NA		NoMitoHomolog	NoHomolog		-10.6961	0.887	0		chr8	67782983	67834283
chr8	67858735	67874825	Tnon_mito	100129654	100039596	TCF24	TCF-24|bHLHa25|Q7RTU0	transcription factor 24	ENSG00000261787	167	4	SharedDomain	NA		NoMitoHomolog	NoHomolog		-3.7952	0.639	0		chr8	67858735	67874825
chr8	67900366	67940786	Tnon_mito	286187	69312	PPP1R42	LRRC67|TLLR|dtr|Q7Z4L9	protein phosphatase 1, regulatory subunit 42	ENSG00000178125	228		SharedDomain	0		NoMitoHomolog	NoHomolog		-6.2297	0.767	0		chr8	67900366	67940786																					Nucleus but not nucleoli;Plasma membrane;Cytoplasm (APE, Uncertain)
chr8	67955314	67974562	Tnon_mito	10987	26754	COPS5	CSN5|JAB1|MOV-34|SGN5|Q92905	COP9 signalosome subunit 5	ENSG00000121022	334		NonMitoDomain	11		NoMitoHomolog	NoHomolog	0-25ambig	-3.9004	0.674	0		chr8	67955314	67974562	1	2	2	12300000	6	brainstem			7.1												Nucleus but not nucleoli (APE, Supportive)
chr8	67976602	68108849	Tnon_mito	79848	211660	CSPP1	CSPP|Q1MSJ5	centrosome and spindle pole associated protein 1	ENSG00000104218	1221		NA	0		NoMitoHomolog	NoHomolog		-8.0704	0.8449999999999999	0		chr8	67976602	68108849																					Centrosome (APE, Supportive)
chr8	68109883	68255912	Tnon_mito	10565	211673	ARFGEF1	ARFGEP1|BIG1|P200|Q9Y6D6	ADP-ribosylation factor guanine nucleotide-exchange factor 1 (brefeldin A-inhibited)	ENSG00000066777	1849		NonMitoDomain	0		NoMitoHomolog	Homolog	0-25ambig	-5.2769	0.7250000000000001	0		chr8	68109883	68255912	6	8	15	611000000	8	cerebrum, brainstem, smallintestine, largeintestine, stomach, placenta	7.9		8.2							7.3	8.1	7.8	8.2		Golgi apparatus (APE, Supportive)
chr8	68334404	68658620	Tnon_mito	57094	329093	CPA6	CPAH|ETL5|FEB11|Q8N4T0	carboxypeptidase A6	ENSG00000165078	437		SharedDomain	0		NoMitoHomolog	NoHomolog		-6.2297	0.767	0		chr8	68334404	68658620
chr8	68864602	69143897	Tnon_mito	80243	109294	PREX2	6230420N16Rik|DEP.2|DEPDC2|P-REX2|Q70Z35	phosphatidylinositol-3,4,5-trisphosphate-dependent Rac exchange factor 2	ENSG00000046889	1606		SharedDomain	0		NoMitoHomolog	NoHomolog	0-25ambig	-3.8147	0.648	0		chr8	68864602	69143897	3	2	12	469000000	3	cerebrum, brainstem, skeletalmuscle	7.8		8.6					7.2
chr8	69242956	69731258	Tnon_mito	116328	320492	C8orf34	VEST-1|VEST1|Q49A92	chromosome 8 open reading frame 34	ENSG00000165084	538	5	NA	0		NoMitoHomolog	NoHomolog		-6.0415	0.762	0		chr8	69242956	69731258
chr8	70378858	70573147	Tnon_mito	23213	240725	SULF1	HSULF-1|SULF-1|Q8IWU6	sulfatase 1	ENSG00000137573	871		NonMitoDomain	1	-0.4	NoMitoHomolog	NoHomolog		-10.0112	0.8830000000000001	0		chr8	70378858	70573147
chr8	70584567	70747299	Tnon_mito	81796	240726	SLCO5A1	OATP-J|OATP-RP4|OATP5A1|OATPRP4|SLC21A15|Q9H2Y9	solute carrier organic anion transporter family, member 5A1	ENSG00000137571	848		SharedDomain	1		NoMitoHomolog	NoHomolog		-6.2297	0.767	0		chr8	70584567	70747299
chr8	70963885	70983562	Tnon_mito	63978	383491	PRDM14	PFM11|Q9GZV8	PR domain containing 14	ENSG00000147596	571		NonMitoDomain	1		NoMitoHomolog	NoHomolog		-10.6961	0.887	0		chr8	70963885	70983562
chr8	71024266	71316020	Tnon_mito	10499	17978	NCOA2	GRIP1|KAT13C|NCoA-2|SRC2|TIF2|bHLHe75|Q15596	nuclear receptor coactivator 2	ENSG00000140396	1464		NonMitoDomain	2		NoMitoHomolog	NoHomolog	0-25ambig	-8.2811	0.8690000000000001	0		chr8	71024266	71316020	1	2	3	28500000	2	cerebrum	7.5
chr8	71485452	71520694	Tnon_mito	23471	72265	TRAM1	PNAS8|TRAM|TRAMP|Q15629	translocation associated membrane protein 1	ENSG00000067167	374	5	NonMitoDomain	3		NoMitoHomolog	NoHomolog		-8.6672	0.875	0		chr8	71485452	71520694
chr8	71549500	71581447	Tpossible_mito	51110	212442	LACTB2	Q53H82	lactamase, beta 2	ENSG00000147592	288	3	SharedDomain	NA	1.2	OrthologMitoLowConf	NoHomolog	25-50pure	15.7502	0.003	1	targetP signal, yeast mito homolog+, mito protein domain, induction, MS/MS+	chr8	71549500	71581447	4	15	80	8960000000	43	kidney, liver, largeintestine, stomach					9.9	8.8					7.8	7.4			Cytoplasm (APE, Supportive)
chr8	71581599	71648177	Tnon_mito	389668	381246	XKR9	XRG9|Q5GH70	XK, Kell blood group complex subunit-related family, member 9	ENSG00000221947	373		NonMitoDomain	NA		NoMitoHomolog	NoHomolog		-10.6961	0.887	0		chr8	71581599	71648177																					Mitochondria (APE, Uncertain)
chr8	72109667	72274467	Tnon_mito	2138	14048	EYA1	BOP|BOR|BOS1|OFC1|Q99502	eyes absent homolog 1 (Drosophila)	ENSG00000104313	592		NA	0		NoMitoHomolog	NoHomolog		-8.0704	0.8449999999999999	0		chr8	72109667	72274467
chr8	72753776	72756731	Tnon_mito	9242	17681	MSC	ABF-1|ABF1|MYOR|bHLHa22|O60682	musculin	ENSG00000178860	206		SharedDomain	0		NoMitoHomolog	NoHomolog		-6.2297	0.767	0		chr8	72753776	72756731
chr8	72933485	72987819	Tnon_mito	8989	277328	TRPA1	ANKTM1|FEPS|O75762	transient receptor potential cation channel, subfamily A, member 1	ENSG00000104321	1119		SharedDomain	0		NoMitoHomolog	Homolog		-3.2256	0.5780000000000001	0		chr8	72933485	72987819
chr8	73449625	73850584	Tnon_mito	9312	98741	KCNB2	KV2.2|Q92953	potassium voltage-gated channel, Shab-related subfamily, member 2	ENSG00000182674	911		NonMitoDomain	NA		NoMitoHomolog	NoHomolog		-10.6961	0.887	0		chr8	73449625	73850584
chr8	73921096	73959987	Tnon_mito	7013	21749	TERF1	PIN2|TRBF1|TRF|TRF1|hTRF1-AS|t-TRF1|P54274	telomeric repeat binding factor (NIMA-interacting) 1	ENSG00000147601	439		NonMitoDomain	0		NoMitoHomolog	NoHomolog		-10.6961	0.887	0		chr8	73921096	73959987																					Nucleus;Nucleoli (APE, Supportive)
chr8	73976777	74005507	Tnon_mito	157869	226866	SBSPON	C8orf84|RPESP|Q8IVN8	somatomedin B and thrombospondin, type 1 domain containing	ENSG00000164764	264		NA	0		NoMitoHomolog	NoHomolog		-8.0704	0.8449999999999999	0		chr8	73976777	74005507
chr8	74153658	74171737	Tnon_mito	100130301	75799	LOC100130301	P0DMQ9	uncharacterized LOC100130301		161		NA	1		NoMitoHomolog	NoHomolog		-8.0704	0.8449999999999999	0		chr8	74153658	74171737
chr8	74202873	74205869	Tnon_mito	6129	19989	RPL7	L7|humL7-1|P18124	ribosomal protein L7	ENSG00000147604	248		SharedDomain	NA		NoMitoHomolog	NoHomolog	50-75crude	-2.9098	0.5590000000000002	0		chr8	74202873	74205869	4	25	58	1200000000	67	liver, adipose, placenta, testis						8.8			7.7				8.1	8.6
chr8	74206836	74237520	Tnon_mito	157506	98711	RDH10	SDR16C4|Q8IZV5	retinol dehydrogenase 10 (all-trans)	ENSG00000121039	341		SharedDomain	0		NoMitoHomolog	Homolog		-3.2256	0.5780000000000001	0		chr8	74206836	74237520
chr8	74332603	74659943	Tnon_mito	27067	29819	STAU2	39K2|39K3|Q9NUL3	staufen double-stranded RNA binding protein 2	ENSG00000040341	570		SharedDomain	1		NoMitoHomolog	NoHomolog		-6.2297	0.767	0		chr8	74332603	74659943																					Nucleus but not nucleoli;Cytoplasm (APE, Uncertain)
chr8	74692331	74791145	Tnon_mito	55284	66799	UBE2W	UBC-16|UBC16|Q96B02	ubiquitin-conjugating enzyme E2W (putative)	ENSG00000104343	191	1	NonMitoDomain	1		NoMitoHomolog	NoHomolog		-3.1131	0.5710000000000001	0		chr8	74692331	74791145
chr8	74857372	74884522	Tnon_mito	6921	67923	TCEB1	SIII|eloC|Q15369	transcription elongation factor B (SIII), polypeptide 1 (15kDa, elongin C)	ENSG00000154582	112		NonMitoDomain	10		NoMitoHomolog	NoHomolog		-6.3154	0.823	0		chr8	74857372	74884522
chr8	74888376	74895018	Tmito	54968	70397	TMEM70	MC5DN2|Q9BUB7	transmembrane protein 70	ENSG00000175606	260	4	MitoDomain	46	0.33	NoMitoHomolog	NoHomolog	0-25pure	17.2263	0	1	literature, APEX_matrix, mito protein domain+, coexpression++, MS/MS	chr8	74888376	74895018	13	5	78	918000000	13	cerebrum, cerebellum, brainstem, spinalcord, liver, heart, skeletalmuscle, adipose, smallintestine, largeintestine, stomach, placenta, testis	8.1	7.7	8.3	7.6		7.6	7.6	7.6	8	7.8	8	7.6	7.5	7.7	Mitochondria (APE, Supportive)
chr8	74903563	74941307	Tnon_mito	23643	17087	LY96	ESOP-1|MD-2|MD2|ly-96|Q9Y6Y9	lymphocyte antigen 96	ENSG00000154589	160		NonMitoDomain	0		NoMitoHomolog	NoHomolog		-10.6961	0.887	0		chr8	74903563	74941307																					Vesicles (APE, Uncertain)
chr8	75146938	75233562	Tnon_mito	56704	57339	JPH1	JP-1|JP1|Q9HDC5	junctophilin 1	ENSG00000104369	661		NonMitoDomain	0		NoMitoHomolog	NoHomolog		-10.6961	0.887	0		chr8	75146938	75233562																					Nucleus (APE, Supportive)
chr8	75262617	75279335	Tmito	54332	14545	GDAP1	CMT4|CMT4A|CMTRIA|Q8TB36	ganglioside induced differentiation associated protein 1	ENSG00000104381	358		SharedDomain	1		HomologMitoLowConf	NoHomolog	50-75pure	6.3802	0.035	1	APEX_IMS, mito protein domain, MS/MS++	chr8	75262617	75279335	4	19	173	5460000000	51	cerebrum, cerebellum, brainstem, spinalcord	9.2	8.8	9.3	9.1											Cytoplasm;Mitochondria (APE, Supportive)
chr8	75736771	75767264	Tnon_mito	51050	94227	PI15	CRISP8|P24TI|P25TI|O43692	peptidase inhibitor 15	ENSG00000137558	258		NonMitoDomain	0		NoMitoHomolog	NoHomolog		-10.6961	0.887	0		chr8	75736771	75767264
chr8	75896707	75946793	Tnon_mito	83690	83691	CRISPLD1	CRISP-10|CRISP10|LCRISP1|Q9H336	cysteine-rich secretory protein LCCL domain containing 1	ENSG00000121005	500		NonMitoDomain	0		NoMitoHomolog	NoHomolog		-10.6961	0.887	0		chr8	75896707	75946793
chr8	76452202	76479061	Tnon_mito	3174	30942	HNF4G	NR2A2|NR2A3|Q14541	hepatocyte nuclear factor 4, gamma	ENSG00000164749	445		SharedDomain	0		NoMitoHomolog	NoHomolog		-6.2297	0.767	0		chr8	76452202	76479061
chr8	77593514	77779521	Tnon_mito	79776	80892	ZFHX4	ZFH4|ZHF4|Q86UP3	zinc finger homeobox 4	ENSG00000091656	3616		NonMitoDomain	2		NoMitoHomolog	NoHomolog		-10.6961	0.887	0		chr8	77593514	77779521																					Nucleus;Nucleoli (APE, Uncertain)
chr8	77892493	77913280	Tnon_mito	5828	19302	PEX2	PAF1|PBD5A|PBD5B|PMP3|PMP35|PXMP3|RNF72|ZWS3|P28328	peroxisomal biogenesis factor 2	ENSG00000164751	305		NonMitoDomain	18	0.95	NoMitoHomolog	NoHomolog		-2.5071	0.522	0		chr8	77892493	77913280
chr8	79428335	79517502	Tnon_mito	5569	18767	PKIA	PRKACN1|P61925	protein kinase (cAMP-dependent, catalytic) inhibitor alpha	ENSG00000171033	76		NA	2	-0.5	NoMitoHomolog	NoHomolog		-7.3854	0.8349999999999999	0		chr8	79428335	79517502
chr8	79578281	79631997	Tnon_mito	51101	67306	ZC2HC1A	C8orf70|FAM164A|Q96GY0	zinc finger, C2HC-type containing 1A	ENSG00000104427	325		NA	2		NoMitoHomolog	NoHomolog		-8.0704	0.8449999999999999	0		chr8	79578281	79631997																					Cytoplasm;Cytoskeleton (Actin filaments) (APE, Supportive)
chr8	79645006	79717758	Tnon_mito	3574	16196	IL7	IL-7|P13232	interleukin 7	ENSG00000104432	177		NonMitoDomain	1		NoMitoHomolog	NoHomolog		-10.6961	0.887	0		chr8	79645006	79717758
chr8	80523048	80578410	Tnon_mito	11075	20257	STMN2	SCG10|SCGN10|Q93045	stathmin-like 2	ENSG00000104435	187		NonMitoDomain	0		NoMitoHomolog	NoHomolog	25-50ambig	-5.2802	0.7250000000000001	0		chr8	80523048	80578410	6	5	25	394000000	31	cerebrum, cerebellum, brainstem, spinalcord, adipose, testis	7.5	7.4	8.4	7.5					7.6					7.5
chr8	80676244	80680098	Tnon_mito	23462	15213	HEY1	BHLHb31|CHF2|HERP2|HESR1|HRT-1|OAF1|hHRT1|Q9Y5J3	hes-related family bHLH transcription factor with YRPW motif 1	ENSG00000164683	308		SharedDomain	0		NoMitoHomolog	NoHomolog		-6.2297	0.767	0		chr8	80676244	80680098																					Nucleus (APE, Uncertain)
chr8	80831094	80942506	Tmito	28957	66230	MRPS28	MRP-S28|MRP-S35|MRPS35|Q9Y2Q9	mitochondrial ribosomal protein S28	ENSG00000147586	187	2	MitoDomain	40	0.41	NoMitoHomolog	NoHomolog	50-75pure	26.2202	0	1	literature, targetP signal, mito protein domain+, coexpression++, MS/MS++	chr8	80831094	80942506	14	14	266	4560000000	69	all 14	8.6	8.5	8.7	8.3	8.6	8.4	7.7	7.8	8.5	8.5	9	8.1	8.5	8.2	Mitochondria (APE, Supportive)
chr8	80947102	81083894	Tnon_mito	7163	21985	TPD52	D52|N8L|PC-1|PrLZ|hD52|P55327	tumor protein D52	ENSG00000076554	247		NA	1		NoMitoHomolog	NoHomolog	25-50ambig	-2.6545	0.53	0		chr8	80947102	81083894	1	5	11	18500000	26	smallintestine										7.3					Cytoplasm;Golgi apparatus (APE, Supportive)
chr8	81397853	81438500	Tnon_mito	65986	229055	ZBTB10	RINZF|Q96DT7	zinc finger and BTB domain containing 10	ENSG00000205189	871	4	NonMitoDomain	0		NoMitoHomolog	NoHomolog		-8.2616	0.8660000000000001	0		chr8	81397853	81438500																					Nucleus but not nucleoli (APE, Supportive)
chr8	81540685	81787016	Tnon_mito	619279	170753	ZNF704	Gig1|Q6ZNC4	zinc finger protein 704	ENSG00000164684	412		NA	2		NoMitoHomolog	NoHomolog		-8.0704	0.8449999999999999	0		chr8	81540685	81787016
chr8	81880045	82024303	Tnon_mito	55824	94212	PAG1	CBP|PAG|Q9NWQ8	phosphoprotein associated with glycosphingolipid microdomains 1	ENSG00000076641	432		NA	0		NoMitoHomolog	NoHomolog		-8.0704	0.8449999999999999	0		chr8	81880045	82024303
chr8	82192717	82197012	Tnon_mito	2171	16592	FABP5	E-FABP|EFABP|KFABP|PA-FABP|PAFABP|Q01469	fatty acid binding protein 5 (psoriasis-associated)	ENSG00000164687	135		SharedDomain	NA		NoMitoHomolog	NoHomolog	50-75crude	-2.9098	0.5590000000000002	0		chr8	82192717	82197012	9	13	76	785000000	74	cerebrum, cerebellum, brainstem, spinalcord, liver, adipose, stomach, placenta, testis	7.8	7.5	8	8		7.2			8.3			7.7	8.2	7.9	Plasma membrane;Cytoplasm (APE, Supportive)
chr8	82352563	82359719	Tnon_mito	5375	18857	PMP2	FABP8|M-FABP|MP2|P2|P02689	peripheral myelin protein 2	ENSG00000147588	132		SharedDomain	0		NoMitoHomolog	NoHomolog		-6.2297	0.767	0		chr8	82352563	82359719
chr8	82370617	82373758	Tnon_mito	646480	21884	FABP9	PERF|PERF15|T-FABP|Q0Z7S8	fatty acid binding protein 9, testis	ENSG00000205186	132		SharedDomain	1		NoMitoHomolog	NoHomolog	50-75crude	-2.9098	0.5590000000000002	0		chr8	82370617	82373758	3	9	30	1500000000	70	adipose, placenta, testis									8.7				7.6	9
chr8	82390731	82395473	Tnon_mito	2167	11770	FABP4	A-FABP|AFABP|ALBP|HEL-S-104|aP2|P15090	fatty acid binding protein 4, adipocyte	ENSG00000170323	132		SharedDomain	11		NoMitoHomolog	NoHomolog	75-100crude	1.5385	0.217	0		chr8	82390731	82395473	5	16	134	5430000000	89	spinalcord, skeletalmuscle, adipose, placenta, testis				6.4				6.8	9.7				8.4	7.8	Cytoplasm (APE, Supportive)
chr8	82437280	82443550	Tnon_mito	646486	75497	FABP12	A6NFH5	fatty acid binding protein 12	ENSG00000197416	140		SharedDomain	0		NoMitoHomolog	NoHomolog		-6.2297	0.767	0		chr8	82437280	82443550
chr8	82569150	82598589	Tpossible_mito	3612	55980	IMPA1	IMP|IMPA|P29218	inositol(myo)-1(or 4)-monophosphatase 1	ENSG00000133731	336	4	NonMitoDomain	5		NoMitoHomolog	Homolog	25-50ambig	3.044	0.131	0		chr8	82569150	82598589	1	10	17	1800000000	44	testis														9.3	Mitochondria (APE, Supportive)
chr8	82605890	82607207	Tnon_mito	347051	241877	SLC10A5	P5|Q5PT55	solute carrier family 10, member 5	ENSG00000253598	438		NonMitoDomain	NA		NoMitoHomolog	NoHomolog		-10.6961	0.887	0		chr8	82605890	82607207																					Plasma membrane (APE, Uncertain)
chr8	82613565	82633539	Tnon_mito	79752	66361	ZFAND1	Q8TCF1	zinc finger, AN1-type domain 1	ENSG00000104231	268		NonMitoDomain	NA		NoMitoHomolog	NoHomolog		-10.6961	0.887	0		chr8	82613565	82633539																					Cytoplasm;Centrosome (APE, Uncertain)
chr8	82644687	82671748	Tnon_mito	92421	66371	CHMP4C	SNF7-3|Shax3|VPS32C|Q96CF2	charged multivesicular body protein 4C	ENSG00000164695	233	4	NonMitoDomain	0		NoMitoHomolog	NoHomolog		-8.2616	0.8660000000000001	0		chr8	82644687	82671748
chr8	82711817	82754521	Tnon_mito	64089	74718	SNX16	P57768	sorting nexin 16	ENSG00000104497	344		NonMitoDomain	0		NoMitoHomolog	NoHomolog		-10.6961	0.887	0		chr8	82711817	82754521																					Nucleus;Vesicles (APE, Uncertain)
chr8	85095452	85834078	Tnon_mito	138046	76897	RALYL	HNRPCL3|Q86SE5	RALY RNA binding protein-like	ENSG00000184672	304		SharedDomain	1		NoMitoHomolog	NoHomolog		-6.2297	0.767	0		chr8	85095452	85834078
chr8	86019322	86058314	Tnon_mito	85444	71710	LRRCC1	CLERC|SAP2|VFL1|Q9C099	leucine rich repeat and coiled-coil centrosomal protein 1	ENSG00000133739	1032		SharedDomain	1		NoMitoHomolog	NoHomolog	0-25crude	-4.7411	0.7120000000000001	0		chr8	86019322	86058314	3	6	10	120000000	5	cerebrum, spinalcord, adipose	6.7			6.3					8						Nucleus but not nucleoli (APE, Uncertain)
chr8	86089618	86126753	Tnon_mito	1875	13559	E2F5	E2F-5|Q15329	E2F transcription factor 5, p130-binding	ENSG00000133740	346		NonMitoDomain	13		NoMitoHomolog	NoHomolog		-6.3154	0.823	0		chr8	86089618	86126753																					Nucleus (APE, Supportive)
chr8	86126287	86132643	Tnon_mito	401466	69126	C8orf59	Q8N0T1	chromosome 8 open reading frame 59	ENSG00000176731	100		NA	NA		NoMitoHomolog	NoHomolog		-8.0704	0.8449999999999999	0		chr8	86126287	86132643
chr8	86157715	86196302	Tnon_mito	377677	71934	CA13	CAXIII|Q8N1Q1	carbonic anhydrase XIII	ENSG00000185015	262		SharedDomain	1		NoMitoHomolog	NoHomolog		-6.2297	0.767	0		chr8	86157715	86196302																					Nucleoli (APE, Uncertain)
chr8	86240457	86290342	Tnon_mito	759	12346	CA1	CA-I|CAB|Car1|HEL-S-11|P00915	carbonic anhydrase I	ENSG00000133742	261		SharedDomain	3		NoMitoHomolog	NoHomolog	25-50ambig	-0.8137999999999999	0.397	0		chr8	86240457	86290342	2	8	36	457000000	39	largeintestine, placenta											8.7		7
chr8	86351055	86361267	Tnon_mito	761	12350	CA3	CAIII|Car3|P07451	carbonic anhydrase III, muscle specific	ENSG00000164879	260		SharedDomain	7		NoMitoHomolog	NoHomolog	75-100crude	0.0434	0.322	0		chr8	86351055	86361267	3	20	105	4660000000	88	liver, skeletalmuscle, adipose						8.9		7	9.6
chr8	86376130	86393721	Tnon_mito	760	12349	CA2	CA-II|CAC|CAII|Car2|HEL-76|P00918	carbonic anhydrase II	ENSG00000104267	260		SharedDomain	4	1.02	NoMitoHomolog	NoHomolog	50-75crude	0.8984999999999999	0.256	0		chr8	86376130	86393721	8	10	77	1600000000	52	cerebrum, cerebellum, brainstem, spinalcord, adipose, largeintestine, stomach, placenta	7.6	7.2	8.1	8					6.9		7.8	8.1	9.1
chr8	87060690	87081851	Tnon_mito	85481		PSKH2	Q96QS6	protein serine kinase H2	ENSG00000147613	385		SharedDomain			NoMitoHomolog	NoHomolog		-8.989	0.879	0		chr8	87060690	87081851
chr8	87111138	87166454	Tnon_mito	245972	242341	ATP6V0D2	ATP6D2|VMA6|Q8N8Y2	ATPase, H+ transporting, lysosomal 38kDa, V0 subunit d2	ENSG00000147614	350		NonMitoDomain	0		NoMitoHomolog	NoHomolog	0-25ambig	-8.2811	0.8690000000000001	0		chr8	87111138	87166454	1	2	3	10200000	11	placenta													7
chr8	87226287	87242604	Tnon_mito	157724	74087	SLC7A13	AGT-1|AGT1|XAT2|Q8TCU3	solute carrier family 7 (anionic amino acid transporter), member 13	ENSG00000164893	470		SharedDomain	2		NoMitoHomolog	Homolog		-3.2256	0.5780000000000001	0		chr8	87226287	87242604
chr8	87354993	87480178	Tnon_mito	11059	107568	WWP1	AIP5|Tiul1|hSDRP1|Q9H0M0	WW domain containing E3 ubiquitin protein ligase 1	ENSG00000123124	922	5	SharedDomain	2		HomologMitoLowConf	NoHomolog		-2.3287	0.511	0		chr8	87354993	87480178																					Cytoplasm (APE, Uncertain)
chr8	87484577	87521009	Tmito	51115	66302	RMDN1	FAM82B|RMD-1|RMD1|Q96DB5	regulator of microtubule dynamics 1	ENSG00000176623	314	1	NA	17	2.64	NoMitoHomolog	NoHomolog	50-75ambig	15.5828	0.003	1	APEX_IMS, targetP signal+, induction, coexpression, MS/MS	chr8	87484577	87521009	14	20	221	8080000000	54	all 14	8.9	8.8	8.8	8.4	8.5	9.2	8.6	8.1	9.1	8.7	8.1	8.8	8.8	8.6	Centrosome (Staining, Supportive)
chr8	87526655	87573726	Tnon_mito	8895	70568	CPNE3	CPN3|PRO1071|O75131	copine III	ENSG00000085719	537		NonMitoDomain	0		HomologMitoLowConf	NoHomolog		-8.824	0.878	0		chr8	87526655	87573726																					Nucleus (APE, Uncertain)
chr8	87586162	87755903	Tnon_mito	54714	30952	CNGB3	ACHM1|Q9NQW8	cyclic nucleotide gated channel beta 3	ENSG00000170289	809		NonMitoDomain	1		NoMitoHomolog	NoHomolog		-10.6961	0.887	0		chr8	87586162	87755903
chr8	87878675	88394955	Tnon_mito	168975		CNBD1	Q8NA66	cyclic nucleotide binding domain containing 1	ENSG00000176571	436		NonMitoDomain			NoMitoHomolog	NoHomolog		-13.4554	0.924	0		chr8	87878675	88394955
chr8	88882970	88886296	Tnon_mito	138009		DCAF4L2	WDR21C|Q8NA75	DDB1 and CUL4 associated factor 4-like 2	ENSG00000176566	395		SharedDomain			NoMitoHomolog	NoHomolog		-8.989	0.879	0		chr8	88882970	88886296
chr8	89049459	89339717	Tnon_mito	4325	17389	MMP16	C8orf57|MMP-X2|MT-MMP2|MT-MMP3|MT3-MMP|P51512	matrix metallopeptidase 16 (membrane-inserted)	ENSG00000156103	607		NonMitoDomain	1		NoMitoHomolog	NoHomolog		-10.6961	0.887	0		chr8	89049459	89339717																					Cytoplasm (APE, Uncertain)
chr8	90769974	90803292	Tnon_mito	8767	192656	RIPK2	CARD3|CARDIAK|CCK|GIG30|RICK|RIP2|O43353	receptor-interacting serine-threonine kinase 2	ENSG00000104312	540		SharedDomain	0		NoMitoHomolog	NoHomolog		-6.2297	0.767	0		chr8	90769974	90803292																					Cytoplasm (APE, Supportive)
chr8	90914095	90940095	Tnon_mito	734	209212	OSGIN2	C8orf1|hT41|Q9Y236	oxidative stress induced growth inhibitor family member 2	ENSG00000164823	549		NA	0	-0.63	NoMitoHomolog	NoHomolog		-7.3854	0.8349999999999999	0		chr8	90914095	90940095																					Nucleoli (APE, Uncertain)
chr8	90945563	90996899	Tnon_mito	4683	27354	NBN	AT-V1|AT-V2|ATV|NBS|NBS1|P95|O60934	nibrin	ENSG00000104320	754		SharedDomain	4		NoMitoHomolog	NoHomolog		-6.2297	0.767	0		chr8	90945563	90996899																					Golgi apparatus (APE, Supportive)
chr8	91013579	91064227	Tmito	1666	67460	DECR1	DECR|NADPH|SDR18C1|Q16698	2,4-dienoyl CoA reductase 1, mitochondrial	ENSG00000104325	335	2	SharedDomain	39		NoMitoHomolog	Homolog	75-100pure	20.6061	0	1	literature, APEX_matrix, targetP signal, Rickettsial homolog, mito protein domain, coexpression+, MS/MS++	chr8	91013579	91064227	14	28	1762	116000000000	84	all 14	8.8	8.5	8.9	9.4	10.1	10.2	10.3	9	10.4	10	9.9	9.4	9.9	9.7	Mitochondria (APE, Supportive)
chr8	91070837	91095107	Tnon_mito	793	12307	CALB1	CALB|P05937	calbindin 1, 28kDa	ENSG00000104327	261		SharedDomain	0		NoMitoHomolog	NoHomolog	0-25crude	-4.7411	0.7120000000000001	0		chr8	91070837	91095107	4	5	17	323000000	25	cerebrum, cerebellum, brainstem, spinalcord	7.4	7.4	8.2	8											Vesicles (APE, Supportive)
chr8	91634222	91658133	Tnon_mito	169200	100201	TMEM64	Q6YI46	transmembrane protein 64	ENSG00000180694	380		NonMitoDomain	1		NoMitoHomolog	NoHomolog		-10.6961	0.887	0		chr8	91634222	91658133
chr8	91803920	91971630	Tnon_mito	64168	69352	NECAB1	EFCBP1|STIP-1|Q8N987	N-terminal EF-hand calcium binding protein 1	ENSG00000123119	351		SharedDomain	0		NoMitoHomolog	NoHomolog		-6.2297	0.767	0		chr8	91803920	91971630																					Nucleus but not nucleoli (APE, Supportive)
chr8	91970705	91997485	Tnon_mito	100127983	100038514	C8orf88	P0DMB2	chromosome 8 open reading frame 88	ENSG00000253250	117		NA	NA		NoMitoHomolog	NoHomolog		-8.0704	0.8449999999999999	0		chr8	91970705	91997485
chr8	92006501	92053203	Tnon_mito	55529	72519	TMEM55A	Q8N4L2	transmembrane protein 55A	ENSG00000155099	257		NA	3	-0.77	NoMitoHomolog	NoHomolog		-7.3854	0.8349999999999999	0		chr8	92006501	92053203
chr8	92082423	92099323	Tnon_mito	51633	72201	OTUD6B	DUBA-5|DUBA5|Q8N6M0	OTU domain containing 6B	ENSG00000155100	323		NonMitoDomain	NA		NoMitoHomolog	NoHomolog		-10.6961	0.887	0		chr8	92082423	92099323																					Nucleus but not nucleoli;Cytoplasm;Golgi apparatus (APE, Uncertain)
chr8	92114846	92231464	Tnon_mito	100130742	73314	LRRC69	Q6ZNQ3	leucine rich repeat containing 69	ENSG00000214954	347		SharedDomain	2		HomologMitoLowConf	NoHomolog		-4.3576	0.6930000000000001	0		chr8	92114846	92231464																					Plasma membrane (APE, Uncertain)
chr8	92221721	92410382	Tnon_mito	115111	208890	SLC26A7	SUT2|Q8TE54	solute carrier family 26 (anion exchanger), member 7	ENSG00000147606	663		NonMitoDomain	0		NoMitoHomolog	NoHomolog		-10.6961	0.887	0		chr8	92221721	92410382
chr8	92967194	93115454	Tnon_mito	862	12395	RUNX1T1	AML1T1|CBFA2T1|CDR|ETO|MTG8|ZMYND2|Q06455	runt-related transcription factor 1; translocated to, 1 (cyclin D-related)	ENSG00000079102	663		NonMitoDomain	1		NoMitoHomolog	NoHomolog		-10.6961	0.887	0		chr8	92967194	93115454																					Nucleus but not nucleoli (APE, Supportive)
chr8	93895757	93978372	Tnon_mito	286144	208820	TRIQK	C8orf83|PRO0845|UPF0599|Q629K1	triple QxxK/R motif containing	ENSG00000205133	86		NonMitoDomain	2		NoMitoHomolog	NoHomolog		-10.6961	0.887	0		chr8	93895757	93978372
chr8	94146323	94179079	Tnon_mito	389676		C8orf87	E5RJ46	chromosome 8 open reading frame 87	ENSG00000254318	101	5	NA			NoMitoHomolog	NoHomolog		-8.8008	0.878	0		chr8	94146323	94179079
chr8	94712734	94741478	Tnon_mito	137392	68099	FAM92A1	A1XBS5	family with sequence similarity 92, member A1	ENSG00000188343	289	5	NA	3		NoMitoHomolog	NoHomolog		-6.0415	0.762	0		chr8	94712734	94741478																					Mitochondria (APE, Supportive)
chr8	94743730	94753224	Tnon_mito	389677	72397	RBM12B	MGC:33837|Q8IXT5	RNA binding motif protein 12B	ENSG00000183808	1001		SharedDomain	0		NoMitoHomolog	NoHomolog		-6.2297	0.767	0		chr8	94743730	94753224
chr8	94767071	94830347	Tnon_mito	91147	329795	TMEM67	JBTS6|MECKELIN|MKS3|NPHP11|TNEM67|Q5HYA8	transmembrane protein 67	ENSG00000164953	995	5	NA	1		NoMitoHomolog	NoHomolog		-6.0415	0.762	0		chr8	94767071	94830347
chr8	94929082	94938296	Tmito	54704	381511	PDP1	PDH|PDP|PDPC|PPM2C|Q9P0J1	pyruvate dehyrogenase phosphatase catalytic subunit 1	ENSG00000164951	562	2	SharedDomain	2		OrthologMitoLowConf	NoHomolog	50-75pure	16.711	0	1	literature, APEX_matrix, targetP signal, yeast mito homolog+, mito protein domain, MS/MS++	chr8	94929082	94938296	10	24	61	1220000000	53	cerebrum, cerebellum, spinalcord, skeletalmuscle, adipose, smallintestine, largeintestine, stomach, placenta, testis	7.4	7		7.5				9	7.7	7.1	7	6.7	6.8	8.2	Nucleus but not nucleoli (APE, Supportive)
chr8	95139393	95229531	Tnon_mito	1015	12557	CDH17	CDH16|HPT-1|HPT1|Q12864	cadherin 17, LI cadherin (liver-intestine)	ENSG00000079112	832		NonMitoDomain	1		NoMitoHomolog	NoHomolog	0-25ambig	-8.2811	0.8690000000000001	0		chr8	95139393	95229531	1	6	13	334000000	11	smallintestine										8.5					Nucleus but not nucleoli (APE, Uncertain)
chr8	95261484	95274547	Tnon_mito	2669	14579	GEM	KIR|P55040	GTP binding protein overexpressed in skeletal muscle	ENSG00000164949	296		SharedDomain	0		NoMitoHomolog	NoHomolog		-6.2297	0.767	0		chr8	95261484	95274547
chr8	95384187	95487343	Tnon_mito	25788	623474	RAD54B	RDH54|Q9Y620	RAD54 homolog B (S. cerevisiae)	ENSG00000197275	910	5	SharedDomain	2		NoMitoHomolog	NoHomolog		-4.2008	0.6820000000000002	0		chr8	95384187	95487343
chr8	95439939	95449180	Tnon_mito	100861412	100503583	FSBP	-	fibrinogen silencer binding protein	ENSG00000265817	299		NonMitoDomain	2		NoMitoHomolog	NoHomolog		-10.6961	0.887	0		chr8	95439939	95449180
chr8	95500004	95565746	Tnon_mito	25962	66185	KIAA1429	fSAP121|Q69YN4	KIAA1429	ENSG00000164944	1812		NA	0		NoMitoHomolog	NoHomolog		-8.0704	0.8449999999999999	0		chr8	95500004	95565746																					Nucleus (APE, Supportive)
chr8	95653363	95719694	Tnon_mito	54845	207920	ESRP1	RBM35A|RMB35A|Q6NXG1	epithelial splicing regulatory protein 1	ENSG00000104413	681		NA	1		NoMitoHomolog	NoHomolog		-8.0704	0.8449999999999999	0		chr8	95653363	95719694
chr8	95732102	95806076	Tnon_mito	286148	381510	DPY19L4	Q7Z388	dpy-19-like 4 (C. elegans)	ENSG00000156162	723		NA	NA		NoMitoHomolog	NoHomolog		-8.0704	0.8449999999999999	0		chr8	95732102	95806076																					Vesicles (APE, Uncertain)
chr8	95835517	95892721	Tnon_mito	55656	72656	INTS8	C8orf52|INT8|Q75QN2	integrator complex subunit 8	ENSG00000164941	995		NA	3		NoMitoHomolog	NoHomolog		-8.0704	0.8449999999999999	0		chr8	95835517	95892721																					Nucleus but not nucleoli (APE, Supportive)
chr8	95892452	95907482	Tnon_mito	9134	12448	CCNE2	CYCE2|O96020	cyclin E2	ENSG00000175305	404	4	NonMitoDomain	0		NoMitoHomolog	NoHomolog		-8.2616	0.8660000000000001	0		chr8	95892452	95907482
chr8	95938199	95961615	Tnon_mito	94241	60599	TP53INP1	SIP|TP53DINP1|TP53INP1A|TP53INP1B|Teap|p53DINP1|Q96A56	tumor protein p53 inducible nuclear protein 1	ENSG00000164938	240		NonMitoDomain	0		NoMitoHomolog	NoHomolog		-10.6961	0.887	0		chr8	95938199	95961615
chr8	96037213	96070944	Tmito	137682	76947	NDUFAF6	C8orf38|Q330K2	NADH dehydrogenase (ubiquinone) complex I, assembly factor 6	ENSG00000156170	333	3	SharedDomain	44	1.31	NoMitoHomolog	NoHomolog	25-50ambig	16.9393	0	1	literature, targetP signal, mito protein domain, induction, coexpression++, MS/MS	chr8	96037213	96070944	5	9	22	169000000	35	adipose, smallintestine, largeintestine, placenta, testis									7.2	7.8	7.2		7.3	7.7	Plasma membrane;Cytoplasm (APE, Uncertain)
chr8	96145948	96168913	Tnon_mito	79666	71801	PLEKHF2	EAPF|PHAFIN2|ZFYVE18|Q9H8W4	pleckstrin homology domain containing, family F (with FYVE domain) member 2	ENSG00000175895	249		SharedDomain	1		NoMitoHomolog	NoHomolog		-6.2297	0.767	0		chr8	96145948	96168913
chr8	96257140	96281462	Tnon_mito	157657	67157	C8orf37	CORD16|RP64|smalltalk|Q96NL8	chromosome 8 open reading frame 37	ENSG00000156172	207		NonMitoDomain	2		NoMitoHomolog	NoHomolog		-10.6961	0.887	0		chr8	96257140	96281462																					Cytoplasm (APE, Uncertain)
chr8	97154557	97173020	Tnon_mito	392255	242316	GDF6	BMP13|CDMP2|KFM|KFS|KFS1|KFSL|LCA17|MCOP4|MCOPCB6|SCDO4|SGM1|Q6KF10	growth differentiation factor 6	ENSG00000156466	455		NonMitoDomain	NA		NoMitoHomolog	NoHomolog		-10.6961	0.887	0		chr8	97154557	97173020
chr8	97238903	97247862	Tmito	7381	67530	UQCRB	MC3DN3|QCR7|QP-C|QPC|UQBC|UQBP|UQCR6|UQPC|P14927	ubiquinol-cytochrome c reductase binding protein	ENSG00000156467	140		MitoDomain	NA		OrthologMitoHighConf	NoHomolog	75-100pure	19.6799	0	1	literature, APEX_matrix, yeast mito homolog++, mito protein domain+, MS/MS++	chr8	97238903	97247862	14	27	1241	158000000000	97	all 14	10.1	9.9	10.3	9.6	10.4	10.1	9.9	9.5	9.9	10.2	10.1	9.9	9.8	9.9
chr8	97251625	97273841	Tmito	51001	66410	MTERFD1	mTERF3|Q96E29	MTERF domain containing 1	ENSG00000156469	417	2	MitoDomain	5		NoMitoHomolog	NoHomolog		7.1079	0.027999999999999997	1	literature, APEX_matrix, GFP, targetP signal, mito protein domain+, coexpression	chr8	97251625	97273841																					Nucleus but not nucleoli;Mitochondria (APE, Supportive)
chr8	97274166	97346774	Tnon_mito	9791	19210	PTDSS1	LMHD|PSS1|PSSA|P48651	phosphatidylserine synthase 1	ENSG00000156471	473		NonMitoDomain	0	-0.65	NoMitoHomolog	NoHomolog		-10.0112	0.8830000000000001	0		chr8	97274166	97346774																					Endoplasmic reticulum (APE, Uncertain)
chr8	97505881	97624037	Tnon_mito	6383	15529	SDC2	CD362|HSPG|HSPG1|SYND2|P34741	syndecan 2	ENSG00000169439	201		NonMitoDomain	1	-0.5700000000000001	NoMitoHomolog	NoHomolog		-10.0112	0.8830000000000001	0		chr8	97505881	97624037																					Cytoplasm (APE, Uncertain)
chr8	97657454	98155731	Tnon_mito	10404	54381	CPQ	LDP|PGCP|Q9Y646	carboxypeptidase Q	ENSG00000104324	472		NonMitoDomain	0		NoMitoHomolog	NoHomolog	25-50pure	-2.8501	0.545	0		chr8	97657454	98155731	6	15	81	4620000000	45	liver, adipose, largeintestine, stomach, placenta, testis						8			7.8		7.4	7.5	9.6	8.1	Vesicles (APE, Uncertain)
chr8	98285713	98290176	Tnon_mito	85453	239364	TSPYL5	Q86VY4	TSPY-like 5	ENSG00000180543	417		NonMitoDomain	1		NoMitoHomolog	NoHomolog		-10.6961	0.887	0		chr8	98285713	98290176																					Cytoplasm (APE, Uncertain)
chr8	98656406	98742488	Tnon_mito	92140	67154	MTDH	3D3|AEG-1|AEG1|LYRIC|LYRIC/3D3|Q86UE4	metadherin	ENSG00000147649	582		NA	1		NoMitoHomolog	NoHomolog	0-25ambig	-5.6553	0.751	0		chr8	98656406	98742488	1	4	4	33500000	7	liver						7.5									Endoplasmic reticulum (APE, Supportive)
chr8	98787808	98864830	Tnon_mito	55353	114128	LAPTM4B	LAPTM4beta|LC27|Q86VI4	lysosomal protein transmembrane 4 beta	ENSG00000104341	317	4	NonMitoDomain	0	-0.44	NoMitoHomolog	NoHomolog		-7.5767	0.8369999999999999	0		chr8	98787808	98864830																					Nucleus but not nucleoli (APE, Uncertain)
chr8	98881310	99048946	Tnon_mito	4147	17181	MATN2	O00339	matrilin 2	ENSG00000132561	956		NonMitoDomain	0		NoMitoHomolog	NoHomolog		-10.6961	0.887	0		chr8	98881310	99048946
chr8	99053937	99057818	Tnon_mito	6156	19946	RPL30	L30|P62888	ribosomal protein L30	ENSG00000156482	115		NonMitoDomain	NA		NoMitoHomolog	NoHomolog	75-100crude	-7.3086	0.8309999999999998	0		chr8	99053937	99057818	6	10	45	724000000	76	cerebellum, liver, adipose, smallintestine, placenta, testis		6.8				8.4			7	8			8.1	8.4	Cytoplasm (APE, Supportive)
chr8	99076749	99105838	Tnon_mito	203111	239368	C8orf47	Q6P6B1	chromosome 8 open reading frame 47	ENSG00000177459	374		NA	NA		NoMitoHomolog	NoHomolog		-8.0704	0.8449999999999999	0		chr8	99076749	99105838
chr8	99114566	99129418	Tmito	10247	15473	HRSP12	P14.5|PSP|UK114|P52758	heat-responsive protein 12	ENSG00000132541	137	4	SharedDomain	10		OrthologMitoHighConf	NoHomolog	75-100ambig	17.7683	0	1	APEX_matrix, yeast mito homolog++, mito protein domain, coexpression, MS/MS+	chr8	99114566	99129418	13	14	224	36100000000	96	cerebrum, cerebellum, brainstem, spinalcord, kidney, liver, heart, adipose, smallintestine, largeintestine, stomach, placenta, testis	8.1	8	8.9	8.6	10.5	9.7	7.1		8.7	7.9	8.2	8.3	8.1	7.5
chr8	99129520	99172069	Tnon_mito	10940	67724	POP1	Q99575	processing of precursor 1, ribonuclease P/MRP subunit (S. cerevisiae)	ENSG00000104356	1024		NonMitoDomain	0		NoMitoHomolog	NoHomolog	0-25ambig	-8.2811	0.8690000000000001	0		chr8	99129520	99172069	2	3	7	1190000000	5	smallintestine, largeintestine										8.8	8.7
chr8	99204386	99306621	Tnon_mito	79815	223473	NIPAL2	NPAL2|Q9H841	NIPA-like domain containing 2	ENSG00000104361	368		NA	2		NoMitoHomolog	NoHomolog		-8.0704	0.8449999999999999	0		chr8	99204386	99306621
chr8	99439249	99443023	Tnon_mito	3788	16539	KCNS2	KV9.2|Q9ULS6	potassium voltage-gated channel, delayed-rectifier, subfamily S, member 2	ENSG00000156486	477		NonMitoDomain	1		NoMitoHomolog	NoHomolog		-10.6961	0.887	0		chr8	99439249	99443023
chr8	99466858	99954799	Tnon_mito	6788	56274	STK3	KRS1|MST2|Q13188	serine/threonine kinase 3	ENSG00000104375	519		SharedDomain	1		NoMitoHomolog	Homolog		-3.2256	0.5780000000000001	0		chr8	99466858	99954799
chr8	99956630	99964332	Tnon_mito	116039	107587	OSR2	Q8N2R0	odd-skipped related transciption factor 2	ENSG00000164920	433		NonMitoDomain	0		NoMitoHomolog	NoHomolog		-10.6961	0.887	0		chr8	99956630	99964332
chr8	100025493	100889814	Tnon_mito	157680	666173	VPS13B	CHS1|COH1|Q7Z7G8	vacuolar protein sorting 13 homolog B (yeast)	ENSG00000132549	4022		NonMitoDomain	0		HomologMitoLowConf	NoHomolog		-8.824	0.878	0		chr8	100025493	100889814																					Cell Junctions (APE, Uncertain)
chr8	100890222	100906242	Tmito	1345	12864	COX6C	P09669	cytochrome c oxidase subunit VIc	ENSG00000164919	75	3	MitoDomain	47	0.85	NoMitoHomolog	NoHomolog	75-100pure	28.397	0	1	literature, APEX_IMS, APEX_matrix, targetP signal, mito protein domain+, induction, coexpression++, MS/MS++	chr8	100890222	100906242	14	18	1436	243000000000	99	all 14	10.3	10.2	10.4	9.8	10.8	10.1	10.1	9.5	9.9	10.4	10.3	10.1	9.9	10	Mitochondria (APE, Supportive)
chr8	100973165	101118344	Tnon_mito	26166	626596	RGS22	CT145|PRTD-NY2|Q8NE09	regulator of G-protein signaling 22	ENSG00000132554	1264		SharedDomain	NA		NoMitoHomolog	NoHomolog	0-25ambig	-3.8147	0.648	0		chr8	100973165	101118344	2	19	32	96200000	18	smallintestine, testis										5.5				8
chr8	101145587	101158099	Tnon_mito	286151	78803	FBXO43	EMI2|ERP1|FBX43|Q4G163	F-box protein 43	ENSG00000156509	708		SharedDomain	NA		NoMitoHomolog	NoHomolog	0-25ambig	-3.8147	0.648	0		chr8	101145587	101158099	1	3	4	76900000	4	brainstem			7.9												Nucleus;Cytoplasm (APE, Supportive)
chr8	101162838	101166230	Tnon_mito	5440	17749	POLR2K	ABC10-alpha|RPABC4|RPB10alpha|RPB12|RPB7.0|hRPB7.0|hsRPB10a|P53803	polymerase (RNA) II (DNA directed) polypeptide K, 7.0kDa	ENSG00000147669	58		NonMitoDomain	NA		NoMitoHomolog	NoHomolog		-10.6961	0.887	0		chr8	101162838	101166230
chr8	101170262	101254132	Tnon_mito	6674	26942	SPAG1	CILD28|CT140|HSD-3.8|SP75|TPIS|Q07617	sperm associated antigen 1	ENSG00000104450	926		SharedDomain	0		NoMitoHomolog	NoHomolog		-6.2297	0.767	0		chr8	101170262	101254132																					Cytoplasm (APE, Uncertain)
chr8	101269286	101348446	Tnon_mito	25897	30945	RNF19A	RNF19|Q9NV58	ring finger protein 19A, RBR E3 ubiquitin protein ligase	ENSG00000034677	838		SharedDomain	0		NoMitoHomolog	NoHomolog		-6.2297	0.767	0		chr8	101269286	101348446
chr8	101521979	101572014	Tnon_mito	157567	68839	ANKRD46	ANK-S|GENX-115279|Q86W74	ankyrin repeat domain 46	ENSG00000186106	232		SharedDomain	0		NoMitoHomolog	Homolog		-3.2256	0.5780000000000001	0		chr8	101521979	101572014
chr8	101585111	101661893	Tnon_mito	169166	66696	SNX31	Q8N9S9	sorting nexin 31	ENSG00000174226	440	5	NonMitoDomain	0		NoMitoHomolog	NoHomolog		-8.6672	0.875	0		chr8	101585111	101661893
chr8	101715143	101734315	Tnon_mito	26986	18458	PABPC1	PAB1|PABP|PABP1|PABPC2|PABPL1|P11940	poly(A) binding protein, cytoplasmic 1	ENSG00000070756	636		SharedDomain	NA	-0.45	NoMitoHomolog	NoHomolog		-5.5448	0.7390000000000001	0		chr8	101715143	101734315																					Cytoplasm (APE, Supportive)
chr8	101930803	101965623	Tpossible_mito	7534	22631	YWHAZ	14-3-3-zeta|HEL-S-3|HEL4|KCIP-1|YWHAD|P63104	tyrosine 3-monooxygenase/tryptophan 5-monooxygenase activation protein, zeta	ENSG00000164924	245		NonMitoDomain	0		NoMitoHomolog	NoHomolog	75-100crude	-7.3086	0.8309999999999998	0		chr8	101930803	101965623	11	27	634	37600000000	79	cerebrum, cerebellum, brainstem, spinalcord, liver, adipose, smallintestine, largeintestine, stomach, placenta, testis	9.8	9.7	10.2	9.7		8.2			8.6	9.2	8.4	7.9	9.2	9.1
chr8	102209265	102218292	Tnon_mito	51123	68036	ZNF706	PNAS-106|PNAS-113|Q9Y5V0	zinc finger protein 706	ENSG00000120963	76		NonMitoDomain	9		NoMitoHomolog	NoHomolog		-7.8105	0.8389999999999999	0		chr8	102209265	102218292																					Plasma membrane (APE, Uncertain)
chr8	102504667	102681952	Tnon_mito	79977	252973	GRHL2	BOM|DFNA28|TFCP2L3|Q6ISB3	grainyhead-like 2 (Drosophila)	ENSG00000083307	625		NonMitoDomain	0		NoMitoHomolog	NoHomolog		-10.6961	0.887	0		chr8	102504667	102681952
chr8	102698769	103137135	Tnon_mito	83988	52589	NCALD	P61601	neurocalcin delta	ENSG00000104490	193		SharedDomain	0		NoMitoHomolog	NoHomolog	25-50ambig	-0.8137999999999999	0.397	0		chr8	102698769	103137135	4	5	20	332000000	26	cerebrum, cerebellum, brainstem, spinalcord	7.3	7.8	8.4	7.1
chr8	103216728	103251346	Tpossible_mito	50484	382985	RRM2B	MTDPS8A|MTDPS8B|P53R2|Q7LG56	ribonucleotide reductase M2 B (TP53 inducible)	ENSG00000048392	423	4	NonMitoDomain	2		NoMitoHomolog	Homolog		-5.2575	0.7250000000000001	0		chr8	103216728	103251346																					Nucleus but not nucleoli (APE, Supportive)
chr8	103264501	103424917	Tpossible_mito	51366	70790	UBR5	DD5|EDD|EDD1|HYD|O95071	ubiquitin protein ligase E3 component n-recognin 5	ENSG00000104517	2799		NonMitoDomain	1		HomologMitoLowConf	NoHomolog	0-25ambig	-6.409	0.8249999999999998	0		chr8	103264501	103424917	2	5	10	78400000	4	cerebrum, cerebellum	7.7	7.5													Nucleus but not nucleoli (APE, Supportive)
chr8	103563847	103573245	Tnon_mito	4956	18285	ODF1	CT133|HSPB10|ODF|ODF2|ODF27|ODFP|ODFPG|ODFPGA|ODFPGB|RT7|SODF|Q14990	outer dense fiber of sperm tails 1	ENSG00000155087	250		SharedDomain	0		NoMitoHomolog	NoHomolog	25-50pure	1.6163	0.21100000000000002	0		chr8	103563847	103573245	2	11	56	3970000000	38	adipose, testis									8.3					9.6
chr8	103661004	103668130	Tnon_mito	7071	21847	KLF10	EGR-alpha|EGRA|TIEG|TIEG1|Q13118	Kruppel-like factor 10	ENSG00000155090	480		NonMitoDomain	0	-0.69	NoMitoHomolog	NoHomolog		-10.0112	0.8830000000000001	0		chr8	103661004	103668130
chr8	103838535	103876397	Tnon_mito	51582	54375	AZIN1	OAZI|OAZIN|ODC1L|O14977	antizyme inhibitor 1	ENSG00000155096	448		MitoDomain	3		NoMitoHomolog	NoHomolog		-1.0247	0.433	0		chr8	103838535	103876397																					Vesicles (APE, Uncertain)
chr8	104033247	104085285	Tnon_mito	528	66335	ATP6V1C1	ATP6C|ATP6D|VATC|Vma5|P21283	ATPase, H+ transporting, lysosomal 42kDa, V1 subunit C1	ENSG00000155097	382		NonMitoDomain	1	0.53	NoMitoHomolog	NoHomolog	25-50crude	-4.1218	0.6810000000000002	0		chr8	104033247	104085285	4	16	40	418000000	40	brainstem, spinalcord, stomach, placenta			8.3	8.1								7	8		Nucleus but not nucleoli (APE, Supportive)
chr8	104152920	104242533	Tnon_mito	79870	118452	BAALC	Q8WXS3	brain and acute leukemia, cytoplasmic	ENSG00000164929	145		NonMitoDomain	0		NoMitoHomolog	NoHomolog		-10.6961	0.887	0		chr8	104152920	104242533
chr8	104310660	104345094	Tnon_mito	8323	14368	FZD6	FZ-6|FZ6|HFZ6|NDNC10|O60353	frizzled class receptor 6	ENSG00000164930	706		NonMitoDomain	0		NoMitoHomolog	NoHomolog		-10.6961	0.887	0		chr8	104310660	104345094																					Plasma membrane (APE, Uncertain)
chr8	104383742	104395232	Tnon_mito	115908	68588	CTHRC1	Q96CG8	collagen triple helix repeat containing 1	ENSG00000164932	243		NonMitoDomain	1		NoMitoHomolog	NoHomolog		-10.6961	0.887	0		chr8	104383742	104395232																					Nucleus but not nucleoli (APE, Uncertain)
chr8	104410865	104427563	Tmito	81034	69906	SLC25A32	MFT|MFTC|Q9H2D1	solute carrier family 25 (mitochondrial folate carrier), member 32	ENSG00000164933	315	3	MitoDomain	3		OrthologMitoLowConf	NoHomolog	0-25ambig	11.7161	0.007	1	literature, targetP signal, yeast mito homolog+, mito protein domain+	chr8	104410865	104427563	2	4	5	40500000	19	adipose, placenta									7.2				7.4
chr8	104426941	104455680	Tnon_mito	25879	223499	DCAF13	GM83|WDSOF1|Q9NV06	DDB1 and CUL4 associated factor 13	ENSG00000164934	597	3	SharedDomain	2	-0.53	NoMitoHomolog	NoHomolog		-2.175	0.505	0		chr8	104426941	104455680																					Nucleus;Nucleoli (APE, Uncertain)
chr8	104512975	105266656	Tnon_mito	9699	116838	RIMS2	OBOE|RAB3IP3|RIM2|Q9UQ26	regulating synaptic membrane exocytosis 2	ENSG00000176406	1349	2	SharedDomain	0		NoMitoHomolog	NoHomolog	0-25ambig	1.4323	0.222	0		chr8	104512975	105266656	2	5	6	111000000	6	brainstem, adipose			7.5						7.9
chr8	105352023	105368917	Tnon_mito	81501	75766	DCSTAMP	FIND|TM7SF4|hDC-STAMP|Q9H295	dendrocyte expressed seven transmembrane protein	ENSG00000164935	470		NA	3		NoMitoHomolog	NoHomolog		-8.0704	0.8449999999999999	0		chr8	105352023	105368917
chr8	105391651	105479277	Tnon_mito	1807	64705	DPYS	DHP|DHPase|Q14117	dihydropyrimidinase	ENSG00000147647	519		NonMitoDomain	2		NoMitoHomolog	NoHomolog		-10.6961	0.887	0		chr8	105391651	105479277
chr8	105501458	105601252	Tnon_mito	29967	239393	LRP12	ST7|Q9Y561	low density lipoprotein receptor-related protein 12	ENSG00000147650	859		NonMitoDomain	0		NoMitoHomolog	NoHomolog		-10.6961	0.887	0		chr8	105501458	105601252																					Nucleoli;Plasma membrane;Mitochondria (APE, Uncertain)
chr8	106331146	106816767	Tnon_mito	23414	22762	ZFPM2	DIH3|FOG2|ZC2HC11B|ZNF89B|hFOG-2|Q8WW38	zinc finger protein, FOG family member 2	ENSG00000169946	1151		NonMitoDomain	0		NoMitoHomolog	NoHomolog		-10.6961	0.887	0		chr8	106331146	106816767
chr8	107282405	107764921	Tmito	55074	170719	OXR1	TLDC3|Q8N573	oxidation resistance 1	ENSG00000164830	874	4	SharedDomain	1		OrthologMitoLowConf	NoHomolog	25-50ambig	8.5766	0.02	1	literature, yeast mito homolog+, mito protein domain, MS/MS	chr8	107282405	107764921	3	28	66	589000000	37	cerebrum, cerebellum, brainstem	7.5	8.2	8.6												Vesicles (APE, Supportive)
chr8	107771710	107782472	Tnon_mito	137735	223513	ABRA	STARS|Q8N0Z2	actin-binding Rho activating protein	ENSG00000174429	381		NonMitoDomain	NA	3.9	NoMitoHomolog	NoHomolog		-5.6238	0.746	0		chr8	107771710	107782472
chr8	108261709	108510254	Tnon_mito	284	11600	ANGPT1	AGP1|AGPT|ANG1|Q15389	angiopoietin 1	ENSG00000154188	498		NonMitoDomain	4		NoMitoHomolog	NoHomolog		-10.6961	0.887	0		chr8	108261709	108510254
chr8	108911543	109095913	Tnon_mito	340419	239405	RSPO2	CRISTIN2|Q6UXX9	R-spondin 2	ENSG00000147655	243		NA	2		NoMitoHomolog	NoHomolog		-8.0704	0.8449999999999999	0		chr8	108911543	109095913
chr8	109213971	109260959	Tnon_mito	3646	16341	EIF3E	EIF3-P48|EIF3S6|INT6|eIF3-p46|P60228	eukaryotic translation initiation factor 3, subunit E	ENSG00000104408	445		NonMitoDomain	2		NoMitoHomolog	NoHomolog		-10.6961	0.887	0		chr8	109213971	109260959																					Cytoplasm (APE, Supportive)
chr8	109455852	109499136	Tmito	9694	66736	EMC2	KIAA0103|TTC35|Q15006	ER membrane protein complex subunit 2	ENSG00000104412	297		SharedDomain	3	0.5700000000000001	NoMitoHomolog	NoHomolog		-2.4214	0.515	1	GFP, mito protein domain, induction	chr8	109455852	109499136																					Nucleoli;Cytoplasm (APE, Supportive)
chr8	109795345	109799770	Tnon_mito	157753	239408	TMEM74	NET36|Q96NL1	transmembrane protein 74	ENSG00000164841	305		NA	NA		NoMitoHomolog	NoHomolog		-8.0704	0.8449999999999999	0		chr8	109795345	109799770
chr8	110099652	110131812	Tnon_mito	7201	22045	TRHR	TRH-R|P34981	thyrotropin-releasing hormone receptor	ENSG00000174417	398		NonMitoDomain	0		NoMitoHomolog	NoHomolog		-10.6961	0.887	0		chr8	110099652	110131812
chr8	110253147	110346350	Tnon_mito	84955	67429	NUDCD1	CML66|OVA66|Q96RS6	NudC domain containing 1	ENSG00000120526	583		NonMitoDomain	8		NoMitoHomolog	NoHomolog		-7.8105	0.8389999999999999	0		chr8	110253147	110346350																					Nucleus (APE, Supportive)
chr8	110346551	110358189	Tnon_mito	56943	223527	ENY2	e(y)2|Q9NPA8	enhancer of yellow 2 homolog (Drosophila)	ENSG00000120533	101		NonMitoDomain	7		NoMitoHomolog	NoHomolog		-7.8105	0.8389999999999999	0		chr8	110346551	110358189																					Nucleus but not nucleoli;Mitochondria (APE, Uncertain)
chr8	110374705	110543500	Tnon_mito	93035	192190	PKHD1L1	PKHDL1|Q86WI1	polycystic kidney and hepatic disease 1 (autosomal recessive)-like 1	ENSG00000205038	4243		NonMitoDomain	5		NoMitoHomolog	NoHomolog	0-25ambig	-5.3955	0.7310000000000001	0		chr8	110374705	110543500	1	3	3	30600000	1	adipose									7.5
chr8	110551928	110578225	Tnon_mito	9166	55960	EBAG9	EB9|PDAF|O00559	estrogen receptor binding site associated, antigen, 9	ENSG00000147654	213	5	NA	4		NoMitoHomolog	NoHomolog		-6.0415	0.762	0		chr8	110551928	110578225																					Plasma membrane;Golgi apparatus (APE, Supportive)
chr8	110586404	110704020	Tnon_mito	55638	319613	SYBU	GOLSYN|OCSYN|SNPHL|Q9NX95	syntabulin (syntaxin-interacting)	ENSG00000147642	663		NA	0		NoMitoHomolog	NoHomolog		-8.0704	0.8449999999999999	0		chr8	110586404	110704020																					Vesicles (APE, Supportive)
chr8	110979232	110986959	Tnon_mito	27012	67498	KCNV1	HNKA|KCNB3|KV2.3|KV8.1|Q6PIU1	potassium channel, subfamily V, member 1	ENSG00000164794	500		NonMitoDomain	0		NoMitoHomolog	NoHomolog		-10.6961	0.887	0		chr8	110979232	110986959
chr8	113235158	114449242	Tnon_mito	114788	239420	CSMD3	Q7Z407	CUB and Sushi multiple domains 3	ENSG00000164796	3707		NonMitoDomain	1		NoMitoHomolog	NoHomolog		-10.6961	0.887	0		chr8	113235158	114449242
chr8	116420723	116681255	Tnon_mito	7227	83925	TRPS1	GC79|LGCR|Q9UHF7	trichorhinophalangeal syndrome I	ENSG00000104447	1294		NonMitoDomain	1		NoMitoHomolog	NoHomolog		-10.6961	0.887	0		chr8	116420723	116681255																					Nucleus but not nucleoli (APE, Uncertain)
chr8	117657054	117768062	Tnon_mito	8667	68135	EIF3H	EIF3S3|eIF3-gamma|eIF3-p40|O15372	eukaryotic translation initiation factor 3, subunit H	ENSG00000147677	352		NonMitoDomain	6		HomologMitoLowConf	NoHomolog		-5.9384	0.76	0		chr8	117657054	117768062																					Cytoplasm (Staining, Uncertain)
chr8	117778741	117786921	Tnon_mito	84294	78581	UTP23	C8orf53|Q9BRU9	UTP23, small subunit (SSU) processome component, homolog (yeast)	ENSG00000147679	249		SharedDomain	1		HomologMitoHighConf	NoHomolog		-2.1416	0.502	0		chr8	117778741	117786921																					Nucleoli (APE, Uncertain)
chr8	117858172	117887105	Tnon_mito	5885	19357	RAD21	CDLS4|HR21|HRAD21|MCD1|NXP1|SCC1|hHR21|O60216	RAD21 homolog (S. pombe)	ENSG00000164754	631	4	NonMitoDomain	0		HomologMitoLowConf	NoHomolog		-6.3895	0.8249999999999998	0		chr8	117858172	117887105																					Nucleus but not nucleoli (APE, Supportive)
chr8	117950463	117956239	Tnon_mito	441376	239435	AARD	C8orf85|Q4LEZ3	alanine and arginine rich domain containing protein	ENSG00000205002	155	5	NA	3		NoMitoHomolog	NoHomolog	25-50ambig	-0.6256	0.375	0		chr8	117950463	117956239	1	6	12	149000000	36	testis														8.2
chr8	117962511	118188953	Tnon_mito	169026	239436	SLC30A8	ZNT8|ZnT-8|Q8IWU4	solute carrier family 30 (zinc transporter), member 8	ENSG00000164756	369		SharedDomain	0		NoMitoHomolog	NoHomolog		-6.2297	0.767	0		chr8	117962511	118188953
chr8	118532951	118552501	Tnon_mito	90390	69790	MED30	MED30S|THRAP6|TRAP25|Q96HR3	mediator complex subunit 30	ENSG00000164758	178		NA	3		NoMitoHomolog	NoHomolog		-8.0704	0.8449999999999999	0		chr8	118532951	118552501
chr8	118811601	119124058	Tnon_mito	2131	14042	EXT1	EXT|LGCR|LGS|TRPS2|TTV|Q16394	exostosin glycosyltransferase 1	ENSG00000182197	746		NonMitoDomain	2	-0.24	NoMitoHomolog	NoHomolog		-10.0112	0.8830000000000001	0		chr8	118811601	119124058
chr8	119201694	119634184	Tnon_mito	401474	320679	SAMD12	Q8N8I0	sterile alpha motif domain containing 12	ENSG00000177570	201		SharedDomain	NA		NoMitoHomolog	NoHomolog		-6.2297	0.767	0		chr8	119201694	119634184																					Cytoplasm;Golgi apparatus (APE, Uncertain)
chr8	119935795	119964383	Tnon_mito	4982	18383	TNFRSF11B	OCIF|OPG|TR1|O00300	tumor necrosis factor receptor superfamily, member 11b	ENSG00000164761	401		NonMitoDomain	0		NoMitoHomolog	NoHomolog		-10.6961	0.887	0		chr8	119935795	119964383
chr8	120079423	120119202	Tnon_mito	10584	239447	COLEC10	CLL1|Q9Y6Z7	collectin sub-family member 10 (C-type lectin)	ENSG00000184374	277		NonMitoDomain	4		NoMitoHomolog	NoHomolog		-10.6961	0.887	0		chr8	120079423	120119202
chr8	120220609	120257914	Tnon_mito	114569	105853	MAL2	Q969L2	mal, T-cell differentiation protein 2 (gene/pseudogene)	ENSG00000147676	176		NonMitoDomain	0		NoMitoHomolog	NoHomolog		-10.6961	0.887	0		chr8	120220609	120257914
chr8	120428551	120436678	Tnon_mito	4856	18133	NOV	CCN3|IBP-9|IGFBP-9|IGFBP9|NOVh|P48745	nephroblastoma overexpressed	ENSG00000136999	357		NonMitoDomain	1		NoMitoHomolog	NoHomolog		-10.6961	0.887	0		chr8	120428551	120436678																					Vesicles (APE, Supportive)
chr8	120569316	120651106	Tnon_mito	5168	18606	ENPP2	ATX|ATX-X|AUTOTAXIN|LysoPLD|NPP2|PD-IALPHA|PDNP2|Q13822	ectonucleotide pyrophosphatase/phosphodiesterase 2	ENSG00000136960	915		SharedDomain	1	-0.81	NoMitoHomolog	NoHomolog		-5.5448	0.7390000000000001	0		chr8	120569316	120651106
chr8	120743013	120845074	Tnon_mito	6873	319944	TAF2	CIF150|MRT40|TAF2B|TAFII150|Q6P1X5	TAF2 RNA polymerase II, TATA box binding protein (TBP)-associated factor, 150kDa	ENSG00000064313	1199		SharedDomain	3		NoMitoHomolog	NoHomolog		-6.2297	0.767	0		chr8	120743013	120845074
chr8	120846180	120868170	Tnon_mito	79075	72107	DSCC1	DCC1|Q9BVC3	DNA replication and sister chromatid cohesion 1	ENSG00000136982	393		NonMitoDomain	0		NoMitoHomolog	NoHomolog		-10.6961	0.887	0		chr8	120846180	120868170																					Nucleus but not nucleoli (APE, Uncertain)
chr8	120885894	121063157	Tnon_mito	64798	97998	DEPTOR	DEP.6|DEPDC6|Q8TB45	DEP domain containing MTOR-interacting protein	ENSG00000155792	409		NonMitoDomain	25	-0.34	NoMitoHomolog	NoHomolog		-4.3795	0.7070000000000001	0		chr8	120885894	121063157																					Nucleus but not nucleoli;Mitochondria (APE, Uncertain)
chr8	121137346	121384273	Tnon_mito	7373	12818	COL14A1	UND|Q05707	collagen, type XIV, alpha 1	ENSG00000187955	1796		NonMitoDomain	0		NoMitoHomolog	NoHomolog	0-25ambig	-8.2811	0.8690000000000001	0		chr8	121137346	121384273	1	4	4	8670000	2	largeintestine											6.9				Vesicles (APE, Uncertain)
chr8	121408082	121457647	Tmito	28998	68537	MRPL13	L13|L13A|L13mt|RPL13|RPML13|Q9BYD1	mitochondrial ribosomal protein L13	ENSG00000172172	178	3	SharedDomain	17	1.14	HomologMitoHighConf	Ortholog	75-100pure	26.3504	0	1	literature, APEX_matrix, targetP signal, yeast mito homolog, Rickettsial homolog, mito protein domain, induction, coexpression, MS/MS++	chr8	121408082	121457647	14	15	262	6220000000	85	all 14	8.7	8.5	8.8	8.2	8.8	8.6	8.1	7.7	8.6	8.5	9.2	8.1	8.8	8.5
chr8	121457637	121535875	Tnon_mito	27085	105837	MTBP	MDM2BP|Q96DY7	Mdm2, transformed 3T3 cell double minute 2, p53 binding protein (mouse) binding protein, 104kDa	ENSG00000172167	904		NA	2		NoMitoHomolog	NoHomolog		-8.0704	0.8449999999999999	0		chr8	121457637	121535875																					Nucleus (APE, Uncertain)
chr8	121547984	121824309	Tnon_mito	6641	20649	SNTB1	59-DAP|A1B|BSYN2|DAPA1B|SNT2|SNT2B1|TIP-43|Q13884	syntrophin, beta 1 (dystrophin-associated protein A1, 59kDa, basic component 1)	ENSG00000172164	538	4	SharedDomain	2		NoMitoHomolog	NoHomolog		-3.7952	0.639	0		chr8	121547984	121824309
chr8	122625270	122653630	Tnon_mito	3037	15117	HAS2	Q92819	hyaluronan synthase 2	ENSG00000170961	552		NA	1		NoMitoHomolog	NoHomolog		-8.0704	0.8449999999999999	0		chr8	122625270	122653630
chr8	123793900	123986755	Tnon_mito	22882	387609	ZHX2	AFR1|RAF|RP11-44N11.3|Q9Y6X8	zinc fingers and homeoboxes 2	ENSG00000178764	837		NonMitoDomain	0		NoMitoHomolog	NoHomolog		-10.6961	0.887	0		chr8	123793900	123986755																					Nucleus but not nucleoli (APE, Supportive)
chr8	124025403	124054663	Tnon_mito	79139	67819	DERL1	DER-1|DER1|Q9BUN8	derlin 1	ENSG00000136986	251		NonMitoDomain	2		NoMitoHomolog	NoHomolog		-10.6961	0.887	0		chr8	124025403	124054663																					Endoplasmic reticulum (APE, Supportive)
chr8	124084919	124164392	Tnon_mito	93594	210544	TBC1D31	Gm85|WDR67|Q96DN5	TBC1 domain family, member 31	ENSG00000156787	1066		SharedDomain	0		NoMitoHomolog	NoHomolog		-6.2297	0.767	0		chr8	124084919	124164392																					Centrosome (APE, Uncertain)
chr8	124191286	124222319	Tnon_mito	84985	239463	FAM83A	BJ-TSA-9|Q86UY5	family with sequence similarity 83, member A	ENSG00000147689	434	5	NonMitoDomain	NA		NoMitoHomolog	NoHomolog		-8.6672	0.875	0		chr8	124191286	124222319
chr8	124232195	124253638	Tnon_mito	84933	75758	C8orf76	Q96K31	chromosome 8 open reading frame 76	ENSG00000189376	380		NA	3		NoMitoHomolog	NoHomolog		-8.0704	0.8449999999999999	0		chr8	124232195	124253638
chr8	124238428	124286727	Tnon_mito	100533106		ZHX1-C8ORF76	Q96EF9	ZHX1-C8ORF76 readthrough	ENSG00000259305	292		NA			NoMitoHomolog	NoHomolog		-10.8297	0.921	0		chr8	124238428	124286727
chr8	124260689	124286727	Tnon_mito	11244	22770	ZHX1	Q9UKY1	zinc fingers and homeoboxes 1	ENSG00000165156	873		NonMitoDomain	0		NoMitoHomolog	NoHomolog		-10.6961	0.887	0		chr8	124260689	124286727																					Nucleus but not nucleoli (APE, Supportive)
chr8	124332090	124408705	Tnon_mito	29028	70472	ATAD2	ANCCA|CT137|PRO2000|Q6PL18	ATPase family, AAA domain containing 2	ENSG00000156802	1390		SharedDomain	1		NoMitoHomolog	Homolog		-3.2256	0.5780000000000001	0		chr8	124332090	124408705																					Nucleus but not nucleoli (APE, Supportive)
chr8	124428964	124454480	Tnon_mito	55093	76773	WDYHV1	C8orf32|Q96HA8	WDYHV motif containing 1	ENSG00000156795	205		NonMitoDomain	4		NoMitoHomolog	NoHomolog		-10.6961	0.887	0		chr8	124428964	124454480																					Nucleus but not nucleoli (APE, Supportive)
chr8	124510126	124553493	Tnon_mito	114907	67731	FBXO32	Fbx32|MAFbx|Q969P5	F-box protein 32	ENSG00000156804	355		NA	0		NoMitoHomolog	NoHomolog		-8.0704	0.8449999999999999	0		chr8	124510126	124553493																					Nucleoli (APE, Supportive)
chr8	124657914	124665190	Tnon_mito	340359	268807	KLHL38	C8ORFK36|Q2WGJ6	kelch-like family member 38	ENSG00000175946	581		NonMitoDomain	NA		NoMitoHomolog	NoHomolog		-10.6961	0.887	0		chr8	124657914	124665190																					Nucleus but not nucleoli;Vesicles (APE, Uncertain)
chr8	124693033	124749647	Tnon_mito	312	69787	ANXA13	ANX13|ISA|P27216	annexin A13	ENSG00000104537	357		NonMitoDomain	0		NoMitoHomolog	NoHomolog	0-25ambig	-8.2811	0.8690000000000001	0		chr8	124693033	124749647	2	5	8	116000000	18	smallintestine, largeintestine										8.1	5.9				Plasma membrane (APE, Supportive)
chr8	124780881	124827690	Tnon_mito	157769	210998	FAM91A1	Q658Y4	family with sequence similarity 91, member A1	ENSG00000176853	838		NA	1		NoMitoHomolog	NoHomolog		-8.0704	0.8449999999999999	0		chr8	124780881	124827690																					Nucleus but not nucleoli;Cytoskeleton (Microtubules) (APE, Uncertain)
chr8	124864226	125132302	Tnon_mito	654463		FER1L6	C8ORFK23|Q2WGJ9	fer-1-like 6 (C. elegans)	ENSG00000214814	1857		NonMitoDomain			NoMitoHomolog	NoHomolog		-13.4554	0.924	0		chr8	124864226	125132302
chr8	125323158	125384940	Tmito	157378	74868	TMEM65	Q6PI78	transmembrane protein 65	ENSG00000164983	240	1	NA	10		NoMitoHomolog	NoHomolog	50-75pure	14.6312	0.004	1	literature, APEX_matrix, targetP signal+, coexpression, MS/MS++	chr8	125323158	125384940	13	14	246	13700000000	58	cerebrum, cerebellum, brainstem, spinalcord, kidney, heart, skeletalmuscle, adipose, smallintestine, largeintestine, stomach, placenta, testis	9.5	9.3	9.7	9.1	8.3		8.5	8	8.6	8.2	8.3	7.4	8.5	8.3	Nucleus but not nucleoli (APE, Uncertain)
chr8	125463047	125465266	Tnon_mito	55039	68260	TRMT12	TRM12|TYW2|Q53H54	tRNA methyltransferase 12 homolog (S. cerevisiae)	ENSG00000183665	448		SharedDomain	1		NoMitoHomolog	NoHomolog		-6.2297	0.767	0		chr8	125463047	125465266																					Nucleus but not nucleoli (APE, Supportive)
chr8	125487007	125500859	Tnon_mito	11236	75841	RNF139	HRCA1|RCA1|TRC8|Q8WU17	ring finger protein 139	ENSG00000170881	664		SharedDomain	0	0.49	NoMitoHomolog	NoHomolog		-5.5448	0.7390000000000001	0		chr8	125487007	125500859
chr8	125500734	125551329	Tnon_mito	83940	69694	TATDN1	Q6P1N9	TatD DNase domain containing 1	ENSG00000147687	297		NonMitoDomain	18		NoMitoHomolog	Homolog		-3.3113	0.613	0		chr8	125500734	125551329																					Nucleus but not nucleoli (APE, Supportive)
chr8	125551342	125562227	Tmito	4715	66218	NDUFB9	B22|CI-B22|LYRM3|UQOR22|Q9Y6M9	NADH dehydrogenase (ubiquinone) 1 beta subcomplex, 9, 22kDa	ENSG00000147684	179	3	MitoDomain	48	1.26	NoMitoHomolog	NoHomolog	75-100pure	28.397	0	1	literature, APEX_IMS, APEX_matrix, targetP signal, mito protein domain+, induction, coexpression++, MS/MS++	chr8	125551342	125562227	14	20	696	64900000000	85	all 14	9.9	9.6	9.9	9.7	9.3	9.6	9.9	9.1	9.6	9.5	9.8	9.5	9.6	9.3	Mitochondria (APE, Supportive)
chr8	125563010	125740748	Tnon_mito	9788	211401	MTSS1	MIM|MIMA|MIMB|O43312	metastasis suppressor 1	ENSG00000170873	759		NonMitoDomain	1		NoMitoHomolog	NoHomolog		-10.6961	0.887	0		chr8	125563010	125740748
chr8	125985538	125991630	Tnon_mito	137209		ZNF572	Q7Z3I7	zinc finger protein 572	ENSG00000180938	529		NonMitoDomain			NoMitoHomolog	NoHomolog		-13.4554	0.924	0		chr8	125985538	125991630
chr8	126010719	126034525	Tnon_mito	6713	20775	SQLE	Q14534	squalene epoxidase	ENSG00000104549	574		SharedDomain	2		NoMitoHomolog	NoHomolog		-6.2297	0.767	0		chr8	126010719	126034525																					Cytoplasm;Vesicles (APE, Uncertain)
chr8	126036502	126104061	Tnon_mito	9897	223593	KIAA0196	RTSC|SPG8|Q12768	KIAA0196	ENSG00000164961	1159		NonMitoDomain	2		NoMitoHomolog	NoHomolog	0-25ambig	-8.2811	0.8690000000000001	0		chr8	126036502	126104061	5	11	15	101000000	15	cerebrum, smallintestine, largeintestine, placenta, testis	7.8									7.4	6.3		6.5	7
chr8	126104082	126379367	Tnon_mito	286053	68501	NSMCE2	C8orf36|MMS21|NSE2|ZMIZ7|Q96MF7	non-SMC element 2, MMS21 homolog (S. cerevisiae)	ENSG00000156831	247	4	NonMitoDomain	3		NoMitoHomolog	NoHomolog		-8.2616	0.8660000000000001	0		chr8	126104082	126379367																					Nucleus (APE, Supportive)
chr8	126442562	126450647	Tnon_mito	10221	211770	TRIB1	C8FW|GIG-2|GIG2|SKIP1|TRB-1|TRB1|Q96RU8	tribbles pseudokinase 1	ENSG00000173334	372	2	SharedDomain	0		HomologMitoLowConf	NoHomolog		0.8893999999999999	0.257	0		chr8	126442562	126450647																					Nucleus but not nucleoli (APE, Uncertain)
chr8	127564682	127570711	Tnon_mito	157638	399603	FAM84B	BCMP101|NSE2|Q96KN1	family with sequence similarity 84, member B	ENSG00000168672	310		NonMitoDomain	2		NoMitoHomolog	NoHomolog		-10.6961	0.887	0		chr8	127564682	127570711
chr8	128427856	128429441	Tnon_mito	5462		POU5F1B	OCT4-PG1|OTF3C|OTF3P1|POU5F1P1|POU5F1P4|POU5FLC20|POU5FLC8|Q06416	POU class 5 homeobox 1B	ENSG00000212993	359		NonMitoDomain			NoMitoHomolog	NoHomolog		-13.4554	0.924	0		chr8	128427856	128429441
chr8	128748314	128753680	Tpossible_mito	4609	17869	MYC	MRTL|MYCC|bHLHe39|c-Myc|P01106	v-myc avian myelocytomatosis viral oncogene homolog	ENSG00000136997	454		SharedDomain	3		NoMitoHomolog	NoHomolog		-6.2297	0.767	0		chr8	128748314	128753680																					Nucleoli (APE, Supportive)
chr8	130760441	130799134	Tpossible_mito	56169	83492	GSDMC	MLZE|Q9BYG8	gasdermin C	ENSG00000147697	508		NonMitoDomain	0		NoMitoHomolog	NoHomolog		-10.6961	0.887	0		chr8	130760441	130799134																					Mitochondria (APE, Supportive)
chr8	130851838	130952118	Tnon_mito	51571	223601	FAM49B	L1|Q9NUQ9	family with sequence similarity 49, member B	ENSG00000153310	324		NonMitoDomain	1		NoMitoHomolog	NoHomolog	0-25crude	-9.2075	0.8820000000000001	0		chr8	130851838	130952118	2	4	7	66000000	14	brainstem, testis			7.1											7.7	Nucleus (APE, Uncertain)
chr8	131064350	131455906	Tnon_mito	50807	13196	ASAP1	AMAP1|CENTB4|DDEF1|PAG2|PAP|ZG14P|Q9ULH1	ArfGAP with SH3 domain, ankyrin repeat and PH domain 1	ENSG00000153317	1129	4	SharedDomain	0		NoMitoHomolog	NoHomolog	0-25ambig	-1.3802	0.456	0		chr8	131064350	131455906	1	3	3	12100000	4	brainstem			7.1												Plasma membrane;Cytoplasm;Centrosome (APE, Uncertain)
chr8	131792546	132052835	Tnon_mito	114	11514	ADCY8	AC8|ADCY3|HBAC1|P40145	adenylate cyclase 8 (brain)	ENSG00000155897	1251		SharedDomain	1		NoMitoHomolog	NoHomolog		-6.2297	0.767	0		chr8	131792546	132052835
chr8	132916355	133025886	Tnon_mito	23167	76740	EFR3A	Q14156	EFR3 homolog A (S. cerevisiae)	ENSG00000132294	821	5	NA	0	0.92	HomologMitoLowConf	NoHomolog		-0.36110000000000003	0.35700000000000004	0		chr8	132916355	133025886																					Cytoplasm;Vesicles (APE, Uncertain)
chr8	133036466	133071627	Tnon_mito	729330	18256	OC90	PLA2L|Q02509	otoconin 90	ENSG00000253117	477		NonMitoDomain	NA		NoMitoHomolog	NoHomolog		-10.6961	0.887	0		chr8	133036466	133071627
chr8	133073732	133117512	Tnon_mito	10086	654498	HHLA1	PLA2L|C9JL84	HERV-H LTR-associating 1	ENSG00000132297	531		NA	NA		NoMitoHomolog	NoHomolog		-8.0704	0.8449999999999999	0		chr8	133073732	133117512
chr8	133133104	133493004	Tnon_mito	3786	110862	KCNQ3	BFNC2|EBN2|KV7.3|O43525	potassium voltage-gated channel, KQT-like subfamily, member 3	ENSG00000184156	872		NonMitoDomain	1		NoMitoHomolog	NoHomolog		-10.6961	0.887	0		chr8	133133104	133493004
chr8	133584200	133687863	Tnon_mito	23639	54562	LRRC6	CILD19|LRTP|TSLRP|Q86X45	leucine rich repeat containing 6	ENSG00000129295	466		NA	2		NoMitoHomolog	NoHomolog		-8.0704	0.8449999999999999	0		chr8	133584200	133687863
chr8	133722191	133772914	Tnon_mito	137835	213068	TMEM71	Q6P5X7	transmembrane protein 71	ENSG00000165071	276	5	NA	0		NoMitoHomolog	NoHomolog		-6.0415	0.762	0		chr8	133722191	133772914
chr8	133787603	133861052	Tnon_mito	51105	239510	PHF20L1	TDRD20B|A8MW92	PHD finger protein 20-like 1	ENSG00000129292	1017		NonMitoDomain	1		NoMitoHomolog	NoHomolog		-10.6961	0.887	0		chr8	133787603	133861052																					Nucleus but not nucleoli (APE, Supportive)
chr8	133879204	134147143	Tnon_mito	7038	21819	TG	AITD3|TGN|P01266	thyroglobulin	ENSG00000042832	2768		NonMitoDomain	1		NoMitoHomolog	NoHomolog		-10.6961	0.887	0		chr8	133879204	134147143
chr8	134048972	134115310	Tnon_mito	6503	20491	SLA	SLA1|SLAP|Q13239	Src-like-adaptor	ENSG00000155926	316	2	SharedDomain	0		NoMitoHomolog	NoHomolog		-0.9827	0.42	0		chr8	134048972	134115310
chr8	134203281	134243932	Tnon_mito	8840	22402	WISP1	CCN4|WISP1c|WISP1i|WISP1tc|O95388	WNT1 inducible signaling pathway protein 1	ENSG00000104415	367		NonMitoDomain	1	-0.78	NoMitoHomolog	NoHomolog		-10.0112	0.8830000000000001	0		chr8	134203281	134243932																					Cytoplasm (APE, Supportive)
chr8	134249413	134309547	Tnon_mito	10397	17988	NDRG1	CAP43|CMT4D|DRG-1|DRG1|GC4|HMSNL|NDR1|NMSL|PROXY1|RIT42|RTP|TARG1|TDD5|Q92597	N-myc downstream regulated 1	ENSG00000104419	394		NonMitoDomain	2		NoMitoHomolog	NoHomolog		-10.6961	0.887	0		chr8	134249413	134309547																					Cytoplasm (APE, Supportive)
chr8	134467090	134584183	Tnon_mito	6482	20442	ST3GAL1	Gal-NAc6S|SIAT4A|SIATFL|ST3GalA|ST3GalA.1|ST3GalIA|ST3GalIA,1|ST3O|Q11201	ST3 beta-galactoside alpha-2,3-sialyltransferase 1	ENSG00000008513	340		NonMitoDomain	0		NoMitoHomolog	NoHomolog		-10.6961	0.887	0		chr8	134467090	134584183
chr8	135490030	135725292	Tnon_mito	57623	380993	ZFAT	AITD3|ZFAT1|ZNF406|Q9P243	zinc finger and AT hook domain containing	ENSG00000066827	1243		NonMitoDomain	1		NoMitoHomolog	NoHomolog		-10.6961	0.887	0		chr8	135490030	135725292																					Vesicles (APE, Uncertain)
chr8	136469715	136659848	Tnon_mito	10656	13992	KHDRBS3	Etle|SALP|SLM-2|SLM2|T-STAR|TSTAR|etoile|O75525	KH domain containing, RNA binding, signal transduction associated 3	ENSG00000131773	346		NA	0	1.82	NoMitoHomolog	NoHomolog		-2.998	0.5650000000000001	0		chr8	136469715	136659848																					Nucleus but not nucleoli (APE, Supportive)
chr8	139142265	139509065	Tnon_mito	51059	70363	FAM135B	C8ORFK32|Q49AJ0	family with sequence similarity 135, member B	ENSG00000147724	1406		NonMitoDomain	2		NoMitoHomolog	NoHomolog		-10.6961	0.887	0		chr8	139142265	139509065																					Nucleus but not nucleoli (APE, Supportive)
chr8	139600477	139926236	Tnon_mito	169044	69700	COL22A1	Q8NFW1	collagen, type XXII, alpha 1	ENSG00000169436	1626		NonMitoDomain	3		NoMitoHomolog	NoHomolog	0-25ambig	-8.2811	0.8690000000000001	0		chr8	139600477	139926236	1	3	3	43100000	3	brainstem			7.6
chr8	140629364	140715299	Tnon_mito	51305	223604	KCNK9	K2p9.1|KT3.2|TASK-3|TASK3|Q9NPC2	potassium channel, subfamily K, member 9	ENSG00000169427	374		NonMitoDomain	NA		NoMitoHomolog	NoHomolog		-10.6961	0.887	0		chr8	140629364	140715299
chr8	140742585	141468678	Tnon_mito	83696	76510	TRAPPC9	IBP|IKBKBBP|MRT13|NIBP|TRS120|Q96Q05	trafficking protein particle complex 9	ENSG00000167632	1246		NonMitoDomain	6		NoMitoHomolog	NoHomolog	0-25ambig	-5.3955	0.7310000000000001	0		chr8	140742585	141468678	2	3	6	22900000	10	cerebellum, brainstem		6.8	7.2												Nucleus (APE, Supportive)
chr8	141521396	141527252	Tnon_mito	54108	93696	CHRAC1	CHARC1|CHARC15|CHRAC-1|CHRAC-15|CHRAC15|YCL1|Q9NRG0	chromatin accessibility complex 1	ENSG00000104472	131		NonMitoDomain	1		NoMitoHomolog	NoHomolog		-10.6961	0.887	0		chr8	141521396	141527252																					Nucleus (APE, Uncertain)
chr8	141541263	141645646	Tnon_mito	27161	239528	AGO2	EIF2C2|Q10|Q9UKV8	argonaute RISC catalytic component 2	ENSG00000123908	859		NonMitoDomain	0		NoMitoHomolog	NoHomolog		-10.6961	0.887	0		chr8	141541263	141645646
chr8	141668480	142011412	Tnon_mito	5747	14083	PTK2	FADK|FAK|FAK1|FRNK|PPP1R71|p125FAK|pp125FAK|Q05397	protein tyrosine kinase 2	ENSG00000169398	1074		SharedDomain	0		NoMitoHomolog	NoHomolog		-6.2297	0.767	0		chr8	141668480	142011412																					Focal Adhesions (APE, Supportive)
chr8	142138719	142205900	Tnon_mito	22898	105841	DENND3	A2RUS2	DENN/MADD domain containing 3	ENSG00000105339	1198		SharedDomain	0		NoMitoHomolog	NoHomolog		-6.2297	0.767	0		chr8	142138719	142205900
chr8	142217264	142264728	Tnon_mito	57210	106068	SLC45A4	Q5BKX6	solute carrier family 45, member 4	ENSG00000022567	808	3	NA	0		NoMitoHomolog	NoHomolog		-4.7007	0.7090000000000001	0		chr8	142217264	142264728																					Plasma membrane (APE, Uncertain)
chr8	142366586	142377365	Tnon_mito	2843	239530	GPR20	Q99678	G protein-coupled receptor 20	ENSG00000204882	358	5	NonMitoDomain	0		NoMitoHomolog	NoHomolog		-8.6672	0.875	0		chr8	142366586	142377365
chr8	142431487	142442554	Tnon_mito	11156	19245	PTP4A3	PRL-3|PRL-R|PRL3|O75365	protein tyrosine phosphatase type IVA, member 3	ENSG00000184489	173	4	SharedDomain	0	-1.33	NoMitoHomolog	NoHomolog		-3.1103	0.5700000000000001	0		chr8	142431487	142442554
chr8	142443928	142517330	Tnon_mito	389690	268816	MROH5	Q6ZUA9	maestro heat-like repeat family member 5	ENSG00000226807	1318	5	NA	NA		NoMitoHomolog	NoHomolog	25-50ambig	-0.6256	0.375	0		chr8	142443928	142517330	1	11	16	160000000	38	testis														8.2
chr8	143293440	143484543	Tnon_mito	203062		TSNARE1	Q96NA8	t-SNARE domain containing 1	ENSG00000171045	513		NonMitoDomain			NoMitoHomolog	NoHomolog		-13.4554	0.924	0		chr8	143293440	143484543																					Nucleus (APE, Uncertain)
chr8	143545376	143626368	Tnon_mito	575	107831	BAI1	GDAIF|O14514	brain-specific angiogenesis inhibitor 1	ENSG00000181790	1584		NonMitoDomain	1		NoMitoHomolog	NoHomolog		-10.6961	0.887	0		chr8	143545376	143626368
chr8	143692404	143695833	Tnon_mito	23237	11838	ARC	Arg3.1|Q7LC44	activity-regulated cytoskeleton-associated protein	ENSG00000198576	396		NA	0		NoMitoHomolog	NoHomolog		-8.0704	0.8449999999999999	0		chr8	143692404	143695833
chr8	143738873	143751412	Tnon_mito	8629	16469	JRK	JH8|Q86XJ5	jerky homolog (mouse)	ENSG00000262023	568		NonMitoDomain	0		NoMitoHomolog	NoHomolog		-10.6961	0.887	0		chr8	143738873	143751412
chr8	143761873	143764145	Tnon_mito	8000	72373	PSCA	PRO232|D3DWI6	prostate stem cell antigen	ENSG00000167653	114		NonMitoDomain	0	4.48	NoMitoHomolog	NoHomolog		-5.6238	0.746	0		chr8	143761873	143764145
chr8	143781528	143785584	Tnon_mito	54742	76486	LY6K	CT97|HSJ001348|ly-6K|Q17RY6	lymphocyte antigen 6 complex, locus K	ENSG00000160886	165		NA	1		NoMitoHomolog	NoHomolog	0-25crude	-6.5817	0.8259999999999998	0		chr8	143781528	143785584	1	4	4	62400000	19	testis														7.8	Nucleus;Plasma membrane;Cytoplasm (APE, Uncertain)
chr8	143808620	143818350	Tnon_mito	51337	223626	THEM6	C8orf55|DSCD75|Q8WUY1	thioesterase superfamily member 6	ENSG00000130193	208		NA	4	1.81	NoMitoHomolog	NoHomolog	25-50ambig	2.4179	0.159	0		chr8	143808620	143818350	3	12	29	157000000	45	cerebrum, brainstem, spinalcord	6.9		8.1	7.5											Nucleus but not nucleoli;Cytoplasm (APE, Uncertain)
chr8	143822361	143823829	Tnon_mito	57152	57277	SLURP1	ANUP|ARS|ArsB|LY6LS|MDM|P55000	secreted LY6/PLAUR domain containing 1	ENSG00000126233	103		NonMitoDomain	0		NoMitoHomolog	NoHomolog		-10.6961	0.887	0		chr8	143822361	143823829
chr8	143831627	143833952	Tnon_mito	137797	68311	LYPD2	LYPDC2|UNQ430|Q6UXB3	LY6/PLAUR domain containing 2	ENSG00000197353	125		NonMitoDomain	0		NoMitoHomolog	NoHomolog		-10.6961	0.887	0		chr8	143831627	143833952
chr8	143845755	143859640	Tnon_mito	66004	23936	LYNX1	SLURP2|Q9BZG9	Ly6/neurotoxin 1	ENSG00000180155	131		NonMitoDomain	0	1.36	NoMitoHomolog	NoHomolog		-6.8878	0.8269999999999998	0		chr8	143845755	143859640
chr8	143866297	143868008	Tnon_mito	8581	17068	LY6D	E48|Ly-6D|Q14210	lymphocyte antigen 6 complex, locus D	ENSG00000167656	128		NonMitoDomain	1		NoMitoHomolog	NoHomolog		-10.6961	0.887	0		chr8	143866297	143868008
chr8	143916216	143928262	Tnon_mito	2765	15202	GML	LY6DL|Q99445	glycosylphosphatidylinositol anchored molecule like	ENSG00000104499	158		NA	1		NoMitoHomolog	NoHomolog		-8.0704	0.8449999999999999	0		chr8	143916216	143928262
chr8	143953772	143961236	Tpossible_mito	1584		CYP11B1	CPN1|CYP11B|FHI|P450C11|P15538	cytochrome P450, family 11, subfamily B, polypeptide 1	ENSG00000160882	503	3	SharedDomain			NoMitoHomolog	NoHomolog		-5.6193	0.746	0		chr8	143953772	143961236
chr8	143991974	143999259	Tmito	1585	13072	CYP11B2	ALDOS|CPN2|CYP11B|CYP11BL|CYPXIB2|P-450C18|P450C18|P450aldo|P19099	cytochrome P450, family 11, subfamily B, polypeptide 2	ENSG00000179142	503	3	SharedDomain	8		NoMitoHomolog	NoHomolog		0.0256	0.326	1	literature, targetP signal, mito protein domain, coexpression	chr8	143991974	143999259
chr8	144099901	144103827	Tnon_mito	4061	17069	LY6E	RIG-E|RIGE|SCA-2|SCA2|TSA-1|Q16553	lymphocyte antigen 6 complex, locus E	ENSG00000160932	131		NonMitoDomain	1		NoMitoHomolog	NoHomolog		-10.6961	0.887	0		chr8	144099901	144103827
chr8	144120678	144135720	Tnon_mito	286122		C8orf31	Q8N9H6	chromosome 8 open reading frame 31	ENSG00000177335	132		NA			NoMitoHomolog	NoHomolog		-10.8297	0.921	0		chr8	144120678	144135720
chr8	144239330	144242053	Tnon_mito	4062	23934	LY6H	NMLY6|O94772	lymphocyte antigen 6 complex, locus H	ENSG00000176956	161	2	NonMitoDomain	0		NoMitoHomolog	NoHomolog		-5.4491	0.7330000000000001	0		chr8	144239330	144242053
chr8	144295067	144299044	Tnon_mito	338328	68453	GPIHBP1	GPI-HBP1|Q8IV16	glycosylphosphatidylinositol anchored high density lipoprotein binding protein 1	ENSG00000182851	184		NonMitoDomain	6		NoMitoHomolog	NoHomolog		-7.8105	0.8389999999999999	0		chr8	144295067	144299044
chr8	144328990	144344875	Tnon_mito	286128	22701	ZFP41	ZNF753|zfp-41|Q8N8Y5	ZFP41 zinc finger protein	ENSG00000181638	198		NonMitoDomain	0		NoMitoHomolog	NoHomolog		-10.6961	0.887	0		chr8	144328990	144344875
chr8	144349606	144359101	Tnon_mito	2738		GLI4	HKR4|ZNF928|P10075	GLI family zinc finger 4	ENSG00000250571	376		NonMitoDomain			NoMitoHomolog	NoHomolog		-13.4554	0.924	0		chr8	144349606	144359101																					Nucleus;Cytoskeleton (Cytokinetic bridge) (APE, Uncertain)
chr8	144373558	144382120	Tnon_mito	79943		ZNF696	Q9H7X3	zinc finger protein 696	ENSG00000185730	374		NonMitoDomain			NoMitoHomolog	NoHomolog		-13.4554	0.924	0		chr8	144373558	144382120
chr8	144391496	144442147	Tmito	116447	72960	TOP1MT	Q969P6	topoisomerase (DNA) I, mitochondrial	ENSG00000184428	601	2	SharedDomain	29	-0.62	NoMitoHomolog	NoHomolog		5.3339	0.052000000000000005	1	literature, APEX_matrix, targetP signal, mito protein domain, coexpression	chr8	144391496	144442147																					Mitochondria (APE, Supportive)
chr8	144451024	144466390	Tnon_mito	114822	14787	RHPN1	ODF5|RHOPHILIN|RHPN|Q8TCX5	rhophilin, Rho GTPase binding protein 1	ENSG00000158106	670		SharedDomain	0		NoMitoHomolog	NoHomolog		-6.2297	0.767	0		chr8	144451024	144466390																					Nucleus but not nucleoli (APE, Uncertain)
chr8	144510229	144512602	Tnon_mito	389692	378435	MAFA	RIPE3b1|hMafA|Q8NHW3	v-maf avian musculoaponeurotic fibrosarcoma oncogene homolog A	ENSG00000182759	353		NonMitoDomain	NA		NoMitoHomolog	NoHomolog		-10.6961	0.887	0		chr8	144510229	144512602
chr8	144519824	144623620	Tnon_mito	23144	223642	ZC3H3	ZC3HDC3|Q8IXZ2	zinc finger CCCH-type containing 3	ENSG00000014164	948		NonMitoDomain	NA		NoMitoHomolog	NoHomolog	0-25ambig	-8.2811	0.8690000000000001	0		chr8	144519824	144623620	1	2	2	2300000	2	testis														6.4	Nucleus but not nucleoli;Golgi apparatus (APE, Uncertain)
chr8	144635556	144645231	Tnon_mito	79792	69146	GSDMD	DF5L|DFNA5L|GSDMDC1|P57764	gasdermin D	ENSG00000104518	484		NonMitoDomain	2		NoMitoHomolog	NoHomolog		-10.6961	0.887	0		chr8	144635556	144645231																					Nucleus but not nucleoli (APE, Supportive)
chr8	144648362	144654928	Tnon_mito	642475	223645	MROH6	C8orf73|A6NGR9	maestro heat-like repeat family member 6	ENSG00000204839	719	3	NA	NA		NoMitoHomolog	NoHomolog		-4.7007	0.7090000000000001	0		chr8	144648362	144654928
chr8	144656954	144660521	Tnon_mito	93100	223646	NAPRT1	PP3856|Q6XQN6	nicotinate phosphoribosyltransferase domain containing 1	ENSG00000147813	538		NonMitoDomain	4		NoMitoHomolog	NoHomolog		-10.6961	0.887	0		chr8	144656954	144660521																					Golgi apparatus (APE, Supportive)
chr8	144661866	144679845	Tnon_mito	1936	66656	EEF1D	EF-1D|EF1D|FP1047|P29692	eukaryotic translation elongation factor 1 delta (guanine nucleotide exchange protein)	ENSG00000104529	647		NonMitoDomain	1	-0.24	NoMitoHomolog	NoHomolog		-10.0112	0.8830000000000001	0		chr8	144661866	144679845																					Nucleus;Nucleoli (APE, Supportive)
chr8	144680073	144682485	Tnon_mito	84948	105734	TIGD5	Q53EQ6	tigger transposable element derived 5	ENSG00000179886	642	5	NonMitoDomain	NA		NoMitoHomolog	NoHomolog		-8.6672	0.875	0		chr8	144680073	144682485
chr8	144686082	144691784	Tnon_mito	65263	66194	PYCRL	Q53H96	pyrroline-5-carboxylate reductase-like	ENSG00000104524	286	4	SharedDomain	13		NoMitoHomolog	NoHomolog		0.5855	0.2810000000000001	0		chr8	144686082	144691784																					Nucleus but not nucleoli (APE, Uncertain)
chr8	144694787	144699732	Tnon_mito	7264	22122	TSTA3	FX|P35B|SDR4E1|Q13630	tissue specific transplantation antigen P35B	ENSG00000104522	321		SharedDomain	2	-0.23	NoMitoHomolog	NoHomolog		-5.5448	0.7390000000000001	0		chr8	144694787	144699732																					Cytoplasm (APE, Uncertain)
chr8	144718182	144735900	Tnon_mito	9831	78834	ZNF623	O75123	zinc finger protein 623	ENSG00000183309	536		NonMitoDomain	2	-0.43	NoMitoHomolog	NoHomolog		-10.0112	0.8830000000000001	0		chr8	144718182	144735900																					Nucleus (APE, Uncertain)
chr8	144766621	144777555	Tnon_mito	286075	69020	ZNF707	Q96C28	zinc finger protein 707	ENSG00000181135	371	4	NonMitoDomain	2		NoMitoHomolog	NoHomolog		-8.2616	0.8660000000000001	0		chr8	144766621	144777555																					Nucleus but not nucleoli (APE, Supportive)
chr8	144788863	144790279	Tnon_mito	100130274	223648	CCDC166	P0CW27	coiled-coil domain containing 166	ENSG00000255181	439		NA	0		NoMitoHomolog	NoHomolog		-8.0704	0.8449999999999999	0		chr8	144788863	144790279
chr8	144798506	144804633	Tnon_mito	225689	332110	MAPK15	ERK7|ERK8|Q8TD08	mitogen-activated protein kinase 15	ENSG00000181085	544	4	SharedDomain	0		NoMitoHomolog	NoHomolog		-3.7952	0.639	0		chr8	144798506	144804633																					Nucleus but not nucleoli (APE, Uncertain)
chr8	144806102	144815914	Tnon_mito	286077	105732	FAM83H	AI3|Q6ZRV2	family with sequence similarity 83, member H	ENSG00000180921	1179		NonMitoDomain	0		NoMitoHomolog	NoHomolog	0-25ambig	-8.2811	0.8690000000000001	0		chr8	144806102	144815914	1	3	3	41900000	2	cerebrum	7.6														Cytoplasm (APE, Uncertain)
chr8	144873089	144897549	Tnon_mito	23513	105782	SCRIB	CRIB1|SCRB1|SCRIB1|Vartul|Q14160	scribbled planar cell polarity protein	ENSG00000180900	1655		SharedDomain	2		HomologMitoLowConf	NoHomolog		-4.3576	0.6930000000000001	0		chr8	144873089	144897549																					Plasma membrane (APE, Supportive)
chr8	144898513	144911556	Tnon_mito	22827	67959	PUF60	FIR|RoBPI|SIAHBP1|VRJS|Q9UHX1	poly-U binding splicing factor 60KDa	ENSG00000179950	559		SharedDomain	1		NoMitoHomolog	NoHomolog		-6.2297	0.767	0		chr8	144898513	144911556																					Nucleus but not nucleoli (APE, Supportive)
chr8	144915754	144923146	Tnon_mito	340371		NRBP2	TRG16|pp9320|Q9NSY0	nuclear receptor binding protein 2	ENSG00000185189	501		SharedDomain			NoMitoHomolog	NoHomolog		-8.989	0.879	0		chr8	144915754	144923146																					Cytoplasm (APE, Uncertain)
chr8	144939491	144952632	Tnon_mito	83481	223650	EPPK1	EPIPL|EPIPL1|P58107	epiplakin 1	ENSG00000227184	5088		SharedDomain	NA		NoMitoHomolog	NoHomolog	0-25crude	-4.7411	0.7120000000000001	0		chr8	144939491	144952632	6	7	18	916000000	2	cerebellum, brainstem, skeletalmuscle, adipose, largeintestine, testis		6.7	8.2					6.9	6.7		7.7			8.8
chr8	144989314	145050913	Tnon_mito	5339	18810	PLEC	EBS1|EBSO|HD1|LGMD2Q|PCN|PLEC1|PLEC1b|PLTN|Q15149	plectin	ENSG00000178209	4684		SharedDomain	0	-0.23	NoMitoHomolog	NoHomolog	25-50crude	-2.7787	0.537	0		chr8	144989314	145050913	12	215	523	2400000000	47	cerebrum, cerebellum, brainstem, spinalcord, liver, skeletalmuscle, adipose, smallintestine, largeintestine, stomach, placenta, testis	6.4	7.7	8.6	7.1		7.3		7.9	7.9	6	9.2	7.2	7.9	8	Cytoplasm;Cytoskeleton (Intermediate filaments) (APE, Supportive)
chr8	145051319	145060635	Tnon_mito	84875	671535	PARP10	ARTD10|Q53GL7	poly (ADP-ribose) polymerase family, member 10	ENSG00000178685	1025		NonMitoDomain	0		NoMitoHomolog	NoHomolog		-10.6961	0.887	0		chr8	145051319	145060635																					Nucleus but not nucleoli (APE, Supportive)
chr8	145064225	145067583	Tnon_mito	2907	66168	GRINA	HNRGW|LFG1|NMDARA1|TMBIM3|Q7Z429	glutamate receptor, ionotropic, N-methyl D-aspartate-associated protein 1 (glutamate binding)	ENSG00000178719	371		SharedDomain	2		HomologMitoLowConf	Homolog		-1.3535	0.451	0		chr8	145064225	145067583
chr8	145086581	145102015	Tnon_mito	375686	74281	SPATC1	SPATA15|Q76KD6	spermatogenesis and centriole associated 1	ENSG00000186583	591		NonMitoDomain	2		NoMitoHomolog	NoHomolog		-10.6961	0.887	0		chr8	145086581	145102015
chr8	145106166	145115584	Tnon_mito	26873	75475	OPLAH	5-Opase|OPLA|OPLAHD|O14841	5-oxoprolinase (ATP-hydrolysing)	ENSG00000178814	1288		NonMitoDomain	24	1.53	NoMitoHomolog	NoHomolog		0.0079	0.332	0		chr8	145106166	145115584																					Nucleus but not nucleoli;Cytoplasm (APE, Uncertain)
chr8	145133521	145135551	Tnon_mito	54512	109075	EXOSC4	RRP41|RRP41A|Rrp41p|SKI6|Ski6p|hRrp41p|p12A|Q9NPD3	exosome component 4	ENSG00000178896	245		SharedDomain	6		NoMitoHomolog	Homolog		-0.33990000000000004	0.34800000000000003	0		chr8	145133521	145135551																					Cytoplasm;Cytoskeleton (Intermediate filaments) (APE, Uncertain)
chr8	145137523	145141119	Tnon_mito	8733	14731	GPAA1	GAA1|hGAA1|O43292	glycosylphosphatidylinositol anchor attachment 1	ENSG00000197858	621	4	NonMitoDomain	3		NoMitoHomolog	NoHomolog		-8.2616	0.8660000000000001	0		chr8	145137523	145141119
chr8	145149937	145152430	Tmito	1537	66445	CYC1	MC3DN6|UQCR4|P08574	cytochrome c-1	ENSG00000179091	325	2	MitoDomain	49	1.24	OrthologMitoHighConf	Ortholog	50-75pure	43.6442	0	1	literature, APEX_IMS, APEX_matrix, targetP signal, yeast mito homolog++, Rickettsial homolog, mito protein domain+, induction, coexpression++, MS/MS++	chr8	145149937	145152430	14	19	2616	205000000000	50	all 14	10.3	10.1	10.4	10.2	10.2	10	10.4	9.8	10.1	10.2	10.2	10	10	9.8	Mitochondria (APE, Supportive)
chr8	145153535	145159140	Tnon_mito	81858	106025	SHARPIN	SIPL1|Q9H0F6	SHANK-associated RH domain interactor	ENSG00000179526	387		NonMitoDomain	1		NoMitoHomolog	NoHomolog		-10.6961	0.887	0		chr8	145153535	145159140																					Nucleus;Cytoplasm (APE, Supportive)
chr8	145159304	145162515	Tnon_mito	84232	68877	MAF1	Q9H063	MAF1 homolog (S. cerevisiae)	ENSG00000179632	256		NonMitoDomain	0		NoMitoHomolog	NoHomolog		-10.6961	0.887	0		chr8	145159304	145162515																					Nucleus (APE, Supportive)
chr8	145192671	145195620	Tnon_mito	51236	59053	FAM203A	BRP16|C8orf30A|Q9BTY7	family with sequence similarity 203, member A	ENSG00000235173	390		NA	2		NoMitoHomolog	NoHomolog		-8.0704	0.8449999999999999	0		chr8	145192671	145195620
chr8	145202918	145316843	Tnon_mito	727957	223658	MROH1	HEATR7A|Q8NDA8	maestro heat-like repeat family member 1	ENSG00000179832	1641		NonMitoDomain	0	0.42	NoMitoHomolog	NoHomolog	0-25ambig	-7.5961	0.8369999999999999	0		chr8	145202918	145316843	2	3	14	203000000	3	spinalcord, skeletalmuscle				8.3				7
chr8	145321460	145323386	Tnon_mito	642658	20289	SCXB	bHLHa48|Q7RTU7	scleraxis homolog B (mouse)	ENSG00000187786	201		SharedDomain	0	-1.07	NoMitoHomolog	NoHomolog		-5.5448	0.7390000000000001	0		chr8	145321460	145323386
chr8	145321516	145323045	Tnon_mito	100129885		SCXA	SCX|bHLHa41	scleraxis homolog A (mouse)	ENSG00000187786	201		SharedDomain			NoMitoHomolog	NoHomolog		-8.989	0.879	0		chr8	145321516	145323045
chr8	145486055	145515120	Tnon_mito	23246	12181	BOP1	Q14137	block of proliferation 1	ENSG00000170727	746	5	SharedDomain	5	-0.5600000000000002	NoMitoHomolog	NoHomolog		-0.6302000000000001	0.376	0		chr8	145486055	145515120
chr8	145515269	145538385	Tnon_mito	3297	15499	HSF1	HSTF1|Q00613	heat shock transcription factor 1	ENSG00000185122	529		NonMitoDomain	6		HomologMitoLowConf	NoHomolog		-5.9384	0.76	0		chr8	145515269	145538385																					Nucleus but not nucleoli (APE, Supportive)
chr8	145538245	145550582	Tnon_mito	8694	13350	DGAT1	ARAT|ARGP1|DGAT|O75907	diacylglycerol O-acyltransferase 1	ENSG00000185000	488		NonMitoDomain	0	2.44	NoMitoHomolog	NoHomolog	0-25crude	-4.1351	0.6820000000000002	0		chr8	145538245	145550582	1	5	10	17600000	15	adipose									7.2						Nucleoli;Endoplasmic reticulum (APE, Uncertain)
chr8	145554227	145559943	Tnon_mito	83482	170729	SCRT1	SCRT|ZNF898|Q9BWW7	scratch family zinc finger 1	ENSG00000170616	348		NonMitoDomain	0		NoMitoHomolog	NoHomolog		-10.6961	0.887	0		chr8	145554227	145559943
chr8	145576885	145579269	Tnon_mito	340393		TMEM249	C8ORFK29|Q2WGJ8	transmembrane protein 249	ENSG00000214597	235	3	NA			NoMitoHomolog	NoHomolog		-7.46	0.8369999999999999	0		chr8	145576885	145579269																					Nucleus (APE, Uncertain)
chr8	145579087	145582183	Tnon_mito	26233	30840	FBXL6	FBL6|FBL6A|PP14630|Q8N531	F-box and leucine-rich repeat protein 6	ENSG00000182325	539	4	SharedDomain	2		NoMitoHomolog	NoHomolog		-3.7952	0.639	0		chr8	145579087	145582183																					Nucleus but not nucleoli (APE, Uncertain)
chr8	145582216	145584948	Tnon_mito	79581		SLC52A2	BVVLS2|D15Ertd747e|GPCR41|GPR172A|PAR1|RFT3|RFVT2|hRFT3|Q9HAB3	solute carrier family 52 (riboflavin transporter), member 2	ENSG00000185803	445		NonMitoDomain			NoMitoHomolog	NoHomolog		-13.4554	0.924	0		chr8	145582216	145584948
chr8	145597703	145618453	Tpossible_mito	203054	268822	ADCK5	Q3MIX3	aarF domain containing kinase 5	ENSG00000173137	580	2	SharedDomain	7	0.77	OrthologMitoLowConf	Homolog	0-25pure	20.2277	0	1	targetP signal, yeast mito homolog+, Rickettsial homolog, mito protein domain, induction, coexpression, MS/MS	chr8	145597703	145618453	3	5	11	22500000	9	cerebrum, largeintestine, testis	6.4										7.1			6.9	Plasma membrane;Cytoplasm (APE, Uncertain)
chr8	145618445	145634733	Tnon_mito	29894	94230	CPSF1	CPSF160|HSU37012|P/cl.18|Q10570	cleavage and polyadenylation specific factor 1, 160kDa	ENSG00000071894	1443		NonMitoDomain	2		HomologMitoHighConf	NoHomolog		-6.608	0.8259999999999998	0		chr8	145618445	145634733
chr8	145637797	145642279	Tnon_mito	55630	72027	SLC39A4	AEZ|AWMS2|ZIP4|Q6P5W5	solute carrier family 39 (zinc transporter), member 4	ENSG00000147804	647		NonMitoDomain	0		NoMitoHomolog	NoHomolog		-10.6961	0.887	0		chr8	145637797	145642279
chr8	145648983	145653946	Tnon_mito	51160	66914	VPS28	Q9UK41	vacuolar protein sorting 28 homolog (S. cerevisiae)	ENSG00000160948	233		NonMitoDomain	5		NoMitoHomolog	NoHomolog	0-25ambig	-5.3955	0.7310000000000001	0		chr8	145648983	145653946	1	2	3	1140000	14	placenta													6.1		Golgi apparatus (APE, Supportive)
chr8	145654162	145669812	Tnon_mito	4796	72749	TONSL	IKBR|NFKBIL2|Q96HA7	tonsoku-like, DNA repair protein	ENSG00000160949	1378	4	SharedDomain	3		NoMitoHomolog	Homolog		-0.7911	0.381	0		chr8	145654162	145669812																					Nucleus but not nucleoli (APE, Supportive)
chr8	145675314	145691031	Tnon_mito	50626	54151	CYHR1	CHRP|Q6ZMK1	cysteine/histidine-rich 1	ENSG00000187954	362		NA	5		NoMitoHomolog	NoHomolog		-5.1848	0.7220000000000001	0		chr8	145675314	145691031																					Nucleus but not nucleoli (APE, Supportive)
chr8	145691737	145699499	Tnon_mito	90990	16581	KIFC2	Q96AC6	kinesin family member C2	ENSG00000167702	838		SharedDomain	0		NoMitoHomolog	NoHomolog		-6.2297	0.767	0		chr8	145691737	145699499																					Nucleus but not nucleoli (APE, Supportive)
chr8	145699114	145701718	Tnon_mito	8928	14106	FOXH1	FAST-1|FAST1|O75593	forkhead box H1	ENSG00000160973	365		NonMitoDomain	0		NoMitoHomolog	NoHomolog		-10.6961	0.887	0		chr8	145699114	145701718
chr8	145722108	145727504	Tnon_mito	84988	73062	PPP1R16A	MYPT3|Q96I34	protein phosphatase 1, regulatory subunit 16A	ENSG00000160972	528		SharedDomain	2	0.54	NoMitoHomolog	Homolog		0.5827	0.28200000000000003	0		chr8	145722108	145727504																					Plasma membrane (APE, Uncertain)
chr8	145729464	145732555	Tpossible_mito	2875	76282	GPT	AAT1|ALT1|GPT1|P24298	glutamic-pyruvate transaminase (alanine aminotransferase)	ENSG00000167701	496		SharedDomain	4	0.82	NoMitoHomolog	Homolog	0-25ambig	2.9978	0.135	0		chr8	145729464	145732555	7	5	22	1060000000	8	liver, skeletalmuscle, adipose, smallintestine, stomach, placenta, testis						8.5		7.8	8.3	7.6		8.1	7.7	8.4
chr8	145734551	145736589	Tnon_mito	113655	69572	MFSD3	Q96ES6	major facilitator superfamily domain containing 3	ENSG00000167700	412		SharedDomain	14		NoMitoHomolog	Ortholog		3.4157	0.116	0		chr8	145734551	145736589
chr8	145736666	145743210	Tnon_mito	9401	79456	RECQL4	RECQ4|O94761	RecQ protein-like 4	ENSG00000160957	1208	2	SharedDomain	10		NoMitoHomolog	Homolog		6.4021	0.034	1	targetP signal, Rickettsial homolog, mito protein domain, coexpression	chr8	145736666	145743210																					Nucleus (APE, Supportive)
chr8	145743348	145750559	Tnon_mito	9684	223664	LRRC14	LRRC14A|Q15048	leucine rich repeat containing 14	ENSG00000160959	493	5	NA	4		NoMitoHomolog	NoHomolog		-6.0415	0.762	0		chr8	145743348	145750559																					Nucleus but not nucleoli (APE, Supportive)
chr8	145747760	145752416	Tnon_mito	441381	378937	LRRC24	LRRC14OS|Q50LG9	leucine rich repeat containing 24	ENSG00000254402	513		SharedDomain	0		HomologMitoLowConf	NoHomolog		-4.3576	0.6930000000000001	0		chr8	145747760	145752416
chr8	145751602	145754458	Tmito	414919	223665	C8orf82	Q6P1X6	chromosome 8 open reading frame 82	ENSG00000213563	216	2	NA	34	0.44	NoMitoHomolog	NoHomolog	75-100pure	16.4463	0	1	APEX_matrix, targetP signal, coexpression+, MS/MS++	chr8	145751602	145754458	14	13	256	6010000000	82	all 14	8.3	8.1	8.7	8.4	9	8.7	8.7	8.1	9.1	8.2	8.4	8.5	7.8	8.9	Nucleus (APE, Uncertain)
chr8	145754562	145838888	Tnon_mito	80728	223666	ARHGAP39	CrGAP|Vilse|Q9C0H5	Rho GTPase activating protein 39	ENSG00000147799	1114		SharedDomain	0		HomologMitoLowConf	NoHomolog	0-25ambig	-1.9426	0.49	0		chr8	145754562	145838888	8	3	12	1130000000	6	cerebrum, kidney, liver, heart, skeletalmuscle, adipose, stomach, placenta	8.6				8.5	8	7.9	7.8	7.9			6.6	8		Cytoplasm (Staining, Uncertain)
chr8	145946293	145980970	Tnon_mito	90987	71591	ZNF251	Q9BRH9	zinc finger protein 251	ENSG00000198169	671		NonMitoDomain	NA		NoMitoHomolog	NoHomolog		-10.6961	0.887	0		chr8	145946293	145980970																					Nucleus but not nucleoli (APE, Uncertain)
chr8	145997608	146012730	Tnon_mito	80778		ZNF34	KOX32|Q8IZ26	zinc finger protein 34	ENSG00000196378	560		NonMitoDomain			NoMitoHomolog	NoHomolog		-13.4554	0.924	0		chr8	145997608	146012730
chr8	146015153	146017805	Tnon_mito	6132	26961	RPL8	L8|P62917	ribosomal protein L8	ENSG00000161016	257	4	SharedDomain	3	-0.34	NoMitoHomolog	Homolog	25-50crude	2.6599	0.152	0		chr8	146015153	146017805	5	10	38	621000000	40	liver, adipose, smallintestine, placenta, testis						8.4			7.2	8			8.1	8.1	Nucleoli;Cytoplasm (APE, Supportive)
chr8	146024260	146034529	Tnon_mito	340385		ZNF517	Q6ZMY9	zinc finger protein 517	ENSG00000197363	492		NonMitoDomain			NoMitoHomolog	NoHomolog		-13.4554	0.924	0		chr8	146024260	146034529
chr8	146052902	146072893	Tnon_mito	7553	223669	ZNF7	HF.16|KOX4|zf30|P17097	zinc finger protein 7	ENSG00000147789	697		NonMitoDomain	1		NoMitoHomolog	NoHomolog		-10.6961	0.887	0		chr8	146052902	146072893																					Mitochondria (APE, Uncertain)
chr8	146075550	146078963	Tnon_mito	28991	66398	COMMD5	HCARG|Q9GZQ3	COMM domain containing 5	ENSG00000170619	224		NonMitoDomain	2		NoMitoHomolog	NoHomolog		-10.6961	0.887	0		chr8	146075550	146078963																					Nucleus but not nucleoli;Cytoplasm (APE, Uncertain)
chr8	146102335	146126846	Tnon_mito	58500	239546	ZNF250	ZFP647|ZNF647|P15622	zinc finger protein 250	ENSG00000196150	560		NonMitoDomain	0		NoMitoHomolog	NoHomolog		-10.6961	0.887	0		chr8	146102335	146126846																					Nucleus but not nucleoli (APE, Uncertain)
chr8	146155743	146176274	Tnon_mito	7564		ZNF16	HZF1|KOX9|P17020	zinc finger protein 16	ENSG00000170631	682		NonMitoDomain			NoMitoHomolog	NoHomolog		-13.4554	0.924	0		chr8	146155743	146176274																					Cell Junctions (APE, Uncertain)
chr8	146277823	146281416	Tnon_mito	65265	117171	C8orf33	Q9H7E9	chromosome 8 open reading frame 33	ENSG00000182307	229		NA	2		NoMitoHomolog	NoHomolog		-8.0704	0.8449999999999999	0		chr8	146277823	146281416																					Nucleus but not nucleoli;Plasma membrane (APE, Uncertain)
