# Supplementary material for: Transmembrane protein GRINA modulates aerobic glycolysis and promotes tumor progression in gastric cancer
Source: J Exp Clin Cancer Res. 2018 Dec 12;37:308. doi: 10.1186/s13046-018-0974-1 (PMC6292005; doi:10.1186/s13046-018-0974-1)
Supplement: Supplementary file 11 [file 13046_2018_974_MOESM7_ESM.docx]

## Sheet: chr8q24

Table S6. Genes differentially expressed on chromosome 8q24
	TCGA-BR-6453-01A	TCGA-BR-6454-01A	TCGA-BR-6457-01A	TCGA-BR-6458-01A	TCGA-BR-6564-01A	TCGA-BR-6802-01A	TCGA-BR-6852-01A	TCGA-BR-7704-01A	TCGA-BR-7715-01A	TCGA-BR-7716-01A	TCGA-BR-7717-01A	TCGA-BR-7851-01A	TCGA-CG-5720-01A	TCGA-CG-5721-01A	TCGA-CG-5722-01A	TCGA-CG-5734-01A	TCGA-FP-7735-01A	TCGA-FP-7829-01A	TCGA-HU-8238-01A	TCGA-HU-A4G3-01A	TCGA-HU-A4GC-01A	TCGA-HU-A4GH-01A	TCGA-HU-A4GN-01A	TCGA-HU-A4GP-01A	TCGA-HU-A4GY-01A	TCGA-HU-A4HB-01A	TCGA-IN-7806-01A	TCGA-IN-8462-01A	TCGA-IN-8663-01A	TCGA-IN-AB1V-01A	TCGA-IN-AB1X-01A	TCGA-IP-7968-01A	TCGA-BR-6453-11A	TCGA-BR-6454-11A	TCGA-BR-6457-11A	TCGA-BR-6458-11A	TCGA-BR-6564-11A	TCGA-BR-6802-11A	TCGA-BR-6852-11A	TCGA-BR-7704-11A	TCGA-BR-7715-11A	TCGA-BR-7716-11A	TCGA-BR-7717-11A	TCGA-BR-7851-11A	TCGA-CG-5720-11A	TCGA-CG-5721-11A	TCGA-CG-5722-11A	TCGA-CG-5734-11A	TCGA-FP-7735-11A
ATAD2	9.55832695417831	9.865485842125707	8.782333204161121	10.046114490696507	7.6995099900932615	10.49511550612795	9.987633522098527	10.278379761124361	10.836334446458478	10.601334386072487	9.333240343793863	10.896574598906254	9.303018109703206	10.026177596889227	10.115914988131555	8.780854681874654	9.598424760527719	10.999062789200662	9.825777678885062	9.321562866478967	10.913495320450238	10.764116880442796	10.265978311438943	11.112876028664994	10.456426291362533	9.916258671146718	9.394323348835476	9.156128594432547	11.302713169840349	9.641108848221323	10.365600686328357	11.246366517951488	8.631919134147433	8.035403969942067	7.99952090061982	8.416451323690096	8.200928022479372	8.116557100435417	8.762551464448594	8.11681178647236	8.62067804213541	8.890036225007954	9.136955236446655	8.984791545897298	7.989178753614199	8.338201539338446	8.03825508712839	7.878087707882035	7.300032180101146
DSCC1	7.586524574378245	7.141085318562331	6.806968434121849	7.493054841539428	6.196188006854777	8.029226480635012	7.7443293104648925	8.057228218697077	9.551169913872759	8.091551749333409	8.264555196421218	8.38129202587899	7.330092245316578	7.863350006133586	7.847809043407229	6.010893943184788	7.710069403609412	8.813024379347574	7.095470870008814	6.0616962856182806	8.932604537067135	7.903200998093015	7.9000214670377815	7.902363054288985	7.977159693903539	7.5154790811702545	7.082500356039423	6.179397588832459	8.232890955717586	6.172235469904423	6.858179973824653	8.434686601399612	6.0932960691635945	6.0426355831644525	5.778713221086107	5.755541909804473	5.771196278796389	6.896501944676451	6.877327083932384	5.691330364267335	6.437079559383805	6.420847846647662	7.171086704602052	6.031853906013212	5.404218524074025	6.023887391088284	5.4775413400260335	5.062670045998984	4.56160108389449
MTBP	5.719788544401166	6.6946852208358045	5.450821943885435	6.688893695073583	4.8672391963486294	7.020668871984187	7.200888799091503	6.6504917307159435	7.897040709837656	7.91988230228365	6.4737349684635985	7.228972569760161	6.18218972947669	6.589666125749582	6.525880948519866	5.114729319147784	6.41600873722311	7.544392649172546	6.6914783319874065	5.956725788726184	7.827634866049794	7.929607572565146	7.082031917445089	7.53730383351611	7.312401976769852	6.46328404031257	6.511861741103661	5.656556433926211	8.271318284247961	6.14798839836129	7.062888026459289	8.22545328883336	5.53909365566718	4.528064911552698	4.148665709007665	5.100674952677191	4.979307431167711	4.68500383040852	5.576455624880795	5.404617060970657	5.86238443761299	5.442223627030027	6.029426381654215	5.550321993430762	3.9594363311077556	4.2353900853395094	4.61504545249424	4.034101239143841	4.232292362089148
RECQL4	9.002142726212133	8.73955448546191	8.59268874687878	9.877066293902985	6.858416225388646	9.893573053585902	10.290756138396674	10.239861030518547	10.4578938373147	9.703695976489001	9.567950373052255	9.59983475606401	8.586944843853583	9.322779935111578	9.127677125269011	8.129679703539304	8.852448360402551	9.776896857536748	8.185673314285635	9.108388544321818	10.164454442645983	10.045623165328758	9.89293739783468	10.40242587625305	8.601391961599003	8.52156903377556	8.781559709655918	7.828320578745797	10.44800270290215	8.737020821278666	8.253511880524426	9.862496335361872	7.195318066871483	7.5636843983949795	6.687088690689267	7.138548279559987	7.5785538424776835	4.343969155977557	8.007088955158952	6.298487865151783	7.560462766457674	7.541282507700241	7.591305411758969	7.705044823042114	8.03267943655475	6.753243496463633	7.468493715955221	6.7996313233878665	4.3731540053857465
ARHGAP39	8.736662370923273	7.794214394867456	9.096983905517384	9.381411004525022	8.345737882089724	9.382122508329264	9.856565589306276	8.775606988810452	9.842898714747724	10.126356016041221	9.327130439634795	9.900042606599236	8.814194704528015	8.96570926256937	8.370486897490897	8.139561584242234	8.838475957035806	8.673036943300655	8.044732894324067	8.692329549506594	7.832168486247283	8.847041681565026	9.205150551146778	9.26044914075333	7.780415094861689	8.169554261334683	9.21722586844693	8.298420045946543	10.28281043957256	8.248340330916788	9.100404785754758	10.510507050620514	7.770591219555997	6.9144808103118685	7.378398908300675	6.30097481002092	7.641134143287071	4.60699882573193	7.089985351019051	7.524808094414424	6.6115766794044575	6.873087239755853	6.383063144208204	8.060803994684562	7.634999767745495	5.419815269229272	6.828034904729643	7.9294063697714945	6.811072795850447
ZC3H3	10.238177807523046	9.691705139832612	10.181276514653067	10.471472003596457	9.86503726937124	10.932510053379895	10.904077605267739	10.78015423178456	10.813989565451834	10.790055203127205	10.450314301233329	9.602876987428544	9.969501644532711	10.025001101215887	9.842719613508823	10.0598446526098	10.535946015586907	10.354954576125849	9.896167385281249	9.57088031712497	10.390889048947123	10.636606496120242	9.915991384972306	10.80401805406166	9.783831488973142	10.222855265414548	9.87643406595861	10.452611906738914	10.152373581803078	10.293713035781295	10.182717123414937	10.339159659559895	9.216881634868589	9.827139426330621	9.105075642034818	9.475346363944821	9.710877017290908	7.6754842876822345	9.581675124172031	9.601013035290785	9.562824346509814	9.922081861901558	9.314241080496322	9.463548894791524	10.079524784059002	9.479863105483503	9.913837850762913	9.598478730307942	9.396037437912206
BOP1	8.982825345504054	7.269033146455237	8.931086143784377	9.078871308015128	8.056583528366367	10.322412668863933	9.614967445230238	9.198587462188087	9.658506062429705	9.770581309268614	9.448060538023682	9.990614562001527	9.426663118386633	9.218829592556782	8.945988036520252	9.389818452684747	9.028925472429933	9.231547610543414	9.678221591046395	8.63252997109434	9.500594937533585	8.864486416878032	9.918846842919617	9.822272860951493	8.230606524118677	9.337753455511274	9.725640625073108	7.88603794356646	9.472593370275511	7.451870478864322	8.350775681573017	9.78867918610963	8.061862583924903	7.688795807024632	7.12267271882414	7.470902861045894	7.907569704662557	5.3439656039194485	8.129710609602736	8.453653381062983	8.19653819656636	8.459796344856066	7.859503726951638	7.928311097868984	8.093290786530432	6.985409348197409	8.436386743058343	8.837328326044158	8.187678684160852
RHPN1	5.968611503224006	6.914803677251784	7.597039465735981	7.798517163062406	7.398427333008611	7.767899728420133	9.123384263218036	7.536938766325673	8.577103996250912	8.598149297097644	8.929137069182266	8.866518575922905	7.277343200309862	8.225033869204976	6.318647647005311	6.83961952676062	8.255264670799683	6.822296764558684	8.249805590606723	9.009072788644874	8.016591031535219	7.975956846542808	9.247668849204969	6.603139677848599	7.523389449597729	7.4758874182861925	6.4574137138633985	8.268892836043161	10.520893640473423	8.178231604015119	8.953733759406495	8.398072415432381	5.212592683241886	6.747508245813997	5.86486956015195	6.938403423670239	6.549840103329999	3.0220304048896347	7.691931912026684	6.536528538078487	7.929850162148505	7.187648995310601	7.369562450738511	6.089186680172795	7.45484213017138	6.235392000189692	7.17797757113859	6.048456357740496	5.901084101329242
PYCRL	9.183843769489295	8.714362302537728	9.337044262386772	9.36263206885545	8.484101869979193	8.6800113357135	8.636878338566241	9.56530013774698	9.546095741843763	9.62971906989134	9.639720553262455	7.64007136848382	8.911347043372643	8.953305985959133	9.28001443270761	8.585048909555137	9.929169624671847	8.088931308816777	8.51134547963131	9.211798275352269	9.618697501797978	8.740694870561164	8.80305263134619	9.549877059068072	7.938426948876573	8.337751225890553	8.454636534295663	8.489735590385484	8.706413131943595	7.705521805299992	7.062888026459289	9.714726335881167	8.003501046602821	8.308671221349096	8.073755978815646	8.40095626414991	7.7773999884660885	8.51389336807438	9.26721506988971	7.62481738392727	8.48272326773966	8.487474290394779	8.387539178820012	7.298135903807882	8.567583473643978	7.965784284662087	8.25890046483218	8.141790395676338	7.489920193709512
FAM91A1	9.438392665587529	9.58027552919677	9.394428395360615	10.518407276671365	8.716699446055493	10.630221613424347	10.826206839534995	10.425824329984751	10.367775106967702	10.988358055449899	9.903904434730123	10.818134760810846	9.671855930838666	9.443884257370147	9.8159644199344	9.615460439100646	10.202148317596064	11.236181659216204	10.471553291349515	10.726992952713411	11.345266626007538	10.943496493904835	11.058769702120852	11.41966002393543	11.10179187643457	10.7547706124134	10.391426197495642	10.50017464061996	11.701219811005664	10.712423824280378	10.763328574622966	11.029722040803787	9.33202983293078	9.151995767240694	9.244185419920552	9.724752577228102	8.995365796924307	9.8820912352532	9.601074338941668	9.039028695770503	9.75384517930536	9.517990170055922	9.614389618607856	9.707334971913784	9.00867792064373	8.809382286801883	8.985338186299655	9.3458953027607	8.776610685205695
TNFRSF11B	5.769829911263765	7.093771426955753	7.366663174598116	7.902326873092948	5.124398448127312	4.743827299150174	6.169862884068571	5.7649485324198455	7.074323461036307	7.372151152022099	6.893192300602642	6.4121396203261325	7.731190031348395	4.267738030862219	7.759834933979272	6.483962457236207	8.939023772654137	6.498833254045011	6.637705874054525	4.752368603135327	6.514753498439754	9.184039725994502	4.916624728050335	6.932557295996994	5.727438568390487	7.45057618632048	6.54878800761623	8.878949059555577	6.647918864107767	6.593271593590955	6.6924411473477745	9.493533258063378	6.454481906893987	3.922340588205618	5.24820084029487	3.1310461490709036	5.062963556559086	6.2184363364085415	4.78985964091365	5.29848969805903	6.226658802715528	4.723995344715188	5.922511857336932	5.8447697781713694	5.517430034422926	2.7759565518494886	4.155611626162185	5.197598084380388	4.076174256724248
ZNF707	7.645788107033837	7.472284675095703	8.207844058370307	8.251349851745733	8.38004973555269	7.764486820753576	7.943979914343739	8.042173721468332	8.21311394291186	8.605009037580727	7.709966051535736	7.7626345090144016	7.764420470728459	7.275733428483265	8.41230732228271	6.8873181121555636	7.8651393706027	8.136811724990459	6.89616284321722	7.646659506286839	7.990484092998002	8.926115090536118	7.796162938567278	7.706606525477615	7.948209519458134	7.641618277631039	7.465713319595965	6.768197560450533	9.060420334402762	7.727505279747476	7.79920389848339	8.1507424388636	7.042666222786198	7.426684081849619	7.223953422720957	7.1310543829501825	7.924444361608158	5.68500383040852	6.983632096789092	7.449940981256892	7.261991456353267	7.384507485849958	7.22707678732014	7.07032492042511	7.7781954322507385	6.82035769232217	7.792237182295482	6.975205990656328	7.543078233290128
TOP1MT	9.531985706207031	7.823513731442783	9.212104804198956	9.08142485030342	8.102143691160467	8.641437649487811	9.434202861772864	9.32005588548285	10.087781047708466	9.67371894415567	10.902262046180715	9.187433732872842	9.24337870784684	9.375797516128642	9.312771799969351	8.662479682519045	9.243826143707246	9.193922016303528	8.450270992819386	8.333839629374324	9.23499700853484	8.075489845982496	9.394413389182448	8.714070322631244	8.70885972064871	8.8231793271302	10.236755685334643	8.531026591878728	8.577662963728223	8.555363708445542	8.315914870887536	8.922777641039378	8.367170094856633	8.269145384960607	7.651166528004659	7.745761706495092	8.042502074360431	7.713200903158988	8.212763863902122	8.53067943809874	8.522114614890757	8.25562819075785	8.403084476159497	7.805395648980169	8.570868935469319	7.985415040997595	8.495463327972704	8.483001512888006	8.556065323535055
TIGD5	8.70914241545972	8.32643847584845	8.652454227521119	8.567823014400883	8.518114134144076	8.728134801345792	9.119330236518762	8.907443522736596	9.447043954606704	9.19841557333781	8.10800164646591	7.9462805324138674	8.742349861298104	8.202148317596064	8.318646743268015	7.51465883030488	8.783623623290568	7.136816850645436	7.930116372061572	8.141693340294934	8.484859137366891	8.913819903595206	8.477685421657508	8.324468606727946	7.761119024033731	7.833984275570127	8.779341808750761	7.924420607285359	8.406570682795577	7.892269581825543	7.704934195933196	9.111884216472752	7.497412775805653	7.323595247391479	7.822436991077395	7.557318180226872	7.707635219545296	7.051785359863547	8.104556821763987	7.396921539975269	7.918821505822748	7.911901260681064	7.383064872753675	7.6265340477227035	8.289354962302436	7.061365791825965	7.994817287747036	7.344437950896303	7.505311385865784
FBXL6	10.265193075588288	8.483114408657755	9.871778977072088	9.782315182525076	8.313395520005828	10.128393939854508	10.191885843088778	9.748934137261005	9.934023999955	9.71563601757222	10.026993659568037	10.081776341361534	10.616217996792075	9.072882905288267	9.367399461437895	10.130763595614521	9.762219238374904	10.305252368089505	9.854731273976757	9.784162048290227	10.092611807176274	10.18966397600896	10.054427955178229	10.089198864813005	8.924744226298918	9.533913701162579	9.527697889610556	8.426596033257994	10.157309035695834	9.177324850233381	9.387885929518406	8.92952176892305	9.762674368708119	8.571407568732852	9.281123282761385	8.305377785627487	9.409581868434637	7.602988302768433	9.514716026379164	8.679233764688798	9.268492876487134	9.104260643720206	8.696094692482614	9.926331579831434	9.49603884889289	8.348166013223516	8.413852142794426	9.825144831414633	7.259366654658848
ZNF696	7.567788796727716	7.737632686369434	7.908500706259614	8.206814135404066	7.804305295963819	7.4314056700011735	7.852498298789891	8.343785325485282	8.856627834133368	7.824042250649159	7.869840568488336	7.782087435180942	7.671123500427583	7.390598905552462	7.759834933979272	6.544717202818148	7.765276151926686	7.831522602618215	7.082117099412649	7.402065543900526	7.85014307158524	7.655716749951758	7.558045640341936	7.694065313481085	7.769421335458927	7.711474257241229	8.297682076918521	6.605929850123819	7.561570536766359	7.719299826332343	7.357384853436719	8.601885902338278	6.395186218718998	6.998691964826192	6.710544727808583	7.005523500866324	7.301230952495578	6.051785359863547	6.677381387768614	7.414812060565847	7.102217193850083	7.087208225541077	6.840047738299252	6.972152690972063	6.998962690111448	6.842048571715613	7.573465419187565	6.529795964580091	7.313209566244711
LY6E	12.996680027203091	13.80761654782512	13.300652852188882	13.611117038286949	14.146767533759942	15.79440571221076	14.808587167301969	13.910989310372493	12.822323854834726	14.859748301871669	14.307748690090234	10.293276210774739	11.321973178402963	14.42573386898034	13.013551239075788	12.480565551243094	14.9662215353289	9.571240689786228	12.82327169416449	12.058265754654983	13.449316330868973	13.567659047343986	12.680377106527112	12.765522314998748	12.158975679898912	11.794671727071396	12.35232487288961	14.073781747881512	11.972265439308817	12.531739652821997	13.965053735361739	12.517339675945822	11.5999965012007	12.191364628756586	10.743614261668345	11.279099802133754	13.279538038247146	11.785803903462826	12.33269954325268	11.849088377028787	13.223861438872174	11.87029535757753	13.327033292669855	9.817461776622617	11.888526371792555	12.202748284988989	11.818282583451978	11.481678361267688	11.714657657374937
FAM49B	10.30475058888014	9.59945542047849	9.314757693904307	10.339705280845733	8.738504399329303	10.558095400523872	10.6082363376181	10.309089764371407	10.583722151631525	10.776235614827591	10.133437658423995	9.822836880488959	9.974242488681094	10.52450253744916	10.278507536561944	9.9823519174295	10.368582850296859	10.247702074730588	9.79847018100521	9.76667134906807	10.78416859325459	10.285668075662578	10.317627419134592	10.887304402082048	10.911496852830616	10.647476415058993	9.62672202613067	9.655519990108594	11.108740060849595	9.710358750032864	9.859576715553873	10.427962277407397	9.748055283736388	9.73081484831608	9.402003702673946	9.533351148672448	9.555267832893364	10.226604860396028	10.189886316763673	8.88344975490334	10.565187780470625	9.7970113561007	10.668237930475009	9.605399880579025	9.595251885982305	9.462461373504691	9.109809764425606	9.704261607999124	8.055412597602919
ARC	4.439682971594521	3.77184333206839	5.729797458624012	3.601435048876921	5.813468252754949	1.8507594254809083	-0.938707661521706	2.20423463918128	2.619577344041877	3.0317304215844847	6.055744457818421	2.6377029763346447	1.892701876202174	2.7758933602837708	3.28301795334611	5.404235557817422	3.5247156506733837	4.679857262172142	2.9188823141076856	2.813770932015825	1.9741988864700952	1.0452332614736564	-0.648145560867621	4.92399533873386	4.19818766625722	3.2153524392275172	2.522507776366807	5.588651680243262	2.305532832239292	2.7678454501823264	-0.7379277116015898	7.068950209048602	8.036212083908293	11.587679083484106	5.24820084029487	4.189943131681823	9.893356142888388	2.7590066552564005	2.467931546026287	12.730376728360184	5.2619933362197076	7.7326897885482335	10.373506840476805	2.281846591576611	3.2489446344771507	6.202223755781361	8.876713385281427	6.6476311077958	9.993334704293835
CPSF1	11.825113817933813	10.596273631324859	11.412749775538392	11.117130080805184	10.731276598193965	11.63514219687484	11.723336706844648	11.649453793439942	12.106997973352376	11.59261587448054	11.620613900568035	11.620393964970544	11.548513689680483	10.950184058091544	11.062194583267855	11.056590301821023	11.480971914709167	11.550458217517319	10.864333192984976	10.85785661950433	11.649179818660679	11.540360556317784	11.545162379371781	11.842998467639031	11.112980253162858	11.389615207656645	11.793148077338818	10.497861814060746	11.943840764456825	11.341935726886604	11.236402925500347	12.077937257049157	11.270803392743181	11.112595888012088	10.925020284224537	10.716013452894018	10.984681908173537	8.924916397655796	10.679216167735179	11.470857116629098	10.999105082503277	11.083532513265412	10.588396083329162	11.401482211745122	11.693012079353988	10.277798821010329	11.15983350337041	11.310970571099192	11.066802286928551
C8orf76	8.672344011282153	8.0386337134005	7.821914259309236	7.625087802686818	7.428552798369282	8.539321686837578	8.455799820750887	8.26168124483959	8.751195895772454	9.405081881579866	8.462874401066728	8.182642644437047	8.318949463595018	8.107049645236911	8.608121729300965	8.5261196870605	8.667232205248816	9.168830041352187	8.649076503186068	8.234429467593543	8.936985321696588	8.743753934342891	8.40078126138584	9.046461047389702	8.48043882319766	8.659403713119637	8.071816288926906	8.244278198578144	8.639007810200372	8.55743689613238	7.969501644532711	8.47720779250232	8.148201999108018	7.598841578133285	7.704948024785732	7.428728651517704	7.624020450143632	9.263623667728211	8.7229611828139	6.796714547818124	8.349453744701245	7.9913857898333305	8.517712688017308	8.036717612106866	7.8822639864635375	7.8104816643163275	7.189478665867684	8.472394350684898	7.0816698379626315
SCRIB	11.726405510159147	11.156632991051104	11.571837951463374	11.980468074751704	11.125316598718948	12.173633417609471	11.804909446886803	11.81396552380682	12.659877743529862	11.896411878625004	11.910252962799278	11.638630492733913	11.799075160489467	11.440552570294088	11.019986789678207	11.149988395153645	11.773559588021879	10.979153637276402	10.972362381470234	11.416982206079458	11.833012606396418	12.471035003436322	11.776618086929348	12.331465625114262	11.112159281344335	11.227700168355856	11.432400964538878	11.381029156448518	12.480638755599145	12.321079698591534	11.223259629341499	12.054695882441152	10.468929450612253	11.171508341674267	10.60887391121387	10.768879033538443	10.916730407463096	8.420069692146853	10.65652318486816	10.924166708529963	10.192724325371337	11.056644488313456	9.900770222652943	10.704664506810811	11.019528182419409	10.374007013616687	11.202736043250168	10.855304553401387	10.384125377023794
PTK2	10.94112098672466	10.471705693542777	10.916357211096791	11.064096033801002	10.644108639318189	11.04408669419675	11.250233270107625	10.71084086515103	11.812582454958386	11.040248618017383	10.729093868248777	10.909165554107254	10.73328654320124	10.372070597529401	10.669602608571758	10.624283706321771	11.48527586764297	11.180157809098542	10.65246856867557	10.953890868823285	11.882169205174822	11.040584258321166	11.117266471168016	11.568022601320918	11.100695186878992	11.123849811410707	11.2249904740074	10.701453756635573	12.263568508000835	11.139557747309448	11.152823533807073	11.828945690071604	10.635663712201126	10.4822533551909	10.638987906228895	10.80524408116307	10.601027897021115	10.422916251716105	10.758531378789547	10.720646138685733	10.445428759238323	10.879629194583119	10.423378576518493	10.904882951553512	10.399609049381446	10.524149891285237	10.701540393538883	10.448911272587152	10.562976943014556
ZNF7	8.764758823958045	8.247068284593816	8.290484306021149	8.939658545669358	8.362404134864784	8.318181243323615	8.867677202152125	9.393542763676146	9.397101291059668	9.256604991845318	8.66993383566896	8.513131448309698	8.212564475388453	8.598711335567385	8.448772959128835	8.046834798696528	8.925426854002305	8.256359674056682	8.17334733974446	8.535217045453418	8.960120703276717	9.235868240243212	8.825979559240858	9.03127387844968	9.123128147662342	8.941314693007218	8.987849499515486	8.692402786563546	9.40004900742891	8.922554671443049	8.40857176201807	9.211788543211092	7.944741339184569	7.867606166522016	7.706931092069249	8.01173602325715	8.019751945385174	8.520555825216592	8.172527518700003	8.34033082109967	8.569563243134661	8.301391018451692	8.559782403167558	7.963896004343884	8.175285063489488	7.678156435548737	7.751430245710345	7.7852628209919486	8.694761820106736
GRINA	12.721477333786954	12.02962887744197	12.690753180021833	12.472607077246941	12.210558723954383	12.331818280856151	13.022334866491473	12.672144643296347	13.467936639128464	13.443785340500018	12.38121576622322	11.452663380639413	12.56913170637083	12.50497536213709	12.627764131900012	12.76578127962578	13.469641817239516	11.806610035086237	12.180785634026444	11.198193315251453	12.251790089630699	12.10214631632101	11.67302633970603	11.686496150067837	11.54530237397191	11.957834814184242	12.31223348292876	11.524223366344843	11.011164345837678	10.964340867792417	11.894984451634432	11.848446895857963	12.079824750454712	11.644924290307209	11.626325052682603	10.760403933540335	11.900481937365045	10.156462350279622	11.48613109618149	11.93062277475035	11.802152818329587	11.009394764105934	11.704768239362599	11.815339261719092	11.915890579840745	11.057890216253918	11.337387751648583	12.502033676535035	11.990940682899652
ADCK5	9.451229660649327	7.420507666229851	8.361553447811454	7.760773164053345	7.1535418986081485	8.225781026195188	8.140824659355475	8.658736328289425	7.583722151631525	9.095033006277312	7.849517697142375	9.115990392644303	9.319879817957839	6.990206097944187	7.764148403717965	7.712987763886716	8.80174068785487	8.573991727054464	8.092149284202204	8.209896828710654	9.19335286743505	8.425215903299385	7.970226709464737	8.870414041647678	8.010214776964267	8.601937886264109	8.76035070333084	7.408372203572678	8.782424947181754	8.192134972532902	7.273217473103496	8.089837090119621	8.48798067968703	7.837571170282651	7.687081690152878	6.972348020079573	8.098090000212013	7.448306311427612	7.7762685196398476	7.616004744079728	7.695812908292795	7.830489854617175	7.62579649782177	8.59718848600642	8.534341794810256	6.925702781351336	7.504342313147085	8.621465834460144	6.708435574541687
GPIHBP1	6.6050201530624095	2.1868808313472345	6.836706837113976	4.845359575232122	4.902006026790864	3.9502724332076578	4.0613355467827805	3.467266436142052	5.204551582149923	0.6624789704232514	4.031898005033566	3.2477604548721155	4.580742462937802	2.9458136807592545	5.019990958166502	4.588660674031317	5.536301619323817	2.7592836888473307	2.7933549005118326	3.73130273696553	3.215197100269113	1.9382857918840528	0.9368169398535352	4.339030461858586	5.609119625226331	2.7678878138599363	3.8944782657088037	4.91405260121663	0.08311122693130706	5.324238163242078	2.431917084417998	4.331984614882395	5.212592683241886	5.968634540969517	5.004272160793689	2.546091882229026	5.356161421141236	4.022039285062234	3.9273655352733785	6.9016875222999055	5.035716274444257	5.179188557556959	6.0965401759312385	4.14433194274491	4.248937046676785	5.36092256313203	5.403540417745252	5.799636503519105	7.334907886832833
FAM83H	11.81968015694713	11.376402177798386	12.34466763143711	12.938583528757174	10.802580986072204	13.47194188513062	13.671032362904743	12.344933881950043	13.05026360482279	13.017614008363385	12.67839547138122	12.422335508968718	12.611693410830986	12.607885089776785	12.129141316723416	12.215390421912273	12.563456086493016	12.668971383869778	12.64112601277859	12.809928865721432	13.19703542318887	13.023470221111246	13.648189033024034	14.159098707974863	11.591283903513217	12.100951374630098	12.922446890450576	12.879724908572085	14.218986961843608	13.063614165535238	12.524744931694201	12.880214864733011	11.365562413406888	11.762365427272895	11.71090542190815	11.716952323739136	11.296921940949934	9.263623667728211	11.75044320765021	10.402052749381477	9.99517355971241	11.516310686354952	10.023685057925313	12.090357259972066	12.78439151337422	11.318988993277069	11.681624953058382	12.49402552115561	4.924166411546191
EXOSC4	9.859221055396391	8.16987991651783	9.404396516762972	9.38210088724371	9.098400607613987	9.31950955460045	9.441798835459913	8.688047443204399	9.641954185837953	9.647859519066348	10.211803141398237	8.083261244502397	9.324529362062158	9.528266557986653	10.077443299985761	9.848685528878228	9.636606496120244	8.757683352536914	8.747551882218003	8.55822936171445	9.653025970590727	8.835914602748488	9.15651038453784	9.098682202287886	8.206359954758662	8.219855815434437	8.07529194799579	8.707469573406886	8.227798825605602	8.342159137654988	7.829944671097709	8.035502946996315	8.973407506857273	8.651442816661719	8.382053319714892	8.353442393285718	8.163513827366865	9.43989112484921	9.385303760336262	7.866833814394551	8.682836615722598	8.386345374631132	9.131630637753064	7.610301423587242	9.407894424938842	9.247635622914142	8.350373481171474	9.24355484277846	8.349900420738926
C8orf33	10.519852444347658	10.119096750838965	10.39630508046153	10.983998264310754	10.075746538060905	10.202001348764307	10.77901342720886	10.504352245472113	11.48355381164471	10.348119527193738	10.446369969997702	9.96341921266191	10.259978594421405	10.354260403255633	10.757673353183526	9.62428370632177	10.911204709977177	10.128729018744236	9.477308998322671	9.551485256211775	10.524933432399346	9.612903504791943	9.821821805322921	10.874866111818346	9.455573813765701	9.663816022937839	10.442072641229416	9.279701109357088	10.187735586082841	9.641108848221323	9.404734982939454	10.734523344756509	9.6308477270687	9.589838375772187	9.629790092098732	9.760407260363218	9.473549499923395	10.512552831124399	10.165773791257466	9.922383704745661	9.694735707175823	9.70643558073732	10.037986147250212	9.27772909247385	9.53602180733962	9.570375644121185	9.698645638328852	9.356029334031204	10.446731810696068
FAM84B	10.507616274016934	10.735225985538584	10.60893857685359	10.684424284352641	9.843960297083868	11.17877687647032	10.1770332725864	8.804147158621486	11.66163560233596	11.0517663308386	10.108707395637113	11.032727642197768	10.046373733863188	10.046182717099091	10.797669630859314	9.744085392468527	11.354128141973668	11.301221805333093	10.239312109744334	11.545278237941044	11.213025215644171	10.227964843308646	11.523748161470488	11.976044946185713	10.399641089691055	10.469285677894481	11.35129815364271	10.349325465626118	12.056119471006854	11.31791000906057	10.449654222421856	11.750070485865693	9.757894989259647	10.000549359323905	10.0359537565578	10.91241035380827	9.384891651446448	10.24281683653688	10.133270674627736	9.370319058485967	9.761065820202427	10.283030458802967	9.608652178441629	10.039755290554375	10.790210004414824	10.439519253125686	10.347909214532756	10.11391142539366	7.111792951137417
OPLAH	10.915879378835774	8.987786983061897	10.109987318536913	10.334362682694806	8.55953387590146	10.776400131384495	10.93554814552848	9.993433779312745	10.870565079981045	10.906657641665024	10.692964147187068	10.020035421292246	9.460007352190289	8.539096758373947	8.833513149139915	10.009548726972257	9.526894336757206	10.99291001998919	9.574716505935527	9.994226134621245	10.233715533710729	10.394580592062194	10.189738093401264	11.443632549807052	9.29525925283409	10.06460805304178	11.056414181663687	9.04491864037528	8.310353754888459	9.19312366616941	9.969775025297754	9.615328009535316	9.743109307879008	9.499441317802818	9.763373395133431	9.516192477879367	10.08813894610106	6.944870155947035	9.093642039515704	9.135429696775391	8.627468223027297	9.176001062858719	8.096541230082472	10.541358019626388	10.18075456001156	9.307341997326718	9.138607152586816	10.43736716361675	8.109282195364004
RAD21	11.961001043438138	11.60340844078375	11.4558409091264	11.856931239213578	11.203929124385493	12.127020592308124	12.293293456374759	11.938774636098149	12.294391002089503	12.258433535404011	11.969706684962944	12.238198710628927	11.660481761654546	11.856861228319772	11.965243172556892	11.61807343523112	11.702207323486123	12.784424232694656	11.744623639343585	11.035307735739924	12.773889213156176	12.43137746172511	12.383310089154213	12.76108785015184	12.192277400979872	12.229654009502609	11.863593929931154	11.3888245380236	12.712552793326793	11.985151725480193	11.97934301868219	12.852433924765402	11.332663184341111	11.366710159611195	11.175206456547517	11.513658518612774	11.255536139169443	11.606298788035057	11.748616369393025	11.437559630806804	11.433517633933278	11.682476735233656	11.883426079632073	11.474344776762223	11.010989582594092	11.415255859443738	11.245005947210103	10.778151069869295	11.931646126524853
PARP10	10.980168145574938	10.876447879723663	10.675269031429641	10.967752220581987	11.286944661556598	12.005017451042743	12.528942415064895	11.545418221299617	11.186182348000253	10.924589848301553	10.726311837786886	9.726836324752307	10.535333705429833	11.045015601239731	10.358431428626352	10.58865842558956	10.494585616294467	8.890048388692069	10.154906692870052	9.825617108196866	10.435482750479029	10.499078504037524	10.558650301637325	10.132049821385886	10.637358468407927	10.82244336468623	10.606849335235108	10.012079759286502	9.625528038306149	10.246431840591423	10.688512593479121	10.407310258318192	9.811798528287175	10.806050110102673	9.989962098963252	10.208758657705847	11.291510320187744	8.158175425930203	10.434440581685958	11.020785529939191	10.898850757869392	10.476725955445922	10.56191797218879	9.921748720629877	10.319909164037561	9.353389460222795	10.409465190589158	10.43053618373656	10.463575459301563
SAMD12	8.696261649738847	9.404843530744442	8.981889747424276	9.134043775626754	7.422577820110286	7.733381458686796	7.968229022898921	8.820415136518777	9.973998643605771	9.003084968368452	8.19646934575224	9.006320467643665	8.240428824396735	6.275731566435521	9.439527564155485	8.648706303521577	9.598424760527719	9.430707614327307	9.342354732608774	10.042056043492222	10.177419537989236	10.394891366442588	9.53671735079471	10.482959174696607	9.818238637762391	9.499525031100545	9.313098436092218	8.941534782217419	10.638046677756385	9.906191620901483	10.690609153398885	10.086494531643362	9.016674595465268	8.157003275300893	8.733625498408232	9.470900827991501	8.228366575214642	8.095286244880155	8.404571081562413	8.468412255624497	9.24737453257037	8.890036225007954	8.060577053041177	9.378908223171479	8.58067312962826	9.11134958022252	8.946777639376736	7.952485004121652	6.389831786278331
SLC45A4	9.392547119327773	8.969884363100364	9.366658803821434	9.703337801516454	9.407531207325876	10.848724645279978	11.277176955911944	9.005133275612101	9.90234496380439	9.896112879569074	9.691061261428967	9.896079569508979	9.498368560469636	9.328553762780986	9.603748727806401	10.325260475587553	9.486396616953384	10.46714699362179	10.644541307216633	10.724326256462104	10.117915772252323	10.371330156412256	11.353130280735316	11.312627739013227	9.064058298878313	9.2850299370168	9.73482645347364	10.229023057817663	10.188613570346227	10.879744050607899	10.648393536578231	10.43584733177138	9.624408005042893	11.413146597827295	9.664738816380257	10.776103988073164	9.093124373683752	9.084534701460893	10.550082992980474	8.970698381451749	9.678483944215772	10.790079646540082	9.1435821926869	8.952971244812252	10.585544721065677	10.432385304150152	11.326165418303269	9.468395963006387	8.16363458235318
KIFC2	8.025056487235583	6.862141394323833	8.270926002627446	8.474752389057915	7.619728455598638	8.658536409645613	9.8929070492669	10.313279868655155	8.630765838113769	8.57759499276414	8.794120148547538	8.675798292480708	6.803252872432831	8.050055411644019	7.4343446643296485	6.631305127863884	9.330513584006667	8.13591958365349	7.553291377662222	8.629629831905232	9.2207483260818	10.186559982018213	8.759751728102918	9.183024929072532	9.311605287006483	8.85578418829895	10.220825699368024	8.651532509393974	8.903957141007245	9.57874251737338	8.45366572605163	9.219134631254446	7.699697371562617	7.805582668169007	6.707400548568905	7.437444153574	7.545883447698389	5.080930487672984	7.449668664877271	7.860807647671286	8.65302417864669	7.193623918708719	7.533142812337848	8.239493515366357	8.022217986917719	7.503881378053863	7.304447155873157	7.354355183049545	8.048732070442105
TAF2	9.223287387483623	8.991017239730192	9.39067413351723	9.601430963069587	8.755238114600704	9.967456841014274	10.064850525061098	9.610225743645215	10.582894659406849	10.493004788803256	9.38003457906647	9.930384011395905	9.227577438946016	9.319884332778187	9.272466495779778	9.016267872123784	9.68945991701494	10.312780874193646	9.776868900673016	9.7313054526549	10.77272694923985	10.41122496284635	10.050256801598989	10.897724470216446	10.314764490216222	10.094328452584625	9.988670486947946	9.59731700362733	10.587955615833515	9.950676906311362	10.32200699011514	10.753576124384704	9.351659244674767	9.118167622405918	8.895896350429732	9.41490928533105	8.854263758010392	9.310519628263714	9.388286419762647	9.492680404981034	9.34259474472371	9.35475847859496	9.54700246509911	9.245517054372645	9.102390435374419	8.858108469014427	8.849101673011202	8.797512385624175	9.137949173905382
PUF60	11.962346577969162	10.974378737097592	11.62741076681092	11.477788617352699	11.235481755940315	11.58883173270021	11.834684357139361	11.283453033203113	11.936836663917772	11.732167425663384	11.835596481894017	11.287239097953613	11.789354315797697	11.555399180785054	11.676199519369192	11.621644401976775	11.876152389793564	11.68643486905806	10.938939997020585	10.58085210827264	11.861269170467967	11.906304381665386	11.311362333939964	12.0256586729435	10.8139014107947	11.042541745274736	11.172083671196807	10.768680579453914	11.333244816996384	10.564168547918518	10.694793154999951	11.528517599670872	11.047089821563784	11.287902802727194	11.188366304887683	10.920583608612647	11.038630970089251	11.318955110758766	11.666685591366033	11.284135837039127	10.98198818492664	11.495165485689888	11.355642909800283	10.682713739795087	11.479507459763852	10.954625273862767	11.569243282152964	11.291113128538681	11.601747578841337
DERL1	10.985030726559604	10.664136133766915	11.00786123722153	10.706651416697774	10.53136196450565	11.04620318439072	11.206635898755739	10.73483661243937	11.235350112261669	11.412220509309607	10.969321760510393	10.899296494640168	10.90204342232664	10.880486565561538	11.327417911404982	11.242292861048249	11.226496488032991	11.601417962696543	10.899500429951816	10.853293958616224	11.250943814408673	10.87561881961999	10.696141651163847	11.796704815365095	11.090244770936424	11.149245390949854	10.193266613167424	10.628299713938295	11.351877843029497	10.591026714865231	10.72372066443522	10.919444371316347	11.009688678957708	11.118597224029624	11.313812694892244	11.264618527919144	10.849483293681898	11.177699827153909	11.572202823025455	10.441865217388118	11.183542341389723	10.957457591594407	11.07173719927922	10.672982157222869	10.934169998509773	10.992704711085993	10.986126464179273	10.947709983952748	10.163033222997708
FBXO32	7.2933888783021095	7.914803677251784	11.21564826513998	9.283502807791889	10.92225732503319	9.064365540263628	8.98317888923001	7.417582511602298	8.18727288364929	9.222826291707449	9.11369008225558	9.182563195955726	7.269650350205413	7.61509254917763	7.58558603100111	9.368971279273614	8.13847148504473	8.235028133014321	8.166815851761946	8.184939791762638	9.124855399929443	7.449751190420481	8.432216161119086	8.214673679040292	8.452998945455125	8.034683432323913	9.818211470666553	10.02819616466563	9.598841578133285	9.985870392246207	8.647850527180445	8.761970018603236	8.911272132809671	9.755231437064552	8.31858799912939	7.404068618459391	9.399045023430984	7.767992393862955	7.189033824390017	11.12653670785191	7.900250965703087	10.105188290468666	10.036572016168716	8.063190123135795	7.468990524393564	7.150996236972222	9.797666388862556	7.975205990656328	12.550398091946207
ZHX1	9.461416008237107	8.933398546017822	9.259547910027235	9.770275708659971	9.339409112038155	9.438877081323945	9.869498225863225	9.370144659614704	8.260185653715498	9.581852095998974	9.359692466238174	10.307121100030544	9.199505492904638	9.374765640948592	9.101805005390727	9.39182570630689	9.138474044927765	9.996247498242525	10.014285544663558	9.231622007007994	10.552592254187353	10.223326006619018	9.784382924429643	10.782547808160402	10.447785800544839	10.323178639842991	9.93335723290992	10.349325465626118	9.689237998475845	10.925235454037017	9.6139827929088	9.688066681715073	9.928801112817926	9.436215986012703	9.900952823844664	9.82685828390863	9.7758538641496	9.94090230216792	9.175292549641542	9.784175138189262	9.411082988557824	9.901654340704601	9.377451321044234	10.564711593198707	9.26726657849074	9.800822264017569	9.653386106165145	9.850700989428557	10.16540952031982
MTSS1	9.490022517985135	7.3056240283455	9.22588463354803	8.50545827859626	8.284241122091563	7.346753054427425	8.00306247423421	7.998646839005795	9.281712157760337	8.652066962453894	7.555455742981717	7.53188046870699	7.38097181642363	7.433635512126826	8.096889057929632	8.419968602319882	10.230368754654545	7.039401635407714	8.553095503599936	8.284000459252118	9.202584237058213	8.06282328322633	6.744066886661632	7.1755345809601785	10.128342382347837	9.105316646567127	8.886010498946858	10.258695576199955	9.386084502511803	11.847957770416468	10.237807473723135	8.876302061088317	8.67999726575316	9.415448129901739	8.497440720041746	9.671422532636244	8.884658968066807	9.080644340523653	9.158402672499342	9.555630209008356	9.430534092993872	9.39550628807323	9.345618142190808	8.56723359663027	8.549195535424948	9.047423874225847	9.009920969283838	8.631035144294364	8.315394142880217
HSF1	11.617191940251335	10.828628408527152	11.845987851655424	11.649332532332648	11.79571500650173	11.810788504665608	11.931738451089748	11.67949769324013	12.269532891427083	11.664780585933512	11.92569165626957	11.112876028664994	11.295367173421504	11.298910288691388	11.135491262380459	11.428386346179513	11.600679534955155	11.357161588427905	10.65473447210127	10.55204462697889	12.02385482568019	11.40931667786423	10.97725129831433	11.571634151857067	10.363094401238122	10.758606327663639	11.674108388088628	11.366890436740597	11.51633531190165	10.71884107472853	10.599708321950818	11.323381328812541	10.778504288057867	11.258695576199955	10.57868403079464	10.623104163538063	10.7865065173505	10.355461221614048	11.531176739263051	11.510610891968314	11.066741754692785	11.092783563663984	11.166194472536148	10.045350134472363	11.057680413508468	10.948549747245734	11.26634148494776	10.974708548407564	11.616829085340783
COMMD5	9.307706290109975	8.677113547197042	9.294147430527168	8.89399919570686	9.319245345211433	8.915628453530179	9.419406160835257	9.480840679395392	9.55857760260687	9.953697389047191	9.563257297021652	8.572018029481569	9.035665144708528	8.903158811119866	9.373071766312549	8.995306432283819	9.650836347490062	9.105135896942395	8.434403049566859	8.295915836221367	9.085889339803218	9.12525330565299	8.345507263052205	8.697613986863821	8.507564741903094	8.814902851215363	8.34441575760245	8.58971480718859	9.406932017055457	8.327355031849747	8.200319940099316	8.527035137479178	8.848807568544304	8.898982226761357	8.658932649995448	8.536134515916132	8.695329224409322	9.964514154086649	9.349152934401255	8.482053646435112	9.016482391189552	8.773960072724389	9.23735577674538	8.164429300482725	9.17159215394643	8.831240729434839	8.694852341274737	9.09286811319335	8.527992999805221
JRK	9.0686224477591	8.200908410918737	8.890887435323016	9.430320821321844	8.869436533869225	8.910663714972152	8.337461345851747	8.734055856761447	8.73407279737739	7.963225561350654	8.415023410391292	9.492404020481056	8.932211798649003	8.047347526150725	8.83556411330968	7.875540494870669	8.894699554459837	8.477458769908738	7.378320869712655	8.483045865851158	8.97929942742908	8.596514046486673	8.734106678012521	8.097731929048066	9.309558206556845	8.95141877736797	9.665065830764703	7.6016296684775275	10.044585373068928	9.924745710622558	8.916625922211235	8.487361735298816	7.82776822460179	8.48092749793943	7.895896350429732	7.980716540270678	8.337381061080318	6.740860117874614	7.147408465439306	8.052970101869416	8.052627822241853	7.417253376764386	7.994658916406118	8.036717612106866	8.929060116922791	8.106876962330192	8.99796413879244	6.6286496720556425	8.898172066965868
EXT1	10.454422683499885	10.617862292782132	10.830721178385398	10.426977538351313	10.953148812832245	10.176522246548958	10.542635314722984	9.938265205335336	10.437939288083017	11.443306196850378	10.312361131585067	10.697827672234599	10.62814475744965	9.498693662261477	11.098940063214071	10.740843256770978	11.340973887532188	10.650638920867667	10.635990130206642	10.815022977365468	11.261060621633376	11.162674552598581	10.201511344500146	12.161717662031993	10.735936743624281	11.223971517214004	10.475682773650046	10.492063474016804	11.381396943923583	10.444424814057925	10.960906953765717	11.329476824919242	11.154552324958443	10.051630401932105	11.034798962577268	10.140408017933229	11.114113208662319	9.733993174752769	10.241899754507111	10.61074803897785	10.022034844982766	10.313790025443685	9.93173032676512	11.652320668674513	10.411563949412965	9.721403421694738	10.086348565615822	10.845568456152998	10.254662536921698
LRRC14	9.591911251462781	9.228859566276302	9.915183252438725	9.94609626691259	9.72281436047902	9.800251188274757	9.747093634135323	9.791635025243249	10.270400458312368	9.420743443367607	9.30313687033682	9.228542147443488	8.953186652372185	9.385342591754187	9.547197239772704	9.064731988276462	9.680315565042651	9.274170790975097	8.329720109895476	9.374398332542498	9.816982023814317	10.112543762818662	9.269944831663443	9.808418839290416	9.539992385928267	9.394801355922745	9.889931308975422	9.050703025117357	9.758797845987457	9.522118539187344	9.339391295434325	10.094618759828808	8.961617540827323	9.281116326108416	9.177127979480547	9.293609589179663	9.309308086450972	7.969392277719989	9.308489208241784	9.845206188452202	9.563650082420503	9.284608980958735	9.160292481722335	8.663116867366103	9.364859104434741	8.580605300340258	9.372991263208418	8.565471513349445	9.678589576314915
LYNX1	7.365683789747648	7.7718459725075695	9.486444888207547	7.111730365856189	10.63768450323359	7.324153538034431	4.992075009310862	6.8709934496315554	8.217376141858702	7.671852393421181	8.00459127697631	6.290640039987265	5.767154216977448	7.682776934436351	6.221623852731244	5.23020685685832	6.988366577206477	5.53690147766844	7.572328848122677	8.720658107874394	7.350806615421626	9.680807823428578	4.81127064230243	6.299861891939005	7.327471805985411	5.7514101600643714	9.306182478743183	13.946412867320127	6.127536298967873	12.874214856373337	10.495605231190781	7.834483691287357	7.338602690352633	8.961021308102898	8.087261274186305	6.755541909804473	9.134803630612218	5.759003991213639	4.825485458696463	10.027449900187355	7.771317788480183	8.330128108719498	8.783466476596963	6.570593472019663	7.389420538748433	6.633941178592134	9.147136223542617	7.083734601932958	11.220390420645726
ZFP41	9.101366433595285	9.045953463592861	9.407051024998564	9.048045415339649	9.186101855840073	9.185990397742271	9.51523660362886	9.286370626495952	10.102645010169022	9.31268332329126	8.694253403410945	8.507806530493442	9.06765484902039	8.184280294419382	8.838860398413676	8.168852600401072	9.415522073255415	8.829097960431552	8.296705177226885	8.182826352294523	9.61648635627111	7.07558611086417	8.794708274899069	9.00733007488883	8.92593119739979	8.843701255106392	9.069449767427784	8.303671172240682	7.738443599234856	8.782159531754589	8.474934864704913	9.16967952202766	8.396446375700945	8.576971776326232	8.403732125844806	8.411976981628015	9.160289960262164	5.9108621533191235	7.982805384806761	8.773373316519075	8.099347810403193	7.945420425315089	7.764194857815411	8.643538761947388	9.0617653989307	8.045273676572174	8.679177453681685	8.010281924605083	8.923639466059564
KIAA0196	10.286245856267863	9.625677797394106	10.057260716464198	10.908332569782425	9.757615022240754	10.570377541718752	10.57061472176119	9.982965062652868	11.410514951646878	11.131625493639145	9.836673704553137	10.631759110294915	10.21009902212005	10.147662850685126	9.959215188064453	10.110966084901454	10.437793677925281	10.933506249629444	10.367349222353312	10.356374936393326	11.043697194925791	10.356484983460527	10.454905858944127	11.011960997967222	10.763204065898455	10.92271818584971	9.963011647599428	10.088589506704624	11.499412415655344	10.414664097263476	10.669257130316531	10.860326576030621	10.146708674642138	9.88460860441497	10.090615289917261	10.27400534576566	9.945950007698023	10.410059088655322	10.21276386390212	10.11173558139999	10.222179060590449	10.293356688477829	10.002702520379824	10.497033479072847	9.740792672277683	10.019131995160885	9.886934180839628	9.742287538620058	9.809525381881363
GPAA1	11.945209708652488	11.608952433399296	12.085801061445098	11.970512175720827	11.390416192733728	11.682274811744602	11.239067414713615	11.478961696428016	11.89021486881066	11.973482129707362	12.068254299169771	10.605303568913223	12.009604709395612	11.032390168866524	10.899205847469716	11.382553781286042	12.833933692185317	10.669186252755217	11.494740604213197	11.294224412776806	12.233820369958774	12.141746338450591	11.157852169141737	12.065981522631246	10.970271294818703	11.086335295244615	10.834589557852878	11.042938394431907	11.443803467135588	11.333451687459847	10.731123829667379	11.643098575987235	11.116428440906024	11.384265711232924	11.062410476801253	11.169054362873954	11.042500706378412	10.420065480212148	11.691028100017936	11.28465640247773	11.249208285753983	11.543346046011576	11.180139156654562	11.368091708918959	11.720983802676866	11.016752584099958	11.406502656812876	11.603348163597493	11.636887399099002
MRPL13	9.769831233327741	9.018191831469652	8.961782474329173	8.869205165393737	8.498370555182518	9.55612665071345	9.540106722581177	9.236554802172217	10.16592449715959	9.890910228694334	9.79919904065445	9.176858796241856	9.503299038301824	9.142206638125188	9.715982575530981	9.41647876971538	9.754773952251979	9.690031156516927	9.314064301852785	9.376010316459565	10.88843123996846	9.59266445648835	9.65562195083178	10.451283244781065	9.02415620084665	9.23401983732443	8.685344507444812	8.735214136603334	9.254735750930914	8.875138053620027	8.946736707627814	9.085384819268862	9.485611982620567	8.677229852500705	8.614327037505321	8.636200449350143	8.444596672789828	11.012617555813112	9.765995460041257	8.10898437318524	9.579259360194532	8.722537747583715	9.523822636907127	8.766379827930178	9.11818319932217	9.596139428695407	8.305359543754095	9.334224114382662	8.666277270559751
PLEC	13.98823199858788	14.060375071972468	15.186949893744677	14.570531713181667	15.329789332108891	15.72311428466807	15.422767803526726	14.138367805964492	13.30171052559045	13.590984625084642	14.232263517322107	14.975879307243376	14.761764172146092	14.564399605323608	13.568348058855154	14.429184409157484	14.743219781623498	15.339432495996776	14.946792074850471	13.875259796261409	14.512419499047967	15.299497816407829	15.497427745999444	16.884146664826556	14.82575880083269	14.184239995805104	14.077124474380033	15.771017400727125	17.52008917373338	14.785840676628256	14.439038690302374	15.762389825255879	13.745086034867708	14.320060117610819	14.17412985916108	13.586030507397693	14.784256538333155	10.244435195022962	12.344257065603376	14.55302924605974	12.543660203342972	13.230637443278269	12.162466860551424	14.68302749149617	15.09286035201732	13.454337854091053	14.185247123619243	15.058520287038201	14.684556876489275
ZNF34	6.857296796470683	6.198112999211606	7.406876760030393	6.7586496296828935	7.486963702210991	6.020666649836348	6.623580119954529	7.130230762318166	6.8943812517190635	6.4438519418192275	6.344175160385658	5.684392372756302	6.11507695274672	5.658528555133311	6.221623852731244	6.192732707733689	6.663273450728284	5.475499987264951	5.264664031056833	5.632527062959738	6.553672295762403	6.509326887884325	5.618629234724319	5.73134890298959	7.028768039457737	6.820217263545022	7.587327482778313	6.7605004082894276	6.564268407370646	6.617020720850056	5.824230687779691	6.910252962799278	6.290595809564139	6.968632237211521	6.53770607718131	5.938405776208136	7.01124962261484	7.759002659190415	6.362750404994704	8.070807906349058	6.767231037023876	6.954673251676543	7.155435551626413	5.82240129834756	5.918791696903783	5.8203525860606655	6.403542121929467	6.076745063569112	8.404268773038599
NDRG1	14.000616254183967	11.913102581924925	13.638537731722542	11.972577785176552	12.914695315617207	11.368129914801006	12.164297296936617	10.89632483466112	10.892049438356253	13.27907948332339	12.361019868105505	13.792383614935188	13.426173010639655	11.795824567952684	12.113169140876016	13.322421119291615	12.748949646565986	12.881750640750894	12.588805972149716	12.302473284207165	13.08395626862151	12.284902159768329	11.422742841711546	13.621456676555793	12.220553665557082	12.887723262175463	13.245417521536034	13.524223366344842	13.121488189584493	14.354793458619001	13.312386659961073	12.915638536192064	13.464584037910829	13.030296415300478	14.117326543785056	14.24142460781397	13.918136490382176	13.063469236604394	12.985109021137589	13.359886796473765	12.433854030733425	13.290350098696704	12.398521927323635	13.7251105893406	13.417153672067943	13.431040486893757	13.206652685680737	13.383798777266279	12.541563721171022
GSDMD	10.6379470888259	10.235368064379744	9.874846136670842	10.109647857806625	9.98339268411991	10.675013152303023	11.258248017077781	11.104192487954196	9.96070862158795	10.510071824622376	10.564321002318332	9.40030096180791	10.690347249968758	10.696237303009003	10.714314215794317	10.674421809682418	10.00982861736811	9.994282714780319	9.859446265241417	9.565684752339711	10.443637729417553	10.507576634092741	10.168822521590837	10.281964790272536	10.000267662725193	10.66354031501955	9.3539098843461	9.6964216323143	9.382349510166621	9.680382381389345	10.097650297446659	9.923608271031291	10.377416614960131	10.37962745957471	9.918738039297503	9.683078826187963	10.794326507471004	10.279424864186668	10.65280912920166	9.827784099798796	10.626904506009094	10.5552505746173	10.695097764326983	10.648950717838684	10.939887700637176	10.13790051765214	10.712122851662341	10.840423892827017	9.50626592137767
ZNF623	8.700568029061275	8.747219540967421	9.024519153103908	9.519667700893718	8.714259257553465	9.193133525037798	8.971230265436233	8.59311096063125	10.090681444008684	9.172927489603836	8.259432568336742	8.650692767171384	8.353936341574766	8.435882743330536	8.474164254923052	8.042835823207582	8.799336672807584	7.749601305844079	7.865306429760376	8.303840098334566	8.882169205174824	8.534781431506255	8.864254253112891	9.118518062348157	9.36810917459058	9.007170267478285	9.3539098843461	7.931287112464089	9.380822505288272	9.057593776377535	9.08216767382633	9.526192083565057	8.29059488808257	8.095745126579525	8.295502636774556	8.672793040904633	8.376741849562835	8.095286244880155	8.303981615780947	8.580213335332877	8.290788386296848	8.356636834629528	8.189137633003918	8.468057849654253	7.811875575779998	8.299519423705947	8.394021029421225	7.25443342392046	8.887936453336632
MAL2	11.57043446848516	11.851776364927026	11.820518051651215	11.992166521508768	10.084276910531493	11.787800555287674	12.427386181888096	12.06919128099183	12.857650598789059	12.10396637124774	13.380920965815655	12.339964100411068	11.946760097340826	12.14520934392478	12.638080626956915	12.18261161039476	12.145110607186158	12.94425634338111	13.206503122557656	13.261425035041173	14.22079396473305	13.252668388789518	12.96340367816121	14.482347904618093	11.922235021581724	12.528466320386794	12.656936509164355	13.857359009758643	13.41708772602252	13.817213524462053	12.275661854293856	13.290983585876178	12.081979341689406	12.482258397983447	11.92783720912208	12.963940063500111	10.944228885912244	13.639046712624479	13.033034054695598	10.984026755940501	11.72459485322717	12.529371986176258	12.085913897483833	12.082438482955045	12.10295660630867	12.815563421296616	12.192132506115593	11.963654738550144	5.076169980165247
UTP23	8.923268052885419	8.276999064816167	8.772750038772797	8.704443185768984	8.460971856768346	8.250397910685303	8.442881309377421	8.665303935906152	8.906310395325244	8.723189922723627	8.72119490937323	8.83167776772096	8.49094311002805	8.619805472390098	8.439527564155485	7.726163652704784	8.730388617091856	9.101684214487143	8.646807087918827	8.518826229359155	9.455801875197453	8.96748566119548	8.53671735079471	9.448304246279323	9.513540092620719	8.832029202469228	9.031235275183755	8.819093340971175	9.1027316078489	8.263466398259256	8.299537739572783	9.273844542803852	8.452059991124987	8.17356721249363	8.10064131597347	8.431781727655306	8.388951504664586	9.346970181962373	8.50410391683023	8.129252101719997	8.721423929475614	8.352882086522959	8.80870359776875	8.036717612106866	8.023127802128144	8.322852025483002	8.115615931428108	7.909881086256198	8.557915490212789
TMEM71	6.659125376228163	5.453672310002383	5.2220007795287	5.121686844104896	5.659433684659165	5.043383879204511	4.615928259565067	5.497015110594358	3.335797646384643	4.791777612703735	4.125006306965968	3.9443618484195335	6.1657029540447015	4.360848839548989	6.221623852731244	5.499393080792079	4.76034338402344	3.951942084372198	4.918887083276544	5.302055788098619	4.537122088206698	6.903194971458094	3.7210773088060534	3.95794244961977	7.386216022451478	6.29715105576363	2.756980562590804	4.7985741875038785	8.485442928587544	6.8045053632724475	6.00345607095681	3.862500984665024	6.464132384005637	6.0426355831644525	5.946171730868201	5.675369958852064	6.36442710496627	6.524538580868065	5.753334433522461	7.646609109100978	7.583917671371739	6.127348509180313	7.246902076308184	5.265343123825232	4.647487208113776	5.5833144829602315	5.770324278704834	5.1181645070024855	7.779778440419791
MFSD3	9.581861508785982	7.063405867753165	9.289435659035519	8.90136170632251	7.785812075358369	9.250400279477368	8.842699188270952	8.471020773298878	8.946657764546671	8.796918914166802	9.450312238957382	8.35122194857359	8.900178496384338	7.517354440789683	7.847809043407229	9.117232699517364	9.563455609812419	7.982605762643002	8.03436430069648	8.991465157534032	9.60817164049368	8.721680252142907	8.598979250347758	9.241623324559562	7.389807699696932	8.04486947463019	8.502481776319145	6.48039923733526	7.222688658651876	8.070550333978378	7.89316802608885	8.224102997047542	8.797557777760957	8.462175047387415	8.764775407860567	8.540441928672697	8.494415610397498	8.18526694923296	9.062256655967783	7.646609109100978	8.65209206611829	8.643239305776394	8.246902076308183	8.82380985340303	9.127130250225731	8.368414789621388	8.810250286028182	9.636985249227163	6.470450639199286
EPPK1	9.994268569948577	9.6575453323844	11.898620280614969	11.607940555647916	11.496239627689372	11.382958995522106	12.734758301895594	9.440725928906355	11.151244915808677	10.17193986032423	9.733261118350097	11.30770173701787	11.24042763178666	10.396101676652442	6.4741650663076955	9.406283676400598	10.908415139251092	10.974307029008662	11.20197685250335	10.06801239859226	9.83724474032449	11.300249588728333	12.29685886491935	12.902160277128797	10.1278524941175	9.937822523469832	11.26000800806384	10.811198058930534	12.939632469640543	9.764526629303994	10.396733205291335	11.986318488305855	9.6297773447805	9.540885521034923	10.411881676996298	8.791641536393257	10.898699629399568	4.137519917918615	7.28810935460344	10.513293340576947	7.597814203107839	8.675184331640283	6.583528109693129	11.20178086744061	10.48037621240547	8.218902226668359	9.661425395874447	9.798575483487717	11.813781191217037
PHF20L1	10.004754178797668	9.346757485928268	9.371748334572573	10.042630658878835	9.142681467510233	9.858768976432156	9.817893417788557	9.973740409771864	10.528620152771325	10.24494650439147	9.661254357850446	9.696972740785252	9.215613079838239	9.760304125286384	9.720428966160007	9.436657414035148	9.929169624671847	10.724667323152634	10.28374818298877	10.215120395490006	11.069818953444845	11.351115917433077	9.452941307971212	10.880126837997748	10.816903649029861	10.535780814636336	10.176933574401874	10.497283027618739	11.257234607140525	10.763195764934748	10.146161328946222	10.419739017861774	9.54706225022162	9.376058083648616	9.050966885060994	9.64411945759723	9.352008103066684	9.414355439248254	9.10817680902465	9.665945202793425	9.783528682543327	9.542110986446628	9.700503875027655	9.980382387063642	9.177526677165911	9.570375644121185	8.991663557685346	9.817557708042846	9.50530940072646
RNF139	9.437953848290524	8.647688663649989	9.419022648043262	9.545205827425605	9.074904096186575	9.223791768513028	9.178799280519272	8.967312731471658	9.630478280077936	9.374446153132784	9.157051291552795	8.699249697670917	8.885327264603712	8.723756495263792	8.910318930884698	8.83120271984812	9.336490938897711	9.073664946742232	8.721943362711396	8.853297077987202	9.919161283973493	9.61904429268583	9.337276238126423	9.949492620345367	9.839047855206262	9.224344213289232	8.596342591709275	9.367165725615441	9.867230855032485	9.64252649438328	9.046747507787616	8.93776515607747	9.156634254981515	9.500754259806133	9.177800717924082	9.632224584949425	9.06423348838765	9.94090230216792	9.429004948879124	9.550050276472499	9.558095400523872	9.20546827047701	9.65391808228534	8.6216819441299	8.671877155161432	8.81718194020627	9.132895532854954	8.98829840174438	9.792378734086647
TSTA3	12.232798640767585	9.267704327365218	11.954381725505577	12.350944704870624	11.237180078609578	12.354709450047196	12.224514246503468	11.200849574637212	11.532263379424663	11.838979333909059	13.117060256938931	10.663860487022836	11.904066312170608	11.56867819257584	10.677631562332035	12.41977851320303	12.539364826435708	12.236328177173023	12.080227940340865	13.3063465536885	11.642426311646343	13.131308773798729	11.39646564216146	13.025156868977016	10.731336003808059	11.680104545735087	11.013657658053619	11.680961619735161	10.791651303063173	11.722214111476706	10.376060254846903	10.521256900005259	12.148203270446356	12.297947972616619	11.833830939224557	12.268041911909325	11.193697832946043	12.8025789667113	13.22979518471646	10.730376728360184	12.144635939511767	12.62310645040662	12.277505357331965	11.11299328069569	12.651370161460719	12.574837109378743	12.193161868909138	12.056248202606913	9.705945081378918
GPR20	3.7027129453624963	3.579204289408447	6.266396773024553	2.523436498859587	5.93595234876881	3.29130885982897	3.9193210116610966	2.789186704382044	3.9821539442088687	3.536960517597321	3.1250063069659677	3.2477604548721155	2.9706880293316216	3.2994498212912173	4.624059941632891	4.032268383595959	3.325400806996613	2.858836408881779	3.3339245825553876	3.853297077987202	3.0997159991609093	1.4077878056243622	3.8961652656531487	4.4624122930630765	4.131070850567762	2.308477376594961	4.756975227012426	4.2136116867827225	3.585635601362301	4.51326965016036	6.972796028129982	5.080921962693646	4.142201020928378	5.937937545574016	4.965798134468001	4.452977537515617	5.571428048001026	3.0220304048896347	5.0528940467474435	7.279090513570238	4.307792796129895	5.497717737901954	4.807035432782361	3.2147154431782874	4.789510170419392	3.9458885744060286	5.988505190711581	4.034101239143841	6.2506698226116395
PPP1R16A	11.223416516166964	9.694739188927255	10.052635972700585	10.237592397561897	9.640785393645762	10.766553753879318	11.430781824911413	10.911721537853714	10.517895714076923	10.750305018670497	10.349424993486496	10.200874090046119	11.049399338613648	9.779888398241996	9.997997999309279	10.843944598894048	10.662045238785785	10.676838606474155	9.819070981356543	10.577334407731328	10.722739236296714	11.110528984648543	10.130416117959966	11.13545919728899	9.173017467777575	10.46971303455988	10.659326997414105	9.535800251024162	10.011814279309414	9.833217430817289	10.18625045324367	10.005371915103321	11.00766505227601	10.567746974025017	11.0234286360969	10.44456975650923	11.07075827358371	10.55152563043608	10.561660267114723	9.831270819647473	10.038191973087791	10.459595551941366	9.783245460094356	11.371798420702493	11.42733379815913	10.466117258134114	10.705796516981625	11.947439695273646	9.089619966309476
SLC39A4	11.83224524485477	9.747217862281936	10.780424937827899	10.84761332661176	9.527887470642849	10.132178380915546	10.734201539558185	11.521193090763962	11.389663601927156	12.152842542773888	13.931457766090135	9.856869007475751	11.2171155771934	9.325669737382746	10.224761422171477	11.600781729879788	11.564516310976941	9.715150353246237	10.396647590488575	10.845968256449984	11.873678646748838	11.182574368662786	9.727243181669184	11.025035717727372	8.321837923629426	11.133822931897654	12.784052006630661	11.320501601424452	12.302207650006938	9.643277192220413	9.665533119408162	11.64032176943857	11.841717650502357	9.895632837570199	12.245190204384743	9.478302462483487	12.184534459738588	11.176011039433643	9.940623394572095	10.628108294680475	9.45118019660736	10.000422602661097	10.00416427456992	12.642979035095385	12.824650524405401	9.975633052043298	9.89370652088399	14.116676977397987	9.633389431464618
LRRC24	3.9168396610902105	4.38734185985289	4.047276627891575	4.324126528944486	3.967804950279498	2.914239210749147	4.073460371360959	5.6995433041332815	5.462853956939445	6.113429635079076	4.35112652089781	4.521729196963873	3.159726051081443	4.012380950039624	3.8414498710374048	4.0839451694803905	3.8942454211726707	4.358951795137611	2.856228798219045	4.16426335121532	3.5902906214164383	6.782552722381925	1.8986340347169746	3.435681927448555	4.585749756928509	5.04777393294783	4.561442231764745	4.83097020607767	3.1140671264541253	4.7417258392971435	3.221753372018402	6.2719391787516665	4.877724621311516	6.031046655803014	5.445925298579273	5.143021920977614	5.22573765346606	1.3478357085622394	5.378185592826204	6.150395170718222	5.617045706921684	4.999386724277298	5.080939012601948	4.015782997240928	4.881209423233052	3.849779136634529	5.762649124363302	2.1726475216147607	5.747919047061942
SLA	10.25730533326396	9.25700590227572	8.614319674844193	9.296019117594522	8.446326543014676	9.116187985774763	10.11594099013287	9.382150615256071	6.8675135060955865	9.551742862818992	7.212472066488507	8.299665944130178	9.533329732305834	9.862022027716808	9.653420144181215	8.878495458006011	9.529071299829113	8.180759531898916	7.930116372061572	7.027552188888177	7.37583877578857	6.888913683814305	7.475960353911942	7.370879172254508	10.564111373364879	9.574667315754311	7.47944076642996	6.961808514737477	6.428916204586105	6.93776809805052	9.414907171818866	7.941775374671109	8.631919134147433	8.981861213559286	8.857007469434244	8.654610969091257	9.086892545763952	7.9369205617749685	9.558045640341936	8.49988175474609	10.285078500966696	8.492606307085332	10.323606505385454	7.290009628404502	9.283363897671643	8.94588564894592	8.835093502592946	7.824876025762556	8.139182957697669
COL14A1	12.565870834721833	9.748174396395342	13.524713104469573	11.421107559128663	12.396717152902818	9.029226480635012	10.064850525061098	8.865297149203547	10.868189790397519	9.581355013598282	10.586999244791066	10.776441257592557	10.724522379661188	8.599250835382	10.808151726582464	8.476187241170162	9.822000331832873	9.521792786239695	9.312635679755491	8.082633407447219	10.121624168578903	7.604189961475013	7.4586198743523076	8.66224823287857	13.126838348215118	8.659403713119637	10.031701202740303	10.828779125961175	7.769639521402048	10.188180827978478	9.07817216106474	9.934783325956113	10.613605318063385	9.88460860441497	10.25818911738189	8.77490563240298	11.521006555259705	8.825092346671923	6.959781744938701	12.673063893752886	10.602420504717223	10.073003453009944	10.062680298293364	11.102592524196762	9.438764827778272	9.580090824425522	10.232432919603713	10.11986192399294	12.400802070753347
PSCA	9.148026543671289	1.7718433320683902	10.155691055054048	10.99825898078995	8.799705785986445	11.150274064141666	9.184166215425067	8.452158857205529	0.3971457973927809	10.038973843574201	14.163107148355634	1.3897870537356576	4.42320164970806	9.247562047689724	14.456495597216481	16.854491009172925	8.890191304298957	8.001785357059504	12.085259989595892	15.387125418809859	16.307860731020735	9.02902765656821	7.868853414231924	9.329338907841983	4.230610366535732	13.088851164814207	9.311562148795153	13.294103767127101	4.142037601993326	14.491633920278854	10.284014344738006	9.081664512586293	15.453064814045813	16.922421802340175	15.5848803136041	17.318463371732616	13.422498455866943	14.340392581269843	16.102941438054955	15.034927891668424	15.53637168526954	16.02370887858558	16.169394616476428	8.68348943797738	15.794913331649965	18.398868920447526	17.275706224890577	16.698650412777866	6.50913049618009
CHRAC1	10.429992488595063	9.322595187090592	9.832103594341932	9.837823432695311	9.721574310961923	9.676380255466924	9.96973186335767	10.877368499187433	10.580833269460912	10.277508263205549	10.82327965656299	10.169085689919727	10.231053136560567	9.782211963363434	9.735882602948724	9.878495458006011	10.770804274270354	10.365567881029262	10.24905417889787	9.739900407545356	11.300615676586709	10.776054624943969	10.167756825780465	11.005126256205928	10.000267662725193	10.391941672017191	10.19991768027457	10.78393950183327	10.463606110051515	9.840883329305731	9.829469052117076	10.83558463981326	10.238130027858006	9.819791106196629	9.593973685871392	9.773702984210333	9.521470885780811	10.195298378901821	9.27044951721616	9.836343915208438	9.829396110006854	9.8724286393038	10.14358729434393	9.930271638495848	9.604892319852182	9.879247806994584	9.452529544677926	10.263691734272037	10.258636694773724
SHARPIN	11.15274749543509	10.255665953813061	10.76067837064057	10.64869911423831	10.65816684421961	10.699364232122534	10.789680352260715	10.62503299216914	10.752915808051489	10.997327695417237	11.385630558819747	9.746223737461623	10.488462790010255	10.288127815901262	10.68105388965002	10.838865124514465	11.371417286858724	10.222819048189756	10.180978277981422	10.231149164186254	11.268179380380287	10.914482305232715	9.577851755160927	10.722739236296714	9.651643720638274	10.195310683914345	10.324934332251502	10.408987773843627	10.179411523174034	9.99723594539198	9.935739655349625	9.965784284662087	10.399587688779716	10.723524432211468	10.048663964497218	10.056854441750614	10.039275504848879	10.215156806494887	10.71408234649986	10.572264415613782	10.614424589218116	10.517147719223674	10.536470646102654	9.727787747736743	10.756731436727536	10.746069183571995	10.925450591762923	10.870526551296319	10.909060519522129
NAPRT1	12.11835713012967	10.787731188377348	11.417683768755419	11.66283746619889	10.302478996233427	11.938204914467022	11.900934715667914	11.183802838504509	10.46865458659697	11.678837781308259	12.407950173933202	11.06352316534924	11.708424365737978	10.908385114536529	9.85894143563628	11.946211801209133	11.48153709962741	11.2508609356035	11.330754453769186	11.831505185085327	11.663389097981963	12.209136522806563	11.459820929823191	12.537038780620865	9.546913747125569	11.737741679298281	10.858952310750938	10.840235040888134	10.669044487185388	11.034358797807936	10.545080307255457	10.669203972471996	11.917211688227528	11.56682466541837	11.552789157019262	11.049412953118482	11.972053580061544	11.315126913804729	11.499287830806459	10.706452848802492	10.825960485140127	11.73846386621771	10.313064414946364	12.184208996105049	11.754933420545703	11.818985532493734	11.67131548976692	12.379995604513589	9.260905424106959
TRMT12	7.7739238194266225	7.311003505984344	7.865856969303424	8.036497839003355	8.102143691160467	6.3562688430735435	7.596614673497282	7.868149654516724	8.191611997733357	8.437281845621737	7.8563259216459445	7.2966592971305575	7.659396576947023	7.5572415841354665	7.609754962172444	7.396013882990757	8.164841576639764	7.739841353548806	7.488739930511653	7.725993306219935	7.165801421851417	7.903200998093015	7.329123596291566	8.592374808782534	7.844661831757011	7.520351526447906	7.82950710740627	7.664824131626982	8.53572639135733	7.786289233789863	7.091710411041154	7.9252695814723655	7.374865602195454	7.729695510263222	7.638986096763325	7.540438053902724	7.203230526845024	8.556019326440392	7.66760832567372	7.527156371535043	7.687172694476425	7.797700429465154	8.077285732157414	7.0367231060055255	7.31307348733037	7.235392000189692	7.694775746809966	7.165027565119382	8.156340993450447
ASAP1	9.686525038783937	9.227175487206923	10.038987556818794	9.501980452330438	9.83048668554089	10.054617883435597	9.670516398281105	9.803401420442661	10.431988517589787	10.778619270669537	8.834091759374497	9.487146650247348	9.160511831889448	9.961380234990973	9.58314671866881	9.43264420075103	9.777531497036179	9.22689862896736	9.611212962649995	9.387836398806586	10.885986103291135	9.111307844178778	9.056897783171479	10.162403917663942	10.941187025879303	10.3158401692866	9.25602229397142	9.65136388244442	10.926273753678931	9.218570501107141	10.630767657918822	10.869632396842178	9.410239330776022	9.293595795738995	9.062407778331474	8.953355462844357	9.310889934240981	7.6754842876822345	8.778543711699957	9.81533045473892	9.580426291072726	8.855933652299967	9.455158690527911	9.022073695214786	8.81741540748274	9.102390435374419	8.972597887881639	8.490907019443776	9.963000083790332
ST3GAL1	10.546412195119352	11.168452751644647	10.12136514992584	9.715448978938898	10.57846704373917	9.652563575223267	9.867081012065023	8.699287876831777	7.994624977428244	11.319813222716473	9.88987808778127	9.287617735650413	9.311473597898964	10.504660113636492	11.057809005907236	9.739220296590121	11.273987868051034	10.277240898332069	10.1444925527918	9.301162347363238	7.9741851170770515	12.764056735441253	11.196024195128059	8.923995932771778	11.132949497609804	10.029135356339031	9.43010751811749	11.430055233107902	10.937748975118458	11.191639137881836	10.0408924306469	10.786392161661045	9.341223026541442	10.127555901987202	9.421681959562923	10.049916599498493	9.768085053354186	8.694465845876683	9.920174182426942	9.465964264418233	10.321116350602374	8.604267812233669	10.017935822812882	8.55372143915988	9.847500581685198	10.906529876756561	9.537156264286246	9.401472611985726	9.932018343061603
TMEM65	8.470268409037036	7.9682463058238735	8.566297638929962	8.798520403147887	8.08333571551656	7.374300512761406	8.451507864290106	8.793697589367609	9.203475257998498	10.197437873009267	8.79211677098979	8.083261244502397	7.902670557825164	8.077381877285813	8.502839758255933	8.042835823207582	8.764042217306587	9.034086378587157	7.772717053612605	7.709931599199123	10.056813807985982	9.696783266667662	7.993289410590425	8.367729248510882	8.916172069876417	9.150029027082459	9.000149333749787	8.741089409322132	9.916558749124587	8.649047753216388	7.526428814337748	9.661404017230332	8.432529373314704	7.528063348291953	8.7731556945688	7.705957173156586	9.069299380953732	8.295057167044707	7.534022682507311	8.044782064723833	8.335702979187424	7.583579255062511	8.136955236446655	9.025748633134393	7.959434012614724	7.202221796397874	7.663408668138533	8.572639599814485	7.8894005298009695
ENPP2	10.806251546993835	9.435999387025342	9.443588004389103	8.976472475360357	8.993501712536444	8.26012918594607	9.50428073120564	8.660381035242413	10.086613947409496	9.991536017863073	8.082106451941748	8.209950424904637	9.532527362993761	9.544069955015564	13.64511799597576	10.623405998850115	8.948813989005886	7.957560612168527	10.278635300683819	9.514978310366583	10.266294628986701	7.395208501616247	6.317626062569454	8.279870544507133	11.07134839050983	11.67492490759752	7.435045131278086	9.0839765392876	7.8223605055750065	7.937768098050519	9.371166772640562	9.002834701369991	10.754461643905337	10.427375705294464	11.174201657320246	10.688197846541456	10.946292231016233	10.254060151627725	9.845143448873616	10.264008553504816	11.275740411140903	10.405513792125682	10.554982085957823	10.521541553010076	10.077216290024074	10.871358542051809	10.041316915829444	10.075866846752403	9.502436027781869
TSNARE1	7.6941001467997125	6.764314304340826	8.691642335076057	8.520921133583139	7.822570830950169	7.9888266773279515	7.900540806580844	8.839408522865362	8.840139031674408	8.677441292670634	7.694859304206259	7.389773289597331	7.477644950742387	6.9407892776927715	7.541042392235063	7.328513372337229	7.663273450728284	6.686640587863964	7.416071248126005	7.791045644464964	7.795526703681586	7.792451945080998	7.4071232750319265	7.632340930150918	7.953073130774828	7.328055534927398	8.529122069453837	7.046501973786264	8.521934085707585	7.835197745047723	7.242126101657938	8.107499570260833	7.444766440833077	7.961021308102898	7.392969922957152	8.096836362130809	7.873216462501948	7.123573086982983	7.344446828118252	8.476952722342249	7.674114568890852	8.174940658496038	7.414093239475195	7.7111438264974534	7.702394354967879	6.390669405017836	8.086353873730125	6.882092764115696	7.757463350751159
MED30	8.462682214831572	7.5488296130845915	7.729797458624011	7.796448503803866	7.392326010223684	7.672743548429623	7.8061693641388965	8.415636149852993	7.961976319428245	8.105437133737391	8.329576610355986	7.358440220159837	7.583842474607435	7.58001731288717	8.85591497014663	8.010891705929733	7.885665872064732	7.627891322147341	7.508848532314879	6.736604875314249	9.283764385542302	8.144755098964934	7.437535703851081	8.628394502644227	8.060679721539847	8.065022924818232	8.126792219068092	7.571168622455053	8.366999684644274	7.094014327133407	7.449338515489783	7.795052591200487	8.087064984454818	7.608232640717719	7.12267271882414	7.1607992057739365	7.401058694943023	9.328858898479885	7.915018881743278	7.734709620225839	8.181241723551334	7.6493783432822	8.565875117665444	7.2859292611541715	7.482993448566388	7.4624613735046905	7.631351696739697	7.57015171008257	7.524314470222152
CYHR1	9.621011532324047	8.7124066275364	9.994311004027757	9.370312518908746	9.776472512726626	9.527201342868473	9.397843596972406	10.211535484494233	10.233775441071012	9.78532004474603	9.67190014782541	8.717323416487318	8.82735858411415	9.426040364622802	9.23100271949828	9.230205415550058	9.823184104880456	9.028754228728268	8.724094285047334	8.701836652431103	9.847909240130281	9.417152089398158	8.260736098671373	8.736249704584406	8.939652669422314	9.113403587775245	9.693612403787904	8.87182517125164	9.75261647655313	9.447738284245641	8.337470266106124	9.147899388715533	8.814630528238643	9.400187801301316	9.091736852962766	8.929790997718598	9.034749450744389	9.266798250834396	9.164899386438	9.47268880870646	9.549778893306387	9.128448073253587	9.33523625055382	8.660027991591715	9.207360915927945	9.243564362973002	9.266749067609771	9.24355484277846	9.585325007443107
EFR3A	10.716605141335366	10.446628436899555	10.647746217208098	10.891692582759262	10.682740071232018	11.16815185192572	11.034675179808932	11.161623197626254	10.748872098373734	11.14210705730255	10.764796966648854	10.743748886168504	10.512296032433637	10.820617768613696	10.772735195544039	10.351005460041158	11.255483029812009	11.295126063107674	10.446359630358407	10.859076591953894	10.947037821198208	11.20328681868658	11.40418360427131	10.910245466235068	11.560127402743436	11.376065682828328	9.98001101492719	10.722892895214475	12.104929578934687	11.294172708733745	11.150350232986153	11.162938844695477	10.695767678543872	10.816631703497352	10.703981414602254	11.06129692942209	10.810684089882553	11.03512899804635	10.732370966160461	11.13697189310176	10.648204765095885	11.147007717697743	10.963163414002619	10.98321452391191	10.315024991372473	10.519115294781997	10.799467815936909	10.45835534384507	11.588035284810429
ZNF251	7.8764555539804295	7.0006648390325585	7.610020834120776	8.550719854824461	7.3676681016341865	7.543232932623405	8.50428271776091	8.520740454984573	8.82764756739539	9.218999066462382	7.879895646560769	7.479885330430267	7.352114068658527	7.700696328570711	7.312774068530775	6.436658246790559	6.914815633899078	6.264821639078001	6.204289424424966	7.504930189021266	7.334934694836195	7.874489610597883	7.302154517297244	7.429523909957227	8.68419808253246	7.8003175287896145	9.343438914528306	6.941528913607528	7.808790462048471	7.431923768373536	8.484782623077873	9.480034834810143	7.206858080782663	7.180137913149721	6.84380801743461	6.85897561429202	7.042502074360431	7.137513770127192	6.617679032808797	8.267877517518754	7.9695764696805185	7.1535013654965525	7.696000770534487	7.112418654670016	6.489947480892636	6.7983227446159615	6.962965391806901	6.477708084874086	7.895254142862869
TATDN1	7.301011404587418	5.919290021104429	6.888475381914904	7.06417150068554	6.612345126007735	6.923577075328484	7.191345503702777	7.56477445892352	8.161887682376895	7.28698276847555	7.7796405710105265	6.840979289001977	7.272965636076688	6.859845343921445	7.199878429407944	6.836833049423092	7.24177346118877	7.232017923650322	7.240819947316999	7.725993306219935	8.610652083696158	7.024685360295356	7.6630029778818605	7.66114388501557	7.554327915148676	7.50076222545792	7.916584125991582	7.285078500966696	8.050849922174676	6.9520306431687615	6.996185344650979	7.75802328932175	7.480152003089322	6.099219448233496	5.9518908110052156	6.601369674570987	6.505673429598207	8.397079893295402	6.8100960132178106	6.932958795794056	7.546199947538742	6.851948881433123	7.591305411758969	6.972152690972063	6.360729469569313	7.0348539737321	5.910502426238799	6.808101838608145	7.528086797025258
NOV	7.745028873994745	6.561924843693991	9.626079513282042	7.081424850303421	8.160738710765608	5.233209337029227	6.623580119954529	9.585343787693002	9.190525972919213	6.325458374908231	7.19109870607096	6.1244811650638615	6.877572490315383	5.884409879761403	8.350545866502163	5.827448143364747	7.954225396687234	7.510360671074933	6.760194328231268	7.065906150371438	6.492034223336518	6.568780020020924	3.052276765452358	5.6609599824388885	8.563711088033047	6.135013547002365	6.649371157347092	7.809800277545323	5.112887754296897	7.139817356747112	5.306382556527158	7.874581824063685	6.766701160409814	6.56870403007072	7.032464557526091	7.138548279559987	8.163513827366865	7.896507999335352	7.222823877205593	8.086836830396896	6.944425103812887	7.886141618529126	7.531927241876955	7.240257078394562	5.939254791827308	7.267816669293391	7.0744733247384355	6.782565826890818	8.683289410934997
TRIB1	11.933137368256713	11.053002695694357	10.413680812770478	10.349977801046785	9.083333055903674	11.298709859102624	11.095106872128353	10.800794766966806	12.101243635865217	11.670687226900233	10.935577610232617	10.783161956158645	11.263368976481726	11.003503857534144	12.030322282644036	12.513473473727935	10.979375175319799	11.02641278093799	10.91747651070519	10.048459627369782	11.441792611997545	12.022090344993964	10.953148812832245	13.234065956483473	10.548118688114206	10.460752800860506	10.407310258318192	12.504208220066136	12.281278059613978	10.583515323067472	10.24355484277846	11.286338279695164	12.18313289372495	12.402822883873172	11.420349758209937	11.717265144960065	11.391227475841095	9.972348020079574	11.112687102569124	11.874358964764365	11.584793424989078	10.609391155191943	11.365031955522811	11.280295205449832	11.021327032624372	10.962071785799944	11.352214508023993	12.265225313830463	8.989916699250687
WDYHV1	6.961449694398196	6.808938440998943	7.421677752332596	6.741520917917981	7.38005406594808	6.926035010922459	7.573094237771858	6.568805855692237	7.263438228782817	7.088756443434514	8.110848667992132	7.015481958431355	6.917515297521901	7.383220433365657	7.246493597458292	6.933490020832605	6.846643745560746	7.039401635407714	7.660352509670921	6.316268858604709	7.733923713129626	7.769242795140539	7.778103419866033	7.313037197452029	7.173377324365331	7.442893746886124	6.54878800761623	7.279703430520974	7.572321267976868	7.4083042624843	7.033478065182842	7.679726392268522	6.8573714520943	6.808166209864794	5.756345490179466	6.765256258126955	6.322607804781313	8.080934750143763	7.479157790723251	6.740792672277682	7.069347721172864	6.7239953447151874	7.81701561170431	6.214719328152916	6.404218524074025	7.235392000189692	6.694768783475156	6.062670045998985	7.1900616947391205
MAF1	12.159609635403108	10.980082440070449	12.160110925325146	11.447088393446247	12.27911141275405	11.597973382457722	11.52358645814244	11.78750266202185	11.846857610639498	11.617173570044088	12.018116703204022	10.506119110861166	11.622303536795284	11.297243012537958	11.441548838601525	11.574163019672714	12.047897550994263	11.42969441488778	10.699155980901335	10.69403570449897	11.57578503239116	11.096813965833453	10.29264372970759	11.168753588618669	10.570851862817477	11.098815085357595	11.334027413924064	11.142132591502087	10.5517659298235	11.163945228420697	10.610443543481898	10.970415109150553	11.303084342800782	11.713077148705565	11.355329070378188	10.94322541460092	11.438734684134895	11.357431041883679	11.52727661783299	12.042938394431909	11.553053252766412	11.457170513463446	11.720808569204296	10.68193235513752	11.373060887777195	11.364676384883232	11.390432315383864	11.599392188182755	12.770952898463428
ZNF250	7.807773500515264	6.7718459725075695	7.759608602769256	7.519290279036526	7.7400438143312105	6.807329159416136	7.620373802529742	7.153764810368253	7.8045763482295305	8.288506220454973	7.458340988088958	7.854749971464537	7.315212976029234	7.375387026828884	6.98154444975335	6.5741605592597345	7.34909099508231	7.023399525873634	6.483672306601474	7.039160332533351	7.386043534835717	7.391698992373825	6.862513382733515	7.064699658372092	7.713166527986755	7.708339003529728	7.640158154963282	7.26345700849489	7.526608781990978	8.354769496016864	7.573374526445944	7.630478280077936	7.172307487427958	7.0246299600257425	6.896405066678117	7.182712158269364	7.315421315945779	7.254310585473226	7.131352829350762	7.756816484529119	7.024585638278273	7.0030849683684515	7.386690257087597	7.617658465106395	6.911248160608	6.372596950693146	7.290350674720483	6.159752284303552	8.14226025572128
VPS28	11.799997933625912	9.823510547005368	10.862699340976599	10.405737143404707	10.812321728267245	10.71625358760593	11.335423853415866	11.000274705810554	10.71196804129201	10.327238248261425	11.366393254297995	9.59617670845584	11.034221218772245	10.58805871602521	10.641157631165298	11.516084111600966	11.4858393693017	10.080577724570256	10.274972116121383	10.622198278523646	11.43603479485248	11.639408545382144	10.026910691224519	10.885650621185578	10.119758195481051	10.799564951310487	10.355031685830841	10.761468125497776	10.841642966571547	10.617384813306256	10.315240152507242	9.493533258063378	11.462630074789038	10.894135655897585	10.545060995490715	10.64345939407657	10.723788912865714	11.821319581039976	11.331728725659268	10.754662063450471	10.847441073865982	10.87701566449191	11.049678410286393	10.603756144613369	11.076067338945943	11.253717506606227	10.866421208943008	11.728422248226863	11.064203842427633
MYC	11.725877459120227	9.67472545467707	11.151631805297994	11.176272899853634	11.121630643449437	11.341162970180184	11.521536645675441	10.470547032900692	12.2378403297578	11.707682666757828	12.048704828449962	11.741264725255283	11.785648628652078	11.870954162029932	12.583451858358577	11.266171624848033	11.246033920465814	10.33888120183432	11.935927457599576	10.423567666698442	11.433428958147912	8.801061627967208	12.678833380882335	11.676970796053913	10.509834373271655	10.241423118079828	11.844968547894156	12.268047761898341	10.360430123463399	11.018283649592737	11.517900633811825	11.812097064404226	11.876915934697514	11.378917974255156	10.317638723794467	10.571231207448328	11.395518069726515	10.786906690896208	11.157056345802138	13.78853482391184	11.313421596970663	11.25386520840484	12.003612776905104	10.504521084536304	10.975625887626439	10.218902226668359	10.913839346350143	11.582847628550267	11.707609338589833
ZFAT	8.208244114486282	8.016607744708448	8.146191883945713	8.257387842692651	8.056583528366367	8.122869398914256	8.442188785555434	7.678931067179552	8.360728591812295	8.554723138849026	7.694859304206259	7.855341933278342	7.460849025776118	7.67976860966357	7.740704988287578	8.171361971254113	7.765276151926686	7.917724131689021	7.4970694167239325	7.530453158085048	8.606190504451305	7.696021642606933	7.610803394891251	7.6458889570180135	8.13321415265704	8.097868849104906	8.121549057956951	7.760507061482602	9.098092632759109	8.221480788063534	7.498003481571745	8.104572546331953	7.606005385485257	8.39547844014663	8.385702811197598	8.134801064208192	8.177050719060368	6.509610341669188	8.592258933415648	8.310730918778589	8.580518624942268	8.365985546123467	8.515975737182153	7.632391828226337	8.088714048634602	7.11188682396893	8.287984734663656	7.418147773129103	8.287315295232888
NRBP2	8.527144639652294	7.057255300220513	9.890885915752124	8.760773164053345	9.749284523050763	10.095950784798582	9.460081095048768	11.249172724094395	9.729603750578512	9.696374682745516	8.07044836554298	8.16784963529398	8.539744302370327	8.684278747715284	8.066132255338625	8.4800792790707	9.04726299321152	8.374004839326151	7.299711728711222	8.45119874582184	9.745375166933785	9.980596596742018	8.660937171182784	9.107217075591384	9.93597090640252	8.978235678559809	10.35428244562383	9.312960078429496	7.177658721483943	9.671903685125805	9.698699458475597	10.150737362445991	8.688586024551869	9.056989879367793	8.341309770205438	8.585007584236758	8.726998059860648	7.391269370791259	8.7229611828139	10.061911173967093	8.896511026655272	9.08267864213078	8.773244725698035	8.69122356681367	8.696400766027912	7.863424349184006	9.221703112183093	8.376086308971495	10.724147164166611
SQLE	8.041719376738596	9.59627735899748	8.541673577981044	9.575091053075159	7.591836470201898	7.778096847328279	9.980939266085512	10.631340782294297	11.006992215568248	8.323081789503732	10.221720026746219	9.740158529497235	7.772604898426509	8.647789380869986	10.620494772948058	9.964395744037576	10.798431298145626	9.314977425106457	10.356297898451396	9.402067676309334	11.407294323187323	11.584652513407853	9.744159413322414	9.226905852031182	8.635086859838824	9.029566075040506	8.349431628433063	10.083200069007734	9.20553669317549	9.944237672359137	8.423120113185526	10.882299145680802	9.46653535819519	10.073860390886002	9.044057997838935	9.319227277844721	9.39602458982091	9.715526199662305	11.070167915178635	8.217807485019087	9.22814768128013	9.927143866410972	9.689678307445092	9.276701367905758	9.54606486463676	10.2955967648349	10.151384461684966	9.95725007712012	7.126797380444538
GPT	7.9287848310065625	6.941775374671109	3.3919326527962452	4.081424850303421	4.795086368750898	6.680014853182145	7.5289463208329845	4.740285380354498	2.8122312250333925	5.929279715975615	9.15429408780132	4.959626434858094	7.708601109810081	4.557244648057203	8.107452491657103	10.098361127332096	6.851286805326559	5.89445158751195	8.875021293928285	10.119576652637528	8.67714526775519	9.309996948892037	5.606083878827466	10.579825034229733	4.59673243545546	9.604808944264414	7.5720407745797385	4.673041891618141	1.3055620156204844	7.127468202199418	6.688250309133179	6.735698171023974	10.259072360431766	9.99589431721598	10.772454794755486	9.821048466708511	9.862313450885262	8.37371345475091	9.413376175813589	8.30672248840673	8.777436154517899	9.626221912968822	7.749111855521455	10.68348592897002	10.942426533447334	10.21266660465505	10.258907529438469	11.09025138818132	1.531867995605555
PTP4A3	9.151176406529608	8.873385665263715	10.76067837064057	9.020616095048329	10.86998704164261	8.804937672051908	9.779870346040221	11.012875906227068	12.16331003040554	10.010486146137938	10.194461391737482	9.426633769026692	8.749282846766324	9.088769691554003	9.078305612217763	9.021621302681725	11.602044633096416	9.004507010195345	8.33110504345726	8.559721231197235	7.994534469583742	8.960670978826148	7.258735614197153	8.061106528021588	8.224580566083445	8.334988309348535	9.890887435323016	9.770118753458066	7.619713785267435	7.465019423803677	7.64136539189785	10.099426715904844	8.95982810291661	9.860258281892545	7.756349493612215	8.801116611373416	8.82160818207442	7.566358532693305	9.543410816351782	9.886201075807836	9.918820015391427	9.09622758611378	9.910128514789363	7.338103463233793	9.046897535775164	9.54414338877213	8.530652130325056	7.948665085236818	11.536072332962792
EIF3H	12.941803245593823	11.610789556112023	12.519778746337982	11.841218373923889	11.975253288914075	11.922201565758005	12.065382473643615	12.015125787427047	12.807735270022762	12.593783258437314	13.335976470058942	11.977315704183216	12.386204703583942	12.009055287919576	12.263163547558898	12.223543219995388	12.45335295377561	12.242965658505106	12.274238361730388	12.570140322677341	12.851261034429402	11.627761852402616	12.413493066696224	12.666104143159389	12.393165186356503	12.522557012096085	12.967523486809622	12.20052164098932	11.902390189590555	12.213833283099293	11.824588873278344	12.285855879741469	12.519849987807987	12.10225131882592	12.073194299638365	11.944832829546014	11.939219230216013	13.07777373593746	12.278504632700397	12.326350833379493	12.53796576761128	12.099453016779735	12.70308598761767	11.954578021426057	11.997437082052652	12.287218893454533	11.806666418364134	12.282219695672588	12.856688131248752
RPL8	15.75870989643585	13.482152495638225	14.736930371689345	13.870037147465837	14.216291155757055	14.28413728331442	14.461620128772983	14.468942174529904	15.530028299522108	14.722978254208725	16.234673378907456	14.108091517972582	14.713171684314805	14.979996729474193	15.187031112092075	14.806474102052901	14.508859423796343	14.279037393448705	14.187576316900842	14.874409879621098	14.790093905006342	14.071118743009942	14.664024992222807	15.073074604923875	14.083512568770097	14.271156597751773	14.653572857500434	14.404981864149413	14.108916440217856	14.077258022476135	13.425753534898595	13.899328597480633	14.961531087885538	14.254379092820313	14.220378327695228	13.686194097030617	14.173036837344807	15.931462383061463	14.743040915130942	14.054595839474073	15.200860300179869	14.248943033928834	15.393423976920781	14.089624269385288	14.577871340857593	14.973329651019673	14.053459271349306	14.894197248283641	14.93909707234309
TRAPPC9	10.811238205052149	9.969069885192411	10.802257852368502	10.449891473010945	10.396144500890236	10.371504412043336	10.556189891635873	9.540517484607156	10.675260208766927	10.40984701026771	9.916982633529939	9.45518952061688	10.42882075651775	8.92308974059257	10.449602641043665	10.198653756700518	10.959342718989767	10.132474024365372	9.66372175454525	9.4095436839967	9.689678307445092	10.4062262682694	10.037176280599583	10.609492734900233	10.165020025512185	10.196073389792943	9.87747126524058	9.681219081994902	10.150128062614995	10.460507091108914	10.544211021820198	10.914437456933426	10.091739497128273	10.536412363243468	10.214756234890123	10.376602951924786	10.230632940529262	8.17178728007393	9.846226891733911	10.40685338142112	9.543810974566252	10.223072549672334	9.538982341648026	10.034895230721705	10.228301632127739	10.13790051765214	10.410642143521965	10.399374065365976	10.879476038991466
DENND3	9.766752500721072	8.658561400991168	8.786151985204379	8.797483204210625	9.146311551463254	9.950275933100814	9.515636768269784	8.986902937788074	6.961982105448787	8.534909795712794	7.249122936298274	8.752812136015136	8.507394276411784	9.015484746115881	9.88194445690957	9.134629104737705	8.569107490912556	8.73329671301624	8.706789534768403	8.309180735919693	8.869001530437426	9.45400311484372	7.521765309736007	9.365357909448957	10.27208152667558	9.971141152605897	8.530445354692906	8.794812228102423	9.993532847528288	8.9853040273419	10.357387052920107	9.799134268038848	8.902426369198738	9.602127240440998	8.707631768778059	9.353442393285718	9.222693488104175	7.414355439248254	9.540734443756092	10.336194109418852	10.117565185961606	8.934705200172562	9.725456583286746	8.54007765479962	9.394987800053858	8.86342125163339	9.539317809057705	8.187906278384292	9.706777449965092
EEF1D	13.675493991101009	11.853995647176394	13.204549748189516	12.305608639406891	13.104477541123464	12.177973835027235	12.386784007752722	12.403291691775122	13.348659004997193	12.118931342283494	14.03400794533537	11.239765581037647	12.500426136306313	12.187528378073447	12.82308655600977	12.742871497592432	12.880136406381324	12.286072546381309	11.924511910826093	12.778990848231608	12.70256880923831	12.430157187121536	11.92509448433672	12.363874661876547	12.434307651035892	12.368822903020186	12.883742990570445	12.163986097924068	11.633567437465361	12.300830138036963	11.74643454243159	11.567200315864639	12.538096759619922	12.809494834685147	12.239908752890978	12.179989928420483	12.364170229371291	13.200704005816352	12.562011021042633	12.647350490120028	13.234555507309043	12.367267307050634	13.46319226997292	11.801825547871559	12.673487965723071	13.168545203016626	12.457968201121881	12.740632476346955	13.605329036565534
ZNF572	4.519850086069655	5.252975696772798	5.0033593685697495	5.900398308759299	4.033246783747945	3.130848521868957	5.061339867542056	5.87098851948143	6.619587614260627	2.7779772219092616	5.102284393032211	1.8491986517647034	3.477651426165098	2.3608418180588777	3.899697691053051	1.9448209727224088	5.155482102156519	2.1218774438492187	5.333924582555388	4.258556612177268	6.1586096882144785	4.697295658582721	2.3518544391323792	3.531680886142242	5.681269338891766	5.7263189911643515	5.989634053907791	3.9140478160394165	1.6681191246864322	5.724775765465129	3.2161909807038613	6.69732902379372	4.85002427129923	3.79109514868741	3.21577177088915	5.1607992057739365	3.9576082022876315	4.68500383040852	4.637859444880041	4.622614141219763	5.089154893670463	3.8572644445006623	4.934790106484969	4.107805020767525	3.7895101704193928	5.097888859409488	4.3255375270361025	3.0906840901092454	4.3731540053857465
KHDRBS3	6.143289327331929	6.044866196854277	8.274569179748486	6.319289157636103	8.39843160859171	6.701604501497982	6.894223590070573	7.830673649131837	9.682597875762838	7.712341278039775	7.702435914226808	7.574646504018891	5.9322123893133645	6.496984762160978	6.546057917174112	6.863665937643798	8.940116819212228	8.070877657619715	5.903780642701515	6.330343270417228	7.474752389057915	7.699343408353099	7.690207548696169	6.1755325849916405	7.842042284133963	6.522785514500749	6.765959006045755	5.4230671540065	3.3561578989752414	5.9283087288124126	3.7538398321186346	6.82738716745774	6.900649481899411	6.528061785029515	6.3413079909006225	6.30097481002092	7.052763657209563	6.912805525784936	6.627803794578136	7.792543046908106	7.095702936654287	6.577152347940231	6.959039814632697	5.729291723988585	5.344097024245269	6.753243496463633	6.345438957261104	6.467060972575854	7.985807789971046
SNTB1	7.636827598531186	8.28228225647811	9.691644079689548	9.323611008558444	8.762352137985184	10.039864933458786	7.723114818095604	10.804147158621484	11.558956362638925	9.969314564682955	9.031896902574486	7.586989865465435	8.467593323802031	10.033863451866283	10.463595893207218	9.199831817616786	10.310294672093839	9.713489622273862	8.372843299842286	9.131684649841832	9.076040608263252	8.312007091534998	6.238553778266498	7.450543195540848	9.514362952338615	8.494579616427378	9.111002612657662	8.440026138347262	7.191661343078113	9.597754622693781	8.138507322993883	9.183729655970826	8.6699409199099	8.17685630356977	9.044057997838935	10.74145855941952	8.481173791884142	9.743137926651361	9.20094763377342	9.572266310728647	9.772340969472813	9.381744092542826	9.394222582736296	7.773686502614778	9.053950310779339	9.953378745497348	9.095193923454977	8.121564598406469	7.786093160384771
DGAT1	12.115222512444483	9.615956941733243	10.877330151769826	10.093536408512234	10.152487667421621	11.362146569954357	11.364205802857349	10.227880633817845	10.738016237387054	12.461131521425393	11.3538415342596	11.94778667827348	11.874631770563463	9.71633934031733	9.961349845448698	11.941792243978124	11.082255509383947	11.570709582861326	11.043437470312249	11.764647706893482	11.256733531146187	11.485084629562962	10.341218578007823	11.744619435068069	9.873820886643518	12.48223318384445	11.616291524901815	10.121896088124291	9.614685921677673	10.306699707272788	10.269944831663445	11.092380564224644	13.203410714187466	10.256763011022706	13.764799039592289	10.112804369954777	13.489132956991696	10.356418956227524	10.399737206350675	11.39129192910667	10.109726201987922	10.419612625503413	9.80959451262142	13.904832160281547	13.452575873915794	10.16799510846326	10.51934139402673	14.563964853227978	9.876317411112426
NSMCE2	8.32720680491085	7.075628893194297	7.993493221058417	8.230020435705635	7.591836470201898	7.853958232400762	7.859000470987619	8.58793874419156	8.855257827193197	8.4719921660893	8.804486003132515	7.2508573835483086	7.852042546912926	6.889631724104744	8.083489964675417	7.537265000971677	8.508626724259573	8.225732834193442	8.134826728043013	7.592210337749907	8.847909240130281	8.893395583622848	7.538367438753996	8.675060802199758	8.102889044859948	8.390616100861678	8.107473415670649	8.150249943169197	8.396022448461236	7.786289233789863	7.662867722440933	7.841539188796596	7.91127213280967	7.632399099233385	7.421677752332596	7.633547458294205	7.440694788175591	9.334921290896782	8.19925027026627	7.682917357062705	8.2793412837967	7.962092035430319	8.575709410525834	7.512400732803554	7.609688486528856	8.0888412292971	7.504342313147085	7.83732517195398	8.037003267099237
ZNF517	7.365683789747648	7.252977588423766	7.9224416870265015	7.8005893312392365	8.352034595194324	7.014924313227865	7.537070822569329	8.781661336410098	8.333830686643203	7.957113652743255	7.228299226772079	7.428695157332461	7.273506565117685	7.163901213814542	7.392944171965046	6.2667322044039055	7.837306247268102	6.752842235115747	6.166815851761945	6.947619406361266	7.272974964157956	7.557532627650661	5.864911644002322	6.18988977512699	7.923630553263172	7.847627419108087	8.157852169141737	6.934716993014772	8.058511324202152	7.914002356064278	7.64136539189785	8.65591348296378	6.473718735845443	7.113023243574772	6.936261022494529	7.077467334243472	7.417042353392818	6.319719081148039	7.014823914196164	8.07724299893246	7.5414141669581545	7.01297085583365	7.012144026041382	6.770762987054898	7.155830171556526	6.884488029741885	7.717854607174318	6.356029334031204	8.557915490212789
ZNF16	8.133763863309838	7.129396367102339	8.128891375137218	8.496889719515735	7.771779960078269	7.247955987406821	7.977216947341896	7.529766297954251	8.327929828648939	8.646436305382744	7.869840568488336	7.803401420442661	8.119366553117443	7.889625640518757	7.359125794762658	6.5741605592597345	7.181539905773607	8.028751466566508	7.498833254045011	7.803711384420392	7.951465404848316	7.719662616082799	7.637240715499948	7.655795446375894	7.250506871094968	7.8553606228536355	8.016245582599357	7.413280963571074	7.443623226461294	8.009055987952665	7.7007795437737965	7.519604804107185	7.4251403565629115	7.225323137169391	7.620535094166846	7.4102732561864455	7.726994655097833	7.829392938527099	7.462289584653541	7.992983640892445	7.477717797572208	7.388180925053965	7.762953355732874	7.928311097868984	7.404221930838794	6.925702781351336	7.547935601929803	7.055586128806361	8.076174256724249
ZHX2	10.25291374383274	9.762425227552777	10.299482791274672	9.314229749181738	10.1444925527918	9.100023627704497	8.884704751679209	8.407824337432233	9.577103996250912	9.42902169247321	9.38003457906647	7.425945984536924	8.859749078233968	8.730082875713821	9.609753115668148	9.185596510151939	10.080790884794618	9.110548559134022	9.721943362711396	9.37006181276497	8.766237358674118	8.659928125187529	8.040673292643762	8.198788791655197	10.035101497974088	9.896712329174093	9.272674103140892	9.50938112846196	9.803101079319074	9.630208869918382	9.873474873275729	10.306392126750834	9.177828119137136	9.552330956256231	9.236126636385178	9.324164791672676	9.31302812483981	8.486923490741443	9.033987304297222	9.770876936899903	9.90398575217991	8.845276767216971	9.775807783959749	8.928311097868983	9.378223315541081	8.995128323704366	9.24837116885969	8.633420311631138	9.66969649349295
SCXB	4.9542568092329775	3.434815015364693	6.784242222057782	4.4195928230306185	7.743306260880315	4.687239893984603	3.7051927735409116	4.836499825021359	5.766428198013189	5.306349743849723	5.852927697579384	3.296662967591957	2.629659699927392	4.097813029419907	4.733685146284154	4.529764736535991	4.740441904337232	2.2738739081606227	4.5433783348730135	6.151637639558463	3.7626783546169222	5.672493231905435	4.124427399594575	5.619140058336987	4.493640929952471	4.19111252785433	2.242389096267312	6.108430364902908	3.783582373940371	5.324238163242078	4.471441113049351	2.6856262412509113	4.483241808987515	5.46486753856108	3.926274643329601	1.8680150367944606	4.46780767624519	4.244430437805942	3.5553989890450217	7.006533665792763	5.8313579193569405	4.93972024481833	5.543999996223509	3.3459640303428717	3.489953901331306	0.7759354883017876	5.828039983872734	3.197598084380389	6.681603871658782
NDUFB9	12.308677477641432	10.589922621011405	11.466137656070432	10.668441828052998	11.190516096215559	11.066479418985079	11.497861814060748	11.529088874132164	12.215132532593739	11.799168274024643	12.283930451225308	10.877866922883126	11.73834986073552	11.526411207600878	12.136250996527163	11.96235742395474	11.830455786682307	11.442788024644216	11.766988477039865	11.532974368914196	12.662870837018064	11.440905494007072	11.440521425821094	12.09391928410816	10.96157268722804	11.418458783422825	11.0594391050047	11.196251706392328	10.373202302338958	10.926525799428543	9.914425497151484	10.633730893019289	12.1568130899718	11.202950258683998	11.315296768525586	11.127317294497558	11.235727059838059	13.28532418637116	11.746304367701304	10.720406734056896	11.65128045863437	10.98986987930072	11.892926017196576	11.608264064069003	11.866177762740106	12.567121865307866	10.86915425934924	12.63312238127581	11.80816781910941
HAS2	8.05105664084546	5.019777516085849	6.926877107387077	6.2880207370853105	4.447969240094467	6.451758407727415	5.282636253185752	5.233332067686572	6.865943551909418	5.225648976272745	6.78984660249015	5.790652155764443	6.763809907280294	6.382994000691125	7.446686327138151	6.650233214102979	6.312726881718568	6.732513427424199	5.691478331987407	5.898399441308833	4.964643527615172	4.070749956574913	5.351840308293695	7.058327320092839	7.201212360154221	5.616713503777762	4.861310273741442	7.457027857866142	8.622132373783307	4.914578873837431	5.745804723624433	5.96224592333346	7.659689131083324	7.202780113023795	6.637398683353243	6.704844305160521	6.5426449868862795	7.031174610821703	6.198164087607665	8.228005743287879	7.861887147068501	6.315225658449346	7.214843641817592	6.051219818875949	5.111436178661517	5.890743372885765	5.792238483984418	7.745775149485796	6.51492073029804
GLI4	8.429649437016227	6.803808234507649	8.134472526795445	7.352908562606697	7.384611091140822	7.717964247995588	8.229732108136714	8.186109286073771	7.420709763278405	7.974529312483882	7.933389693308577	6.9959932166189915	7.445428759238323	8.164916980264637	8.576763977673126	8.000033812768779	8.895396543734362	6.897845456005512	7.378537633146205	7.6012062258446	8.039039665959507	8.276054595760726	7.161575333299709	6.439047939400388	6.982594354827899	7.722179096358366	8.011434138714973	7.779745615564987	6.2809804327595025	8.031654341094214	7.190150610638223	6.91744369027655	7.151189093678589	7.473941106826723	7.087441624990795	7.080977374158919	7.3086211700336765	8.195697007930443	7.668636833557682	8.04885192909681	7.850361888612105	7.696745021166909	8.357389252400141	6.819016677992507	7.237917389158097	7.553775187834172	7.615048396081995	7.76767465899052	8.419315559867732
CYC1	13.01408674346819	11.146524127786641	11.713605609220302	11.849483293681898	11.314220684065992	11.49434060142669	12.612085212673644	11.801166741476356	12.28428913415251	11.838250601557187	12.52174519267827	11.561731856475172	12.466540456228659	12.039888916733409	12.363042368854979	12.63501972681459	11.814734657799555	12.350013726207532	11.248686626873827	11.383833870041695	12.18695917607294	11.338012068134946	11.628646027096082	12.128683916503444	10.750330144924087	11.529147453596233	11.898060936813375	10.217133757650728	10.111513904159061	10.905890882436424	10.49680385628072	11.274983760008189	12.287882607521022	11.394548439167169	11.99236128433921	11.040940362648369	11.80043887549639	13.015920255661639	12.655593331005367	11.2178062735616	11.853001487627079	11.56319627221674	12.143580917269825	12.00059865057415	12.571278618514595	12.144467060327345	11.457108937238068	12.945313973347853	11.92184093707449
... (truncated at 50 columns)
